# Supplementary material for: Dimethyl sulfate and diisopropyl sulfate as practical and versatile O-sulfation reagents
Source: Nat Commun. 2024 Feb 29;15:1861. doi: 10.1038/s41467-024-46214-x (PMC10904734; doi:10.1038/s41467-024-46214-x)
Supplement: Supplementary file 1 — Supplementary Information [file 41467_2024_46214_MOESM1_ESM.pdf]

SUPPLEMENTARY INFORMATION

Dimethyl sulfate and diisopropyl sulfate as practical and versatile *O*-sulfation reagents

Shuaishuai Yue,[1‡] Guoping Ding,[2,3‡] Ye Zheng,[1] Chunlan Song,[1*] Peng Xu[2*], Biao Yu,[2] and Jiakun Li [1*]

[1]State Key Laboratory of Chemo/Biosensing and Chemometrics, College of Chemistry and Chemical Engineering, Hunan University, Changsha 410082, P. R. China

[2]State Key Laboratory of Chemical Biology, Shanghai Institute of Organic Chemistry, Chinese Academy of Sciences 345 Lingling Road, Shanghai 200032, P. R. China

[3]Key Laboratory of Structure-based Drug Design & Discovery (Ministry of Education), School of Pharmaceutical Engineering, Shenyang Pharmaceutical University, 110016, P. R. China

*E-mail: songcl@hnu.edu.cn; peterxu@sioc.ac.cn; jkli@hnu.edu.cn

CONTENTS

Supplementary Notes 3

Supplementary Methods 3

1. General procedure for sulfation reactions 3

2. Screening reaction conditions for the sulfation of alcohols 5

3. Screening reaction conditions for the sulfation of phenols 7

4. Characterization data 8

5. Gram scale synthesis 52

6. Mechanistic experiments 53

7. X-ray crystallographic data 60

8. Spectroscopic data 63

Supplementary References 137

Supplementary Notes

All glasswares were oven dried at 110 °C for several hours and cooled down under vacuum. Unless otherwise noted, materials were obtained from commercial suppliers and used without further purification. The sulfation reactions were performed in 4.0 mL (15 x 45 mm) glass vials on Synthware H221520 heating block (15.3 x 20 mm) for heat transmission. Flash chromatography columns were packed with 200 mesh silica gel in petroleum (bp. 60-90 °C). ^{1}H, ^{13}C and ^{19}F NMR data were recorded with Bruker Advance III (400 MHz, 500 MHz or 600 MHz) spectrometers with tetramethylsilane as an internal standard. Chemical shifts are reported in ppm with the solvent resonance as the internal standard. For ^{1}H NMR: Chloroform-*d*, 7.26; Methanol-d_4, 3.31. For ^{13}C NMR: Chloroform-*d*, 77.16; Methanol-d_4, 49.00. Data is reported as follows: s = singlet, d = doublet, t = triplet, q = quartet, m = multiplet, br = broad; coupling constants in Hz. High resolution mass spectra (HRMS) were measured on a Finnigan-MAT95XP equipped with EI ion source and double-focusing mass analyzer, or a Bruker APEX III 7.0 Tesla FTMS with ESI ion source.

Supplementary Methods

1. General procedure for sulfation reactions

1.1 General procedure A:

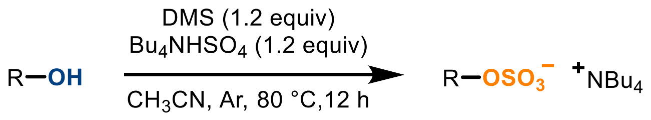

To a 4.0 mL borosilicate vial equipped with a stir bar was added alcohols (0.2 mmol, 1.0 equiv), dimethyl sulfate (30.3 mg, 0.24 mmol, 1.2 equiv) and tetrabutylammonium hydrogen sulfate (81.5 mg, 0.24 mmol, 1.2 equiv). The vial was evacuated and backfilled with argon for three times, then CH_3CN (1.0 mL, c = 0.2 M) was added. After stirring for 12 h at 80 °C, dibromomethane (14.0 μL, 0.2 mmol, 1.0 equiv) was added as an internal standard. The reaction mixture was diluted with $CDCl_3$, and the yield of tetrabutylammonium sulfates was determined by ^{1}H NMR integration relative to the internal standard (standard: δ 4.94 ppm). Once NMR signals of the sulfates products were not easily discernible, the reaction mixture was loaded onto a short plug of silica gel eluting with PE/EA (1/1, v/v) to DCM/MeOH (10:1, v/v). The crude sulfate salts were diluted with $CDCl_3$ and dibromomethane (14.0 μL, 0.2 mmol, 1.0 equiv) was

added as an internal standard. The yield of tetrabutylammonium sulfates was determined by ^1H NMR integration relative to the internal standard (standard: δ 4.94 ppm). To isolate the products, the concentrated reaction mixture or crude sulfate salts were purified by flash chromatography on silica gel eluting with DCM/MeOH (100:1 to 20:1, v/v) or plate chromatography on silica gel eluting with DCM/MeOH (30:1, v/v) to afford the product tetrabutylammonium sulfate salts.

1.2 General procedure B:

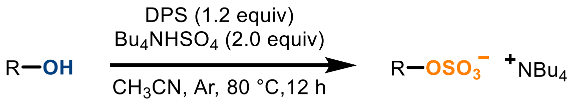

To a 4.0 mL borosilicate vial equipped with a stir bar was added alcohols (0.2 mmol, 1.0 equiv), diisopropyl sulfate (43.7 mg, 0.24 mmol, 1.2 equiv) and tetrabutylammonium hydrogen sulfate (135.8 mg, 0.4 mmol, 2.0 equiv). The vial was evacuated and backfilled with argon for three times, then CH_3CN (1.0 mL, c = 0.2 M) was added. After stirring for 12 h at 80 °C, dibromomethane (14.0 μL, 0.2 mmol, 1.0 equiv) was added as an internal standard. The reaction mixture was diluted with $CDCl_3$, and the yield of tetrabutylammonium sulfates was determined by ^1H NMR integration relative to the internal standard (standard: δ 4.94 ppm). Once NMR signals of the sulfates products were not easily discernible, the reaction mixture was loaded onto a short plug of silica gel eluting with PE/EA (1/1, v/v) to DCM/MeOH (10:1, v/v). The crude sulfate salts were diluted with $CDCl_3$ and dibromomethane (14.0 μL, 0.2 mmol, 1.0 equiv) was added as an internal standard. The yield of tetrabutylammonium sulfates was determined by ^1H NMR integration relative to the internal standard (standard: δ 4.94 ppm). To isolate the products, the concentrated reaction mixture or crude sulfate salts were purified by flash chromatography on silica gel eluting with DCM/MeOH (100:1 to 20:1, v/v) or plate chromatography on silica gel eluting with DCM/MeOH (30:1, v/v) to afford the product tetrabutylammonium sulfate salts.

1.3 General procedure C:

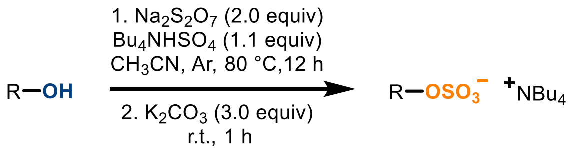

To a 4.0 mL borosilicate vial equipped with a stir bar was added phenols (0.2 mmol, 1.0 equiv), sodium pyrosulfate (88.8 mg, 0.4 mmol, 2.0 equiv) and tetrabutylammonium hydrogen sulfate (74.7 mg, 0.22 mmol, 1.1 equiv). The vial was evacuated and

backfilled with argon for three times, then CH_3CN (2.0 mL, c = 0.1 M) was added. After stirring for 12 h at 80 °C, the reaction flask was cooled to room temperature, anhydrous potassium carbonate (82.9 mg, 0.6 mmol, 3.0 equiv) was added and stirring was continued at room temperature for 1 h. Then dibromomethane (14.0 μL, 0.2 mmol, 1.0 equiv) was added as an internal standard. The reaction mixture was diluted with $CDCl_3$, and the yield of tetrabutylammonium sulfates was determined by ^1H NMR integration relative to the internal standard (standard: δ 4.94 ppm). Once NMR signals of the sulfates products were not easily discernible, the reaction mixture was loaded onto a short plug of silica gel eluting with PE/EA (1/1, v/v) to DCM/MeOH (10:1, v/v). The crude sulfate salts were diluted with $CDCl_3$ and dibromomethane (14.0 μL, 0.2 mmol, 1.0 equiv) was added as an internal standard. The yield of tetrabutylammonium sulfates was determined by ^1H NMR integration relative to the internal standard (standard: δ 4.94 ppm). To isolate the products, the concentrated reaction mixture or crude sulfate salts were purified by flash chromatography on silica gel eluting with DCM/MeOH (100:1 to 20:1, v/v) or plate chromatography on silica gel eluting with DCM/MeOH (30:1, v/v) to afford the product tetrabutylammonium sulfate salts.

Note: Alkyl sulfate is highly toxic, so the reaction process should be handled with care. It is recommended to work in a fume hood.

2. Screening reaction conditions for the sulfation of alcohols

Supplementary Table 1. Optimization of promoters

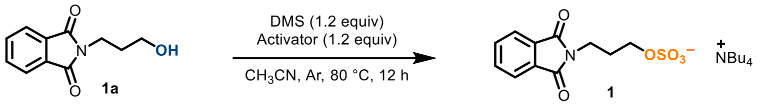

Entry	Activator	Yield of **1** (%)[a]	Entry	Activator	Yield of **1** (%)[a]
1	Bu_4NHSO_4	84%	9	Bu_4NI	49%
2	$KHSO_4$	n.d.	10	Bu_4NI, $KHSO_4$	66%
3	$NaHSO_4$	44%[b]	11	Bu_4NOAc	58%
4	NH_4HSO_4	28%[c]	12	Bu_4NOAc ,$KHSO_4$	79%
5	Bu_4NCl	46%	13	Bu_4NOAc, HOAc	30%
6	Bu_4NPF_6	30%	14	Bu_4NBF_4	21%
7	Bu_4NNO_2	49%	15	Bu_4NBF_4, $KHSO_4$	62%
8	$Bu_4NH_2PO_4$	36%			

[a] Yields were determined by ^1H NMR using dibromomethane as an internal standard. [b] The product was sodium alkyl sulfate salt. [c] The product was alkyl ammonium sulfate salt.

The potential release of conjugated acid (HX) from the equilibrium between the substrate ROH and activator Bu_4NX ($X \neq HSO_4^-$) may indeed promote sulfation. This

hypothesis can be verified by the decreased pH over time for the solution of **1a** and Bu_4NOAc.

*p*H over time for the mixture of **1a** and Bu_4NOAc (1.0 equiv) in CH_3CN (1M) at 80 °C

Time	*p*H indicator paper	*p*H meter
1 h	6.7	11.38
3 h	6.4	10.22
7 h	6.2	10.02
12 h	6.0	9.01

Supplementary Table 2. Optimization of dialkyl sulfates

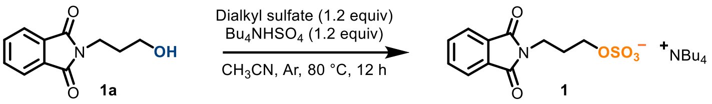

Entry	Variation from the standard conditions	Yield of **1** (%)[a]
1	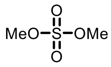	84%
2	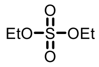	74%
3	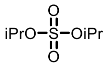	87%
4	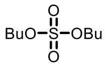	57%
5	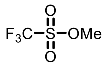	n.d.
6	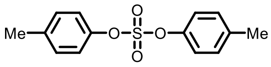	n.d.
7	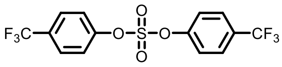	n.d.

[a] Yields were determined by ^{1}H NMR using dibromomethane as an internal standard.

Supplementary Table 3. Optimization of other alkyl ammonium bisulfates

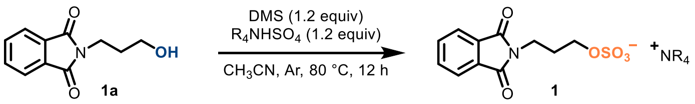

Entry	R_4NHSO_4	Yield of **1** (%)[a]
1	Me_4NHSO_4	76%
2	Et_4NHSO_4	75%
3	$^nPro_4NHSO_4$	74%
4	$^nBu_4NHSO_4$	84%
5	$^nHex_4NHSO_4$	70%

[a] Yields were determined by ^{1}H NMR using dibromomethane as an internal standard.

Supplementary Table 4. Unsuccessful substrates for *N*-sulfation

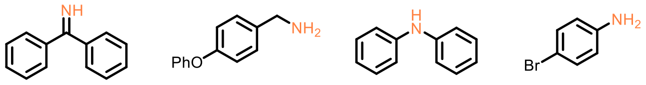

all failed with no conversion

3. Screening reaction conditions for the sulfation of phenols

3.1 Supplementary Table 5. Optimization of reaction conditions

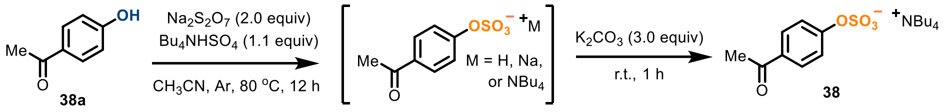

Entry	Variation from the standard conditions	Yield of **38** (%)[a]
1	None	82%
2	$NaHCO_3$ instead of K_2CO_3	52%
3	$PhCO_2Na$ instead of K_2CO_3	76%
4	NaOAc instead of K_2CO_3	75%
5	NaH_2PO_4 instead of K_2CO_3	76%
6	Na_2HPO_4 instead of K_2CO_3	52%
7	NaOH instead of K_2CO_3	49%
8	NaOtBu instead of K_2CO_3	77%
9	Bu_4NOAc instead of Bu_4NHSO_4	n.d.
10	Bu_4NI instead of Bu_4NHSO_4	n.d.
11	Bu_4NBF_4 instead of Bu_4NHSO_4	n.d.
12	$K_2S_2O_7$ instead of $Na_2S_2O_7$	n.d.

[a] Yields were determined by ^{1}H NMR using dibromomethane as an internal standard.

3.2 Supplementary Figure 1. The mechanistic purpose for sulfation with $Na_2S_2O_7$.

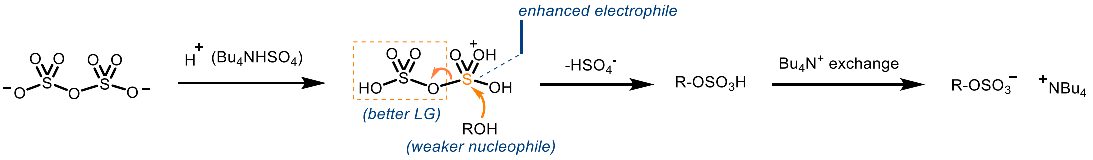

The addition of a base (K_2CO_3) to neutralize the protonic acid in the system could prevent the hydrolysis of the desired aryl sulfate in an acidic environment,[6] and thus enhance the stability of the final product as tetrabutylammonium arylsulfates. $Na_2S_2O_7$, possesses a stronger electrophilic sulfur atom and a better leaving group (SO_4^{2-}). These unique properties makes it more favorable for the sulfation to occur, especially when dealing with challenging substrates.

4. Characterization Data

Tetrabutylammonium 3-phthalimido-1-propanol sulfate (1)

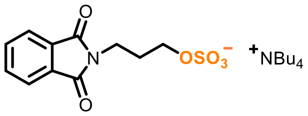

The reaction was carried out according to general procedure **A**. The yield of tetrabutylammonium 3-phthalimido-1-propanol sulfate **1** was purified by plate chromatography on silica gel eluting with DCM/MeOH (30:1, v/v) to afford the product as a light yellow oil (84.3 mg, 0.160 mmol, 80%).

R*f* = 0.40 (DCM/MeOH = 15:1, v/v).

NMR Spectroscopy:

^{1}H NMR (400 MHz, Chloroform-*d*, 298 K, δ): 7.77 - 7.72 (m, 2H), 7.68 - 7.62 (m, 2H), 4.03 (t, *J* = 6.5 Hz, 2H), 3.73 (t, *J* = 7.5 Hz, 2H), 3.32 - 3.18 (m, 8H), 1.97 (p, *J* = 6.7 Hz, 2H), 1.68 - 1.56 (m, 8H), 1.38 (h, *J* = 7.4 Hz, 8H), 0.93 (t, *J* = 7.3 Hz, 12H) ppm.

^{13}C NMR (101 MHz, Chloroform-*d*, 298 K, δ): 168.2, 133.9, 132.1, 123.1, 64.6, 58.6, 35.5, 28.8, 23.9, 19.7, 13.7 ppm.

HRMS *m/z* (ESI-) calculated for $C_{11}H_{10}NO_6S^-$, 284.0234, found, 284.0233 $[M\text{-}NBu_4]^-$.

Tetrabutylammonium 3-bromophenylethanol sulfate (2)

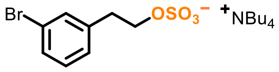

The reaction was carried out according to general procedure **A**. The yield of tetrabutylammonium 3-bromophenylethanol sulfate **2** was purified by flash chromatography on silica gel eluting with DCM/MeOH (100:1 to 20:1, v/v) to afford the product as a light yellow oil (69.9 mg, 0.134 mmol, 67%).

R*f* = 0.40 (DCM/MeOH = 15:1, v/v).

NMR Spectroscopy:

^{1}H NMR (400 MHz, Chloroform-*d*, 298 K, δ): 7.31 (s, 1H), 7.22 (d, *J* = 7.8 Hz, 1H), 7.11 (d, *J* = 7.8 Hz, 1H), 7.05 (t, *J* = 7.7 Hz, 1H), 4.11 (t, *J* = 7.2 Hz, 2H), 3.21 - 3.05 (m, 8H), 2.88 (t, *J* = 7.2 Hz, 2H), 1.59 - 1.46 (m, 8H), 1.31 (h, *J* = 7.3 Hz, 8H), 0.89 (t, *J* = 7.4 Hz, 12H) ppm.

^{13}C NMR (101 MHz, Chloroform-*d*, 298 K, δ): 141.2, 131.8, 129.8, 129.1, 127.7, 122.0, 66.8, 58.4, 35.6, 23.8, 19.6, 13.6 ppm.

HRMS *m/z* (ESI-) calculated for $C_8H_8BrO_4S^-$, 278.9332, found, 278.9332 $[M\text{-}NBu_4]^-$

Tetrabutylammonium 4-fluorophenylethanol sulfate (3)

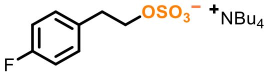

The reaction was carried out according to general procedure **A**. The yield of tetrabutylammonium 4-fluorophenylethanol sulfate **3** was purified by plate chromatography on silica gel eluting with DCM/MeOH (30:1, v/v) to afford the product as a light yellow oil (65.6 mg, 0.142 mmol, 71%).

$\mathbf{R_f}$ = 0.40 (DCM/MeOH = 15:1, v/v).

NMR Spectroscopy:

^{1}**H NMR** (400 MHz, Chloroform-*d*, 298 K, δ): 7.13 (dd, *J* = 8.4, 5.6 Hz, 2H), 6.85 (t, *J* = 8.7 Hz, 2H), 4.10 (t, *J* = 7.4 Hz, 2H), 3.22 - 3.08 (m, 8H), 2.88 (t, *J* = 7.4 Hz, 2H), 1.57 - 1.48 (m, 8H), 1.32 (h, *J* = 7.3 Hz, 8H), 0.90 (t, *J* = 7.4 Hz, 12H) ppm.

^{13}**C NMR** (101 MHz, Chloroform-*d*, 298 K, δ): 161.3 (d, *J* = 243.2 Hz), 134.4 (d, *J* = 3.2 Hz), 130.4 (d, *J* = 7.8 Hz), 114.8 (d, *J* = 21.2 Hz), 67.3, 58.5, 35.1, 23.8, 19.6, 13.6 ppm.

^{19}**F NMR** (376 MHz, Chloroform-*d*, 298 K, δ): -117.58 ppm.

HRMS *m/z* (ESI-) calculated for $C_8H_8FO_4S^-$, 219.0133, found, 219.0132 $[M\text{-}NBu_4]^-$.

Tetrabutylammonium 4-nitrophenylethanol sulfate (4)

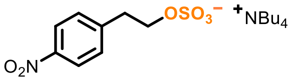

The reaction was carried out according to general procedure **A**. The yield of tetrabutylammonium 4-nitrophenylethanol sulfate **4** was purified by plate chromatography on silica gel eluting with DCM/MeOH (30:1, v/v) to afford the product as a yellow oil (77.9 mg, 0.159 mmol, 80%).

$\mathbf{R_f}$ = 0.30 (DCM/MeOH = 15:1, v/v).

NMR Spectroscopy:

^{1}**H NMR** (400 MHz, Chloroform-*d*, 298 K, δ): 8.06 (d, *J* = 8.7 Hz, 2H), 7.42 (d, *J* = 8.7 Hz, 2H), 4.23 (t, *J* = 6.8 Hz, 2H), 3.26 - 3.14 (m, 8H), 3.07 (t, *J* = 6.8 Hz, 2H), 1.64 - 1.52 (m, 8H), 1.37 (h, *J* = 7.3 Hz, 8H), 0.94 (t, *J* = 7.3 Hz, 12H) ppm.

^{13}**C NMR** (101 MHz, Chloroform-*d*, 298 K, δ): 147.3, 146.4, 130.0, 123.4, 66.4, 58.7, 35.8, 23.9, 19.7, 13.7 ppm.

HRMS *m/z* (ESI-) calculated for $C_8H_8NO_6S^-$, 246.0078, found, 246.0075 $[M\text{-}NBu_4]^-$

Tetrabutylammonium 4-cyanophenylethanol sulfate (5)

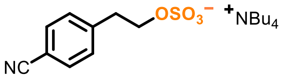

The reaction was carried out according to general procedure **A**. The yield of tetrabutylammonium 4-cyanophenylethanol sulfate **5** was purified by plate chromatography on silica gel eluting with DCM/MeOH (30:1, v/v) to afford the product as a light yellow oil (67.5 mg, 0.144 mmol, 72%).

$\mathbf{R}_f$ = 0.40 (DCM/MeOH = 15:1, v/v).

NMR Spectroscopy:

^{1}**H NMR** (400 MHz, Chloroform-*d*, 298 K, δ): 7.53 (d, *J* = 8.2 Hz, 2H), 7.39 (d, *J* = 8.2 Hz, 2H), 4.25 (t, *J* = 6.9 Hz, 2H), 3.27 - 3.20 (m, 8H), 3.06 (t, *J* = 6.9 Hz, 2H), 1.67 - 1.56 (m, 8H), 1.41 (h, *J* = 7.4 Hz, 8H), 0.98 (t, *J* = 7.3 Hz, 12H) ppm.

^{13}**C NMR** (101 MHz, Chloroform-*d*, 298 K, δ): 145.0, 132.1, 130.1, 119.4, 109.9, 66.7, 58.9, 36.1, 24.1, 19.8, 13.8 ppm.

HRMS *m/z* (ESI-) calculated for $C_9H_8NO_4S^-$, 226.0180, found, 226.0178 $[M\text{-}NBu_4]^-$.

Tetrabutylammonium 4-tertbutylphenylethanol sulfate (6)

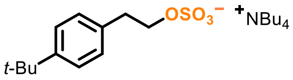

The reaction was carried out according to general procedure **A**. The yield of tetrabutylammonium 4-tertbutylphenylethanol sulfate **6** was purified by plate chromatography on silica gel eluting with DCM/MeOH (30:1, v/v) to afford the product as a light yellow oil (81.3 mg, 0.163 mmol, 81%).

$\mathbf{R}_f$ = 0.40 (DCM/MeOH = 15:1, v/v).

NMR Spectroscopy:

^{1}**H NMR** (400 MHz, Chloroform-*d*, 298 K, δ): 7.23 (d, *J* = 8.3 Hz, 2H), 7.12 (d, *J* = 8.3 Hz, 2H), 4.14 (t, *J* = 7.8 Hz, 2H), 3.27 - 3.14 (m, 8H), 2.92 (t, *J* = 7.8 Hz, 2H), 1.62 - 1.50 (m, 8H), 1.36 (h, *J* = 7.4 Hz, 8H), 1.24 (s, 9H), 0.92 (t, *J* = 7.3 Hz, 12H) ppm.

^{13}**C NMR** (101 MHz, Chloroform-*d*, 298 K, δ): 148.8, 135.4, 128.6, 125.1, 67.5, 58.5, 35.5, 34.3, 31.4, 23.9, 19.6, 13.6 ppm.

HRMS *m/z* (ESI-) calculated for $C_{12}H_{17}O_4S^-$, 257.0853, found, 257.0852 $[M\text{-}NBu_4]^-$.

Tetrabutylammonium 4-methoxyphenylethanol sulfate (7)

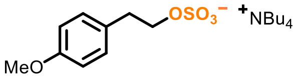

The reaction was carried out according to general procedure **A**. The yield of tetrabutylammonium 4-methoxyphenylethanol sulfate **7** was purified by plate chromatography on silica gel eluting with DCM/MeOH (30:1, v/v) to afford the product as a light yellow oil (77.7 mg, 0.164 mmol, 82%).

R*f* = 0.40 (DCM/MeOH = 15:1, v/v).

NMR Spectroscopy:

^{1}H NMR (400 MHz, Chloroform-*d*, 298 K, δ): 7.13 (d, *J* = 8.5 Hz, 2H), 6.77 (d, *J* = 7.5 Hz, 2H), 4.15 (t, *J* = 7.6 Hz, 2H), 3.74 (s, 3H), 3.27 - 3.17 (m, 8H), 2.92 (t, *J* = 7.6 Hz, 2H), 1.65 - 1.52 (m, 8H), 1.45 - 1.34 (m, 8H), 0.96 (t, *J* = 7.1 Hz, 12H) ppm.

^{13}C NMR (101 MHz, Chloroform-*d*, 298 K, δ): 158.0, 130.8, 130.0, 113.8, 67.8, 58.7, 55.3, 35.3, 24.0, 19.8, 13.8 ppm.

HRMS *m/z* (ESI-) calculated for $C_9H_{11}O_5S^-$, 231.0333, found, 231.0332 $[M\text{-}NBu_4]^-$.

Tetrabutylammonium 2,6-dichlorophenethanol sulfate (8)

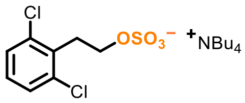

The reaction was carried out according to general procedure **A**. The yield of tetrabutylammonium 2,6-dichlorophenethanol sulfate **8** was purified by flash chromatography on silica gel eluting with DCM/MeOH (100:1 to 20:1, v/v) to afford the product as a white solid (81.8 mg, 0.160 mmol, 80%).

R*f* = 0.40 (DCM/MeOH = 15:1, v/v).

NMR Spectroscopy:

^{1}H NMR (400 MHz, Chloroform-*d*, 298 K, δ): 7.20 (d, *J* = 8.0 Hz, 2H), 7.06 - 7.00 (m, 1H), 4.09 - 4.03 (m, 2H), 3.36 - 3.26 (m, 2H), 3.26 - 3.17 (m, 8H), 1.64 - 1.52 (m, 8H), 1.36 (h, *J* = 7.4 Hz, 8H), 0.92 (t, *J* = 7.3 Hz, 12H) ppm.

^{13}C NMR (101 MHz, Chloroform-*d*, 298 K, δ): 136.0, 134.1, 128.13, 128.09, 63.8, 58.5, 31.8, 23.9, 19.7, 13.7 ppm.

HRMS *m/z* (ESI-) calculated for $C_8H_7Cl_2O_4S^-$, 268.9448, found, 268.9445 $[M\text{-}NBu_4]^-$.

Tetrabutylammonium 1-naphthaleneethanol sulfate (9)

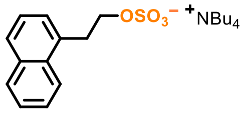

The reaction was carried out according to general procedure **A** with modified conditons: 2.0 equiv Bu_4NHSO_4 was used. The yield of tetrabutylammonium 1-naphthaleneethanol sulfate **9** was purified by plate chromatography on silica gel eluting

with DCM/MeOH (30:1, v/v) to afford the product as a light yellow oil (79.9 mg, 0.192 mmol, 81%).

R*f* = 0.40 (DCM/MeOH = 15:1, v/v).

NMR Spectroscopy:

^{1}H NMR (400 MHz, Chloroform-*d*, 298 K, δ): 8.13 (d, *J* = 8.3 Hz, 1H), 7.81 (d, *J* = 7.9 Hz, 1H), 7.69 (d, *J* = 8.0 Hz, 1H), 7.51 - 7.40 (m, 3H), 7.36 (t, *J* = 7.6 Hz, 1H), 4.37 (t, *J* = 7.6 Hz, 2H), 3.50 (t, *J* = 7.5 Hz, 2H), 3.23 - 3.18 (m, 8H), 1.62 - 1.56 (m, 8H), 1.41 - 1.36 (m, 8H), 0.97 (t, *J* = 7.8 Hz, 12H) ppm.

^{13}C NMR (101 MHz, Chloroform-*d*, 298 K, δ): 134.9, 133.9, 132.4, 128.7, 126.9, 126.0, 125.7, 125.5, 124.2, 67.0, 58.8, 33.3, 24.1, 19.8, 13.8 ppm.

HRMS *m/z* (ESI-) calculated for $C_{12}H_{11}O_4S^-$, 251.0384, found, 251.0381 $[M-NBu_4]^-$.

Tetrabutylammonium 2,4,6-trimethylphenylethanol sulfate (10)

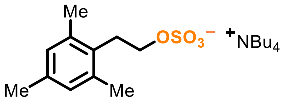

The reaction was carried out according to general procedure **A** with modified conditons: 2.0 equiv Bu_4NHSO_4 was used. The yield of tetrabutylammonium 2,4,6-trimethylphenylethanol sulfate **10** was purified by plate chromatography on silica gel eluting with DCM/MeOH (30:1, v/v) to afford the product as a light yellow oil (87.2 mg, 0.180 mmol, 90%).

R*f* = 0.40 (DCM/MeOH = 15:1, v/v).

NMR Spectroscopy:

^{1}H NMR (400 MHz, Chloroform-*d*, 298 K, δ): 6.77 (s, 2H), 4.02 - 3.95 (m, 2H), 3.26 - 3.21 (m, 8H), 3.04 - 2.98 (m, 2H), 2.29 (s, 6H), 2.19 (s, 3H), 1.63 - 1.57 (m, 8H), 1.39 (h, *J* = 7.4 Hz, 8H), 0.96 (t, *J* = 7.3 Hz, 12H) ppm.

^{13}C NMR (101 MHz, Chloroform-*d*, 298 K, δ): 136.9, 135.4, 131.7, 128.8, 65.3, 58.7, 30.0, 24.0, 20.8, 19.9, 19.7, 13.7 ppm.

HRMS *m/z* (ESI-) calculated for $C_{11}H_{15}O_4S^-$, 243.0697, found, 243.0696 $[M-NBu_4]^-$.

Tetrabutylammonium 4-pentenol sulfate (11)

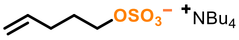

The reaction was carried out according to general procedure **B**. 1,3,5-trimethoxybenzene (33.6 mg, 0.2 mmol, 1.0 equiv) was added as an internal standard. The yield of tetrabutylammonium 4-pentenol sulfate **11** was determined by ^{1}H NMR integration relative to the internal standard (92% yield; standard: δ 3.75 ppm, **11**: δ

4.00 (t, J = 6.7 Hz) ppm). Then the reaction was purified by plate chromatography on silica gel eluting with DCM/MeOH (30:1, v/v) to afford the product as a light yellow oil (57.3 mg, 0.141 mmol, 70%).

R*f* = 0.40 (DCM/MeOH = 15:1, v/v).

NMR Spectroscopy:

^{1}H NMR (400 MHz, Chloroform-*d*, 298 K, δ): 5.84 - 5.64 (m, 1H), 4.98 - 4.80 (m, 2H), 3.93 (t, J = 6.8 Hz, 2H), 3.26 - 3.15 (m, 8H), 2.11 - 2.02 (m, 2H), 1.72 - 1.62 (m, 2H), 1.61 - 1.52 (m, 8H), 1.36 (h, J = 7.4 Hz, 8H), 0.92 (t, J = 7.4 Hz, 12H) ppm.

^{13}C NMR (101 MHz, Chloroform-*d*, 298 K, δ): 138.2, 114.5, 66.5, 58.5, 30.1, 28.9, 23.9, 19.6, 13.6 ppm.

HRMS *m/z* (ESI-) calculated for $C_5H_9O_4S^-$, 165.0227, found, 165.0211 $[M\text{-}NBu_4]^-$.

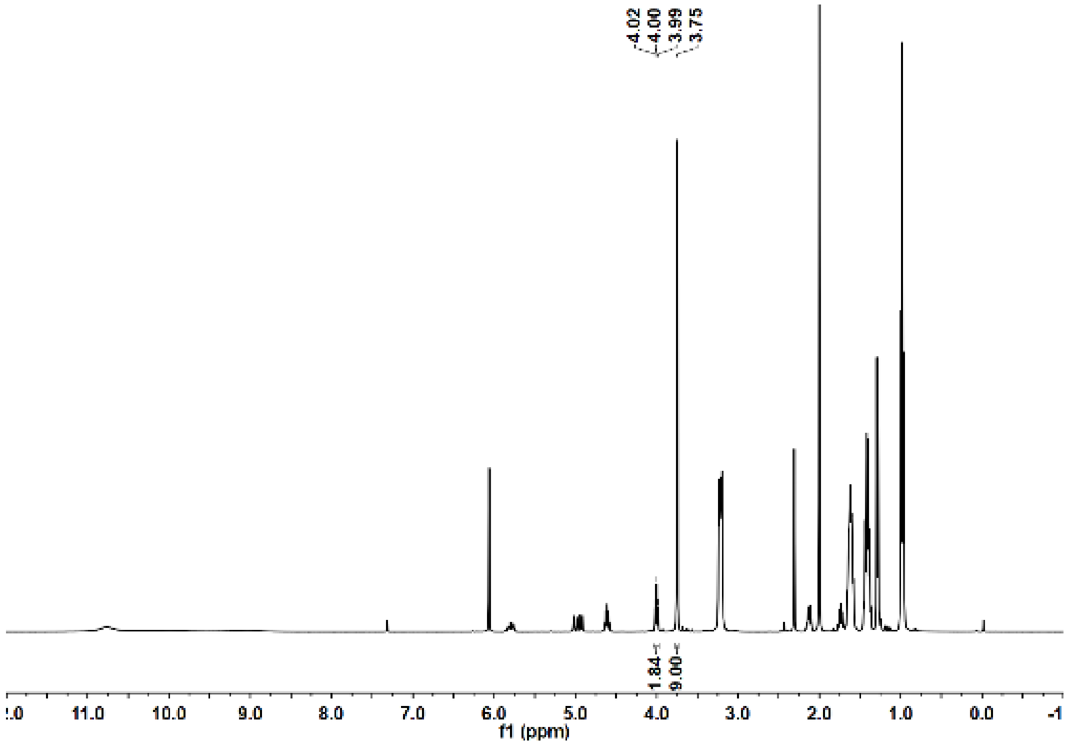

Supplementary Figure 2. NMR yield of sulfation for **11**.

Tetrabutylammonium 4-pentynyl alcohol sulfate (12)

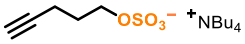

The reaction was carried out according to general procedure **B**. The yield of tetrabutylammonium 4-pentynyl alcohol sulfate **12** was purified by plate chromatography on silica gel eluting with DCM/MeOH (30:1, v/v) to afford the product as a light yellow oil (70.6 mg, 0.174 mmol, 87%).

R*f* = 0.40 (DCM/MeOH = 15:1, v/v).

NMR Spectroscopy:

^{1}H NMR (400 MHz, Chloroform-*d*, 298 K, δ): 4.04 (t, *J* = 6.3 Hz, 2H), 3.27 - 3.20 (m, 8H), 2.28 (td, *J* = 7.4, 2.7 Hz, 2H), 1.89 - 1.81 (m, 3H), 1.67 - 1.57 (m, 8H), 1.45 - 1.36 (m, 8H), 0.97 (t, *J* = 7.3 Hz, 12H) ppm.

^{13}C NMR (101 MHz, Chloroform-*d*, 298 K, δ): 84.3, 68.3, 65.8, 58.7, 28.9, 24.0, 19.8, 15.3, 13.7 ppm.

HRMS *m/z* (ESI-) calculated for $C_5H_7O_4S^-$, 163.0071, found, 163.0068 $[M\text{-}NBu_4]^-$.

Tetrabutylammonium 3-phenyl-2-propynol sulfate (13)

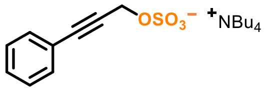

The reaction was carried out according to general procedure **B**. The yield of tetrabutylammonium 3-phenyl-2-propynol sulfate **13** was purified by plate chromatography on silica gel eluting with DCM/MeOH (30:1, v/v) to afford the product as a light yellow oil (57.2 mg, 0.126 mmol, 63%).

R*f* = 0.40 (DCM/MeOH = 15:1, v/v).

NMR Spectroscopy:

^{1}H NMR (400 MHz, Chloroform-*d*, 298 K, δ): 7.36 - 7.31 (m, 2H), 7.27 - 7.23 (m, 3H), 4.78 (s, 2H), 3.22 - 3.16 (m, 8H), 1.59 - 1.52 (m, 8H), 1.36 (h, *J* = 7.4 Hz, 8H), 0.92 (t, *J* = 7.4 Hz, 12H) ppm.

^{13}C NMR (101 MHz, Chloroform-*d*, 298 K, δ): 131.6, 128.4, 128.3, 122.7, 85.5, 85.1, 58.5, 55.5, 23.8, 19.6, 13.6 ppm.

HRMS *m/z* (ESI-) calculated for $C_9H_7O_4S^-$, 211.0071, found, 211.0066 $[M\text{-}NBu_4]^-$.

Tetrabutylammonium 4-ethenylphenylmethanol sulfate (14)

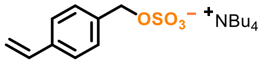

The reaction was carried out according to general procedure **B**. The yield of tetrabutylammonium 4-ethenylphenylmethanol sulfate **14** was purified by plate chromatography on silica gel eluting with DCM/MeOH (30:1, v/v) to afford the product as a light yellow oil (50.3 mg, 0.110 mmol, 55%).

R*f* = 0.40 (DCM/MeOH = 15:1, v/v).

NMR Spectroscopy:

^{1}H NMR (400 MHz, Chloroform-*d*, 298 K, δ): 7.38 - 7.30 (m, 4H), 6.67 (dd, *J* = 17.6, 10.9 Hz, 1H), 5.70 (d, *J* = 17.6 Hz, 1H), 5.20 (d, *J* = 10.8 Hz, 1H), 5.02 (s, 2H), 3.22 -

3.13 (m, 8H), 1.59 - 1.51 (m, 8H), 1.36 (h, *J* = 7.3 Hz, 8H), 0.94 (t, *J* = 7.3 Hz, 12H) ppm.

^{13}C NMR (101 MHz, Chloroform-*d*, 298 K, δ): 137.5, 136.8, 136.7, 128.4, 126.0, 113.7, 68.7, 58.6, 24.0, 19.8, 13.8 ppm.

HRMS *m/z* (ESI-) calculated for $C_9H_9O_4S^-$, 213.0227, found, 213.0223 $[M-NBu_4]^-$.

Tetrabutylammonium 4-pinacol-ester-phenylmethanol sulfate (15)

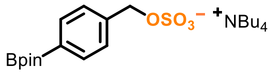

The reaction was carried out according to general procedure **B**. The yield of tetrabutylammonium 4-pinacol-ester-phenylmethanol sulfate **15** was purified by plate chromatography on silica gel eluting with DCM/MeOH (30:1, v/v) to afford the product as a light yellow oil (88.1 mg, 0.158 mmol, 79%).

R_f = 0.40 (DCM/MeOH = 15:1, v/v).

NMR Spectroscopy:

^{1}H NMR (400 MHz, Chloroform-*d*, 298 K, δ): 7.72 (d, *J* = 7.7 Hz, 2H), 7.38 (d, *J* = 7.7 Hz, 2H), 5.05 (s, 2H), 3.22 - 3.11 (m, 8H), 1.59 - 1.48 (m, 8H), 1.41 - 1.28 (m, 20H), 0.94 (t, *J* = 7.3 Hz, 12H) ppm.

^{13}C NMR (101 MHz, Chloroform-*d*, 298 K, δ): 141.0, 134.7, 127.1, 83.8, 68.8, 58.6, 25.0, 23.9, 19.7, 13.8 ppm.

HRMS *m/z* (ESI-) calculated for $C_{13}H_{18}BO_6S^-$, 313.0923, found, 313.0925 $[M-NBu_4]^-$.

Tetrabutylammonium 4-formyllphenylmethanol sulfate (16)

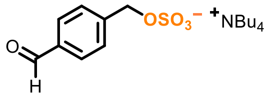

The reaction was carried out according to general procedure **B**. The yield of tetrabutylammonium 4-formyllphenylmethanol sulfate **16** was purified by plate chromatography on silica gel eluting with DCM/MeOH (30:1, v/v) to afford the product as a light yellow oil (64.1 mg, 0.140 mmol, 70%).

R_f = 0.40 (DCM/MeOH = 15:1, v/v).

NMR Spectroscopy:

^{1}H NMR (400 MHz, Chloroform-*d*, 298 K, δ): 9.96 (s, 1H), 7.79 (d, *J* = 8.2 Hz, 2H), 7.57 (d, *J* = 7.9 Hz, 2H), 5.12 (s, 2H), 3.25 - 3.18 (m, 8H), 1.63 - 1.54 (m, 8H), 1.42 - 1.33 (m, 8H), 0.94 (t, *J* = 7.3 Hz, 12H) ppm.

^{13}C NMR (101 MHz, Chloroform-*d*, 298 K, δ): 192.3, 145.2, 135.6, 129.7, 128.0, 68.1, 58.8, 24.0, 19.8, 13.7 ppm.

HRMS *m/z* (ESI-) calculated for $C_8H_7O_5S^-$, 215.0020, found, 215.0016 $[M-NBu_4]^-$.

Tetrabutylammonium 4-sulfoxylphenylmethanol sulfate (17)

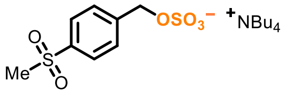

The reaction was carried out according to general procedure **B**. The yield of tetrabutylammonium 4-sulfoxylphenylmethanol sulfate **17** was purified by flash chromatography on silica gel eluting with DCM/MeOH (100:1 to 20:1, v/v) to afford the product as a light yellow oil (73.2 mg, 0.144 mmol, 72%).

$\mathbf{R}_f$ = 0.40 (DCM/MeOH = 15:1, v/v).

NMR Spectroscopy:

^{1}H NMR (400 MHz, Chloroform-*d*, 298 K, δ): 7.77 (d, *J* = 8.4 Hz, 2H), 7.55 (d, *J* = 8.1 Hz, 2H), 5.06 (s, 2H), 3.19 - 3.08 (m, 8H), 2.95 (s, 3H), 1.58 - 1.49 (m, 8H), 1.32 (h, *J* = 7.4 Hz, 8H), 0.88 (t, *J* = 7.4 Hz, 12H) ppm.

^{13}C NMR (101 MHz, Chloroform-*d*, 298 K, δ): 144.7, 139.0, 128.0, 127.0, 67.5, 58.5, 44.5, 23.8, 19.6, 13.6 ppm.

HRMS *m/z* (ESI-) calculated for $C_8H_9O_6S_2^-$, 264.9846, found, 264.9841 $[M-NBu_4]^-$.

Tetrabutylammonium thiophene-3-ethanol sulfate (18)

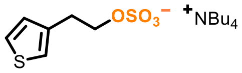

The reaction was carried out according to general procedure **A**. The yield of tetrabutylammonium thiophene-3-ethanol sulfate **18** was purified by plate chromatography on silica gel eluting with DCM/MeOH (30:1, v/v) to afford the product as a light yellow oil (71.8 mg, 0.160 mmol, 80%).

$\mathbf{R}_f$ = 0.40 (DCM/MeOH = 15:1, v/v).

NMR Spectroscopy:

^{1}H NMR (400 MHz, Chloroform-*d*, 298 K, δ): 7.15 (dd, *J* = 4.9, 3.0 Hz, 1H), 7.03 - 6.99 (m, 1H), 6.93 (dd, *J* = 4.9, 1.3 Hz, 1H), 4.17 (t, *J* = 7.3 Hz, 2H), 3.27 - 3.12 (m, 8H), 2.96 (t, *J* = 7.3 Hz, 2H), 1.62 - 1.49 (m, 8H), 1.36 (h, *J* = 7.3 Hz, 8H), 0.93 (t, *J* = 7.3 Hz, 12H) ppm.

^{13}C NMR (101 MHz, Chloroform-*d*, 298 K, δ): 139.0, 128.6, 124.9, 121.1, 66.7, 58.6, 30.5, 23.9, 19.7, 13.7 ppm.

HRMS *m/z* (ESI-) calculated for $C_6H_7O_4S_2^-$, 206.9791, found, 206.9788 $[M-NBu_4]^-$.

Tetrabutylammonium trichloroethanol sulfate (19)

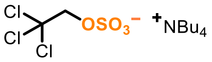

The reaction was carried out according to general procedure **B**. The yield of tetrabutylammonium trichloroethanol sulfate **19** was determined by ^{1}H NMR integration relative to the internal standard (34% yield; standard: δ 4.94 ppm, **19**: δ 4.53 (s) ppm). Then the reaction was purified by plate chromatography on silica gel eluting with DCM/MeOH (30:1, v/v) to afford the product as a colorless oil (22.6 mg, 0.048 mmol, 24%).

R*f* = 0.40 (DCM/MeOH = 15:1, v/v).

NMR Spectroscopy:

^{1}H NMR (400 MHz, Chloroform-*d*, 298 K, δ): 4.49 (s, 2H), 3.25 - 3.12 (m, 8H), 1.57 (p, *J* = 7.7 Hz, 8H), 1.37 (h, *J* = 7.3 Hz, 8H), 0.93 (t, *J* = 7.3 Hz, 12H) ppm.

^{13}C NMR (101 MHz, Chloroform-*d*, 298 K, δ): 96.2, 77.8, 58.6, 23.9, 19.7, 13.6 ppm.

HRMS *m/z* (ESI-) calculated for $C_2H_2Cl_3O_4S^-$, 226.8745, found, 226.8743 $[M\text{-}NBu_4]^-$.

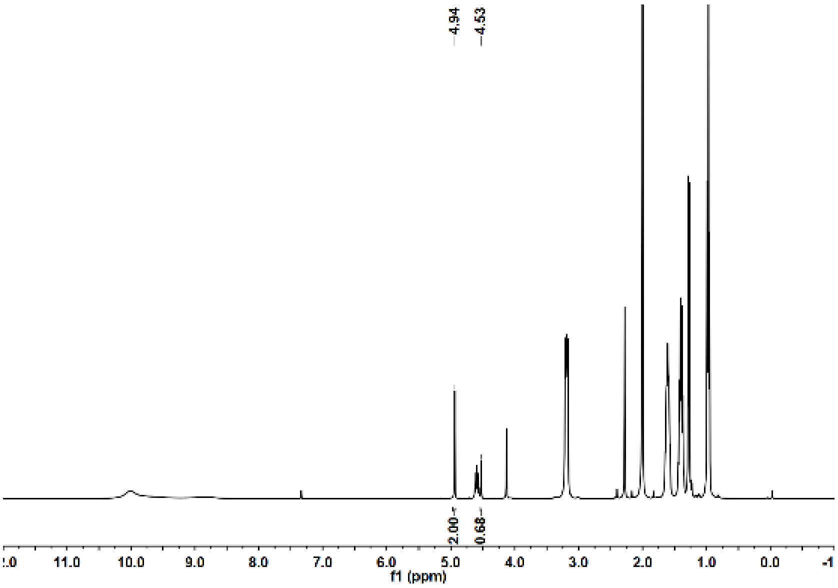

Supplementary Figure 3. NMR yield of sulfation for **19**.

Tetrabutylammonium 2-phenylethanethiol sulfate (20)

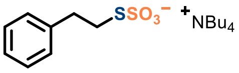

The reaction was carried out according to general procedure **A**. The yield of tetrabutylammonium 2-phenylethanethiol sulfate **20** was purified by plate chromatography on silica gel eluting with DCM/MeOH (30:1, v/v) to afford the product as a colorless oil (37.7 mg, 0.082 mmol, 41%).

R$_f$ = 0.40 (DCM/MeOH = 15:1, v/v).

NMR Spectroscopy:

^{1}H NMR (400 MHz, Chloroform-*d*, 298 K, δ): 7.26 - 7.21 (m, 4H), 7.16 (q, *J* = 4.3 Hz, 1H), 3.36 – 3.29 (m, 2H), 3.29 – 3.20 (m, 8H), 3.10 (t, *J* = 7.8 Hz, 2H), 1.62 (p, *J* = 7.6 Hz, 8H), 1.43 (h, *J* = 7.4 Hz, 8H), 0.99 (t, *J* = 7.3 Hz, 12H) ppm.

^{13}C NMR (101 MHz, Chloroform-*d*, 298 K, δ): 141.3, 128.8, 128.3, 126.1, 58.9, 36.4, 36.4, 24.1, 19.8, 13.8 ppm.

HRMS *m/z* (ESI-) calculated for $C_8H_9O_3S_2^-$, 216.9999, found, 217.0000 $[M\text{-}NBu_4]^-$.

Tetrabutylammonium 4-phenyl-2-butanol sulfate (21)

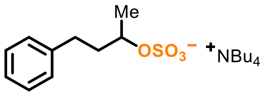

The reaction was carried out according to general procedure **B**. The yield of tetrabutylammonium 4-phenyl-2-butanol sulfate **21** was purified by plate chromatography on silica gel eluting with DCM/MeOH (30:1, v/v) to afford the product as a light yellow oil (79.3 mg, 0.168 mmol, 84%).

R$_f$ = 0.40 (DCM/MeOH = 15:1, v/v).

NMR Spectroscopy:

^{1}H NMR (400 MHz, Chloroform-*d*, 298 K, δ): 7.24 - 7.15 (m, 4H), 7.14 - 7.07 (m, 1H), 4.52 (h, *J* = 6.3 Hz, 1H), 3.29 - 3.19 (m, 8H), 2.80 (td, *J* = 16.3, 11.4, 5.3 Hz, 1H), 2.68 (td, *J* = 13.6, 12.6, 5.5 Hz, 1H), 1.95 (tt, *J* = 12.3, 6.2 Hz, 1H), 1.76 (tt, *J* = 11.6, 5.3 Hz, 1H), 1.61 (p, *J* = 7.8 Hz, 8H), 1.41 (h, *J* = 7.3 Hz, 8H), 1.33 (d, *J* = 6.2 Hz, 3H), 0.96 (t, *J* = 7.3 Hz, 12H) ppm.

^{13}C NMR (101 MHz, Chloroform-*d*, 298 K, δ): 143.0, 128.5, 128.2, 125.5, 73.9, 58.7, 32.0, 24.0, 21.2, 19.8, 13.7 ppm.

HRMS *m/z* (ESI-) calculated for $C_{10}H_{13}O_4S^-$, 229.0540, found, 229.0538 $[M\text{-}NBu_4]^-$.

Tetrabutylammonium methyl 3-hydroxyhexanoate sulfate (22)

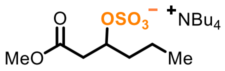

The reaction was carried out according to general procedure **B**. The yield of tetrabutylammonium methyl 3-hydroxyhexanoate sulfate **22** was purified by plate chromatography on silica gel eluting with DCM/MeOH (30:1, v/v) to afford the product as a light yellow oil (38.2 mg, 0.082 mmol, 41%).

R$_f$ = 0.40 (DCM/MeOH = 15:1, v/v).

NMR Spectroscopy:

^{1}**H NMR** (400 MHz, Chloroform-*d*, 298 K, δ): 4.71 - 4.60 (m, 1H), 3.60 (s, 3H), 3.27 - 3.21 (m, 8H), 3.09 (dd, *J* = 15.3, 4.4 Hz, 1H), 2.50 (dd, *J* = 15.3, 9.0 Hz, 1H), 1.65 - 1.47 (m, 12H), 1.43 - 1.37 (m, 8H), 0.96 (t, *J* = 7.3 Hz, 12H), 0.85 (t, *J* = 7.0 Hz, 3H) ppm.

^{13}**C NMR** (101 MHz, Chloroform-*d*, 298 K, δ): 172.1, 73.6, 58.6, 51.4, 40.2, 37.2, 24.0, 19.7, 18.5, 14.1, 13.7 ppm.

HRMS *m/z* (ESI-) calculated for $C_7H_{13}O_6S^-$, 225.0438, found, 225.0427 $[M\text{-}NBu_4]^-$.

Tetrabutylammonium 2-indanol sulfate (23)

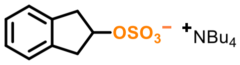

The reaction was carried out according to general procedure **B**. The yield of tetrabutylammonium 2-indanol sulfate **23** was purified by plate chromatography on silica gel eluting with DCM/MeOH (30:1, v/v) to afford the product as a light yellow oil (66.5 mg, 0.146 mmol, 73%).

$\mathbf{R}_f$ = 0.40 (DCM/MeOH = 15:1, v/v).

NMR Spectroscopy:

^{1}**H NMR** (400 MHz, Chloroform-*d*, 298 K, δ): 7.18 - 7.12 (m, 2H), 7.12 - 7.06 (m, 2H), 5.25 (p, *J* = 5.2 Hz, 1H), 3.26 - 3.22 (m, 4H), 3.21 - 3.14 (m, 8H), 1.56 (p, *J* = 7.6 Hz, 8H), 1.38 (h, *J* = 7.4 Hz, 8H), 0.96 (t, *J* = 7.3 Hz, 12H) ppm.

^{13}**C NMR** (101 MHz, Chloroform-*d*, 298 K, δ): 141.5, 126.3, 124.6, 78.0, 40.4, 24.0, 19.7, 13.8 ppm.

HRMS *m/z* (ESI-) calculated for $C_9H_9O_4S^-$, 213.0227, found, 213.0227 $[M\text{-}NBu_4]^-$.

Tetrabutylammonium DL-menthol sulfate (24)[7]

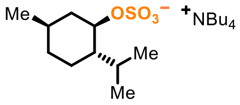

The reaction was carried out according to general procedure **B**. The yield of tetrabutylammonium DL-menthol sulfate **24** was purified by plate chromatography on silica gel eluting with DCM/MeOH (30:1, v/v) to afford the product as a light yellow oil (68.8 mg, 0.144 mmol, 72%).

$\mathbf{R}_f$ = 0.40 (DCM/MeOH = 15:1, v/v).

NMR Spectroscopy:

^{1}**H NMR** (400 MHz, Chloroform-*d*, 298 K, δ): 4.13 (td, *J* = 10.7, 4.4 Hz, 1H), 3.32 - 3.22 (m, 8H), 2.52 (d, *J* = 12.5 Hz, 1H), 2.35 (p, *J* = 7.1 Hz, 1H), 1.68 - 1.56 (m, 10H), 1.43

(h, J = 7.4 Hz, 9H), 1.28 - 1.18 (m, 1H), 0.98 (t, J = 7.3 Hz, 15H), 0.84 (q, J = 6.9, 6.3 Hz, 9H) ppm.

^{13}C NMR (101 MHz, Chloroform-*d*, 298 K, δ): 77.6, 58.8, 48.3, 42.3, 34.7, 31.7, 25.3, 24.1, 23.3, 22.3, 21.4, 19.8, 16.2, 13.8 ppm.

HRMS *m/z* (ESI-) calculated for $C_{10}H_{19}O_4S^-$, 235.1010, found, 235.1006 $[M\text{-}NBu_4]^-$.

Tetrabutylammonium 2-adamantanol sulfate (25)

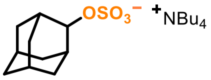

The reaction was carried out according to general procedure **B**. The yield of tetrabutylammonium 2-adamantanol sulfate **25** was determined by ^{1}H NMR integration relative to the internal standard (94% yield; standard: δ 4.94 ppm, **25**: δ 4.50 - 4.49 (m) ppm). Then the reaction was purified by plate chromatography on silica gel eluting with DCM/MeOH (30:1, v/v) to afford the product as a colorless oil (71.1 mg, 0.150 mmol, 75%).

R_f = 0.40 (DCM/MeOH = 15:1, v/v).

NMR Spectroscopy:

^{1}H NMR (400 MHz, Chloroform-*d*, 298 K, δ): 4.53 - 4.45 (m, 1H), 3.31 - 3.22 (m, 8H), 2.22 (s, 2H), 2.16 (s, 2H), 1.80 - 1.70 (m, 6H), 1.68 - 1.58 (m, 10H), 1.42 (h, J = 7.0 Hz, 10H), 0.98 (t, J = 7.3 Hz, 12H) ppm.

^{13}C NMR (101 MHz, Chloroform-*d*, 298 K, δ): 80.3, 58.8, 37.8, 36.8, 32.7, 31.7, 27.5, 27.3, 24.1, 19.8, 13.8 ppm.

HRMS *m/z* (ESI-) calculated for $C_{10}H_{15}O_4S^-$, 231.0697, found, 231.0692 $[M\text{-}NBu_4]^-$.

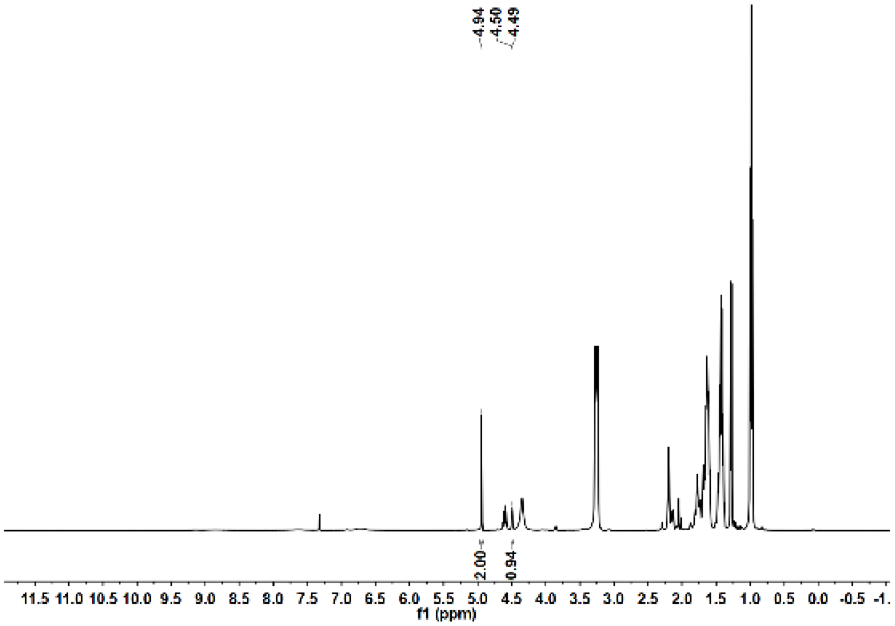

Supplementary Figure 4. NMR yield of sulfation for **25**.

Tetrabutylammonium cyclododecanol sulfate (26)

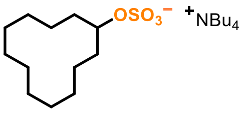

The reaction was carried out according to general procedure **B**. The yield of tetrabutylammonium cyclododecanol sulfate **26** was purified by plate chromatography on silica gel eluting with DCM/MeOH (30:1, v/v) to afford the product as a light yellow oil (84.8 mg, 0.168 mmol, 84%).

R*f* = 0.40 (DCM/MeOH = 15:1, v/v).

NMR Spectroscopy:

^{1}H NMR (400 MHz, Chloroform-*d*, 298 K, δ): 4.57 - 4.45 (m, 1H), 3.32 - 3.27 (m, 8H), 1.82 - 1.68 (m, 4H), 1.67 - 1.62 (m, 8H), 1.45 (p, *J* = 7.4 Hz, 13H), 1.30 (td, *J* = 21.5, 19.4, 12.2 Hz, 14H), 0.99 (d, *J* = 7.3 Hz, 12H) ppm.

^{13}C NMR (101 MHz, Chloroform-*d*, 298 K, δ): 76.5, 59.0, 29.7, 25.1, 24.7, 24.2, 23.4, 23.3, 20.6, 19.9, 13.8 ppm.

HRMS *m/z* (ESI-) calculated for $C_{12}H_{23}O_4S^-$, 263.1323, found, 263.1319 $[M\text{-}NBu_4]^-$.

Tetrabutylammonium *N*-tosyl-4-piperidinol sulfate (27)

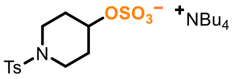

The reaction was carried out according to general procedure **B**. The yield of tetrabutylammonium *N*-tosyl-4-piperidinol sulfate **27** was purified by flash chromatography on silica gel eluting with DCM/MeOH (100:1 to 20:1, v/v) to afford the product as a white solid (92.2 mg, 0.160 mmol, 80%).

R*f* = 0.40 (DCM/MeOH = 15:1, v/v).

NMR Spectroscopy:

^{1}H NMR (400 MHz, Chloroform-*d*, 298 K, δ): 7.55 (d, *J* = 7.9 Hz, 2H), 7.25 (d, *J* = 8.1 Hz, 2H), 4.40 (p, *J* = 5.0 Hz, 1H), 3.22 - 3.14 (m, 8H), 3.12 - 3.04 (m, 2H), 3.04 - 2.93 (m, 2H), 2.38 (s, 3H), 1.92 (q, *J* = 5.4 Hz, 4H), 1.62 - 1.50 (m, 8H), 1.35 (h, *J* = 7.4 Hz, 8H), 0.92 (t, *J* = 7.3 Hz, 12H) ppm.

^{13}C NMR (101 MHz, Chloroform-*d*, 298 K, δ): 143.6, 133.2, 129.7, 127.6, 70.6, 58.7, 43.2, 31.0, 23.9, 21.5, 19.7, 13.7 ppm.

HRMS *m/z* (ESI-) calculated for $C_{12}H_{16}NO_6S_2^-$, 334.0425, found, 334.0418 $[M\text{-}NBu_4]^-$.

Tetrabutylammonium DL-pantolactone sulfate (28)

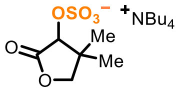

The reaction was carried out according to general procedure **B**. The yield of tetrabutylammonium DL-pantolactone sulfate **28** was determined by ^{1}H NMR integration relative to the internal standard (33% yield; standard: δ 4.94 ppm, **28**: δ 4.82 (s) ppm). Then the reaction was purified by plate chromatography on silica gel eluting with DCM/MeOH (30:1, v/v) to afford the product as a light yellow oil (19.1 mg, 0.042 mmol, 21%).

R*f* = 0.40 (DCM/MeOH = 15:1, v/v).

NMR Spectroscopy:

^{1}**H NMR** (400 MHz, Chloroform-*d*, 298 K, δ): 4.87 (s, 1H), 3.98 - 3.90 (m, 2H), 3.31 - 3.25 (m, 8H), 1.69 - 1.60 (m, 8H), 1.43 (h, *J* = 7.4 Hz, 8H), 1.34 (s, 3H), 1.12 (s, 3H), 0.99 (t, *J* = 7.3 Hz, 12H) ppm.

^{13}**C NMR** (101 MHz, Chloroform-*d*, 298 K, δ): 174.9, 79.1, 76.5, 58.8, 40.7, 24.1, 23.2, 19.8, 19.7, 13.8 ppm.

HRMS *m/z* (ESI-) calculated for $C_6H_9O_6S^-$, 209.0125, found, 209.0117 [M-NBu_4]$^-$.

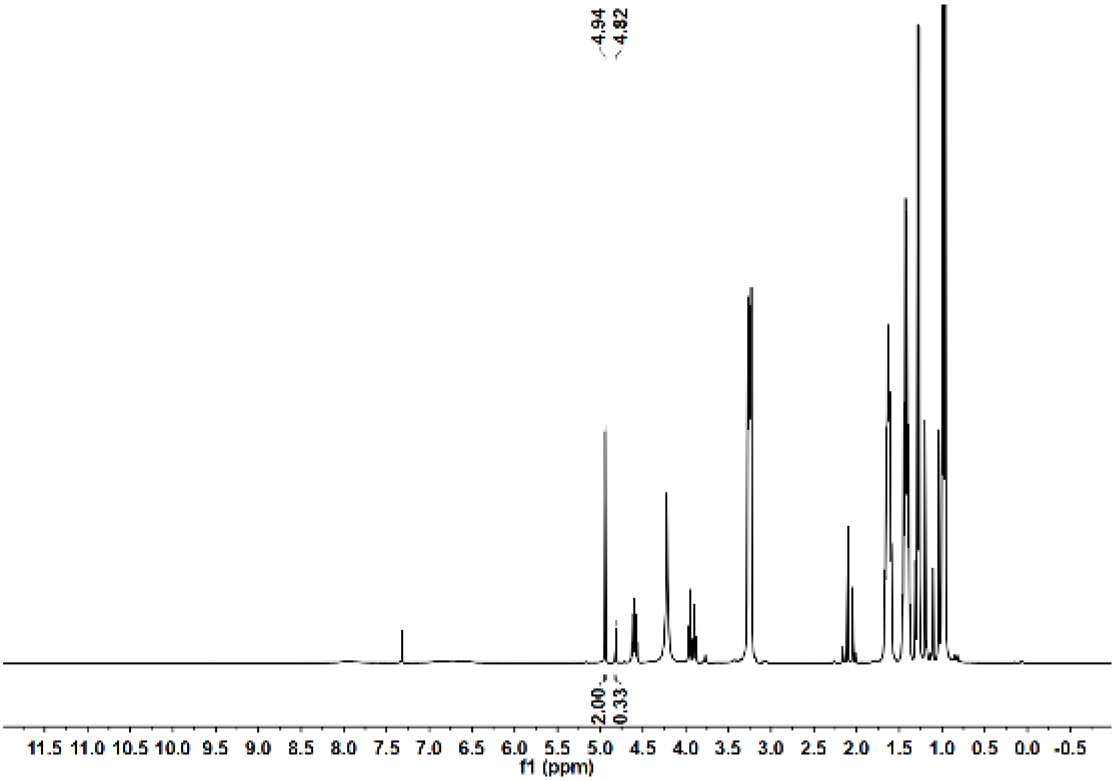

Supplementary Figure 5. NMR yield of sulfation for **28**.

Tetrabutylammonium 4-chloro-6,7-dihydro-5H-[1]pyrindin-7-ol sulfate (29)

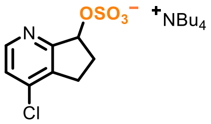

The reaction was carried out according to general procedure **B**. The yield of tetrabutylammonium 4-chloro-6,7-dihydro-5H-[1]pyrindin-7-ol sulfate **29** was determined by ^{1}H NMR integration relative to the internal standard (40% yield; standard: δ 4.94 ppm, **29**: δ 5.68 (t, *J* = 7.2 Hz) ppm). Then the reaction was purified by plate chromatography on silica gel eluting with DCM/MeOH (30:1, v/v) to afford the product as a light yellow oil (28.5 mg, 0.058 mmol, 29%).

R_f = 0.40 (DCM/MeOH = 15:1, v/v).

NMR Spectroscopy:

^{1}H NMR (400 MHz, Chloroform-*d*, 298 K, δ): 8.30 (d, *J* = 5.3 Hz, 1H), 7.15 (d, *J* = 5.3 Hz, 1H), 5.78 (dd, *J* = 7.0, 4.0 Hz, 1H), 3.29 - 3.23 (m, 8H), 3.15 - 3.03 (m, 1H), 2.90 - 2.84 (m, 1H), 2.66 - 2.46 (m, 2H), 1.63 (q, *J* = 7.9 Hz, 8H), 1.39 (q, *J* = 7.4 Hz, 8H), 0.95 (t, *J* = 7.3 Hz, 12H) ppm.

^{13}C NMR (101 MHz, Chloroform-*d*, 298 K, δ): 163.6, 149.0, 141.7, 137.2, 123.3, 80.3, 58.7, 30.5, 27.3, 24.0, 19.8, 13.8 ppm.

HRMS *m/z* (ESI-) calculated for $C_8H_7ClNO_4S^-$, 247.9790, found, 247.9784 $[M-NBu_4]^-$.

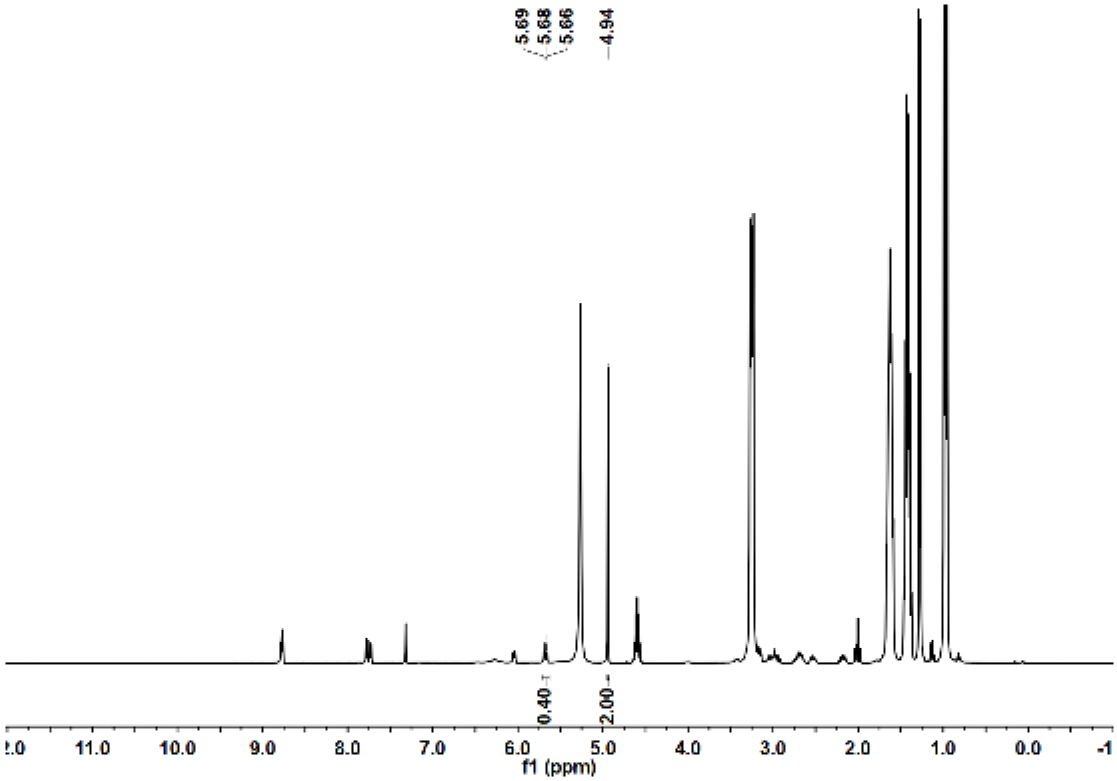

Supplementary Figure 6. NMR yield of sulfation for **29**.

Tetrabutylammonium (1-hydroxycyclohexyl)phenyl-methanon sulfate (30)

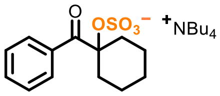

The reaction was carried out according to general procedure **C**. The yield of tetrabutylammonium (1-hydroxycyclohexyl)phenyl-methanon sulfate **30** was purified by plate chromatography on silica gel eluting with DCM/MeOH (30:1, v/v) to afford the product as a light yellow oil (89.4 mg, 0.170 mmol, 85%).

$\mathbf{R}_f$ = 0.40 (DCM/MeOH = 15:1, v/v).

NMR Spectroscopy:

^{1}**H NMR** (400 MHz, Chloroform-*d*, 298 K, δ): 8.35 (d, *J* = 7.6 Hz, 2H), 7.44 - 7.32 (m, 3H), 3.13 - 3.05 (m, 8H), 2.32 (d, *J* = 13.7 Hz, 2H), 1.92 (t, *J* = 12.1 Hz, 2H), 1.74 (t, *J* = 12.0 Hz, 2H), 1.52 (p, *J* = 8.5, 7.8 Hz, 10H), 1.33 (h, *J* = 7.3 Hz, 8H), 1.28 - 1.19 (m, 2H), 0.94 (t, *J* = 7.3 Hz, 12H) ppm.

^{13}**C NMR** (101 MHz, Chloroform-*d*, 298 K, δ): 201.9, 136.3, 131.4, 130.3, 127.9, 85.9, 58.5, 33.7, 25.7, 24.0, 21.8, 19.7, 13.8 ppm.

HRMS *m/z* (ESI-) calculated for $C_{13}H_{15}O_5S^-$, 283.0646, found, 283.0630 $[M\text{-}NBu_4]^-$.

Tetrabutylammonium HOBt sulfate (31)

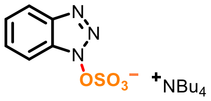

The reaction was carried out according to general procedure **C**. The yield of tetrabutylammonium HOBt sulfate **31** was purified by plate chromatography on silica gel eluting with DCM/MeOH (30:1, v/v) to afford the product as a light yellow oil (70.0 mg, 0.153 mmol, 77%).

$\mathbf{R}_f$ = 0.40 (DCM/MeOH = 15:1, v/v).

NMR Spectroscopy:

^{1}**H NMR** (400 MHz, Chloroform-*d*, 298 K, δ): 7.86 (d, *J* = 8.4 Hz, 1H), 7.79 (d, *J* = 7.9 Hz, 1H), 7.40 (t, *J* = 7.6 Hz, 1H), 7.28 (t, *J* = 7.6 Hz, 1H), 3.11 - 3.02 (m, 8H), 1.55 - 1.44 (m, 8H), 1.28 (p, *J* = 7.4 Hz, 8H), 0.88 (t, *J* = 7.2 Hz, 12H) ppm.

^{13}**C NMR** (101 MHz, Chloroform-*d*, 298 K, δ): 143.3, 128.9, 127.5, 124.2, 119.2, 111.5, 58.4, 23.8, 19.5, 13.6 ppm.

HRMS *m/z* (ESI-) calculated for $C_6H_4N_3O_4S^-$, 213.9928, found, 213.9924 $[M\text{-}NBu_4]^-$.

Tetrabutylammonium HOAt sulfate (32)

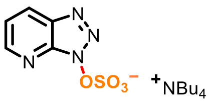

The reaction was carried out according to general procedure **C**. The yield of tetrabutylammonium HOAt sulfate **32** was purified by plate chromatography on silica gel eluting with DCM/MeOH (30:1, v/v) to afford the product as a light yellow oil (68.6 mg, 0.150 mmol, 75%).

R*f* = 0.40 (DCM/MeOH = 15:1, v/v).

NMR Spectroscopy:

^{1}H NMR (400 MHz, Chloroform-*d*, 298 K, δ): 8.67 (d, *J* = 4.4 Hz, 1H), 8.26 (d, *J* = 8.3 Hz, 1H), 7.32 (dd, *J* = 8.4, 4.4 Hz, 1H), 3.27 - 3.22 (m, 8H), 1.65 - 1.56 (m, 8H), 1.34 (h, *J* = 7.4 Hz, 8H), 0.89 (t, *J* = 7.3 Hz, 12H) ppm.

^{13}C NMR (101 MHz, Chloroform-*d*, 298 K, δ): 151.0, 140.7, 134.8, 128.5, 120.2, 58.5, 23.9, 19.6, 13.7 ppm.

HRMS *m/z* (ESI-) calculated for $C_5H_3N_4O_4S^-$, 214.9880, found, 214.9875 $[M-NBu_4]^-$.

Tetrabutylammonium 4-phenylphenol sulfate (33)

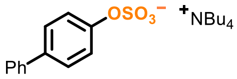

The reaction was carried out according to general procedure **C**. The yield of tetrabutylammonium 4-phenylphenol sulfate **33** was purified by plate chromatography on silica gel eluting with DCM/MeOH (30:1, v/v) to afford the product as a light yellow solid (83.5 mg, 0.170 mmol, 85%).

R*f* = 0.40 (DCM/MeOH = 15:1, v/v).

NMR Spectroscopy:

^{1}H NMR (400 MHz, Chloroform-*d*, 298 K, δ): 7.53 - 7.43 (m, 4H), 7.42 - 7.34 (m, 4H), 7.30 - 7.26 (m, 1H), 3.18 - 3.09 (m, 8H), 1.57 - 1.43 (m, 8H), 1.32 (h, *J* = 7.4 Hz, 8H), 0.90 (t, *J* = 7.3 Hz, 12H) ppm.

^{13}C NMR (101 MHz, Chloroform-*d*, 298 K, δ): 153.2, 140.8, 136.3, 128.7, 127.5, 126.9, 126.8, 121.3, 58.4, 23.8, 19.6, 13.6 ppm.

HRMS *m/z* (ESI-) calculated for $C_{12}H_9O_4S^-$, 249.0227, found, 249.0221 $[M-NBu_4]^-$.

Tetrabutylammonium 1-naphthol sulfate (34)

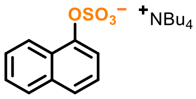

The reaction was carried out according to general procedure **C**. The yield of tetrabutylammonium 1-naphthol sulfate **34** was purified by plate chromatography on silica gel eluting with DCM/MeOH (30:1, v/v) to afford the product as a light yellow oil (81.8 mg, 0.176 mmol, 88%).

R*f* = 0.40 (DCM/MeOH = 15:1, v/v).

NMR Spectroscopy:

^{1}H NMR (400 MHz, Chloroform-*d*, 298 K, δ): 8.42 - 8.34 (m, 1H), 7.79 - 7.67 (m, 2H), 7.53 (d, *J* = 8.2 Hz, 1H), 7.44 - 7.33 (m, 3H), 3.05 - 2.99 (m, 8H), 1.44 (p, *J* = 8.1 Hz, 8H), 1.27 (h, *J* = 7.4 Hz, 8H), 0.90 (t, *J* = 7.2 Hz, 12H) ppm.

^{13}C NMR (101 MHz, Chloroform-*d*, 298 K, δ): 149.6, 134.6, 128.0, 127.3, 126.0, 125.4, 123.6, 123.3, 116.4, 58.4, 23.9, 19.7, 13.8 ppm.

HRMS *m/z* (ESI-) calculated for $C_{10}H_7O_4S^-$, 223.0071, found, 223.0067 $[M\text{-}NBu_4]^-$.

Tetrabutylammonium 4-ethylphenol sulfate (35)

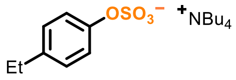

The reaction was carried out according to general procedure **C**. The yield of tetrabutylammonium 4-ethylphenol sulfate **35** was purified by flash chromatography on silica gel eluting with DCM/MeOH (100:1 to 20:1, v/v) to afford the product as a light yellow oil (72.6 mg, 0.164 mmol, 82%).

R*f* = 0.40 (DCM/MeOH = 15:1, v/v).

NMR Spectroscopy:

^{1}H NMR (400 MHz, Chloroform-*d*, 298 K, δ): 7.23 (d, *J* = 8.2 Hz, 2H), 7.05 (d, *J* = 8.1 Hz, 2H), 3.19 - 3.08 (m, 8H), 2.55 (q, *J* = 7.6 Hz, 2H), 1.53 (p, *J* = 7.6 Hz, 8H), 1.34 (h, *J* = 7.3 Hz, 8H), 1.16 (t, *J* = 7.6 Hz, 3H), 0.93 (t, *J* = 7.3 Hz, 12H) ppm.

^{13}C NMR (101 MHz, Chloroform-*d*, 298 K, δ): 151.5, 139.4, 128.2, 121.1, 58.5, 28.3, 23.9, 19.7, 15.9, 13.8 ppm.

HRMS *m/z* (ESI-) calculated for $C_8H_9O_4S^-$, 201.0227, found, 201.0224 $[M\text{-}NBu_4]^-$.

Tetrabutylammonium 4-(tert-butyl)phenol sulfate (36)

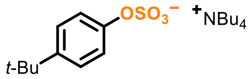

The reaction was carried out according to general procedure **C**. The yield of tetrabutylammonium 4-(tert-butyl)phenol sulfate **36** was purified by plate chromatography on silica gel eluting with DCM/MeOH (30:1, v/v) to afford the product as a light yellow oil (81.9 mg, 0.174 mmol, 87%).

R_f = 0.40 (DCM/MeOH = 15:1, v/v).

NMR Spectroscopy:

^{1}H NMR (400 MHz, Chloroform-*d*, 298 K, δ): 7.25 (d, *J* = 7.4 Hz, 4H), 3.21 - 3.12 (m, 8H), 1.55 (p, *J* = 7.7 Hz, 8H), 1.35 (h, *J* = 7.3 Hz, 8H), 1.25 (s, 9H), 0.93 (t, *J* = 7.3 Hz, 12H) ppm.

^{13}C NMR (101 MHz, Chloroform-*d*, 298 K, δ): 151.2, 146.2, 125.8, 120.6, 58.6, 34.3, 31.6, 23.9, 19.7, 13.8 ppm.

HRMS *m/z* (ESI-) calculated for $C_{10}H_{13}O_4S^-$, 229.0540, found, 229.0542 $[M-NBu_4]^-$.

Tetrabutylammonium 4-(trifluoromethyl)phenol sulfate (37)

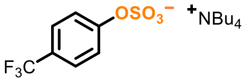

The reaction was carried out according to general procedure **C**. The yield of tetrabutylammonium 4-(trifluoromethyl)phenol sulfate **37** was purified by plate chromatography on silica gel eluting with DCM/MeOH (30:1, v/v) to afford the product as a light yellow oil (87.0 mg, 0.182 mmol, 90%).

R_f = 0.40 (DCM/MeOH = 15:1, v/v).

NMR Spectroscopy:

^{1}H NMR (400 MHz, Chloroform-*d*, 298 K, δ): 7.52 (d, *J* = 8.7 Hz, 2H), 7.47 (d, *J* = 8.7 Hz, 2H), 3.25 - 3.13 (m, 8H), 1.65 - 1.54 (m, 8H), 1.39 (h, *J* = 7.4 Hz, 8H), 0.97 (t, *J* = 7.3 Hz, 12H) ppm.

^{13}C NMR (101 MHz, Chloroform-*d*, 298 K, δ): 156.6, 126.2 (q, *J* = 3.8 Hz), 125.8, 125.4, 125.1, 123.1, 120.7, 58.7, 24.0, 19.7, 13.7 ppm.

^{19}F NMR (376 MHz, Chloroform-*d*, 298 K, δ): -61.74 ppm.

HRMS *m/z* (ESI-) calculated for $C_7H_4F_3O_4S^-$, 240.9788, found, 240.9789 $[M-NBu_4]^-$.

Tetrabutylammonium 4-cyanophenol sulfate (38)

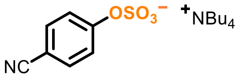

The reaction was carried out according to general procedure **C**. The yield of tetrabutylammonium 4-cyanophenol sulfate **38** was purified by plate chromatography on silica gel eluting with DCM/MeOH (30:1, v/v) to afford the product as a light yellow oil (71.4 mg, 0.162 mmol, 81%).

R_f = 0.40 (DCM/MeOH = 15:1, v/v).

NMR Spectroscopy:

^{1}**H NMR** (400 MHz, Chloroform-*d*, 298 K, δ): 7.56 (d, *J* = 8.5 Hz, 2H), 7.45 (d, *J* = 8.6 Hz, 2H), 3.24 - 3.16 (m, 8H), 1.60 (p, *J* = 8.4 Hz, 8H), 1.40 (h, *J* = 7.4 Hz, 8H), 0.97 (t, *J* = 7.3 Hz, 12H) ppm.

^{13}**C NMR** (101 MHz, Chloroform-*d*, 298 K, δ): 157.5, 133.5, 121.0, 119.4, 106.3, 58.8, 24.0, 19.8, 13.7 ppm.

HRMS *m/z* (ESI-) calculated for $C_7H_4NO_4S^-$, 197.9867, found, 197.9867 $[M\text{-}NBu_4]^-$.

Tetrabutylammonium 4-acetophenol sulfate (39)

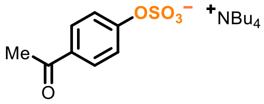

The reaction was carried out according to general procedure **C**. The yield of tetrabutylammonium 4-acetophenol sulfate **39** was purified by plate chromatography on silica gel eluting with DCM/MeOH (30:1, v/v) to afford the product as a light yellow oil (75.8 mg, 0.166 mmol, 83%).

$\mathbf{R_f}$ = 0.40 (DCM/MeOH = 15:1, v/v).

NMR Spectroscopy:

^{1}**H NMR** (400 MHz, Chloroform-*d*, 298 K, δ): 7.90 (d, *J* = 8.3 Hz, 2H), 7.45 (d, *J* = 8.3 Hz, 2H), 3.26 - 3.22 (m, 8H), 2.55 (s, 3H), 1.65 - 1.59 (m, 8H), 1.45 - 1.39 (m, 8H), 0.99 (t, *J* = 7.3 Hz, 12H) ppm.

^{13}**C NMR** (101 MHz, Chloroform-*d*, 298 K, δ): 197.5, 158.0, 132.3, 130.0, 120.1, 58.9, 26.7, 24.1, 19.8, 13.8 ppm.

HRMS *m/z* (ESI-) calculated for $C_8H_7O_5S^-$, 215.0020, found, 215.0020 $[M\text{-}NBu_4]^-$.

Tetrabutylammonium 2-iodophenol sulfate (40)[4]

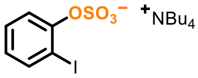

The reaction was carried out according to general procedure **C**. The yield of tetrabutylammonium 2-iodophenol sulfate **40** was purified by plate chromatography on silica gel eluting with DCM/MeOH (30:1, v/v) to afford the product as a light yellow oil (80.9 mg, 0.149 mmol, 75%).

$\mathbf{R_f}$ = 0.30 (DCM/MeOH = 15:1, v/v).

NMR Spectroscopy:

^{1}**H NMR** (400 MHz, Chloroform-*d*, 298 K, δ): 7.80 - 7.69 (m, 2H), 7.24 (d, *J* = 7.1 Hz, 1H), 6.76 (t, *J* = 7.6 Hz, 1H), 3.23 - 3.19 (m, 8H), 1.63 - 1.57 (m, 8H), 1.43 - 1.37 (m, 8H), 0.96 (t, *J* = 7.3 Hz, 12H) ppm.

^{13}**C NMR** (101 MHz, Chloroform-*d*, 298 K, δ): 153.6, 139.0, 129.2, 125.0, 121.0, 89.7, 58.8, 24.1, 19.8, 13.8 ppm.

HRMS *m/z* (ESI-) calculated for $C_6H_4IO_4S^-$, 298.8880, found, 298.8881 $[M-NBu_4]^-$.

Tetrabutylammonium 2-phenylphenol sulfate (41)

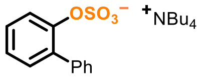

The reaction was carried out according to general procedure **C**. The yield of tetrabutylammonium 2-phenylphenol sulfate **41** was purified by plate chromatography on silica gel eluting with DCM/MeOH (30:1, v/v) to afford the product as a light yellow oil (66.9 mg, 0.136 mmol, 68%).

$\mathbf{R}_{\boldsymbol{f}}$ = 0.40 (DCM/MeOH = 15:1, v/v).

NMR Spectroscopy:

^{1}**H NMR** (400 MHz, Chloroform-*d*, 298 K, δ): 7.91 (d, *J* = 8.7 Hz, 1H), 7.69 (d, *J* = 7.1 Hz, 2H), 7.38 - 7.28 (m, 3H), 7.26 - 7.21 (m, 2H), 7.09 (t, *J* = 7.4 Hz, 1H), 3.07 - 3.00 (m, 8H), 1.49 - 1.41 (m, 8H), 1.27 (h, *J* = 7.3 Hz, 8H), 0.90 (t, *J* = 7.3 Hz, 12H) ppm.

^{13}**C NMR** (101 MHz, Chloroform-*d*, 298 K, δ): 150.7, 138.8, 133.0, 130.6, 130.0, 128.2, 128.0, 126.7, 123.6, 121.2, 58.5, 23.9, 19.7, 13.7 ppm.

HRMS *m/z* (ESI-) calculated for $C_{12}H_9O_4S^-$, 249.0227, found, 249.0228 $[M-NBu_4]^-$.

Tetrabutylammonium 3-methoxyphenol sulfate (42)[4]

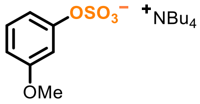

The reaction was carried out according to general procedure **C**. The yield of tetrabutylammonium 3-methoxyphenol sulfate **42** was purified by plate chromatography on silica gel eluting with DCM/MeOH (30:1, v/v) to afford the product as a light yellow oil (71.3 mg, 0.160 mmol, 80%).

$\mathbf{R}_{\boldsymbol{f}}$ = 0.40 (DCM/MeOH = 15:1, v/v).

NMR Spectroscopy:

^{1}**H NMR** (400 MHz, Chloroform-*d*, 298 K, δ): 10.43 (s, 1H), 7.50 (d, *J* = 8.4 Hz, 1H), 6.37 - 6.21 (m, 2H), 3.67 (s, 3H), 3.08 - 2.98 (m, 8H), 1.44 (p, *J* = 7.6 Hz, 8H), 1.26 (h, *J* = 7.4 Hz, 8H), 0.86 (t, *J* = 7.3 Hz, 12H) ppm.

^{13}**C NMR** (101 MHz, Chloroform-*d*, 298 K, δ): 161.9, 155.2, 128.7, 123.2, 105.6, 101.1, 58.3, 55.2, 23.7, 19.5, 13.5 ppm.

HRMS *m/z* (ESI-) calculated for $C_7H_7O_5S^-$, 203.0020, found, 203.0028 $[M-NBu_4]^-$.

Tetrabutylammonium methyl 3-hydroxybenzoate sulfate (43)

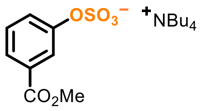

The reaction was carried out according to general procedure **C**. The yield of tetrabutylammonium methyl 3-hydroxybenzoate sulfate **43** was purified by plate chromatography on silica gel eluting with DCM/MeOH (30:1, v/v) to afford the product as a light yellow oil (78.6 mg, 0.166 mmol, 83%).

R*f* = 0.40 (DCM/MeOH = 15:1, v/v).

NMR Spectroscopy:

^{1}H NMR (400 MHz, Chloroform-*d*, 298 K, δ): 7.98 (s, 1H), 7.75 (d, *J* = 7.7 Hz, 1H), 7.62 (d, *J* = 8.5 Hz, 1H), 7.34 (t, *J* = 7.9 Hz, 1H), 3.86 (s, 3H), 3.27 - 3.16 (m, 8H), 1.60 (p, *J* = 7.6 Hz, 8H), 1.39 (h, *J* = 7.4 Hz, 8H), 0.97 (t, *J* = 7.3 Hz, 12H) ppm.

^{13}C NMR (101 MHz, Chloroform-*d*, 298 K, δ): 167.0, 153.7, 131.1, 129.1, 126.1, 124.9, 122.3, 58.8, 52.2, 24.0, 19.8, 13.8 ppm.

HRMS *m/z* (ESI-) calculated for $C_8H_7O_6S^-$, 230.9969, found, 230.9970 [M-NBu$_4$]$^-$.

Tetrabutylammonium 4-chloro-3-ethylphenol sulfate (44)

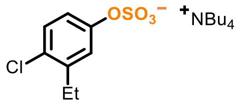

The reaction was carried out according to general procedure **C**. The yield of tetrabutylammonium 4-chloro-3-ethylphenol sulfate **44** was purified by plate chromatography on silica gel eluting with DCM/MeOH (30:1, v/v) to afford the product as a light yellow oil (78.3 mg, 0.164 mmol, 82%).

R*f* = 0.40 (DCM/MeOH = 15:1, v/v).

NMR Spectroscopy:

^{1}H NMR (400 MHz, Chloroform-*d*, 298 K, δ): 7.23 (s, 1H), 7.18 (d, *J* = 2.4 Hz, 2H), 3.23 - 3.17 (m, 8H), 2.68 (q, *J* = 7.5 Hz, 2H), 1.64 - 1.56 (m, 8H), 1.39 (h, *J* = 7.4 Hz, 8H), 1.19 (t, *J* = 7.5 Hz, 3H), 0.97 (t, *J* = 7.3 Hz, 12H) ppm.

^{13}C NMR (101 MHz, Chloroform-*d*, 298 K, δ): 152.4, 142.1, 129.5, 128.4, 122.3, 119.9, 58.8, 27.0, 24.0, 19.8, 14.0, 13.8 ppm.

HRMS *m/z* (ESI-) calculated for $C_8H_8ClO_4S^-$, 234.9837, found, 234.9839 [M-NBu$_4$]$^-$.

Tetrabutylammonium 4-methyl-benzenethio sulfate (45)

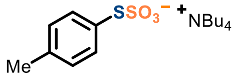

The reaction was carried out according to general procedure **C**. The yield of tetrabutylammonium 4-methyl-benzenethio sulfate **45** was purified by plate chromatography on silica gel eluting with DCM/MeOH (30:1, v/v) to afford the product as a light yellow oil (73.8 mg, 0.166 mmol, 83%).

$\mathbf{R}_f$ = 0.40 (DCM/MeOH = 15:1, v/v).

NMR Spectroscopy:

^{1}H NMR (400 MHz, Chloroform-*d*, 298 K, δ): 7.56 (d, *J* = 7.6 Hz, 2H), 7.06 (d, *J* = 7.7 Hz, 2H), 3.18 - 3.09 (m, 8H), 2.28 (s, 3H), 1.53 (p, *J* = 7.7 Hz, 8H), 1.35 (h, *J* = 7.5 Hz, 8H), 0.93 (t, *J* = 7.3 Hz, 12H) ppm.

^{13}C NMR (101 MHz, Chloroform-*d*, 298 K, δ): 137.5, 133.7, 131.4, 129.3, 58.5, 23.9, 21.2, 19.7, 13.7 ppm.

HRMS *m/z* (ESI-) calculated for $C_7H_7O_3S_2^-$, 202.9842, found, 202.9843 $[M\text{-}NBu_4]^-$.

Tetrabutylammonium saccharide derivative sulfate (46)

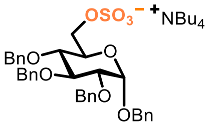

The reaction was carried out according to general procedure **A** with modified conditons: 2.0 equiv DMS and 2.0 equiv Bu_4NHSO_4 were used. The yield of tetrabutylammonium saccharide derivative sulfate **46** was purified by plate chromatography on silica gel eluting with DCM/MeOH (30:1, v/v) to afford the product as a white oil (26.0 mg, 0.030 mmol, 81%).

$\mathbf{R}_f$ = 0.40 (DCM/MeOH = 15:1, v/v).

NMR Spectroscopy:

^{1}H NMR (600 MHz, Chloroform-*d*, 298 K, δ): 7.42 - 7.26 (m, 19H), 7.24 - 7.22 (m, 1H), 4.99 (d, *J* = 11.2 Hz, 1H), 4.89 (dd, *J* = 16.2, 10.6 Hz, 2H), 4.82 - 4.78 (m, 2H), 4.71 (d, *J* = 12.3 Hz, 1H), 4.66 (d, *J* = 12.0 Hz, 1H), 4.56 (d, *J* = 1.5 Hz, 1H), 4.54 (d, *J* = 1.9 Hz, 1H), 4.42 (dd, *J* = 10.8, 3.5 Hz, 1H), 4.23 (dd, *J* = 10.9, 2.1 Hz, 1H), 4.04 (t, *J* = 9.3 Hz, 1H), 3.90 - 3.86 (m, 1H), 3.74 (t, *J* = 9.5 Hz, 1H), 3.53 (dd, *J* = 9.7, 3.7 Hz, 1H), 3.26 - 3.22 (m, 8H), 1.63 - 1.59 (m, 8H), 1.42 (p, *J* = 7.4 Hz, 8H), 0.98 (t, *J* = 7.3 Hz, 12H) ppm.

^{13}C NMR (150 MHz, Chloroform-*d*, 298 K, δ): 139.4, 138.7, 138.4, 137.4, 128.8, 128.5, 128.4, 128.3, 128.3, 128.0, 127.8, 127.7, 127.6, 127.4, 95.4, 82.1, 79.8, 77.7, 75.5, 75.1, 73.1, 70.0, 68.8, 65.4, 58.8, 24.0, 19.8, 13.8 ppm.

HRMS *m/z* (ESI+) calculated for $C_{34}H_{36}O_9SNa^+$, 643.1972, found, 643.1979 [M-NBu_4+Na+H]$^+$.

Tetrabutylammonium saccharide derivative sulfate (47)

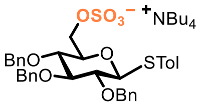

The reaction was carried out according to general procedure **A** with modified conditons: 2.0 equiv DMS and 2.0 equiv Bu_4NHSO_4 were used. The yield of tetrabutylammonium saccharide derivative sulfate **47** was purified by plate chromatography on silica gel eluting with DCM/MeOH (30:1, v/v) to afford the product as a white oil (26.0 mg, 0.030 mmol, 81%).

$\mathbf{R_f}$ = 0.40 (DCM/MeOH = 15:1, v/v).

NMR Spectroscopy:

^{1}H NMR (500 MHz, Chloroform-*d*, 298 K, δ): 7.45 (d, *J* = 8.1 Hz, 2H), 7.40 - 7.27 (m, 13H), 7.26 - 7.21 (m, 2H), 7.10 (d, *J* = 7.9 Hz, 2H), 4.94 (d, *J* = 10.1 Hz, 1H), 4.91 (d, *J* = 11.1 Hz, 1H), 4.85 (d, *J* = 11.1 Hz, 1H), 4.80 (d, *J* = 10.3 Hz, 1H), 4.76 (d, *J* = 10.0 Hz, 1H), 4.69 (d, *J* = 10.3 Hz, 1H), 4.60 (d, *J* = 9.8 Hz, 1H), 4.40 (dd, *J* = 10.8, 3.9 Hz, 1H), 4.35 (dd, *J* = 10.8, 2.1 Hz, 1H), 3.73 (t, *J* = 9.3 Hz, 1H), 3.67 (t, *J* = 8.9 Hz, 1H), 3.55 - 3.51 (m, 1H), 3.43 - 3.37 (m, 1H), 3.28 - 3.24 (m, 8H), 2.31 (s, 3H), 1.65 - 1.60 (m, 8H), 1.44 - 1.39 (m, 8H), 0.98 (t, *J* = 7.3 Hz, 12H) ppm.

^{13}C NMR (150 MHz, Chloroform-*d*, 298 K, δ): 138.8, 138.3, 138.2, 137.5, 131.7, 130.5, 129.9, 128.8, 128.5, 128.5, 128.4, 128.3, 127.9, 127.8, 127.7, 127.6, 88.0, 86.7, 80.8, 78.3, 75.7, 75.4, 75.2, 65.4, 58.9, 24.1, 21.2, 19.8, 13.8 ppm.

HRMS *m/z* (ESI+) calculated for $C_{34}H_{35}O_8S_2Na_2^+$, 681.1563, found, 681.1546 [M-NBu_4+2Na]$^+$.

Tetrabutylammonium saccharide derivative sulfate (48)

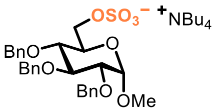

The reaction was carried out according to general procedure **A** with modified conditons: 2.0 equiv DMS and 2.0 equiv Bu_4NHSO_4 were used. The yield of tetrabutylammonium saccharide derivative sulfate **48** was purified by plate chromatography on silica gel eluting with DCM/MeOH (30:1, v/v) to afford the product as a white oil (18.0 mg, 0.023 mmol, 53%).

$\mathbf{R_f}$ = 0.40 (DCM/MeOH = 15:1, v/v).

NMR Spectroscopy:

^{1}**H NMR** (500 MHz, Chloroform-*d*, 298 K, δ): 7.38 - 7.26 (m, 12H), 7.26 - 7.20 (m, 3H), 4.95 (d, *J* = 11.1 Hz, 1H), 4.89 - 4.84 (m, 2H), 4.80 - 4.74 (m, 2H), 4.63 (d, *J* = 12.1 Hz, 1H), 4.57 (d, *J* = 3.5 Hz, 1H), 4.38 (dd, *J* = 10.8, 3.6 Hz, 1H), 4.23 (dd, *J* = 10.8, 2.1 Hz, 1H), 3.96 (t, *J* = 9.3 Hz, 1H), 3.80 - 3.76 (m, 1H), 3.72 - 3.67 (m, 1H), 3.51 (dd, *J* = 9.7, 3.5 Hz, 1H), 3.34 (s, 3H), 3.25 - 3.21 (m, 8H), 1.60 (p, *J* = 8.1, 7.6 Hz, 8H), 1.41 (h, *J* = 7.4 Hz, 8H), 0.97 (t, *J* = 7.4 Hz, 12H) ppm.

^{13}**C NMR** (150 MHz, Chloroform-*d*, 298 K, δ): 139.3, 138.7, 138.4, 128.7, 128.5, 128.4, 128.3, 128.2, 127.9, 127.8, 127.6, 127.4, 98.1, 82.1, 79.7, 77.7, 75.6, 75.1, 73.4, 69.6, 65.4, 58.8, 55.1, 54.3, 24.0, 19.8, 13.8 ppm.

HRMS *m/z* (ESI+) calculated for $C_{28}H_{32}O_9SNa^+$, 567.1659, found, 567.1661 $[M-NBu_4+Na+H]^+$.

Tetrabutylammonium saccharide derivative sulfate (49)

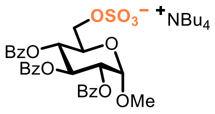

The reaction was carried out according to general procedure **A** with modified conditons: 2.0 equiv DMS and 2.0 equiv Bu_4NHSO_4 were used. The yield of tetrabutylammonium saccharide derivative sulfate **49** was purified by flash chromatography on silica gel eluting with DCM/MeOH (100:1 to 20:1, v/v) to afford the product as a white oil (18.0 mg, 0.022 mmol, 56%).

R*f* = 0.40 (DCM/MeOH = 15:1, v/v).

NMR Spectroscopy:

^{1}**H NMR** (600 MHz, Chloroform-*d*, 298 K, δ): 7.96 (d, *J* = 7.2 Hz, 2H), 7.90 (d, *J* = 6.9 Hz, 2H), 7.83 (d, *J* = 6.9 Hz, 2H), 7.51 - 7.43 (m, 2H), 7.40 - 7.29 (m, 5H), 7.24 (d, *J* = 7.8 Hz, 1H), 6.11 (t, *J* = 9.8 Hz, 1H), 5.51 (d, *J* = 9.8 Hz, 1H), 5.24 (dd, *J* = 10.1, 3.6 Hz, 1H), 5.20 (d, *J* = 3.6 Hz, 1H), 4.45 - 4.38 (m, 1H), 4.27 (d, *J* = 11.2 Hz, 1H), 4.17 (dd, *J* = 11.2, 7.0 Hz, 1H), 3.48 (s, 3H), 3.29 - 3.19 (m, 8H), 1.67 - 1.55 (m, 8H), 1.42 (h, *J* = 7.4 Hz, 8H), 0.98 (t, *J* = 7.4 Hz, 12H) ppm.

^{13}**C NMR** (150 MHz, Chloroform-*d*, 298 K, δ): 166.0, 165.9, 165.5, 133.4, 133.2, 133.0, 130.0, 130.0, 129.8, 129.5, 129.3, 129.3, 128.5, 128.4, 128.3, 96.7, 72.4, 71.0, 69.8, 68.3, 65.9, 58.9, 55.7, 24.1, 19.8, 13.8 ppm.

HRMS *m/z* (ESI+) calculated for $C_{28}H_{26}O_{12}SNa^+$, 609.1037, found, 609.1044 $[M-NBu_4+Na+H]^+$.

Tetrabutylammonium saccharide derivative sulfate (50)

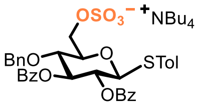

The reaction was carried out according to general procedure **A** with modified conditons: 2.0 equiv DMS and 2.0 equiv Bu_4NHSO_4 were used. The yield of tetrabutylammonium saccharide derivative sulfate **50** was purified by flash chromatography on silica gel eluting with DCM/MeOH (100:1 to 20:1, v/v) to afford the product as a white oil (26.0 mg, 0.029 mmol, 84%).

R_f = 0.40 (DCM/MeOH = 15:1, v/v).

NMR Spectroscopy:

^{1}H NMR (600 MHz, Chloroform-*d*, 298 K, δ): 7.91 (d, *J* = 7.2 Hz, 2H), 7.85 (d, *J* = 7.2 Hz, 2H), 7.52 - 7.43 (m, 2H), 7.35 - 7.30 (m, 6H), 7.21 - 7.14 (m, 2H), 7.07 - 7.03 (m, 5H), 5.67 (t, *J* = 9.4 Hz, 1H), 5.29 (t, *J* = 9.8 Hz, 1H), 4.86 (dd, *J* = 12.3, 10.2 Hz, 2H), 4.53 (d, *J* = 10.5 Hz, 1H), 4.49 (dd, *J* = 11.0, 3.3 Hz, 1H), 4.42 (dd, *J* = 11.0, 2.0 Hz, 1H), 4.05 (t, *J* = 9.5 Hz, 1H), 3.78 - 3.74 (m, 1H), 3.34 - 3.25 (m, 8H), 2.27 (s, 3H), 1.73 - 1.62 (m, 8H), 1.52 - 1.40 (m, 8H), 1.01 (t, *J* = 7.3 Hz, 12H) ppm.

^{13}C NMR (150 MHz, Chloroform-*d*, 298 K, δ): 165.8, 165.5, 138.2, 137.6, 133.3, 133.2, 132.3, 130.0, 130.0, 129.8, 129.6, 129.5, 128.9, 128.4, 128.4, 128.3, 127.7, 87.1, 78.5, 76.3, 75.1, 74.9, 71.1, 65.3, 59.1, 24.2, 21.2, 19.9, 13.9 ppm.

HRMS *m/z* (ESI+) calculated for $C_{34}H_{33}O_{10}S_2^+$, 665.1510, found, 665.1508 [M-NBu$_4$+2H]$^+$.

Tetrabutylammonium saccharide derivative sulfate (51)

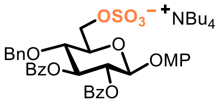

The reaction was carried out according to general procedure **A** with modified conditons: 2.0 equiv DMS and 2.0 equiv Bu_4NHSO_4 were used. The yield of tetrabutylammonium saccharide derivative sulfate **51** was purified by flash chromatography on silica gel eluting with DCM/MeOH (100:1 to 20:1, v/v) to afford the product as a white oil (26.0 mg, 0.029 mmol, 84%).

R_f = 0.40 (DCM/MeOH = 15:1, v/v).

NMR Spectroscopy:

^{1}H NMR (500 MHz, Chloroform-*d*, 298 K, δ): 7.93 - 7.87 (m, 4H), 7.52 - 7.46 (m, 2H), 7.39 - 7.32 (m, 4H), 7.23 (dd, *J* = 6.6, 2.9 Hz, 2H), 7.07 (dd, *J* = 4.8, 1.8 Hz, 3H), 6.87

(d, J = 9.0 Hz, 2H), 6.74 (d, J = 9.1 Hz, 2H), 5.73 (t, J = 9.7 Hz, 1H), 5.49 (dd, J = 10.0, 8.0 Hz, 1H), 5.09 (d, J = 8.0 Hz, 1H), 4.92 (d, J = 10.4 Hz, 1H), 4.65 - 4.51 (m, 2H), 4.39 (dd, J = 11.1, 2.0 Hz, 1H), 4.17 (t, J = 9.5 Hz, 1H), 3.84 (d, J = 9.6 Hz, 1H), 3.72 (s, 3H), 3.71 (s, 1H), 3.25 - 3.21 (m, 8H), 1.64 - 1.58 (m, 8H), 1.44 - 1.39 (m, 8H), 0.98 (t, J = 7.3 Hz, 12H) ppm.

^{13}C NMR (150 MHz, Chloroform-*d*, 298 K, δ): 165.7, 165.5, 155.7, 151.4, 137.5, 133.3, 133.2, 129.9, 129.6, 129.5, 128.9, 128.5, 128.4, 128.3, 127.8, 118.6, 114.8, 101.0, 75.2, 75.0, 74.8, 74.5, 72.1, 65.4, 60.5, 59.0, 55.7, 24.0, 19.9, 13.8 ppm.

HRMS *m/z* (ESI+) calculated for $C_{34}H_{32}O_{12}SNa^+$, 687.1507, found, 687.1503 [M-NBu$_4$+Na+H]$^+$.

Tetrabutylammonium saccharide derivative sulfate (52)

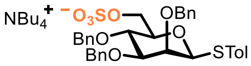

The reaction was carried out according to general procedure **A** with modified conditons: 2.0 equiv DMS and 2.0 equiv Bu_4NHSO_4 were used. The yield of tetrabutylammonium saccharide derivative sulfate **52** was purified by flash chromatography on silica gel eluting with DCM/MeOH (100:1 to 20:1, v/v) to afford the product as a white oil (14.0 mg, 0.016 mmol, 44%).

R_f = 0.40 (DCM/MeOH = 15:1, v/v).

NMR Spectroscopy:

^{1}H NMR (600 MHz, Chloroform-*d*, 298 K, δ): 7.41 (d, J = 7.2 Hz, 2H), 7.34 - 7.27 (m, 15H), 7.24 - 7.22 (m, 1H), 7.06 (d, J = 7.9 Hz, 2H), 5.45 (d, J = 1.8 Hz, 1H), 4.87 (d, J = 10.1 Hz, 1H), 4.82 (d, J = 10.0 Hz, 1H), 4.71 (d, J = 12.0 Hz, 1H), 4.64 (d, J = 11.8 Hz, 1H), 4.58 (d, J = 11.8 Hz, 1H), 4.11 (t, J = 9.5 Hz, 2H), 3.96 (dd, J = 3.1, 1.8 Hz, 1H), 3.83 (dd, J = 9.3, 3.1 Hz, 1H), 3.22 - 3.13 (m, 8H), 2.26 (s, 3H), 1.59 - 1.51 (m, 8H), 1.37 (h, J = 7.4 Hz, 8H), 0.94 (t, J = 7.3 Hz, 12H) ppm.

^{13}C NMR (150 MHz, Chloroform-*d*, 298 K, δ): 138.7, 138.5, 132.6, 130.0, 128.9, 128.5, 128.5, 128.4, 128.3, 127.8, 127.8, 127.7, 127.6, 86.7, 80.2, 75.3, 74.7, 72.6, 72.5, 72.2, 65.7, 58.9, 24.1, 21.2, 19.8, 13.8 ppm.

HRMS *m/z* (ESI+) calculated for $C_{34}H_{36}O_8S_2Na^+$, 659.1744, found, 659.1743 [M-NBu$_4$+Na+H]$^+$.

Tetrabutylammonium saccharide derivative sulfate (53)

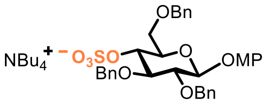

The reaction was carried out according to general procedure **A** with modified conditons: 2.0 equiv DMS and 2.0 equiv Bu_4NHSO_4 were used, 24 h instead of 12 h. The yield of tetrabutylammonium saccharide derivative sulfate **53** was purified by plate chromatography on silica gel eluting with DCM/MeOH (30:1, v/v) to afford the product as a white oil (12.0 mg, 0.014 mmol, 38%).

R_f = 0.40 (DCM/MeOH = 15:1, v/v).

NMR Spectroscopy:

^{1}H NMR (600 MHz, Chloroform-*d*, 298 K, δ): 7.52 - 7.48 (m, 2H), 7.35 - 7.27 (m, 6H), 7.25 - 7.20 (m, 6H), 7.05 (d, *J* = 9.1 Hz, 2H), 6.72 (d, *J* = 9.1 Hz, 2H), 5.33 (d, *J* = 10.8 Hz, 1H), 4.93 (d, *J* = 10.9 Hz, 1H), 4.88 (d, *J* = 7.8 Hz, 1H), 4.76 (d, *J* = 10.9 Hz, 1H), 4.71 (d, *J* = 10.9 Hz, 1H), 4.62 - 4.54 (m, 2H), 4.44 - 4.37 (m, 2H), 3.79 - 3.75 (m, 2H), 3.74 (s, 3H), 3.69 - 3.63 (m, 1H), 3.22 - 3.19 (m, 8H), 1.60 - 1.56 (m, 8H), 1.41 (h, *J* = 7.4 Hz, 8H), 0.98 (t, *J* = 7.3 Hz, 12H) ppm.

^{13}C NMR (150 MHz, Chloroform-*d*, 298 K, δ): 155.1, 151.9, 139.8, 139.3, 138.8, 128.8, 128.4, 128.32, 128.27, 128.0, 127.7, 127.6, 127.3, 127.1, 118.3, 114.6, 102.5, 83.6, 81.7, 76.6, 75.5, 75.3, 73.4, 70.8, 58.9, 55.8, 29.8, 24.1, 19.9, 13.8 ppm.

HRMS *m/z* (ESI+) calculated for $C_{34}H_{36}O_{10}SNa^+$, 659.1921, found, 659.1917 $[M-NBu_4+Na+H]^+$.

Tetrabutylammonium saccharide derivative sulfate (54)

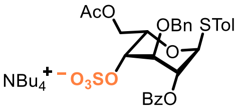

The reaction was carried out according to general procedure **A** with modified conditons: 2.0 equiv DMS and 2.0 equiv Bu_4NHSO_4 were used. The yield of tetrabutylammonium saccharide derivative sulfate **54** was purified by plate chromatography on silica gel eluting with DCM/MeOH (30:1, v/v) to afford the product as a white oil (22.0 mg, 0.026 mmol, 69%).

R_f = 0.40 (DCM/MeOH = 15:1, v/v).

NMR Spectroscopy:

^{1}H NMR (500 MHz, Chloroform-*d*, 298 K, δ): 8.18 (d, *J* = 7.7 Hz, 2H), 7.46 (dd, *J* = 12.5, 7.6 Hz, 6H), 7.37 (t, *J* = 7.6 Hz, 3H), 7.31 (t, *J* = 7.5 Hz, 2H), 7.23 (d, *J* = 7.4 Hz, 1H), 7.06 (d, *J* = 7.8 Hz, 2H), 5.48 (s, 1H), 5.38 (s, 1H), 5.03 (d, *J* = 8.4 Hz, 1H), 4.86 - 4.77 (m, 2H), 4.59 (s, 1H), 4.50 - 4.43 (m, 1H), 4.43 - 4.38 (m, 1H), 4.35 (s, 1H), 3.25

- 3.16 (m, 8H), 2.29 (s, 3H), 2.00 (s, 3H), 1.61 (p, *J* = 7.4 Hz, 8H), 1.40 (h, *J* = 7.4 Hz, 8H), 0.96 (t, *J* = 7.4 Hz, 12H) ppm.

^{13}C NMR (150 MHz, Chloroform-*d*, 298 K, δ): 171.8, 166.0, 137.9, 137.6, 133.2, 132.8, 132.5, 130.5, 129.9, 129.6, 128.5, 128.4, 127.8, 127.7, 86.3, 73.6, 72.7, 70.9, 69.4, 66.0, 64.9, 58.9, 24.1, 21.2, 21.0, 19.8, 13.8 ppm.

HRMS *m/z* (ESI+) calculated for $C_{29}H_{31}O_{10}S_2^+$, 603.1353, found, 603.1343 [M-NBu_4+2H]$^+$.

Tetrabutylammonium saccharide derivative sulfate (55)

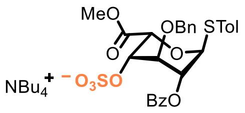

The reaction was carried out according to general procedure **A** with modified conditons: 2.0 equiv DMS and 2.0 equiv Bu_4NHSO_4 were used, 24 h instead of 12 h. The yield of tetrabutylammonium saccharide derivative sulfate **55** was purified by plate chromatography on silica gel eluting with DCM/MeOH (30:1, v/v) to afford the product as a white oil (18.0 mg, 0.022 mmol, 56%).

R_f = 0.40 (DCM/MeOH = 15:1, v/v).

NMR Spectroscopy:

^{1}H NMR (500 MHz, Chloroform-*d*, 298 K, δ): 8.24 (d, *J* = 7.0 Hz, 2H), 7.51 - 7.36 (m, 7H), 7.30 (dd, *J* = 8.3, 6.7 Hz, 2H), 7.23 (t, *J* = 7.3 Hz, 1H), 7.05 (d, *J* = 7.8 Hz, 2H), 5.62 (s, 1H), 5.38 (d, *J* = 2.6 Hz, 1H), 5.37 - 5.35 (m, 1H), 4.91 (t, *J* = 3.2 Hz, 1H), 4.88 - 4.81 (m, 2H), 4.48 (t, *J* = 3.4 Hz, 1H), 3.80 (s, 3H), 3.21 - 3.15 (m, 8H), 2.28 (s, 3H), 1.58 - 1.52 (m, 8H), 1.40 - 1.34 (m, 8H), 0.94 (t, *J* = 7.3 Hz, 12H) ppm.

^{13}C NMR (150 MHz, Chloroform-*d*, 298 K, δ): 169.6, 166.0, 138.2, 137.4, 132.9, 132.2, 130.8, 130.1, 129.7, 128.3, 127.7, 127.5, 86.2, 74.0, 72.7, 71.2, 69.9, 68.9, 58.9, 54.4, 52.1, 24.1, 21.2, 19.8, 13.8 ppm.

HRMS *m/z* (ESI+) calculated for $C_{28}H_{27}O_{10}S_2Na_2^+$, 633.0836, found, 633.0833 [M-NBu_4+2Na]$^+$.

Tetrabutylammonium saccharide derivative sulfate (56)

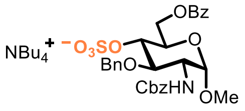

The reaction was carried out according to general procedure **A**. The yield of tetrabutylammonium saccharide derivative sulfate **56** was purified by plate

chromatography on silica gel eluting with DCM/MeOH (30:1, v/v) to afford the product as a white oil (12.0 mg, 0.014 mmol, 38%).

R*f* = 0.40 (DCM/MeOH = 15:1, v/v).

NMR Spectroscopy:

^{1}H NMR (600 MHz, Chloroform-*d*, 298 K, δ): 8.03 (d, *J* = 8.3 Hz, 2H), 7.52 (t, *J* = 7.4 Hz, 1H), 7.44 - 7.28 (m, 9H), 7.22 - 7.16 (m, 3H), 5.23 (d, *J* = 12.3 Hz, 1H), 5.05 (q, *J* = 12.2 Hz, 2H), 4.91 (dd, *J* = 12.0, 2.1 Hz, 1H), 4.75 - 4.60 (m, 4H), 4.51 (t, *J* = 9.5 Hz, 1H), 4.04 (t, *J* = 8.4 Hz, 1H), 3.92 (td, *J* = 10.0, 3.6 Hz, 1H), 3.70 - 3.63 (m, 1H), 3.30 (s, 3H), 3.24 - 3.21 (m, 8H), 1.63 - 1.58 (m, 8H), 1.40 (h, *J* = 7.4 Hz, 8H), 0.97 (t, *J* = 7.3 Hz, 12H) ppm.

^{13}C NMR (150 MHz, Chloroform-*d*, 298 K, δ): 166.6, 156.1, 139.6, 136.6, 132.8, 130.8, 129.7, 128.6, 128.4, 128.22, 128.15, 127.2, 98.5, 78.4, 76.7, 74.4, 69.4, 66.8, 64.9, 58.8, 55.0, 54.6, 29.8, 24.1, 19.8, 13.8, 1.2 ppm.

HRMS *m/z* (ESI+) calculated for $C_{29}H_{30}NO_{11}SNa_2^+$, 646.1329, found, 646.1315 [M-NBu_4+2Na]$^+$.

Tetrabutylammonium saccharide derivative sulfate (57)

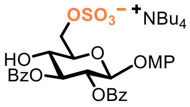

The reaction was carried out according to general procedure **A** with modified conditons: 2.0 equiv Bu_4NHSO_4 were used, 24 h instead of 12 h. The yield of tetrabutylammonium saccharide derivative sulfate **57** was purified by plate chromatography on silica gel eluting with DCM/MeOH (30:1, v/v) to afford the product as a white oil (20.0 mg, 0.024 mmol, 59%).

R*f* = 0.40 (DCM/MeOH = 15:1, v/v).

NMR Spectroscopy:

^{1}H NMR (500 MHz, Chloroform-*d*, 298 K, δ): 7.95 (dd, *J* = 12.6, 6.8 Hz, 4H), 7.51 - 7.46 (m, 2H), 7.35 (t, *J* = 7.8 Hz, 4H), 6.88 (d, *J* = 9.0 Hz, 2H), 6.74 (d, *J* = 9.0 Hz, 2H), 5.66 (t, *J* = 9.8 Hz, 1H), 5.55 (dd, *J* = 9.9, 7.9 Hz, 1H), 5.10 (d, *J* = 7.9 Hz, 1H), 4.68 (dd, *J* = 12.9, 2.5 Hz, 1H), 4.29 (t, *J* = 9.6 Hz, 1H), 4.21 (dd, *J* = 12.9, 2.0 Hz, 1H), 3.73 (s, 3H), 3.70 - 3.66 (m, 1H), 3.22 - 3.18 (m, 8H), 1.63 - 1.58 (m, 8H), 1.41 (h, *J* = 7.3 Hz, 8H), 0.98 (t, *J* = 7.3 Hz, 12H) ppm.

^{13}C NMR (150 MHz, Chloroform-*d*, 298 K, δ): 166.1, 165.6, 155.7, 151.5, 133.3, 133.0, 130.0, 129.9, 129.9, 129.6, 128.5, 128.3, 118.8, 114.6, 101.4, 76.2, 74.3, 72.3, 68.0, 65.4, 58.9, 55.8, 24.0, 19.8, 13.8 ppm.

HRMS *m/z* (ESI+) calculated for $C_{27}H_{26}O_{12}SNa^+$, 597.1037, found, 597.1040 $[M-NBu_4+Na+H]^+$.

Tetrabutylammonium saccharide derivative sulfate (58)

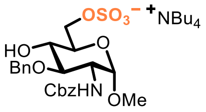

The reaction was carried out according to general procedure **A** with modified conditons: 2.0 equiv Bu_4NHSO_4 was used. The yield of tetrabutylammonium saccharide derivative sulfate **58** was purified by flash chromatography on silica gel eluting with DCM/MeOH (100:1 to 20:1, v/v) to afford the product as a white oil (17.0 mg, 0.023 mmol, 49%).

R_f = 0.40 (DCM/MeOH = 15:1, v/v).

NMR Spectroscopy:

^{1}H NMR (600 MHz, Chloroform-*d*, 298 K, δ): 7.33 - 7.27 (m, 7H), 7.25 - 7.15 (m, 3H), 5.09 (d, *J* = 12.2 Hz, 1H), 5.03 (d, *J* = 12.4 Hz, 1H), 4.92 (d, *J* = 11.3 Hz, 2H), 4.71 - 4.63 (m, 2H), 4.58 (d, *J* = 12.5 Hz, 1H), 4.09 (d, *J* = 12.7 Hz, 1H), 3.96 (t, *J* = 9.5 Hz, 1H), 3.87 (t, *J* = 10.6 Hz, 1H), 3.64 (d, *J* = 9.7 Hz, 1H), 3.54 (t, *J* = 9.7 Hz, 1H), 3.29 (s, 3H), 3.24 - 3.12 (m, 8H), 1.66 - 1.57 (m, 8H), 1.47 - 1.39 (m, 8H), 1.00 (t, *J* = 7.0 Hz, 12H) ppm.

^{13}C NMR (150 MHz, Chloroform-*d*, 298 K, δ): 156.3, 139.3, 136.6, 128.6, 128.3, 128.2, 128.2, 127.9, 127.4, 99.2, 79.4, 74.5, 71.5, 70.7, 66.9, 66.0, 59.1, 55.2, 54.7, 24.1, 19.9, 13.8 ppm.

HRMS *m/z* (ESI+) calculated for $C_{22}H_{27}O_{10}SNa^+$, 520.1248; found, 520.1253 $[M-NBu_4+Na+H]^+$.

Tetrabutylammonium saccharide derivative sulfate (59)

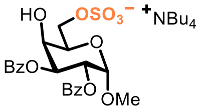

The reaction was carried out according to general procedure **A** with modified conditons: 2.0 equiv DMS and 2.0 equiv Bu_4NHSO_4 were used. The yield of tetrabutylammonium saccharide derivative sulfate **59** was purified by plate chromatography on silica gel eluting with DCM/MeOH (30:1, v/v) to afford the product as a white oil (17.0 mg, 0.024 mmol, 49%).

R_f = 0.40 (DCM/MeOH = 15:1, v/v).

NMR Spectroscopy:

^{1}H NMR (600 MHz, Chloroform-*d*, 298 K, δ): 8.02 - 7.94 (m, 4H), 7.50 - 7.45 (m, 2H), 7.37 - 7.32 (m, 4H), 5.71 (dd, *J* = 10.8, 3.4 Hz, 1H), 5.65 (dd, *J* = 10.8, 3.6 Hz, 1H), 5.14 (d, *J* = 3.6 Hz, 1H), 4.57 (d, *J* = 3.1 Hz, 1H), 4.43 - 4.39 (m, 1H), 4.12 (dd, *J* = 9.7, 5.1 Hz, 1H), 4.05 (dd, *J* = 10.9, 5.1 Hz, 1H), 3.39 (s, 3H), 3.24 (d, *J* = 4.2 Hz, 8H), 1.64 - 1.61 (m, 8H), 1.44 - 1.40 (m, 8H), 0.98 (t, *J* = 7.4 Hz, 12H) ppm.

^{13}C NMR (150 MHz, Chloroform-*d*, 298 K, δ): 166.2, 166.0, 133.2, 133.0, 130.2, 130.0, 129.9, 129.8, 128.4, 128.4, 97.8, 70.3, 69.5, 68.3, 66.9, 64.0, 59.0, 55.6, 24.1, 19.8, 13.8 ppm.

HRMS *m/z* (ESI+) calculated for $C_{21}H_{22}O_{11}SNa^+$, 505.0775; found, 505.0773 [M-NBu_4+Na+H]$^+$.

Sulfation for substrate with similar hindrance

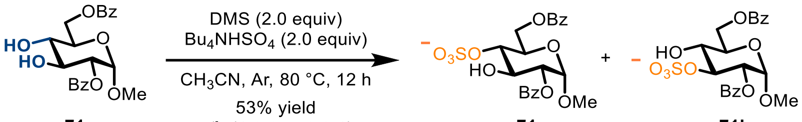

The reaction was carried out according to general procedure **A** with modified conditons: 2.0 equiv Bu_4NHSO_4 and 2.0 equiv DMS were used. The yield of tetrabutylammonium saccharide derivative sulfate **71a** and **b** was purified by flash chromatography on silica gel eluting with DCM/MeOH (100:1 to 20:1, v/v) to afford the mixture as a white solid (76.7 mg, 0.106 mmol, 53%).

R_f = 0.30 (DCM/MeOH = 15:1, v/v).

NMR Spectroscopy:

^{1}H NMR (400 MHz, DMSO-d_6, 298 K, δ): 8.07 (d, *J* = 7.8 Hz, 2H), 7.99 (t, *J* = 7.2 Hz, 5H), 7.70 - 7.64 (m, 3H), 7.59 - 7.50 (m, 7H), 5.88 (s, 1H), 5.54 (s, 1H), 5.03 (d, *J* = 3.6 Hz, 1H), 4.96 (d, *J* = 3.7 Hz, 1H), 4.91 – 4.86 (m, 1H), 4.82 (dd, *J* = 10.0, 3.6 Hz, 1H), 4.68 – 4.58 (m, 3H), 4.42 (dd, *J* = 11.9, 6.1 Hz, 1H), 4.35 (dd, *J* = 12.1, 6.6 Hz, 1H), 4.20 (t, *J* = 9.4 Hz, 1H), 4.09 (t, *J* = 9.2 Hz, 1H), 3.95 – 3.83 (m, 2H), 3.70 (t, *J* = 9.3 Hz, 1H), 3.32 (s, 5H), 3.15 (dd, *J* = 8.1, 4.1 Hz, 13H), 1.59 – 1.53 (m, 13H), 1.32 – 1.27 (m, 13H), 0.92 (t, *J* = 7.3 Hz, 19H) ppm.

^{13}C NMR (101 MHz, DMSO-d_6, 298 K, δ): 165.6, 165.3, 133.6, 133.5, 133.5, 133.3, 129.73, 129.68, 129.5, 129.40, 129.35, 129.3, 129.2, 128.9, 128.81, 128.76, 128.6,

96.3, 96.0, 76.0, 75.1, 73.6, 71.2, 69.82, 69.75, 69.4, 67.4, 63.6, 63.5, 57.6, 57.54, 57.51, 54.7, 54.5, 52.8, 23.1, 19.2, 13.5 ppm.

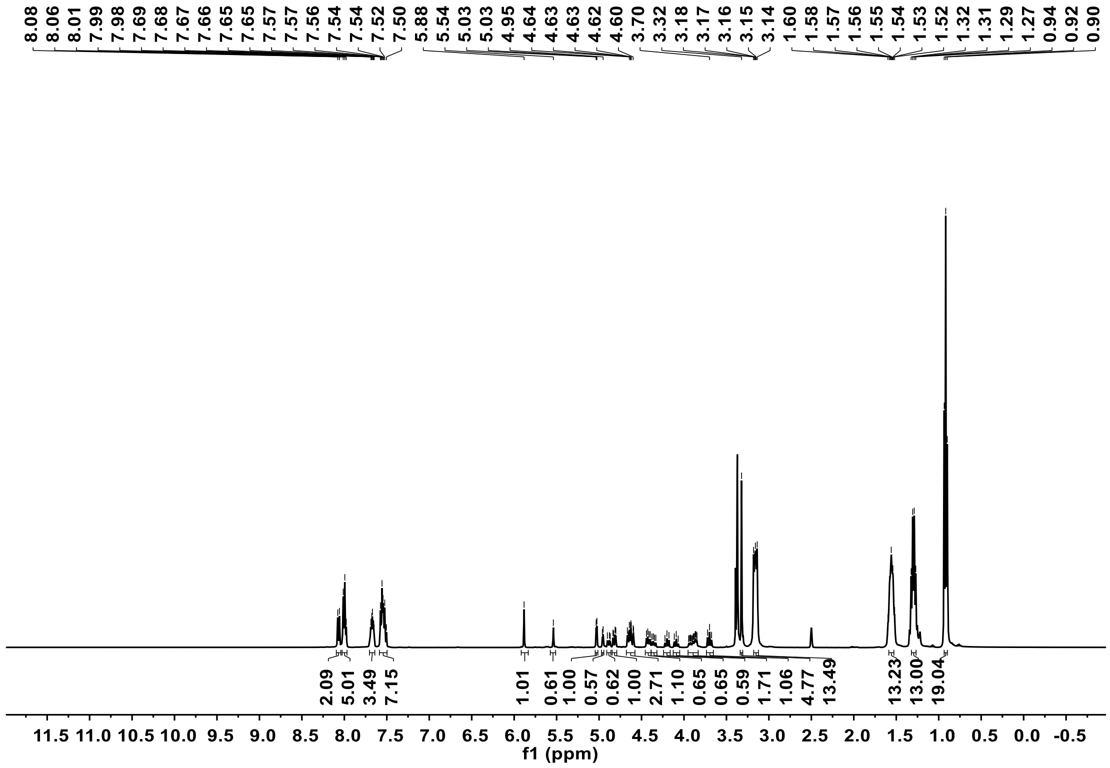

Supplementary Figure 7. ^{1}H-NMR spectrum of **71a** and **71b**.

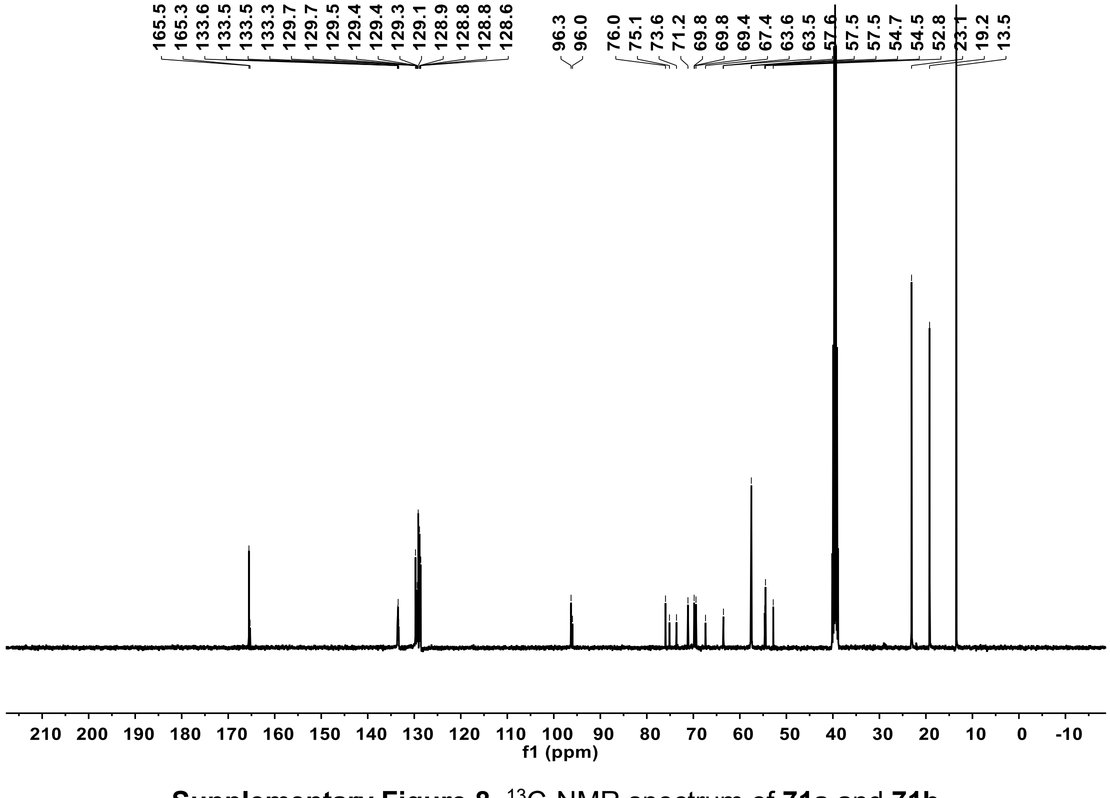

Supplementary Figure 8. ^{13}C-NMR spectrum of **71a** and **71b**.

Tetrabutylammonium 4-(4-(3-hydroxypropoxy)phenyl)-2-methylbutan-2-ol sulfate (60)

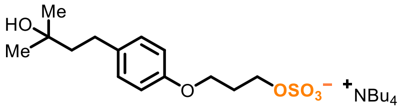

The reaction was carried out according to general procedure **A** with modified conditons: 2.0 equiv DMS and 2.0 equiv Bu_4NHSO_4 were used. The yield of tetrabutylammonium 4-(4-(3-hydroxypropoxy)phenyl)-2-methylbutan-2-ol sulfate **60** was purified by plate chromatography on silica gel eluting with DCM/MeOH (30:1, v/v) to afford the product as a white oil (57.3 mg, 0.102 mmol, 51%).

R*f* = 0.30 (DCM/MeOH = 15:1, v/v).

NMR Spectroscopy:

^{1}H NMR (400 MHz, Chloroform-*d*, 298 K, δ): 7.04 (d, *J* = 8.3 Hz, 2H), 6.76 (d, *J* = 8.1 Hz, 2H), 4.15 (t, *J* = 6.3 Hz, 2H), 4.03 (t, *J* = 6.6 Hz, 2H), 3.25 – 3.19 (m, 8H), 2.63 – 2.54 (m, 2H), 2.11 – 2.06 (m, 2H), 1.75 – 1.68 (m, 2H), 1.61 - 1.54 (m, 8H), 1.39 (h, *J* = 7.4 Hz, 8H), 1.24 (s, 6H), 0.95 (t, *J* = 7.4 Hz, 12H) ppm.

^{13}C NMR (101 MHz, Chloroform-*d*, 298 K, δ): 157.2, 134.4, 129.1, 114.5, 70.8, 65.1, 64.0, 58.7, 46.0, 29.9, 29.7, 29.3, 24.0, 19.7, 13.7 ppm.

HRMS *m/z* (ESI-) calculated for $C_{14}H_{21}O_6S^-$, 317.1064, found, 317.1034 $[M\text{-}NBu_4]^-$.

Sodium 4-hydroxyphenethyl alcohol sulfate (61)

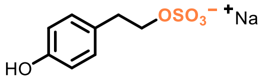

The reaction was carried out according to general procedure **B**. The yield of tetrabutylammonium 4-hydroxyphenethyl alcohol sulfate **61** was determined by ^{1}H NMR integration relative to the internal standard (88% yield; standard: δ 4.94 ppm, **61**: δ 4.14 (t, *J* = 7.2 Hz) ppm). Then the reaction was purified by plate chromatography on silica gel eluting with DCM/MeOH (20:1, v/v) to afford the product as a white oil (64.4 mg, 0.140 mmol, 70%). It was eluted with MeOH through an activated Na^+ resin, and then continue to repeat the above operation to afford the product as a white solid.

R*f* = 0.20 (DCM/MeOH = 10:1, v/v).

NMR Spectroscopy:

^{1}H NMR (400 MHz, Methanol-*d$_4$*, 298 K, δ): 7.06 (d, *J* = 8.0 Hz, 2H), 6.69 (d, *J* = 7.9 Hz, 2H), 4.12 (t, *J* = 7.2 Hz, 2H), 2.87 (t, *J* = 7.4 Hz, 2H) ppm.

^{13}C NMR (101 MHz, Methanol-*d$_4$*, 298 K, δ): 157.0, 130.9, 130.0, 116.2, 70.1, 35.9 ppm.

HRMS *m/z* (ESI-) calculated for $C_8H_9O_5S^-$, 217.0176, found, 217.0165 [M-Na]$^-$.

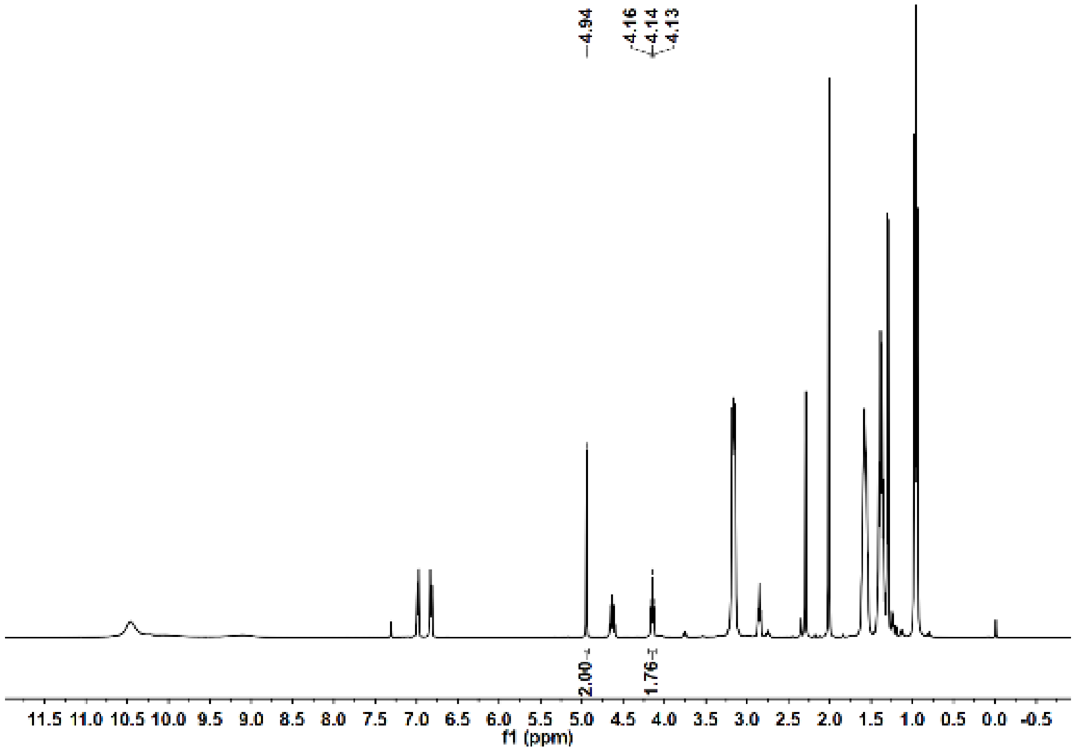

Supplementary Figure 9. NMR yield of sulfation for **61**.

Tetrabutylammonium proline derivative sulfate (62)

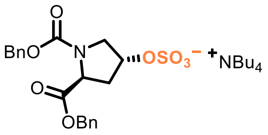

The reaction was carried out according to general procedure **B**. The yield of tetrabutylammonium proline derivative sulfate **62** was purified by plate chromatography on silica gel eluting with DCM/MeOH (30:1, v/v) to afford the product as a white oil (82.7 mg, 0.122 mmol, 61%).

R*f* = 0.40 (DCM/MeOH = 15:1, v/v).

NMR Spectroscopy:

^{1}H NMR (400 MHz, Chloroform-*d*, 298 K, δ): 7.33 - 7.27 (m, 5H), 7.25 - 7.20 (m, 4H), 7.18 - 7.13 (m, 1H), 5.22 - 5.06 (m, 2H), 5.04 - 5.00 (m, 1H), 4.95 (dd, *J* = 12.3, 4.4 Hz, 2H), 4.50 (dt, *J* = 15.3, 8.0 Hz, 1H), 3.92 (dd, *J* = 25.4, 12.0 Hz, 1H), 3.76 - 3.65 (m, 1H), 3.24 - 3.16 (m, 8H), 2.78 - 2.66 (m, 1H), 2.17 - 2.06 (m, 1H), 1.66 - 1.54 (m, 8H), 1.40 (h, *J* = 7.3 Hz, 8H), 0.97 (t, *J* = 7.3 Hz, 12H) ppm.

^{13}C NMR (101 MHz, Chloroform-*d*, 298 K, δ): 172.8, 172.5, 155.0, 154.4, 136.7, 136.5, 135.8, 135.6, 128.6, 128.5, 128.4, 128.3, 128.2, 128.1, 128.0, 127.9, 127.8, 127.8, 75.2, 67.1, 66.8, 66.8, 58.8, 58.4, 58.0, 53.5, 53.1, 37.5, 36.6, 24.0, 19.8, 13.7 ppm.

HRMS *m/z* (ESI-) calculated for $C_{20}H_{20}NO_8S^-$, 434.0915, found, 434.0900 $[M-NBu_4]^-$.

Tetrabutylammonium serine derivative sulfate (63)

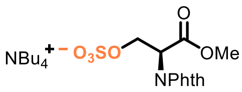

The reaction was carried out according to general procedure **B**. The yield of tetrabutylammonium serine derivative sulfate **63** was purified by flash chromatography on silica gel eluting with DCM/MeOH (100:1 to 20:1, v/v) to afford the product as a white oil (85.7 mg, 0.150 mmol, 75%).

$\mathbf{R_f}$ = 0.40 (DCM/MeOH = 15:1, v/v).

NMR Spectroscopy:

^{1}**H NMR** (400 MHz, Chloroform-*d*, 298 K, δ): 7.78 (dd, *J* = 5.5, 3.1 Hz, 2H), 7.69 (dd, *J* = 5.5, 3.1 Hz, 2H), 5.31 (dd, *J* = 10.2, 4.4 Hz, 1H), 4.75 (dd, *J* = 11.3, 4.4 Hz, 1H), 4.62 (dd, *J* = 11.4, 10.2 Hz, 1H), 3.69 (s, 3H), 3.23 - 3.18 (m, 8H), 1.62 - 1.55 (m, 8H), 1.39 - 1.33 (m, 8H), 0.93 (t, *J* = 7.3 Hz, 12H) ppm.

^{13}**C NMR** (101 MHz, Chloroform-*d*, 298 K, δ): 168.0, 167.4, 134.1, 132.1, 123.4, 63.3, 58.6, 52.8, 52.2, 23.9, 19.7, 13.7 ppm.

HRMS *m/z* (ESI-) calculated for $C_{12}H_{10}NO_8S^-$, 328.0133, found, 328.0122 $[M-NBu_4]^-$.

Tetrabutylammonium picaridin sulfate (64)

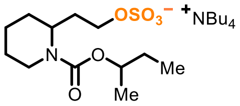

The reaction was carried out according to general procedure **A**. The yield of tetrabutylammonium picaridin sulfate **64** was determined by ^{1}H NMR integration relative to the internal standard (60% yield; standard: δ 4.94 ppm, **61**: δ 4.66 (h, *J* = 6.2 Hz) ppm). Then the reaction was purified by plate chromatography on silica gel eluting with DCM/MeOH (30:1, v/v) to afford the product as a light yellow oil (49.7 mg, 0.090 mmol, 45%).

$\mathbf{R_f}$ = 0.40 (DCM/MeOH = 15:1, v/v).

NMR Spectroscopy:

^{1}**H NMR** (400 MHz, Chloroform-*d*, 298 K, δ): 4.66 (h, *J* = 6.2 Hz, 1H), 4.39 - 4.27 (m, 1H), 4.06 - 3.89 (m, 3H), 3.34 - 3.16 (m, 8H), 2.81 (t, *J* = 13.0 Hz, 1H), 2.05 - 1.84 (m, 2H), 1.67 - 1.48 (m, 15H), 1.40 (h, *J* = 7.4 Hz, 9H), 1.15 (t, *J* = 6.2 Hz, 3H), 0.97 (t, *J* = 7.3 Hz, 12H), 0.85 (td, *J* = 7.4, 3.1 Hz, 3H) ppm.

^{13}C NMR (101 MHz, Chloroform-*d*, 298 K, δ): 155.5, 72.8, 64.9, 58.7, 48.3, 39.2, 29.7, 29.1, 28.2, 25.6, 24.0, 19.9, 19.8, 19.1, 13.8, 9.84, 9.81 ppm.

HRMS *m/z* (ESI-) calculated for $C_{12}H_{22}NO_6S^-$, 308.1173, found, 208.1164 $[M\text{-}NBu_4]^-$.

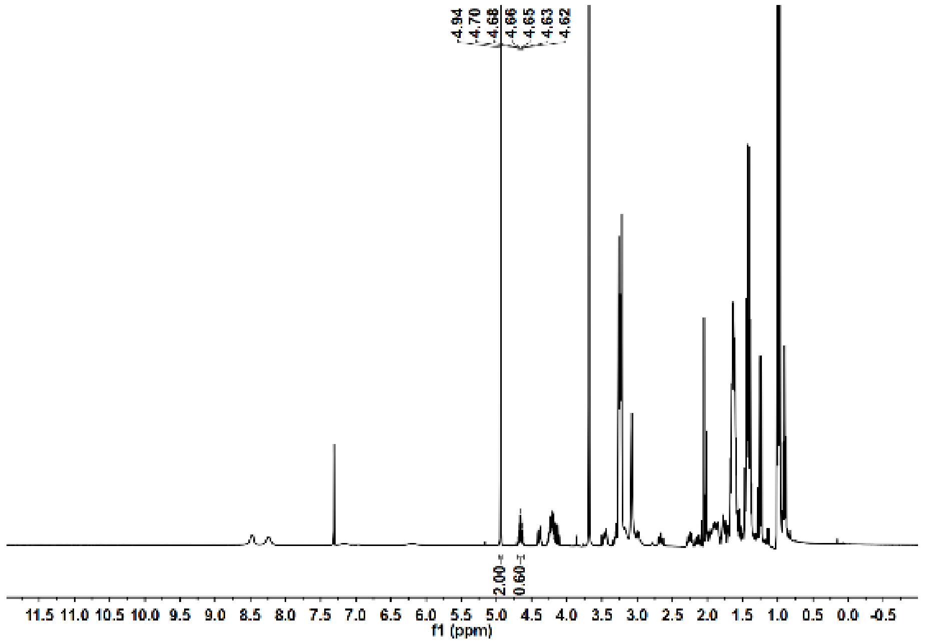

Supplementary Figure 10. NMR yield of sulfation for **64**.

Tetrabutylammonium aloe emodin sulfate (65)

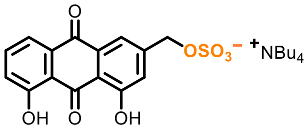

The reaction was carried out according to general procedure **B**. The yield of tetrabutylammonium aloe emodin sulfate **65** was purified by plate chromatography on silica gel eluting with DCM/MeOH (30:1, v/v) to afford the product as a yellow solid (73.4 mg, 0.124 mmol, 62%).

R*f* = 0.20 (DCM/MeOH = 15:1, v/v).

NMR Spectroscopy:

^{1}H NMR (400 MHz, Chloroform-*d*, 298 K, δ): 12.01 (s, 1H), 11.95 (s, 1H), 7.76 - 7.70 (m, 2H), 7.65 - 7.58 (m, 1H), 7.34 (s, 1H), 7.22 (dd, *J* = 8.4, 1.2 Hz, 1H), 5.10 (s, 2H), 3.28 - 3.20 (m, 8H), 1.66 - 1.56 (m, 8H), 1.38 (h, *J* = 7.3 Hz, 8H), 0.93 (t, *J* = 7.4 Hz, 12H) ppm.

^{13}C NMR (101 MHz, Chloroform-*d*, 298 K, δ): 192.6, 181.6, 162.8, 162.5, 149.3, 137.1, 133.6, 133.3, 124.6, 122.4, 119.9, 118.6, 115.8, 114.7, 67.4, 58.7, 23.9, 19.7, 13.7 ppm.

HRMS *m/z* (ESI-) calculated for $C_{15}H_9O_8S^-$, 349.0024, found, 349.0009 $[M\text{-}NBu_4]^-$.

Tetrabutylammonium ospemifene sulfate (66)

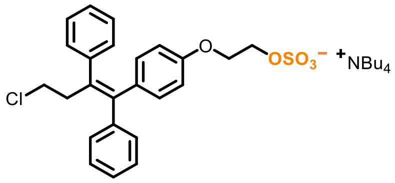

The reaction was carried out according to general procedure **B**. The yield of tetrabutylammonium ospemifene sulfate **66** was purified by plate chromatography on silica gel eluting with DCM/MeOH (30:1, v/v) to afford the product as a light yellow oil (111.9 mg, 0.160 mmol, 80%).

$\mathbf{R}_f$ = 0.40 (DCM/MeOH = 15:1, v/v).

NMR Spectroscopy:

^{1}**H NMR** (400 MHz, Chloroform-*d*, 298 K, δ): 7.31 - 7.25 (m, 2H), 7.24 - 7.18 (m, 3H), 7.15 - 7.03 (m, 5H), 6.68 (d, *J* = 8.8 Hz, 2H), 6.44 (d, *J* = 8.8 Hz, 2H), 4.18 (t, *J* = 5.1 Hz, 2H), 3.99 (t, *J* = 5.1 Hz, 2H), 3.33 (t, *J* = 7.4 Hz, 2H), 3.17 - 3.11 (m, 8H), 2.83 (t, *J* = 7.5 Hz, 2H), 1.55 - 1.47 (m, 8H), 1.30 (h, *J* = 7.4 Hz, 8H), 0.86 (t, *J* = 7.3 Hz, 12H) ppm.

^{13}**C NMR** (101 MHz, Chloroform-*d*, 298 K, δ): 157.1, 142.9, 141.7, 140.9, 135.1, 134.8, 131.6, 129.5, 129.4, 128.3, 128.2, 126.9, 126.6, 113.5, 66.9, 65.2, 58.6, 42.9, 38.6, 23.9, 19.7, 13.7 ppm.

HRMS *m/z* (ESI-) calculated for $C_{24}H_{22}ClO_5S^-$, 457.0882, found, 457.0868 $[M\text{-}NBu_4]^-$.

Tetrabutylammonium testosterone sulfate (67)

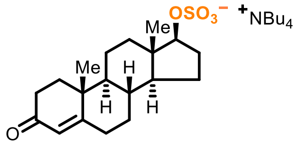

The reaction was carried out according to general procedure B. The yield of tetrabutylammonium testosterone sulfate **67** was purified by flash chromatography on silica gel eluting with DCM/MeOH (100:1 to 20:1, v/v) to afford the product as a light yellow oil (107.1 mg, 0.176 mmol, 88%).

$\mathbf{R}_f$ = 0.40 (DCM/MeOH = 15:1, v/v).

NMR Spectroscopy:

^{1}**H NMR** (400 MHz, Chloroform-*d*, 298 K, δ): 5.70 (s, 1H), 4.28 (t, *J* = 8.5 Hz, 1H), 3.30 - 3.25 (m, 8H), 2.47 - 2.16 (m, 5H), 2.12 - 2.01 (m, 2H), 1.87 - 1.74 (m, 2H), 1.68 - 1.60

(m, 9H), 1.57 - 1.49 (m, 2H), 1.44 (q, J = 7.4 Hz, 9H), 1.40 - 1.35 (m, 1H), 1.34 - 1.20 (m, 3H), 1.17 (s, 3H), 1.00 (t, J = 7.3 Hz, 12H), 0.95 - 0.87 (m, 2H), 0.84 (s, 3H) ppm.

^{13}C NMR (101 MHz, Chloroform-*d*, 298 K, δ): 199.8, 171.9, 123.8, 85.5, 58.9, 54.0, 50.3, 42.7, 38.8, 36.6, 35.8, 35.7, 34.1, 33.0, 31.7, 28.4, 24.1, 23.5, 20.7, 19.9, 17.5, 13.8, 11.8 ppm.

HRMS *m/z* (ESI-) calculated for $C_{19}H_{27}O_5S^-$, 367.1585, found, 367.1577 $[M\text{-}NBu_4]^-$.

Tetrabutylammonium diosgenin sulfate (68)

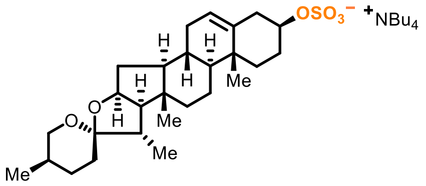

The reaction was carried out according to general procedure A. The yield of tetrabutylammonium diosgenin sulfate **68** was determined by ^{1}H NMR integration relative to the internal standard (90% yield; standard: δ 4.94 ppm, **68**: δ 4.38 (q, J = 7.4 Hz) ppm). Then the reaction was purified by plate chromatography on silica gel eluting with DCM/MeOH (30:1, v/v) to afford the product as a white solid (104.5 mg, 0.142 mmol, 71%).

R*f* = 0.40 (DCM/MeOH = 15:1, v/v).

NMR Spectroscopy:

^{1}H NMR (400 MHz, Chloroform-*d*, 298 K, δ): 5.28 (s, 1H), 4.35 (q, J = 7.4 Hz, 1H), 4.21 - 4.10 (m, 1H), 3.42 (d, J = 10.8 Hz, 1H), 3.32 (t, J = 10.9 Hz, 1H), 3.27 - 3.16 (m, 8H), 2.55 (d, J = 16.4 Hz, 1H), 2.38 (s, 1H), 2.33 - 2.22 (m, 1H), 2.08 (d, J = 11.0 Hz, 1H), 1.99 - 1.88 (m, 2H), 1.82 (t, J = 6.9 Hz, 1H), 1.76 - 1.67 (m, 2H), 1.64 - 1.52 (m, 14H), 1.45 - 1.33 (m, 11H), 1.22 (dt, J = 12.1, 6.6 Hz, 1H), 1.16 - 1.01 (m, 3H), 0.99 - 0.90 (m, 19H), 0.74 (d, J = 6.1 Hz, 6H) ppm.

^{13}C NMR (101 MHz, Chloroform-*d*, 298 K, δ): 140.9, 121.4, 109.3, 80.9, 66.8, 62.1, 58.6, 56.5, 50.0, 41.6, 40.3, 39.8, 39.5, 37.3, 36.7, 32.1, 31.9, 31.4, 31.4, 30.3, 29.1, 28.8, 24.0, 20.8, 19.8, 19.4, 17.2, 16.3, 14.6, 13.8 ppm.

HRMS *m/z* (ESI-) calculated for $C_{27}H_{41}O_6S^-$, 493.2629, found, 493.2622 $[M\text{-}NBu_4]^-$.

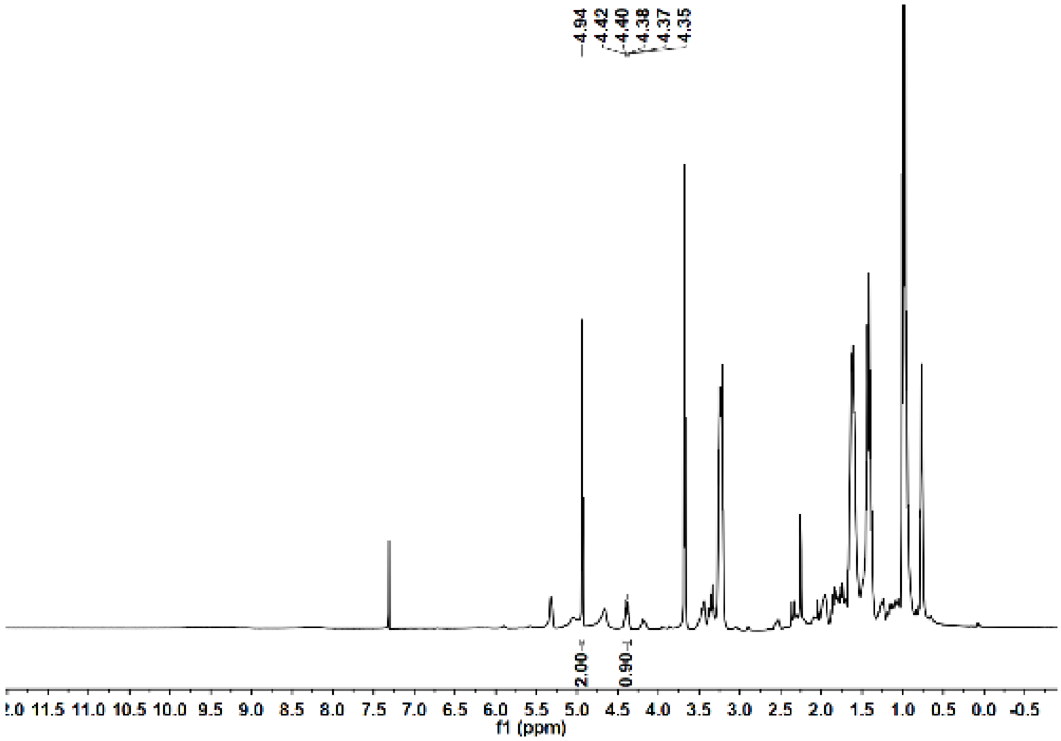

Supplementary Figure 11. NMR yield of sulfation for **68**.

Tetrabutylammonium cholesterol sulfate (69)

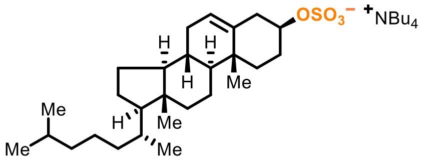

The reaction was carried out according to general procedure **B**. The yield of tetrabutylammonium cholesterol sulfate **69** was determined by ^{1}H NMR integration relative to the internal standard (49% yield; standard: δ 4.94 ppm, **69**: δ 4.20 - 4.14 (m) ppm). Then the reaction was purified by plate chromatography on silica gel eluting with DCM/MeOH (30:1, v/v) to afford the product as a white solid (53.8 mg, 0.076 mmol, 38%).

R*f* = 0.40 (DCM/MeOH = 15:1, v/v).

NMR Spectroscopy:

^{1}H NMR (400 MHz, Chloroform-*d*, 298 K, δ): 5.31 (s, 1H), 4.27 - 4.11 (m, 1H), 3.31 - 3.20 (m, 8H), 2.57 (d, *J* = 15.9 Hz, 1H), 2.32 (t, *J* = 12.6 Hz, 1H), 2.17 - 2.06 (m, 2H), 2.00 - 1.87 (m, 2H), 1.84 - 1.74 (m, 2H), 1.68 - 1.57 (m, 9H), 1.51 - 1.38 (m, 12H), 1.36 - 1.26 (m, 3H), 1.15 - 0.93 (m, 25H), 0.88 (d, *J* = 6.4 Hz, 4H), 0.84 (d, *J* = 6.6 Hz, 6H), 0.64 (s, 3H) ppm.

^{13}**C NMR** (101 MHz, Chloroform-*d*, 298 K, δ): 141.0, 121.7, 58.8, 56.8, 56.2, 50.2, 42.4, 39.9, 39.6, 37.4, 36.6, 36.3, 35.9, 32.0, 31.97, 29.2, 28.3, 28.1, 24.4, 24.1, 23.9, 22.9, 22.7, 21.1, 19.8, 19.5, 18.8, 13.8, 12.0 ppm.

HRMS *m/z* (ESI-) calculated for $C_{27}H_{45}O_4S^-$, 465.3044, found, 465.3041 $[M\text{-}NBu_4]^-$.

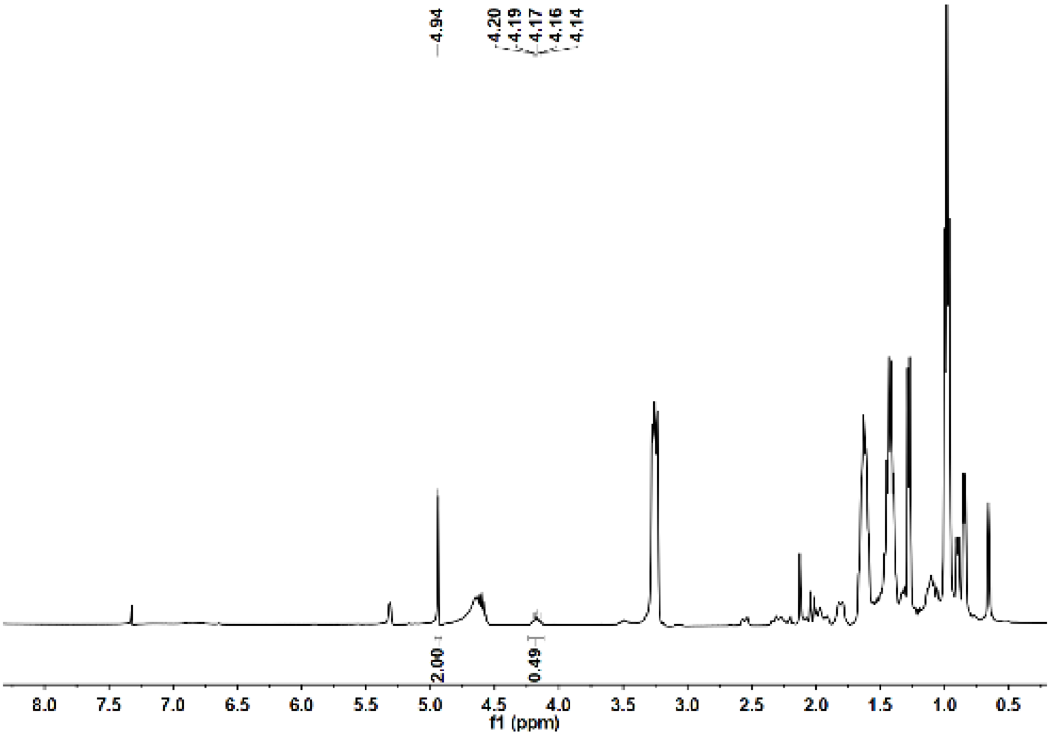

Supplementary Figure 12. NMR yield of sulfation for **69**.

N-tosyl-4-piperidinol (27a) [1]

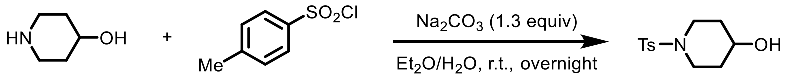

27s **PTSC** **27a**

N-tosyl-4-piperidinol (**27a**) was prepared according to previous report. [1] To a solution of 4-hydroxypiperidine (1.01 g, 10 mmol, 1.0 equiv) and Na_2CO_3 (1.38 g, 13 mmol, 1.3 equiv) in distilled water (50 mL) was added slowly a solution of tosyl chloride (2.29 g, 12 mmol, 1.2 equiv) in Et_2O (50 mL). The reaction was stirred at rt overnight. The crude mixture was purified by flashcolumn chromatography using PE/EtOAc (4:1, v/v) to afford **27a** as a white solid (2.09 g, 8.2 mmol, 82%).

R*f* = 0.35 (PE/EA = 5:1, v/v).

NMR Spectroscopy:

^{1}**H NMR** (400 MHz, Chloroform-*d*, 298 K, δ): 7.63 (d, *J* = 8.0 Hz, 2H), 7.31 (d, *J* = 7.9 Hz, 2H), 3.74 (tt, *J* = 7.6, 3.7 Hz, 1H), 3.35 - 3.26 (m, 2H), 2.88 - 2.77 (m, 2H), 2.43 (s, 3H), 1.97 - 1.86 (m, 2H), 1.69 - 1.59 (m, 2H) ppm.

^{13}C NMR (101 MHz, Chloroform-*d*, 298 K, δ): 143.7, 133.4, 129.8, 127.8, 66.1, 43.3, 33.4, 21.6 ppm.

4-(4-(3-hydroxypropoxy)phenyl)-2-methylbutan-2-ol (60a) [2]

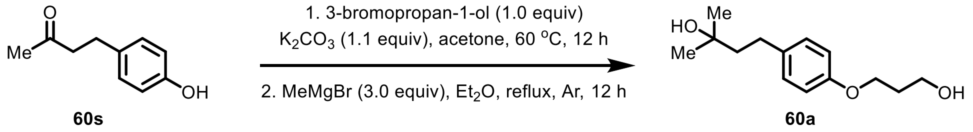

4-(4-(3-hydroxypropoxy)phenyl)-2-methylbutan-2-ol (**60a**) was prepared according to previous report. [2] Raspberry ketone (1.64 g, 10 mmol,1.0 equiv) was dissolved in acetone (30 mL) and 3-bromopropan-1-ol (1.38 g, 10 mmol, 1 equiv), potassium carbonate (1.52 g, 11 mmol, 1.1 equiv) were sequentially added. The reaction mixture was heated at 60 °C for 12 h. After cooling, the solid was filtered off and water was added and the mixture was extracted with EtOAc (3 × 50 mL). The combined organic extracts were dried over Na_2SO_4, concentrated under reduced pressure, then transfered to a 3-necked 50-mL round bottomed flask with a magnetic stirrer. The vial was evacuated and backfilled with argon for three times, then Et_2O (c = 0.2 M) was added. After the dropwise addition of methylmagnesium bromide (3 M in Et_2O, 3.0 equiv), the mixture was refluxed for 12 h. After cooling to room temperature, water (20 mL) was added carefully and the mixture was extracted with EtOAc (3 × 30 mL). The combined organic layers were dried over Na_2SO_4, filtered, and concentrated under reduced pressure. The residue was purified by flashcolumn chromatography using PE/EtOAc (5:1, v/v) to afford **60a** as a white solid (1.13 g, 4.7 mmol, 47%).

R*f* = 0.40 (PE/EA = 5:1, v/v).

NMR Spectroscopy:

^{1}**H NMR** (400 MHz, Chloroform-*d*, 298 K, δ): 7.10 (d, *J* = 8.2 Hz, 2H), 6.83 (d, *J* = 8.7 Hz, 2H), 4.09 (t, *J* = 6.0 Hz, 2H), 3.85 (t, *J* = 5.9 Hz, 2H), 2.67 – 2.59 (m, 2H), 2.02 (p, *J* = 5.9 Hz, 2H), 1.79 – 1.71 (m, 2H), 1.27 (s, 6H) ppm.

^{13}**C NMR** (101 MHz, Chloroform-*d*, 298 K, δ): 157.0, 134.9, 129.3, 114.6, 71.0, 66.0, 60.7, 46.0, 32.1, 29.9, 29.4 ppm.

Methyl (2*S*)-2-(1,3-dioxoisoindolin-2-yl)-3-hydroxypropanoate (63a) [3]

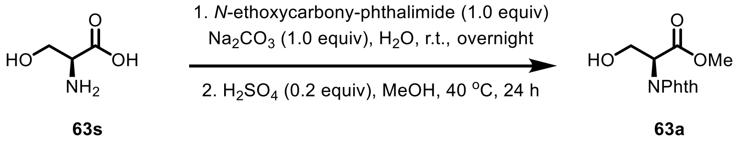

Methyl (2*S*)-2-(1,3-dioxoisoindolin-2-yl)-3-hydroxypropanoate (**63a**) was prepared according to previous report. [3] L-serine (1.05 g, 10.0 mmol, 1.0 equiv), sodium carbonate (1.06 g, 10.0 mmol, 1.0 equiv) and *N*-ethoxycarbonylphthalimide (2.19 g, 10.0 mmol, 1.0 equiv) was reacted in H_2O (50 mL) at rt overnight, then the above solution was acidified with 2 M aqueous HCl until pH is 1-2 at 0 °C and extracted with ethyl acetate, and the organic phase was dried with anhydrous Na_2SO_4 and concentrated to obtain the crude product. Then crude product was dissolved in methanol (20 mL). Concentrated sulfuric acid (1.07 mL, 2.0 mmol, 0.2 equiv) was added and the reaction mixture was stirred at 40 °C for 24 hours. Quenched with ice water (50 mL), extracted with diethyl ether (3 x 50 mL), dried over anhydrous Na_2SO_4 and removed of the solvent under reduced pressure, the crude mixture was purified by flashcolumn chromatography using PE/EtOAc (5:1, v/v) to afford **63a** as a colorless oil (1.82 g, 7.3 mmol, 73%).

R*f* = 0.30 (PE/EA = 5:1, v/v).

NMR Spectroscopy:

^{1}H NMR (400 MHz, Chloroform-*d*, 298 K, δ): 7.90 - 7.83 (m, 2H), 7.78 - 7.72 (m, 2H), 5.02 (t, *J* = 5.2 Hz, 1H), 4.23 - 4.16 (m, 2H), 3.77 (s, 3H). ppm.

^{13}C NMR (101 MHz, Chloroform-*d*, 298 K, δ): 168.5, 168.1, 134.5, 131.7, 123.8, 61.0, 54.7, 52.9 ppm.

Tetrabutylammonium methanol sulfate (D’) [4]

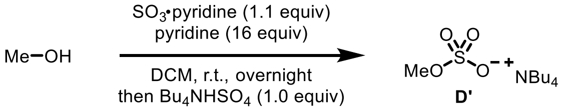

Tetrabutylammonium methanol sulfate (**D’**) was prepared according to previous report. [4] Methanol (0.80 g, 25 mmol, 1.0 equiv) and SO_3•pyridine complex (4.39 g, 27.5 mmol, 1.1 equiv) were placed in a 250 mL round bottom flask. Pyridine (33 mL) and dry dichloromethane (30 mL) were added and the mixture was stirred at rt overnight. Water (100 mL) was added and the mixture was washed once with dichloromethane (3 x 50 mL). The aqueous phase was treated with Bu_4NHSO_4 (8.45 g, 25 mmol, 1.0 equiv) and stirred for 1 h. The solution was extracted with dichloromethane (3 x 50 mL). The organic layer was dried over Na_2SO_4, filtered, and concentrated, resulting in a clear oil. After overnight drying under high vacuum D′ was isolated as a white solid (4.86 g, 13.8 mmol, 55%).

R*f* = 0.35 (DCM/MeOH = 15:1, v/v).

NMR Spectroscopy:

^{1}H NMR (400 MHz, DMSO-d_6, 298 K, δ): 3.37 (s, 3H), 3.20 – 3.14 (m, 8H), 1.61 – 1.53 (m, 8H), 1.31 (q, J = 7.3 Hz, 8H), 0.93 (t, J = 7.3 Hz, 12H) ppm.

^{13}C NMR (101 MHz, DMSO-d_6, 298 K, δ): 58.0, 53.3, 23.6, 19.7, 14.0 ppm.

4-biphenylmethanol-^{18}O (^{18}O-70a) [5]

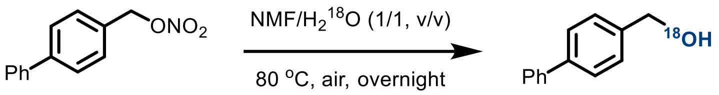

70s 78% yield **^{18}O-70a**, 58% rich

To a 4.0 mL borosilicate vial equipped with a stir bar was added 4-biphenylmethanol nitrate (0.2 mmol, 1.0 equiv), then solvent (NMF/H$_2$^{18}O = 1:1, v/v, c = 0.25 M) was added. After stirring for 12 h at 80 °C, the reaction was cooled down to room temperature, and concentrated by rotary evaporation. The residue was purified by chromatography on silica gel eluting to get **^{18}O-70a** as a white solid (29.1 mg, 0.156 mmol, 78%).

R$_f$ = 0.55 (PE/EA = 5:1, v/v).

NMR Spectroscopy:

^{1}H NMR (400 MHz, Chloroform-d, 298 K, δ): 7.60 (d, J = 7.5 Hz, 4H), 7.48 - 7.42 (m, 4H), 7.35 (t, J = 7.4 Hz, 1H), 4.75 (s, 2H) ppm.

^{13}C NMR (101 MHz, Chloroform-d, 298 K, δ): 141.0, 140.8, 140.0, 128.9, 127.6, 127.49, 127.47 127.2, 65.29, 65.27 ppm.

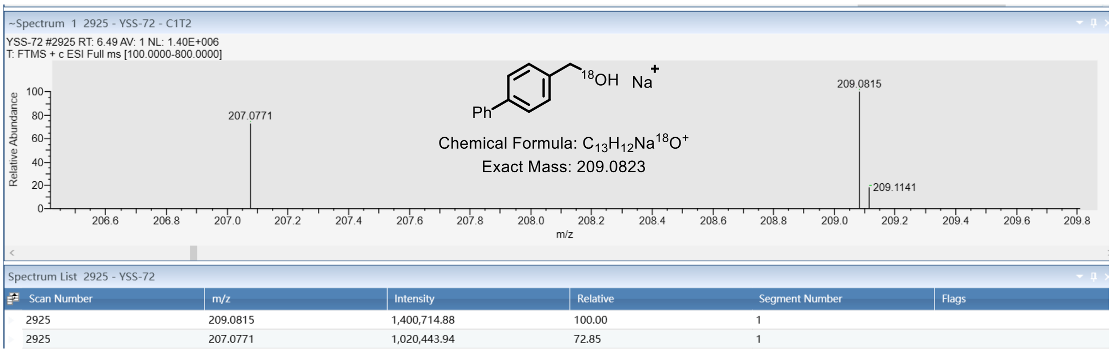

Supplementary Figure 13. HRMS of **^{18}O-70a**.

5. Gram scale synthesis and isolation of methy sulfate monoester 1-B

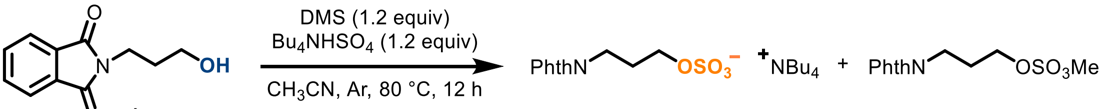

1a **1**, 79%, 4.15 g **1-B**, 8%, 0.23 g

To a 100 mL round bottom flask equipped with a stir bar was added 3-phthalimido-1-propanol (**1a**, 2.05 g, 10 mmol, 1.0 equiv), dimethyl sulfate (1.51 g, 12 mmol, 1.2 equiv) and tetrabutylammonium hydrogen sulfate (4.07 g, 12 mmol, 1.2 equiv). The flask was evacuated and backfilled with argon for three times, then CH_3CN (50.0 mL, c = 0.2 M) was added. After stirring for 12 h at 80 °C, the solvent was concentrated by rotary evaporation and the residue was purified by flash chromatography on silica gel eluting with PE/EA (5:1, v/v) to afford 3-phthalimido-1-propanol methyl sulfate (**1-B**, 0.23 g, 0.768 mmol, 8%), then eluting with DCM/MeOH (30:1, v/v) to afford the product tetrabutylammonium 3-phthalimido-1-propanol sulfate (**1**, 4.15 g, 7.88 mmol, 79%).

3-phthalimido-1-propanol methyl sulfate (**1-B**):

R_f = 0.45 (PE/EA = 3:1, v/v).

NMR Spectroscopy:

^{1}H NMR (400 MHz, Chloroform-*d*, 298 K, δ): 7.88 - 7.82 (m, 2H), 7.75 - 7.70 (m, 2H), 4.32 (t, *J* = 6.1 Hz, 2H), 3.99 (s, 3H), 3.84 (t, *J* = 6.7 Hz, 2H), 2.17 (p, *J* = 6.5 Hz, 2H) ppm.

^{13}C NMR (101 MHz, Chloroform-*d*, 298 K, δ): 168.3, 134.3, 132.1, 123.5, 70.7, 58.9, 34.5, 28.2 ppm.

HRMS *m/z* (ESI+) calculated for $C_{12}H_{13}NO_6S^+$, 299.0458, found, 299.0448 $[M]^+$.

6. Mechanistic experiments

6.1 Reaction-time profiles studies on **1** monitored by ^{1}H NMR spectra

To a 4.0 mL borosilicate vial equipped with a stir bar was added 3-phthalimido-1-propanol **1a** (41.0 mg, 0.2 mmol, 1.0 equiv), dimethyl sulfate (DMS, 30.3 mg, 0.24 mmol, 1.2 equiv) and tetrabutylammonium hydrogen sulfate (81.5 mg, 0.24 mmol, 1.2 equiv). The vial was evacuated and backfilled with argon for three times, then CH_3CN (1.0 mL, c = 0.2 M) was added. The reaction mixture was then stirred at 80 °C. After the indicated time, a stock solution (0.1 ml) of the reaction mixture in $CDCl_3$ (0.3 ml) was monitored by ^{1}H NMR spectra. We obserbed the full reaction–time profiles of the reaction from the spectrum, and validates the formation of intermediates **1-B** and **D'**(**D**), as well as their preliminary reactivitity in the reaction system.

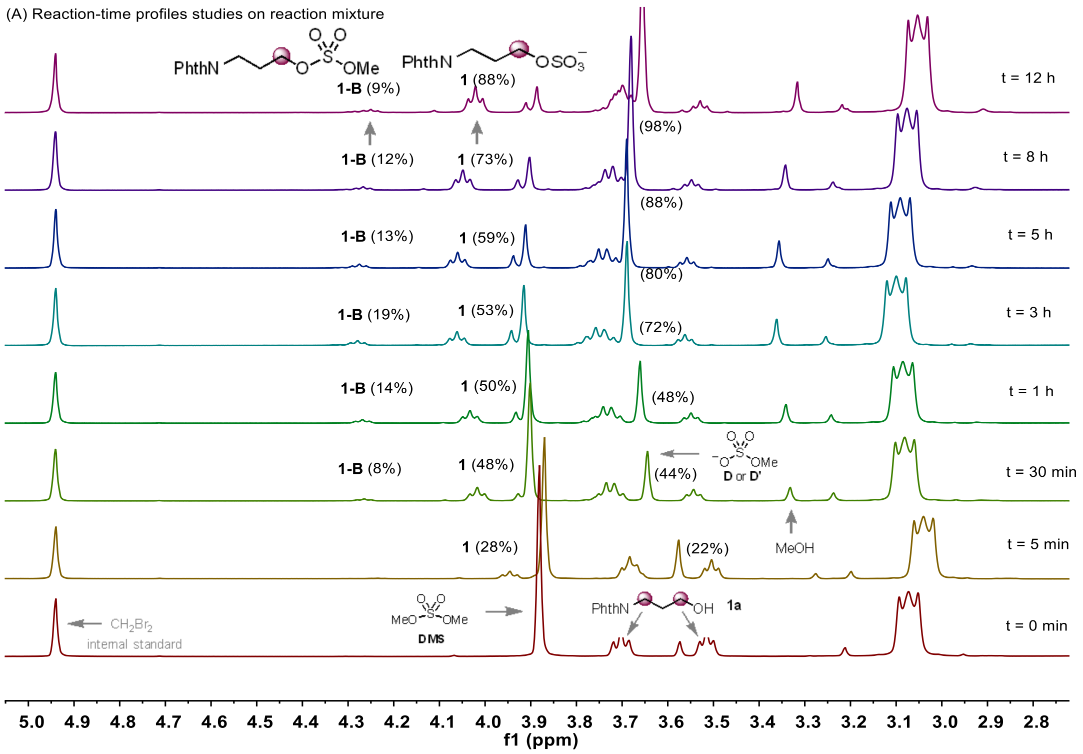

Supplementary Figure 14. Reaction-time profiles studies on reaction mixture.

6.3 Formation of **D** and **D'**

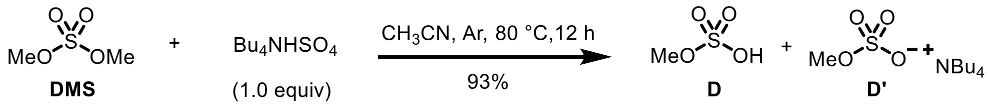

To a 4.0 mL borosilicate vial equipped with a stir bar was added dimethyl sulfate (DMS, 25.2 mg, 0.2 mmol, 1.0 equiv) and tetrabutylammonium hydrogen sulfate (67.9 mg, 0.2 mmol, 1.0 equiv). The vial was evacuated and backfilled with argon for three times, then CH_3CN (1.0 mL, c = 0.2 M) was added. After stirring for 12 h at 80 °C, dibromomethane (14.0 μL, 0.2 mmol, 1.0 equiv) was added as an internal standard. The reaction mixture was diluted with $CDCl_3$, and the yield of tetrabutylammonium sulfates was determined by ^{1}H NMR integration relative to the internal standard (standard: δ 4.94 ppm, **D** and **D'**: δ 3.72 (s) ppm, DMS: 3.96 (s) ppm).

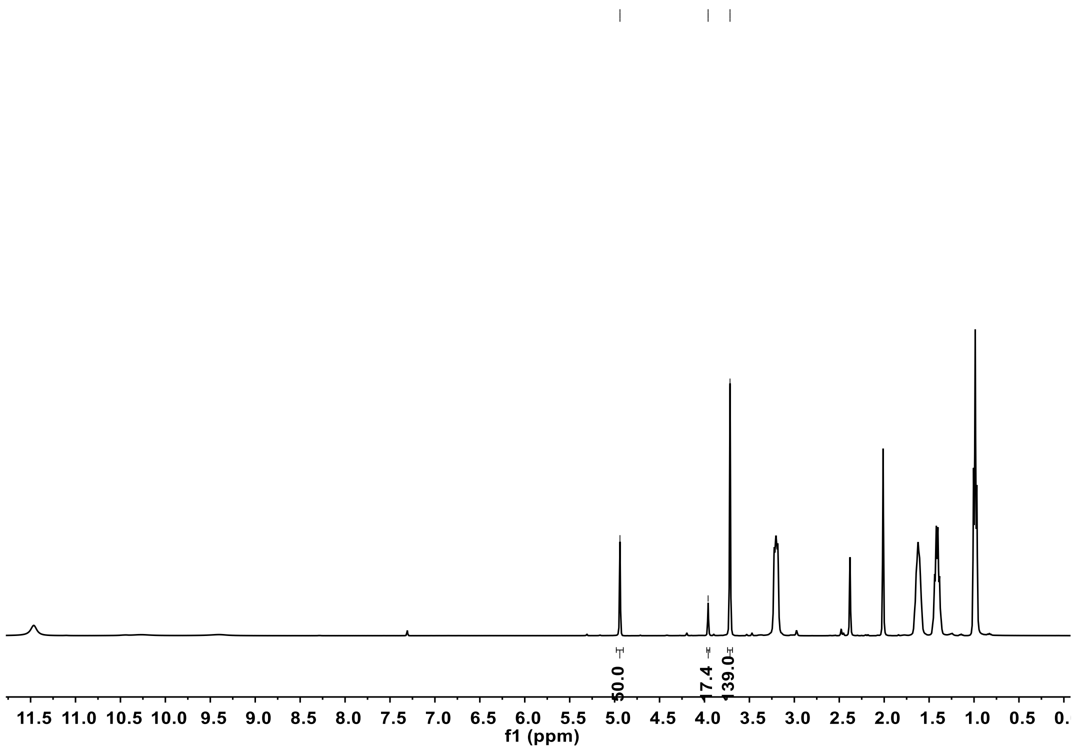

Supplementary Figure 15. NMR yield of formation for **D** and **D'** mixture.

LC-MS of the above mixture: 110.9757 was HRMS of the methyl sulfate ($MeOSO_3^-$), no information was found for $Bu_3N•SO_3$ (exact mass: 265.1712).

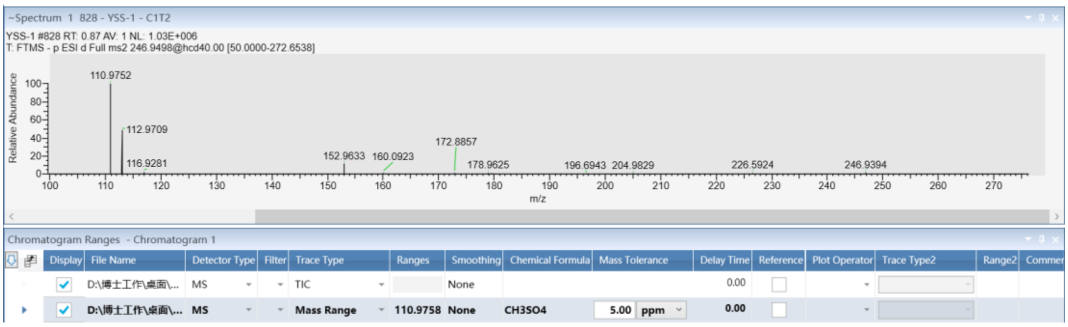

Supplementary Figure 16. LC-MS of reaction mixture.

GC-MS of the above mixture: no mass signal was found for SO_3 (m/z = 79.9568).

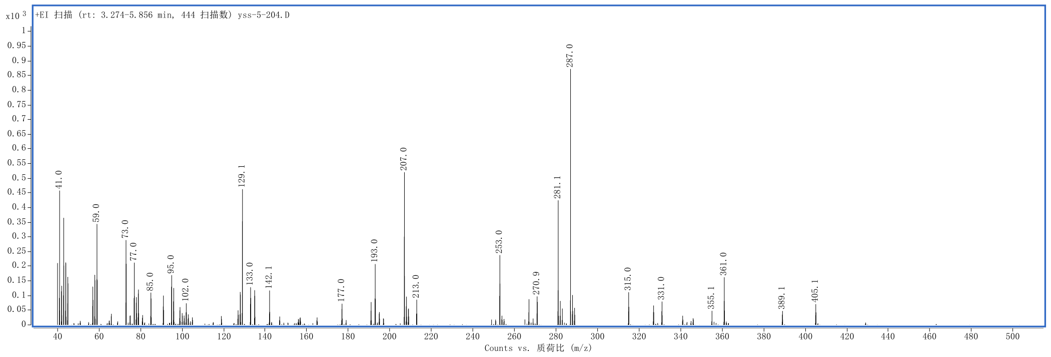

Supplementary Figure 17. GC-MS of reaction mixture.

6.4 Stoichiometric *O*-sulfation

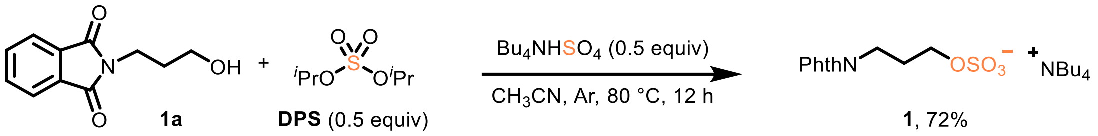

The reaction was carried out according to general procedure **B** with modified conditons: 0.5 equiv DPS and 0.5 equiv Bu_4NHSO_4 were used. After stirring for 12 h at 80 °C, dibromomethane (14.0 μL, 0.2 mmol, 1.0 equiv) was added as an internal standard. The reaction mixture was diluted with $CDCl_3$, and the yield of tetrabutylammonium sulfates was determined by ^1H NMR integration relative to the internal standard (standard: δ 4.94 ppm, **1**: δ 4.08 (t, *J* = 6.3Hz) ppm).

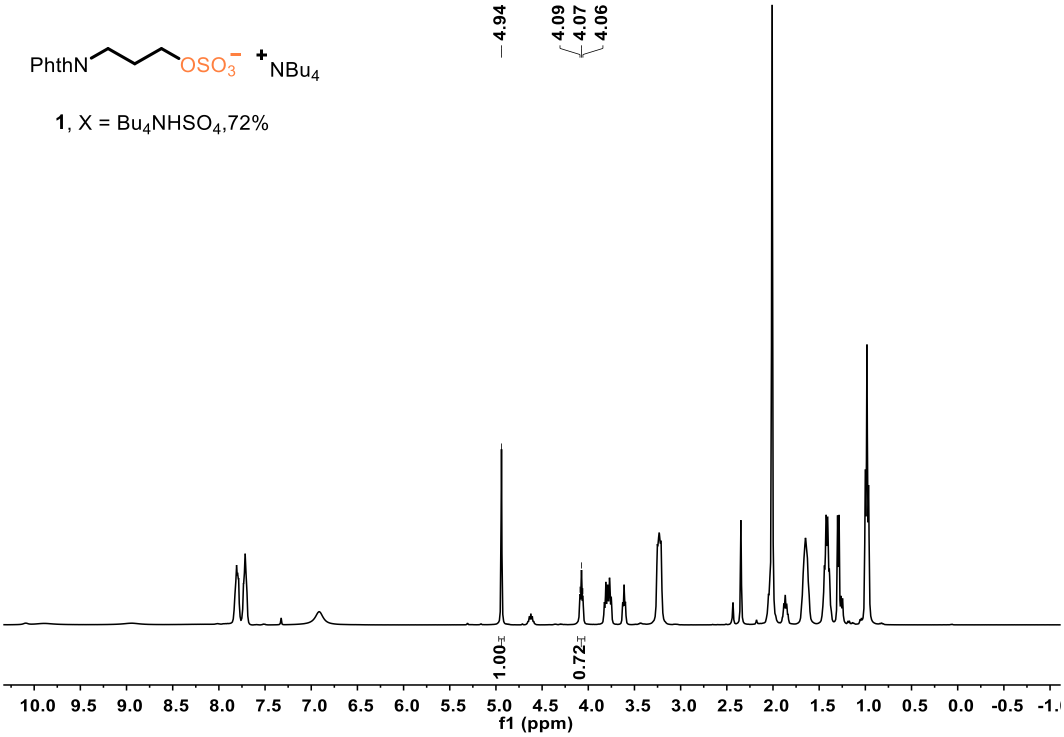

Supplementary Figure 18. NMR yield of stoichiometric O-sulfation with DPS.

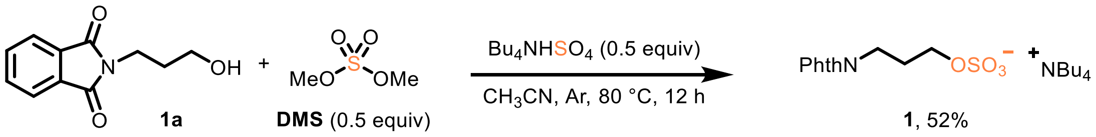

The reaction was carried out according to general procedure **A** with modified conditons: 0.5 equiv DMS and 0.5 equiv Bu_4NHSO_4 were used. After stirring for 12 h at 80 °C, dibromomethane (14.0 μL, 0.2 mmol, 1.0 equiv) was added as an internal standard. The reaction mixture was diluted with $CDCl_3$, and the yield of tetrabutylammonium

sulfates was determined by ^{1}H NMR integration relative to the internal standard (standard: δ 4.94 ppm, **1**: δ 4.08 (t, J = 6.3Hz) ppm).

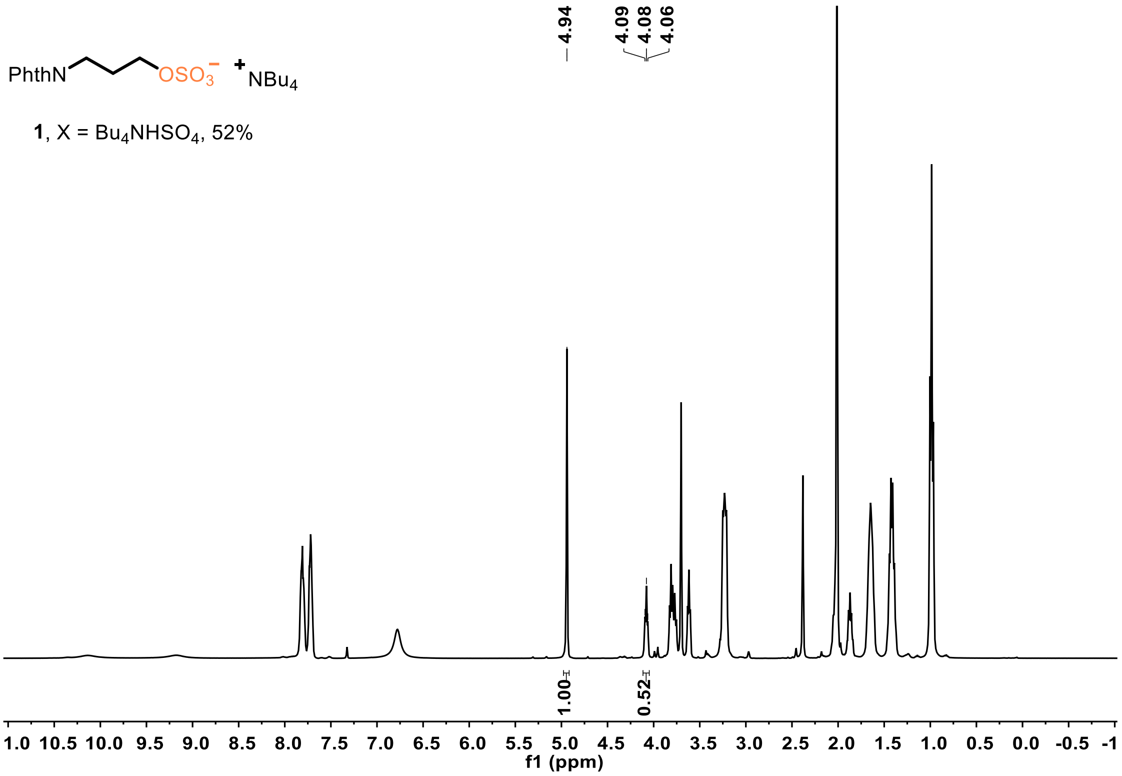

Supplementary Figure 19. NMR yield of stoichiometric O-sulfation with DMS.

6.5 Reactivity of **1-B**

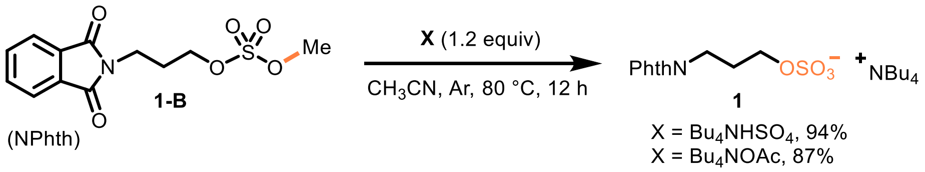

To a 4.0 mL borosilicate vial equipped with a stir bar was added 3-phthalimido-1-propanol methyl sulfate **1-B** (59.8 mg, 0.2 mmol, 1.0 equiv) and tetrabutyl ammonium salt (Bu_4NHSO_4 or Bu_4NOAc, 0.24 mmol, 1.2 equiv). The vial was evacuated and backfilled with argon for three times, then CH_3CN (1.0 mL, c = 0.2 M) was added. After stirring for 12 h at 80 °C, dibromomethane (14.0 μL, 0.2 mmol, 1.0 equiv) was added as an internal standard. The reaction mixture was diluted with $CDCl_3$, and the yield of tetrabutylammonium sulfates was determined by ^{1}H NMR integration relative to the internal standard (standard: δ 4.94 ppm, **1**: δ 4.08 (t, J = 6.3Hz) ppm).

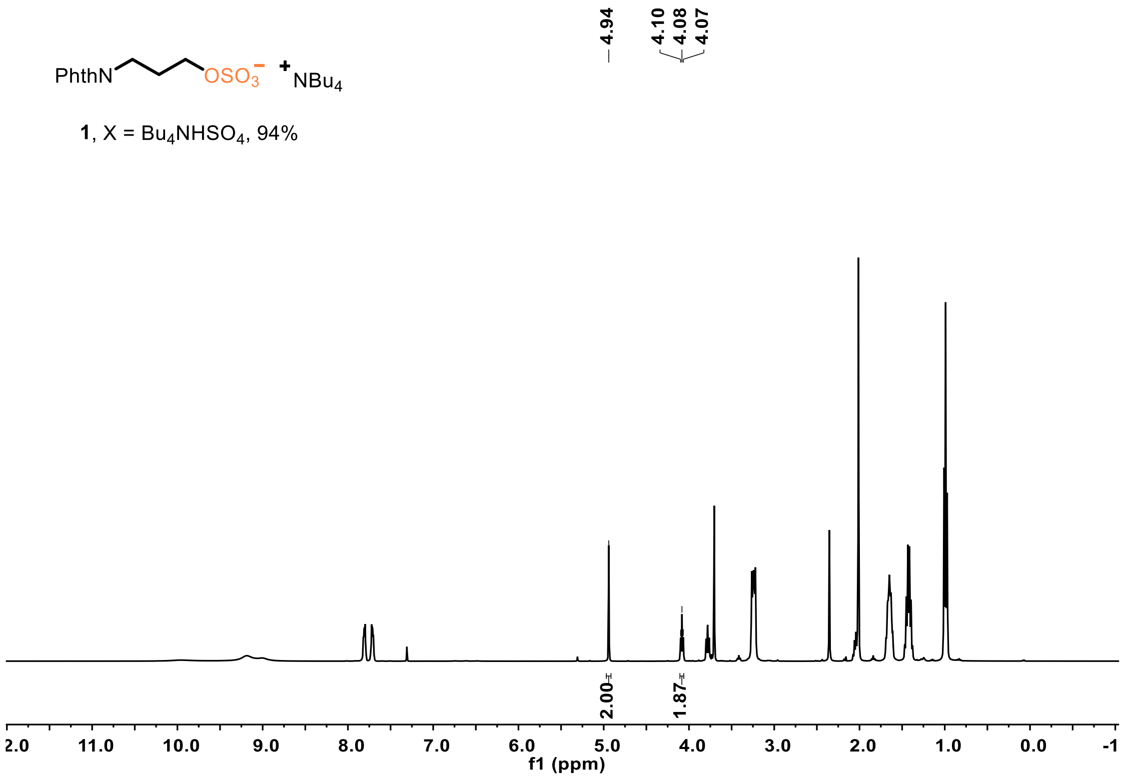

Supplementary Figure 20. NMR yield of O-sulfation for **1-B** with Bu_4NHSO_4.

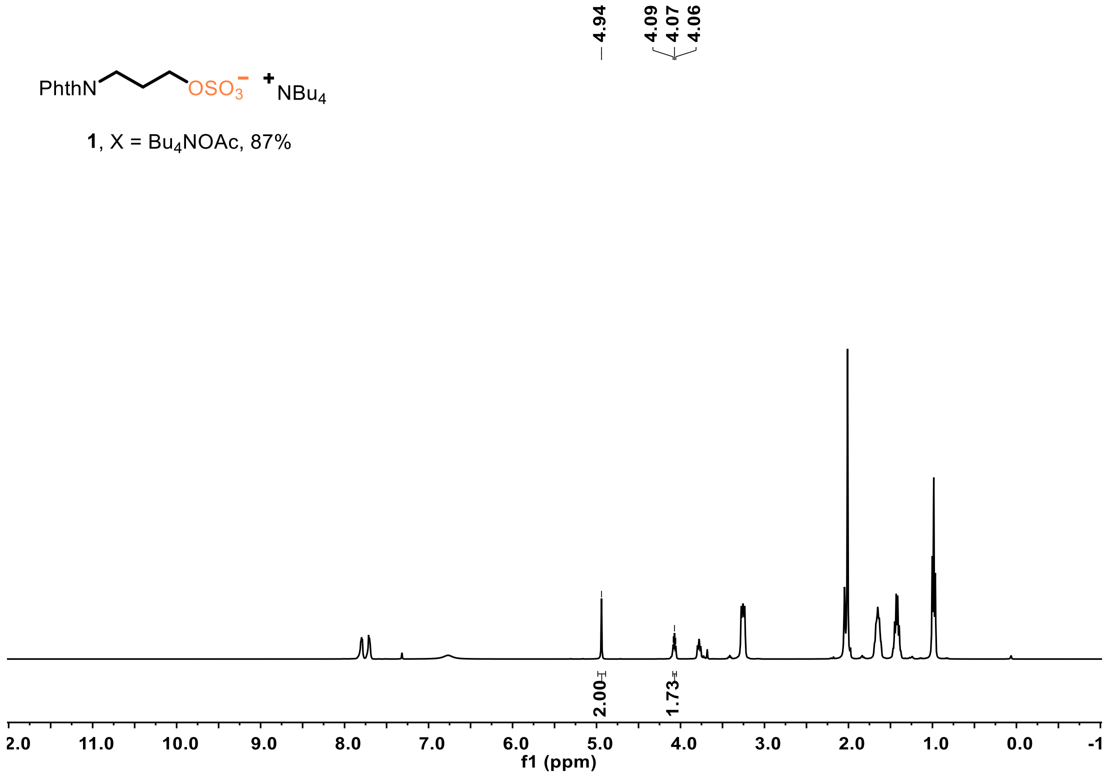

Supplementary Figure 21. NMR yield of O-sulfation for **1-B** with Bu_4NOAc.

6.6 Reactivity of **D'**

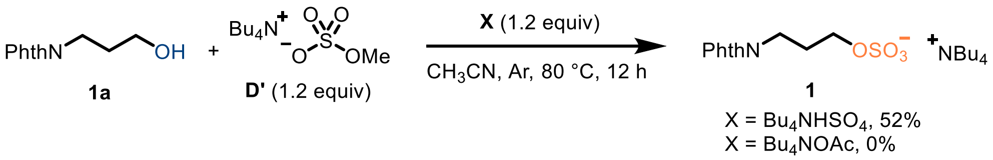

To a 4.0 mL borosilicate vial equipped with a stir bar was added 3-phthalimido-1-propanol **1a** (41.0 mg, 0.2 mmol, 1.0 equiv), tetrabutylammonium methanol sulfate **D'** (84.8 mg, 0.24 mmol, 1.2 equiv) and tetrabutyl ammonium salt (Bu_4NHSO_4 or Bu_4NOAc, 0.24 mmol, 1.2 equiv). The vial was evacuated and backfilled with argon for three times, then CH_3CN (1.0 mL, c = 0.2 M) was added. After stirring for 12 h at 80 °C, dibromomethane (14.0 μL, 0.2 mmol, 1.0 equiv) was added as an internal standard. The reaction mixture was diluted with $CDCl_3$, and the yield of tetrabutylammonium sulfates was determined by ^{1}H NMR integration relative to the internal standard(standard: δ 4.94 ppm, **1**: δ 4.08 (t, J = 6.3Hz) ppm).

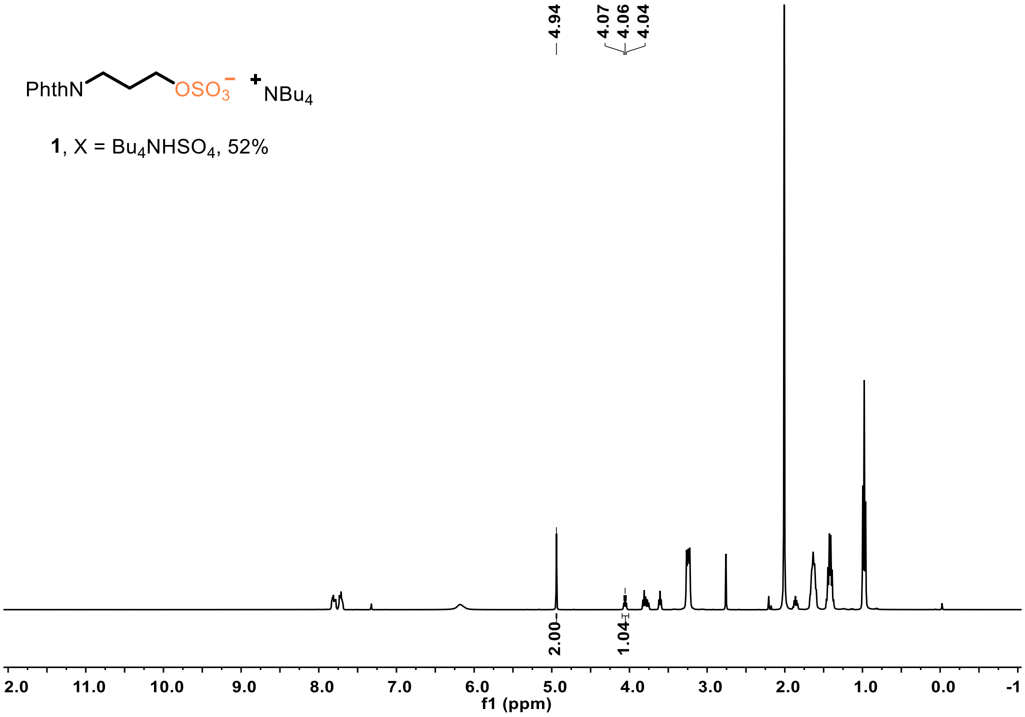

Supplementary Figure 22. NMR yield of O-sulfation for **D'** with Bu_4NHSO_4.

Additional data for reactivity of D' and its variants:

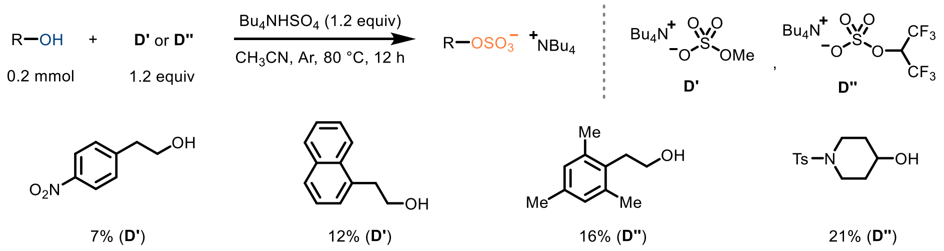

Supplementary Figure 23. Reactivity of **D'** and its variants with other substrate.

6.7 ¹⁸O-labeling experiments

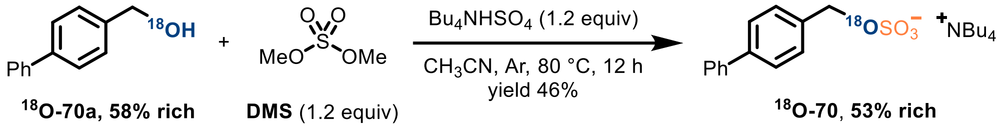

The reaction was carried out according to general procedure **A**. The yield of tetrabutylammonium 4-biphenylmethanol-^{18}O sulfate **^{18}O-70** was purified by plate chromatography on silica gel eluting with DCM/MeOH (30:1, v/v) to afford the product as a light yellow oil (46.7 mg, 0.092 mmol, 46%).

Tetrabutylammonium 4-biphenylmethanol-^{18}O sulfate (**^{18}O-70**):

R_f = 0.40 (DCM/MeOH = 15:1, v/v).

NMR Spectroscopy:

^{1}H NMR (400 MHz, Chloroform-*d*, 298 K, δ): 7.53 (dd, *J* = 12.9, 7.7 Hz, 4H), 7.45 (d, *J* = 7.9 Hz, 2H), 7.40 (t, *J* = 7.5 Hz, 2H), 7.30 (t, *J* = 7.4 Hz, 1H), 5.07 (s, 2H), 3.22 - 3.16 (m, 8H), 1.59 - 1.52 (m, 8H), 1.40 - 1.34 (m, 8H), 0.93 (t, *J* = 7.3 Hz, 12H) ppm.

^{13}C NMR (101 MHz, Chloroform-*d*, 298 K, δ): 140.9, 140.3, 136.9, 128.8, 128.6, 127.3, 127.0, 126.8, 68.62, 68.59, 58.6, 23.9, 19.7, 13.7 ppm.

HRMS *m/z* (ESI-) calculated for $C_{13}H_{11}O_3{}^{18}OS^-$, 265.0426, found, 265.0422 [M-NBu$_4$]$^-$.

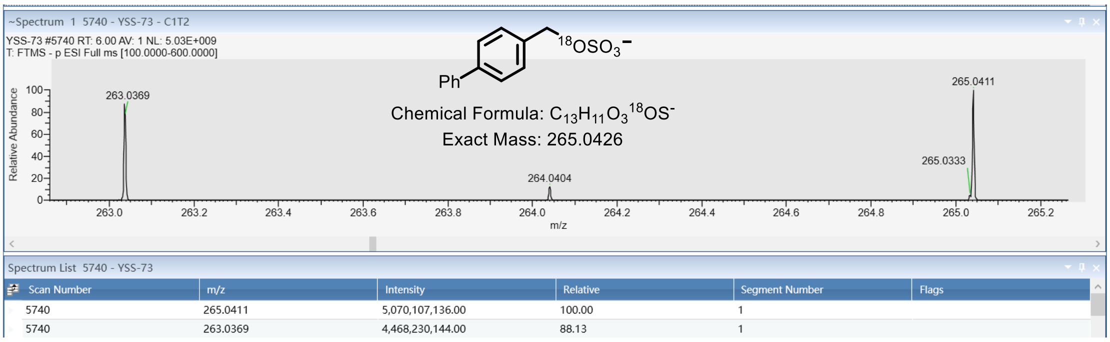

Supplementary Figure 24. HRMS of **^{18}O-70**.

7. X-ray crystallographic data

Tetrabutylammonium 2,6-dichlorophenethanol sulfate 8 (CCDC 2290725)

Tetrabutylammonium 2,6-dichlorophenethanol sulfate **8** was crystallized from MeOH at room temperature. Refer to the synthesis section of **8** for the details of the crystallization conditions. The atoms are depicted with 50% probability ellipsoids. The crystallographic data are summarized in the following table.

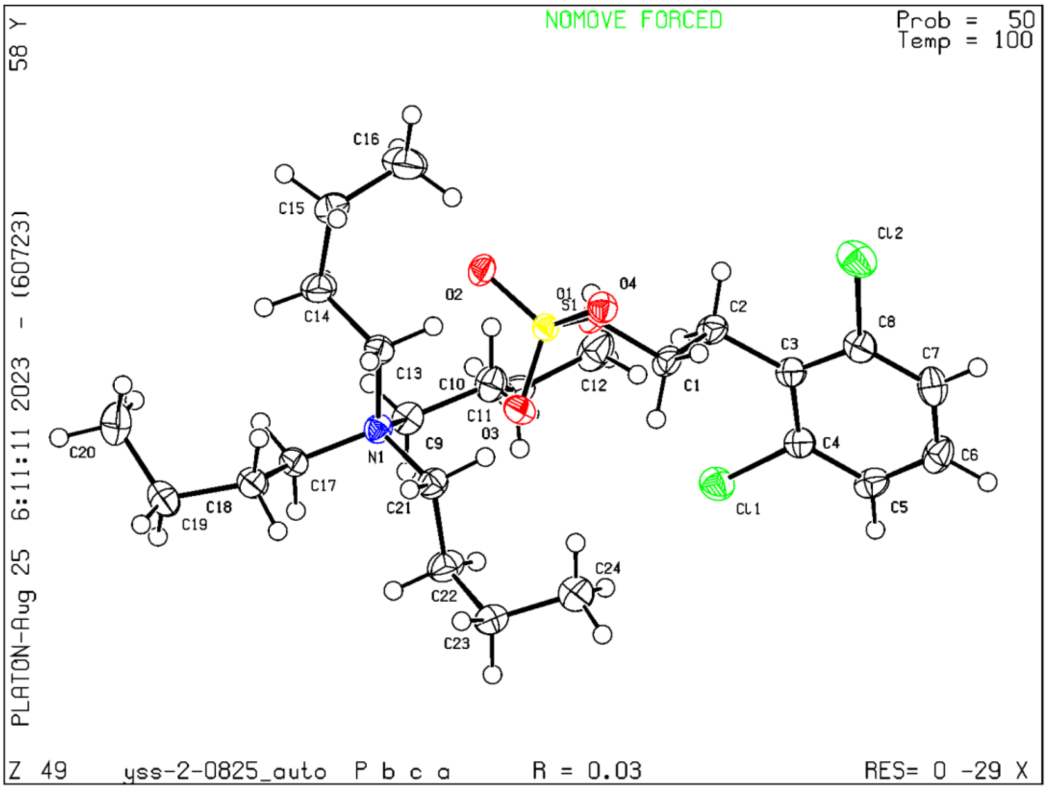

Supplementary Figure 25. X-ray structure of **8**.

Supplementary Table 6. Crystal data and structure refinement.

Empirical formula	$C_{24}H_{43}Cl_2NO_4S$
Formula weight	512.55
Temperature/K	100.01(10)
Crystal system	orthorhombic
Space group	Pbca
a/Å	15.85430(10)
b/Å	16.50680(10)
c/Å	20.7859(2)
α/°	90
β/°	90
γ/°	90
Volume/Å^3	5439.75(7)
Z	8
ρ_{calc}g/cm^3	1.252
μ/mm^{-1}	3.092
F(000)	2208.0
Crystal size/mm^3	0.15 × 0.13 × 0.12
Radiation	Cu Kα (λ = 1.54184)

2Θ range for data collection/°	8.508 to 154.96
Index ranges	$-13 \le h \le 19$, $-20 \le k \le 20$, $-24 \le l \le 26$
Reflections collected	32012
Independent reflections	5609 [R_{int} = 0.0302, R_{sigma} = 0.0218]
Data/restraints/parameters	5609/0/293
Goodness-of-fit on F^2	1.072
Final R indexes [I>=2σ (I)]	R_1 = 0.0321, wR_2 = 0.0831
Final R indexes [all data]	R_1 = 0.0347, wR_2 = 0.0850
Largest diff. peak/hole / e $Å^{-3}$	0.35/-0.39

8. Spectroscopic data

Tetrabutylammonium 3-phthalimido-1-propanol sulfate (1)

^{1}H NMR of tetrabutylammonium 3-phthalimido-1-propanol sulfate (1)

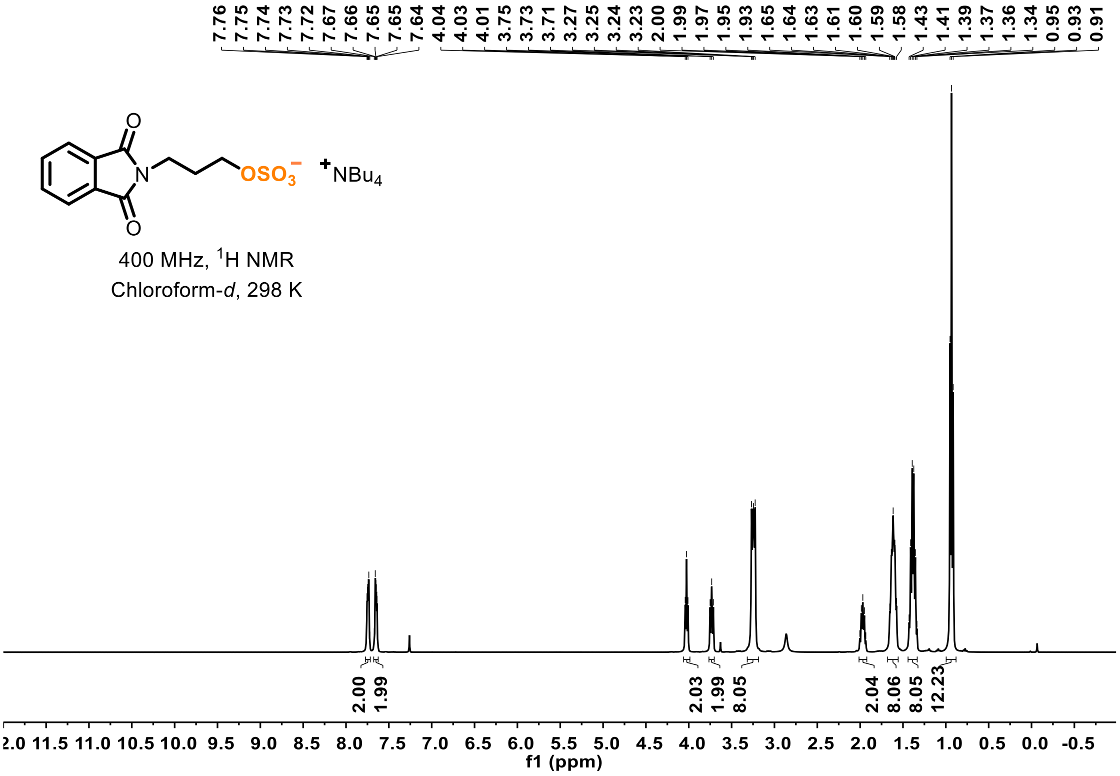

^{13}C NMR of tetrabutylammonium 3-phthalimido-1-propanol sulfate (1)

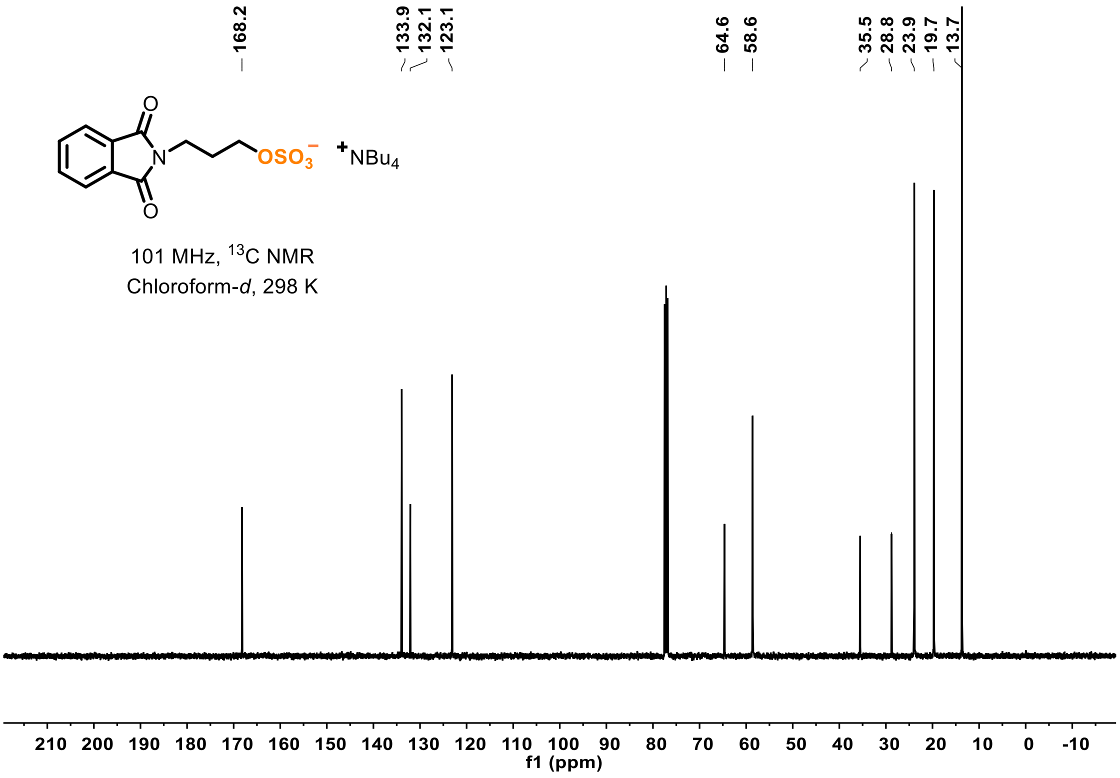

Tetrabutylammonium 3-bromophenylethanol sulfate (2)

^{1}H NMR of tetrabutylammonium 3-bromophenylethanol sulfate (2)

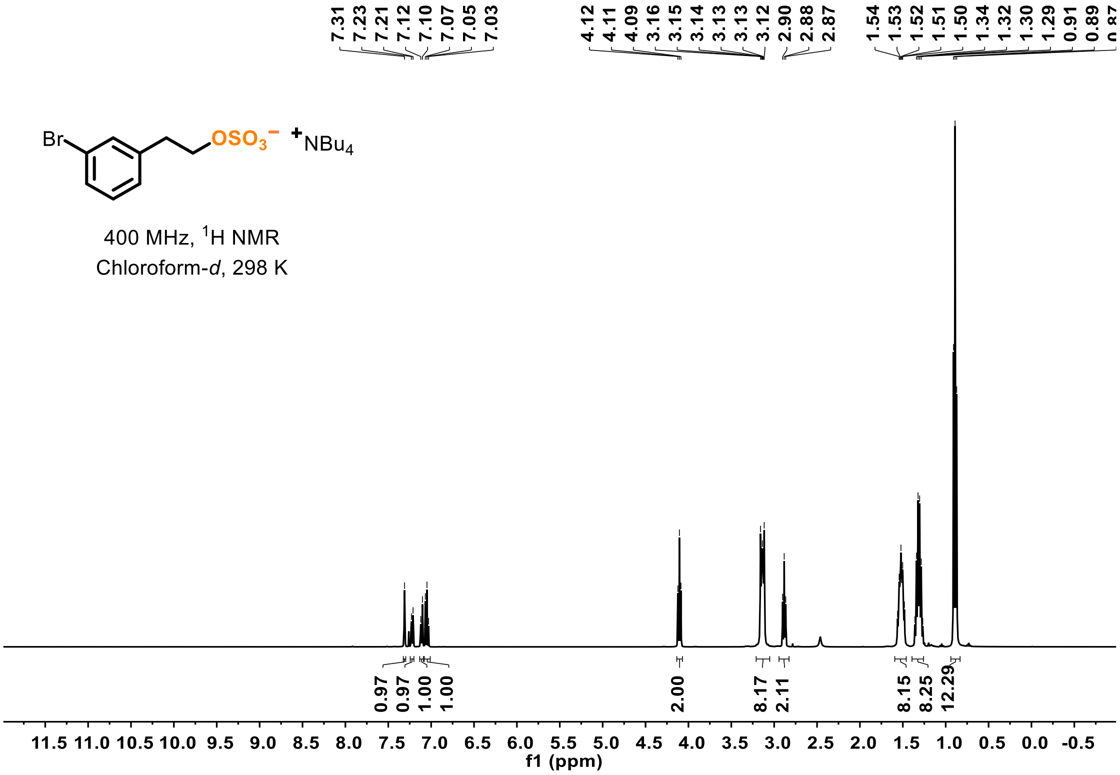

^{13}C NMR of tetrabutylammonium 3-bromophenylethanol sulfate (2)

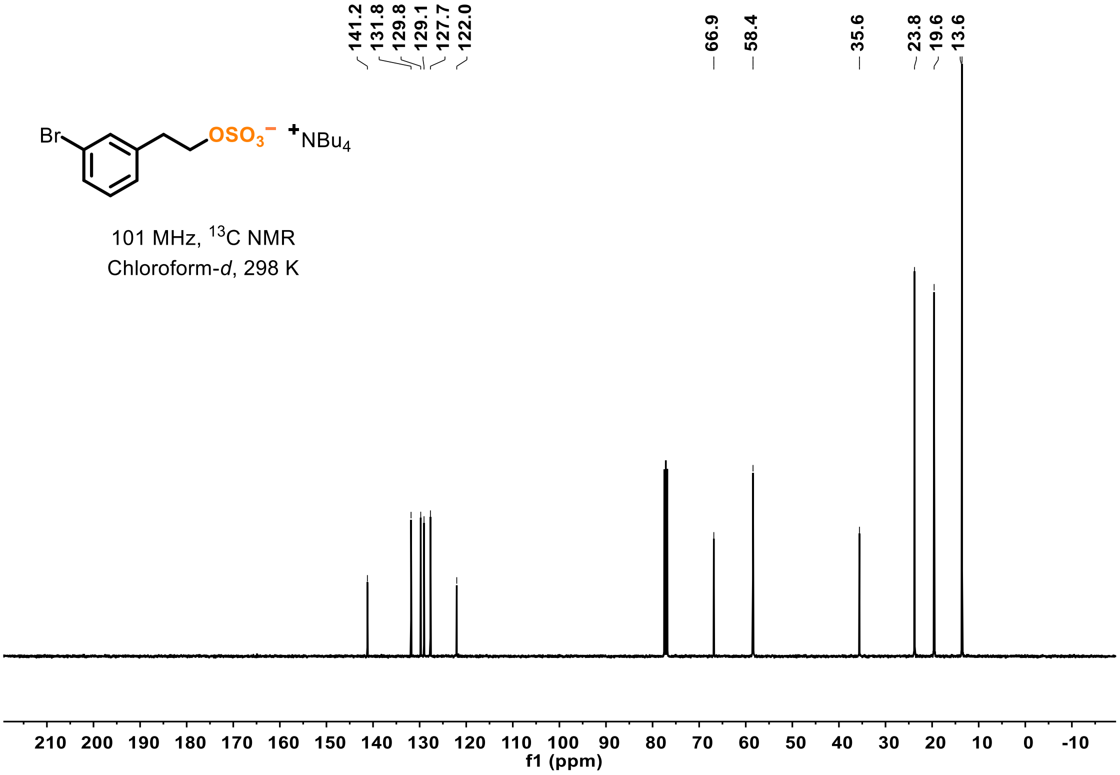

Tetrabutylammonium 4-fluorophenylethanol sulfate (3)

1H NMR of tetrabutylammonium 4-fluorophenylethanol sulfate (3)

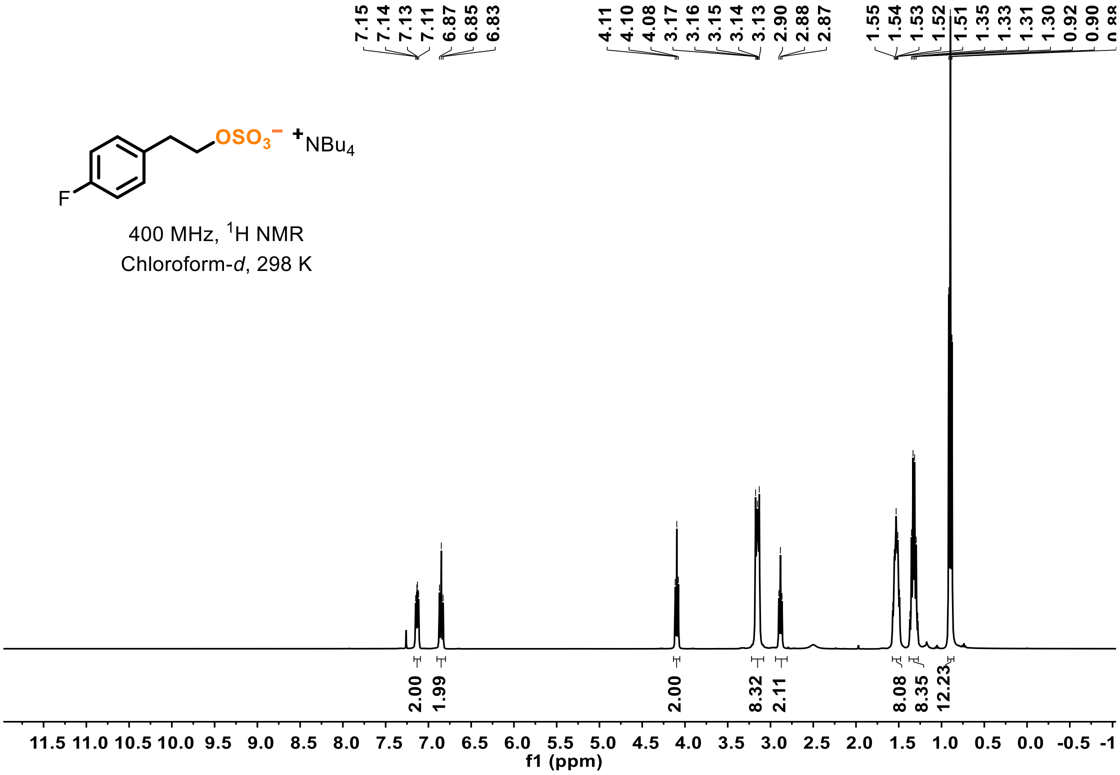

13C NMR of tetrabutylammonium 4-fluorophenylethanol sulfate (3)

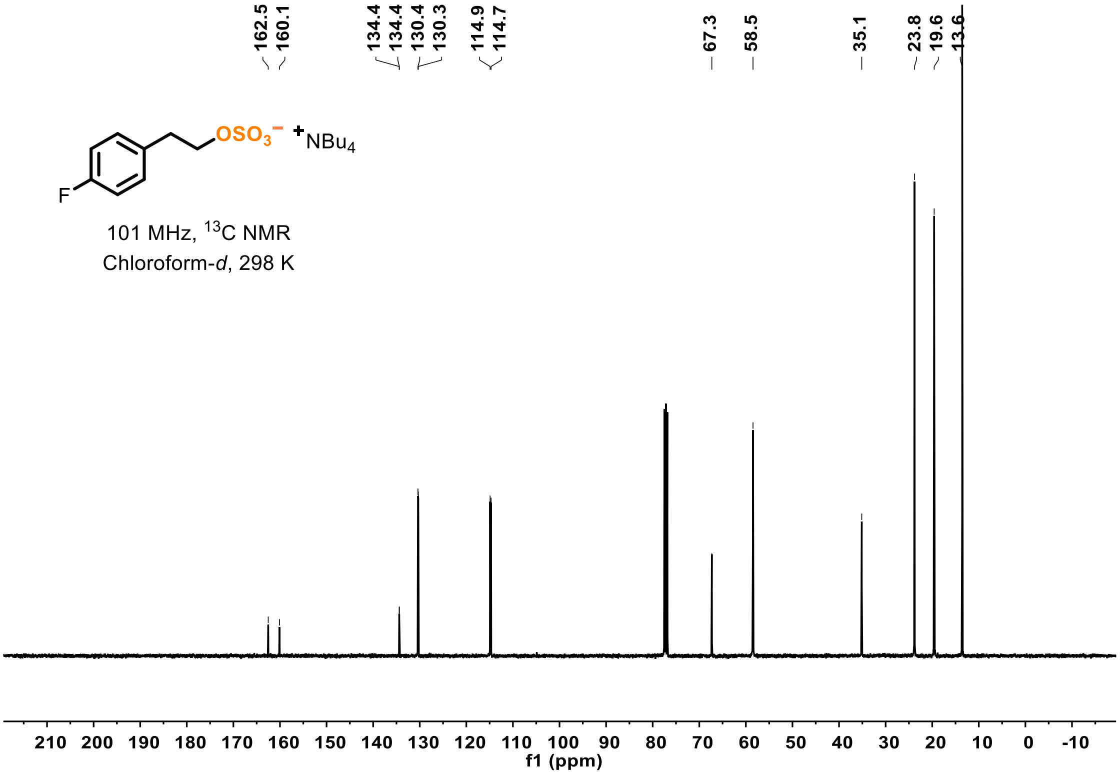

^{19}F NMR of tetrabutylammonium 4-fluorophenylethanol sulfate (3)

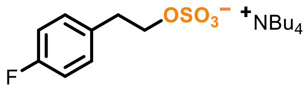

376 MHz, ^{19}F NMR
Chloroform-*d*, 298 K

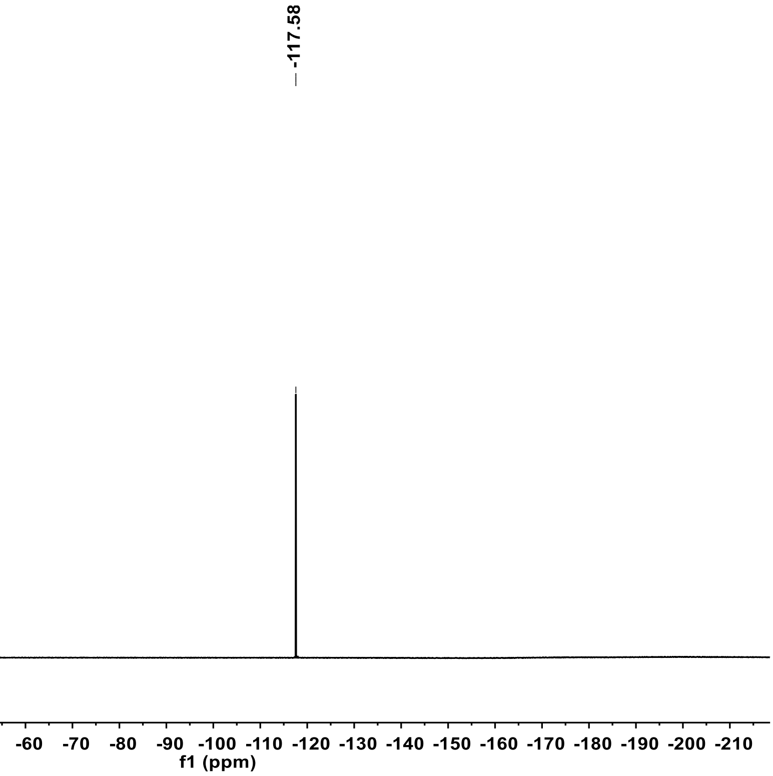

Tetrabutylammonium 4-nitrophenylethanol sulfate (4)

^{1}H NMR of tetrabutylammonium 4-nitrophenylethanol sulfate (4)

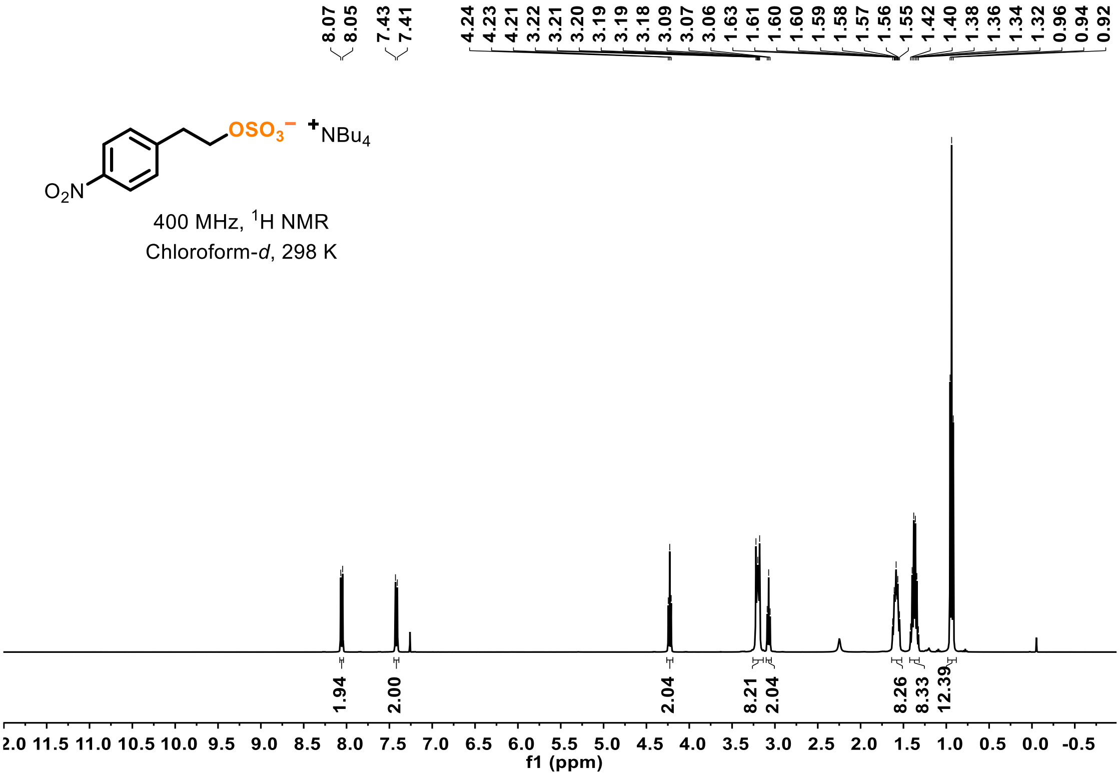

^{13}C NMR of tetrabutylammonium 4-nitrophenylethanol sulfate (4)

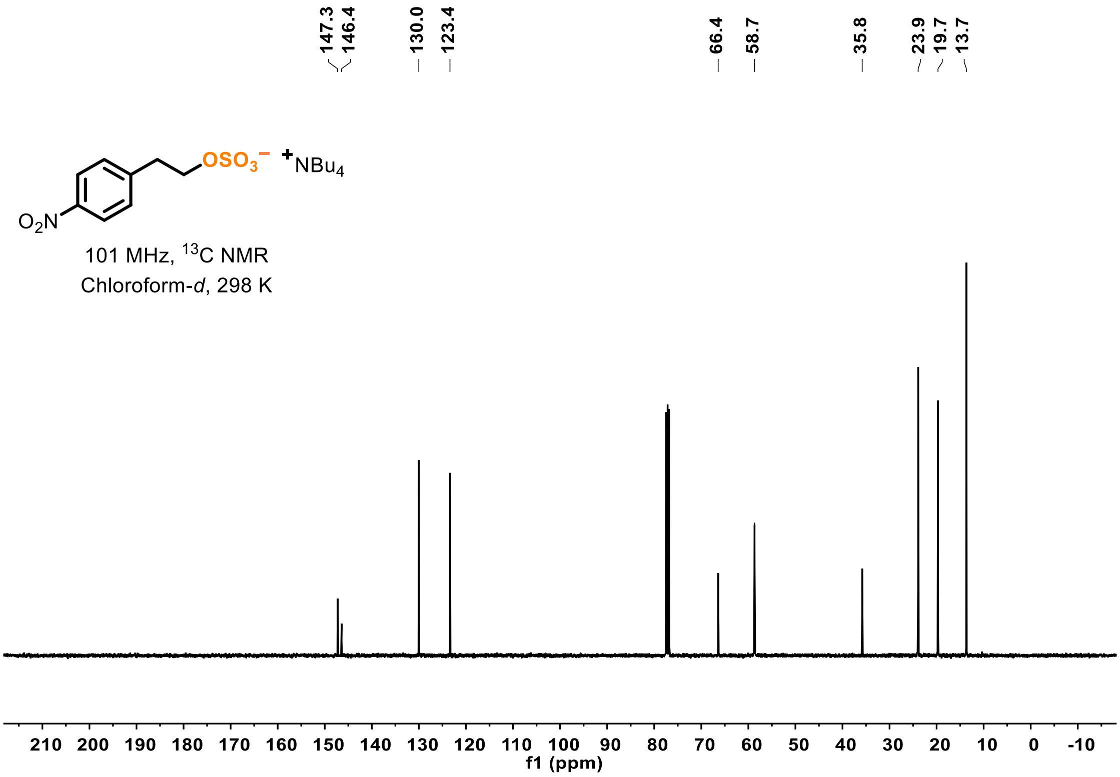

Tetrabutylammonium 4-cyanophenylethanol sulfate (5)

^{1}H NMR of tetrabutylammonium 4-cyanophenylethanol sulfate (5)

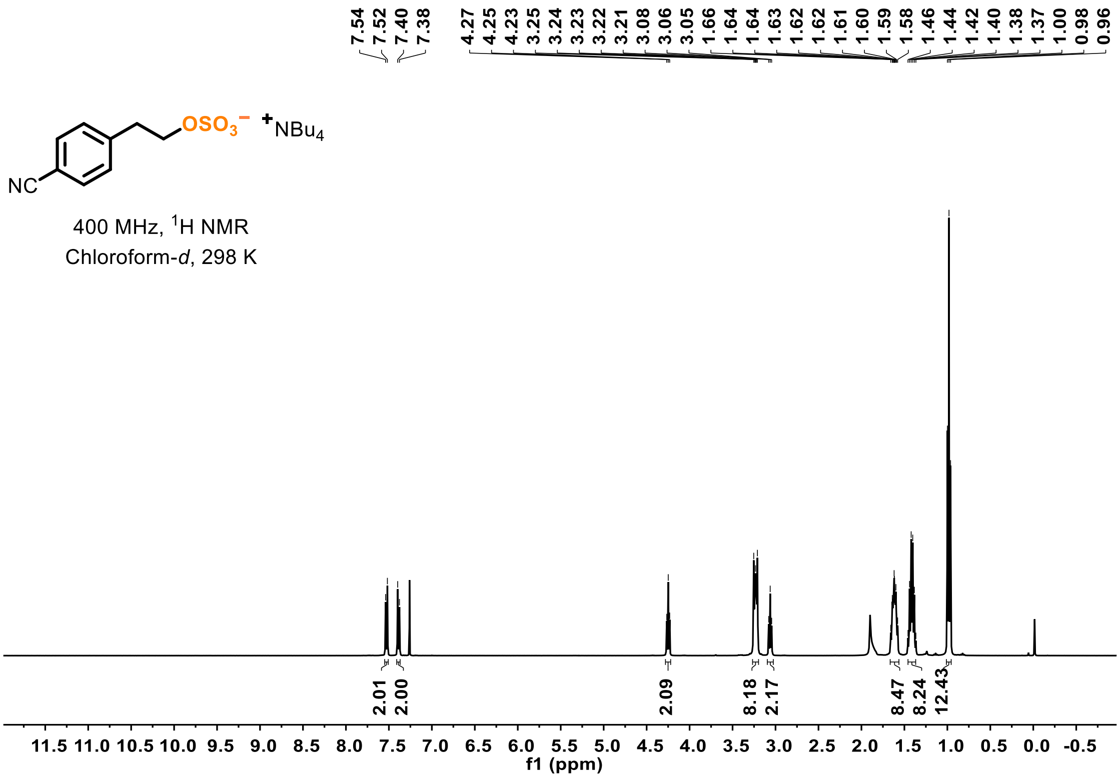

^{13}C NMR of tetrabutylammonium 4-cyanophenylethanol sulfate (5)

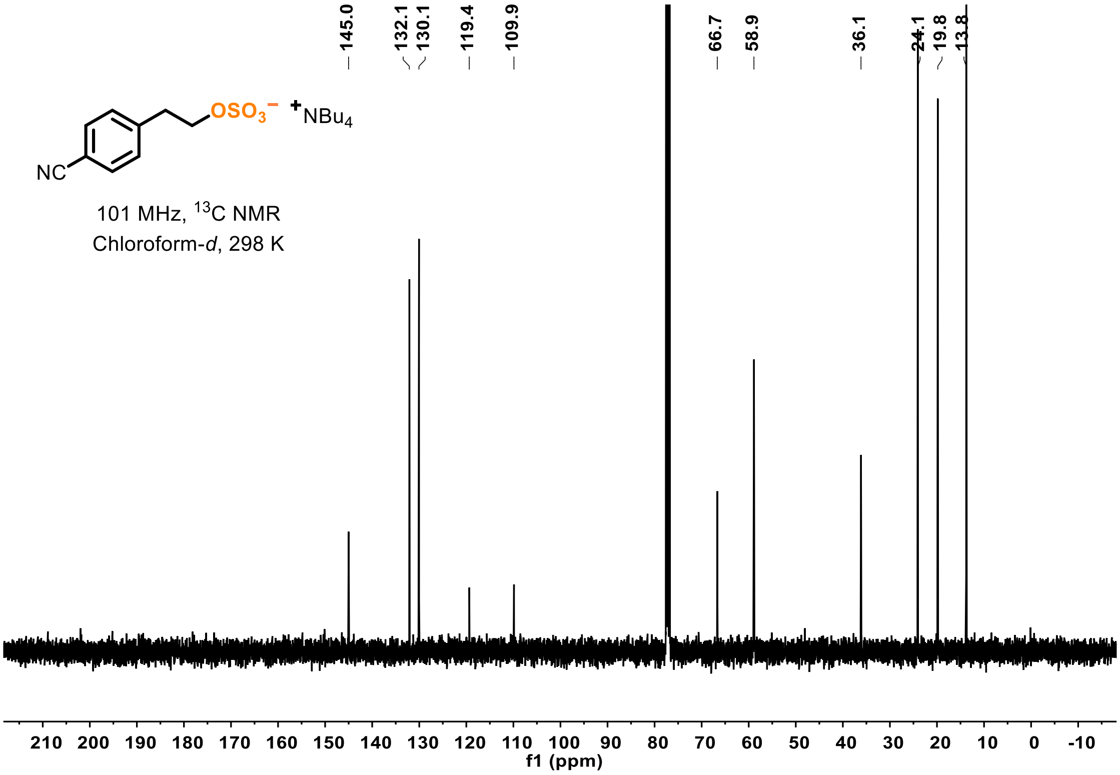

Tetrabutylammonium 4-tertbutylphenylethanol sulfate (6)

^{1}H NMR of tetrabutylammonium 4-tertbutylphenylethanol sulfate (6)

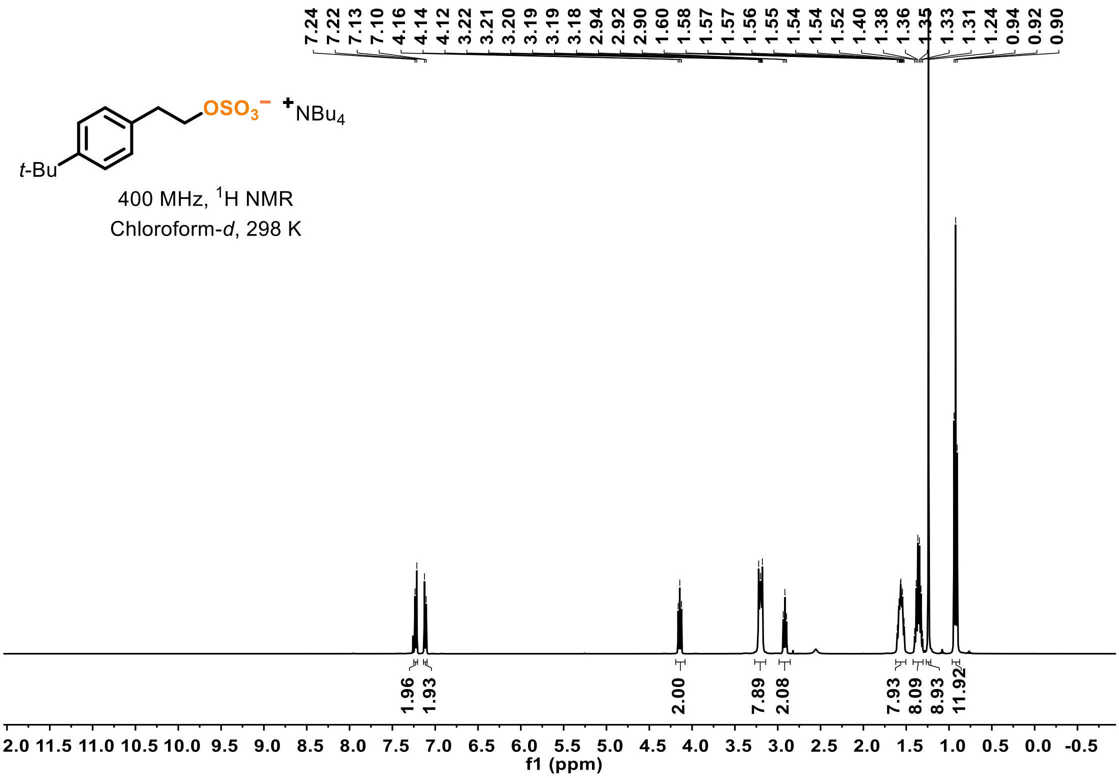

^{13}C NMR of tetrabutylammonium 4-tertbutylphenylethanol sulfate (6)

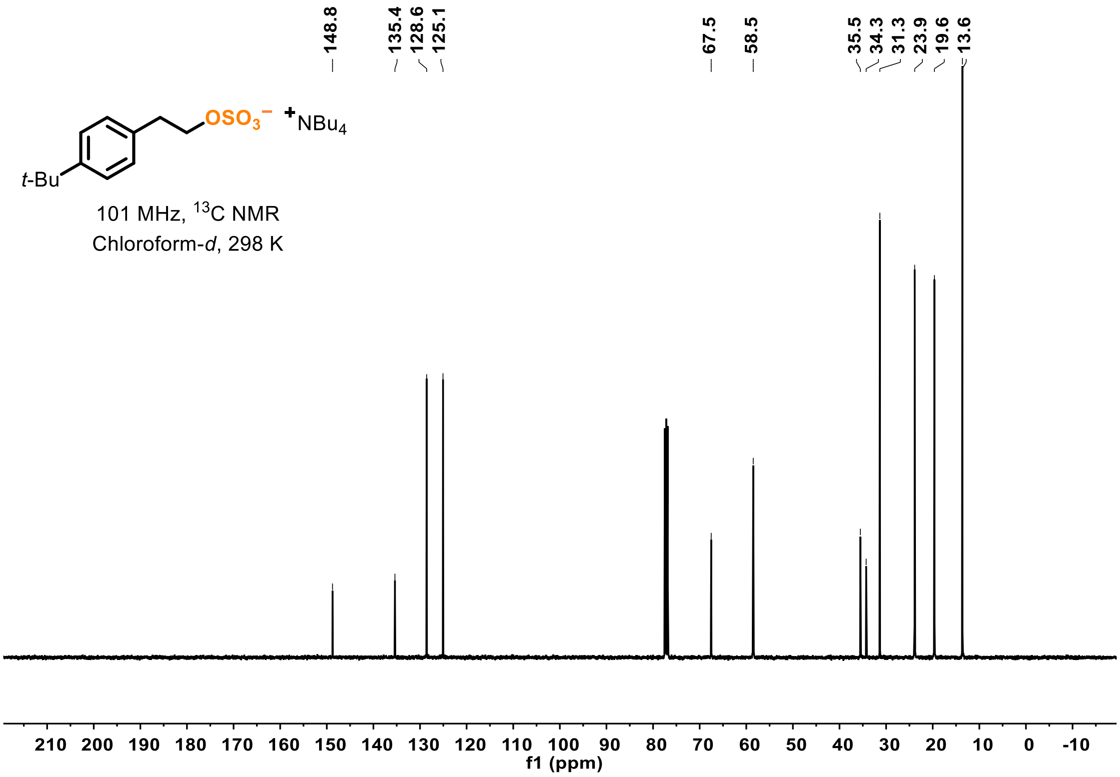

Tetrabutylammonium 4-methoxyphenylethanol sulfate (7)

^{1}H NMR of tetrabutylammonium 4-methoxyphenylethanol sulfate (7)

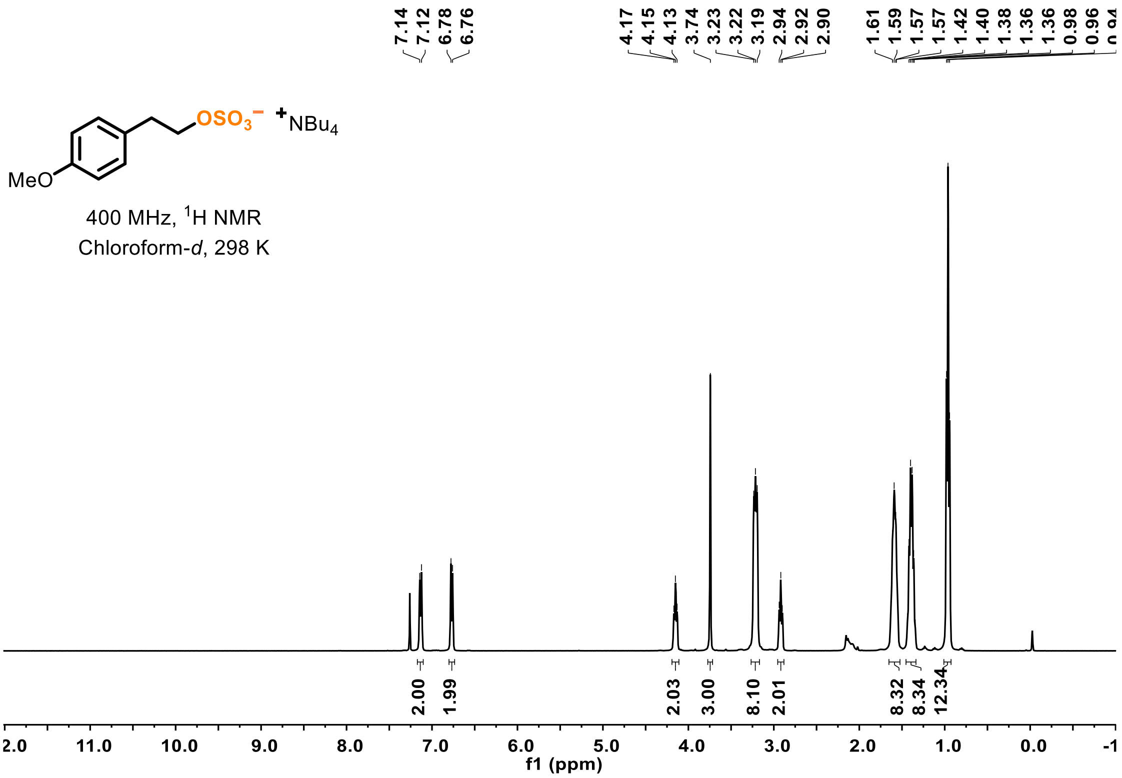

^{13}C NMR of tetrabutylammonium 4-methoxyphenylethanol sulfate (7)

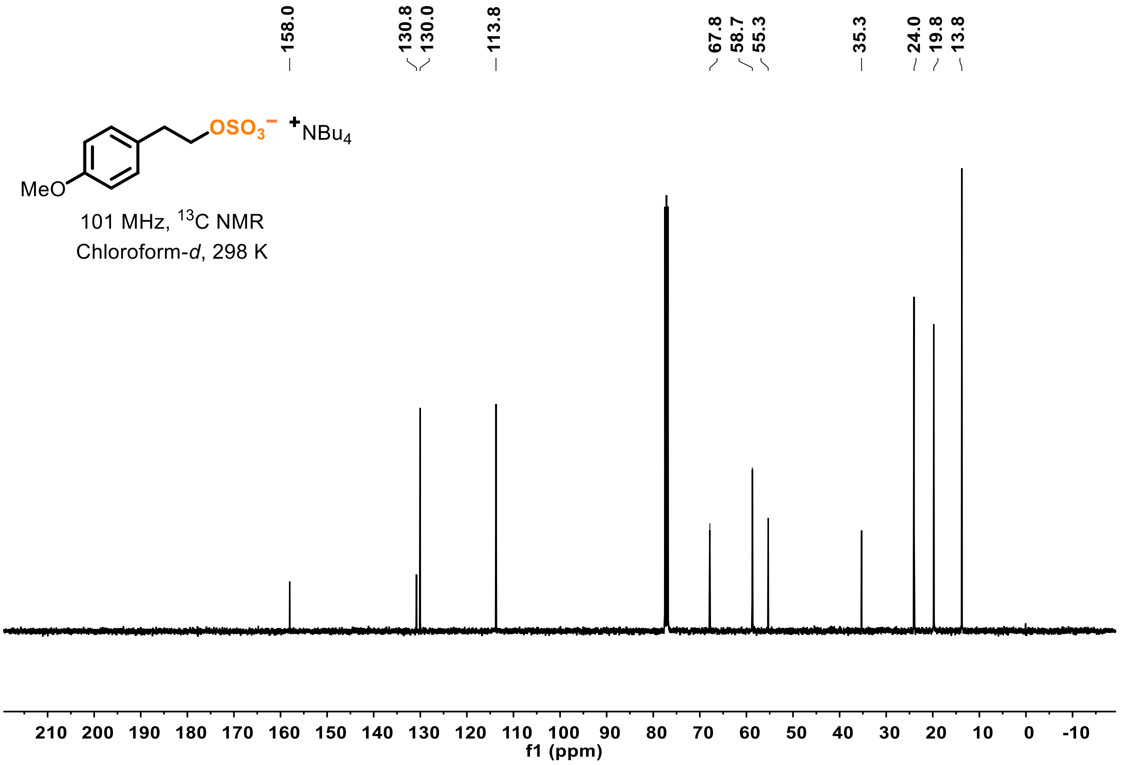

Tetrabutylammonium 2,6-dichlorophenethanol sulfate (8)

^{1}H NMR of tetrabutylammonium 2,6-dichlorophenethanol sulfate (8)

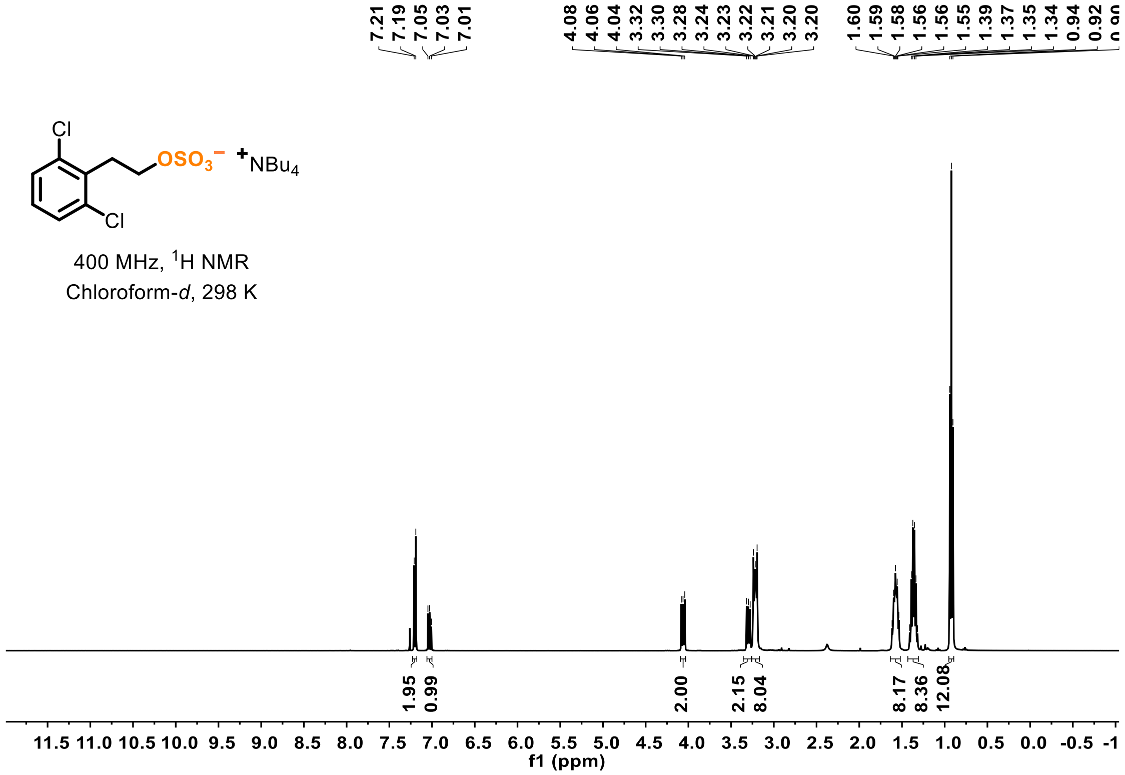

^{13}C NMR of tetrabutylammonium 2,6-dichlorophenethanol sulfate (8)

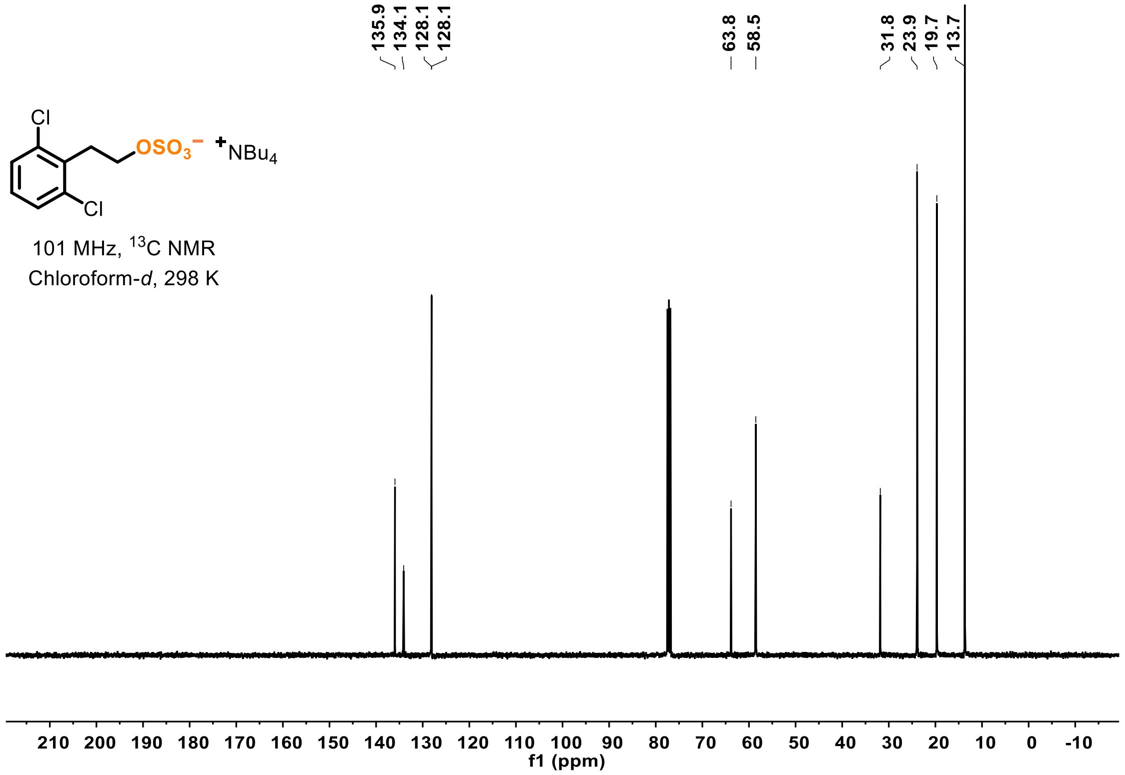

Tetrabutylammonium 1-naphthaleneethanol sulfate (9)

^{1}H NMR of tetrabutylammonium 1-naphthaleneethanol sulfate (9)

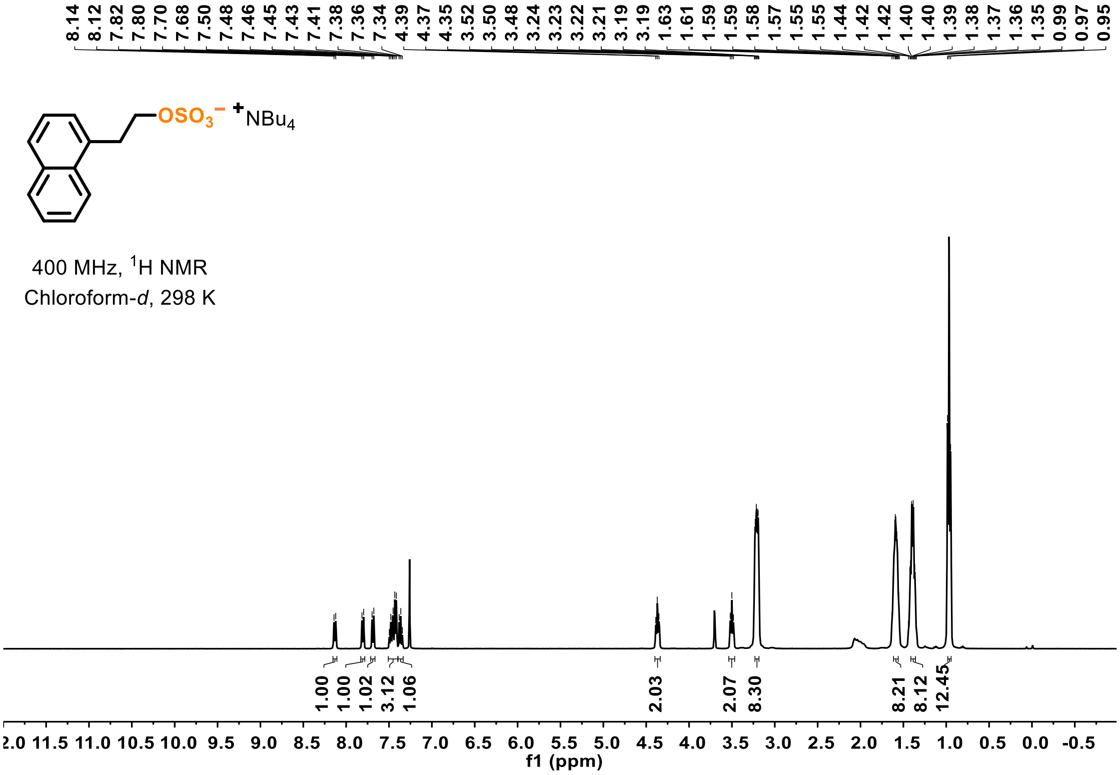

^{13}C NMR of tetrabutylammonium 1-naphthaleneethanol sulfate (9)

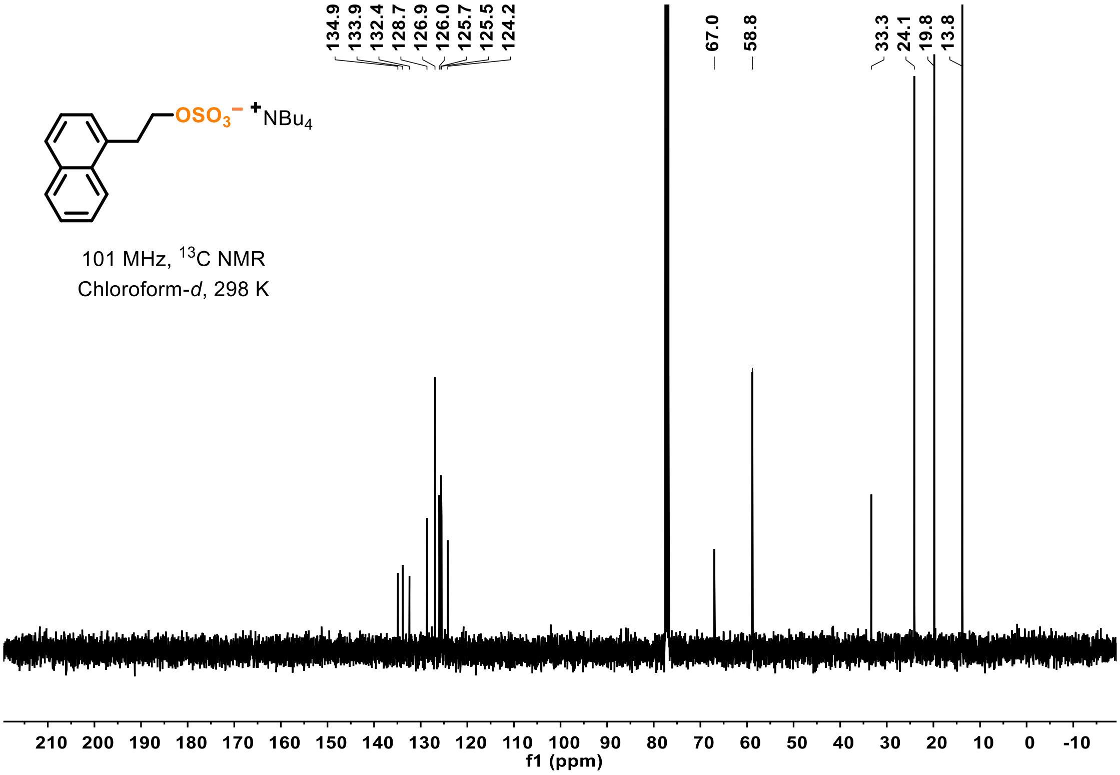

Tetrabutylammonium 2,4,6-trimethylphenylethanol sulfate (10)

^{1}H NMR of tetrabutylammonium 2,4,6-trimethylphenylethanol sulfate (10)

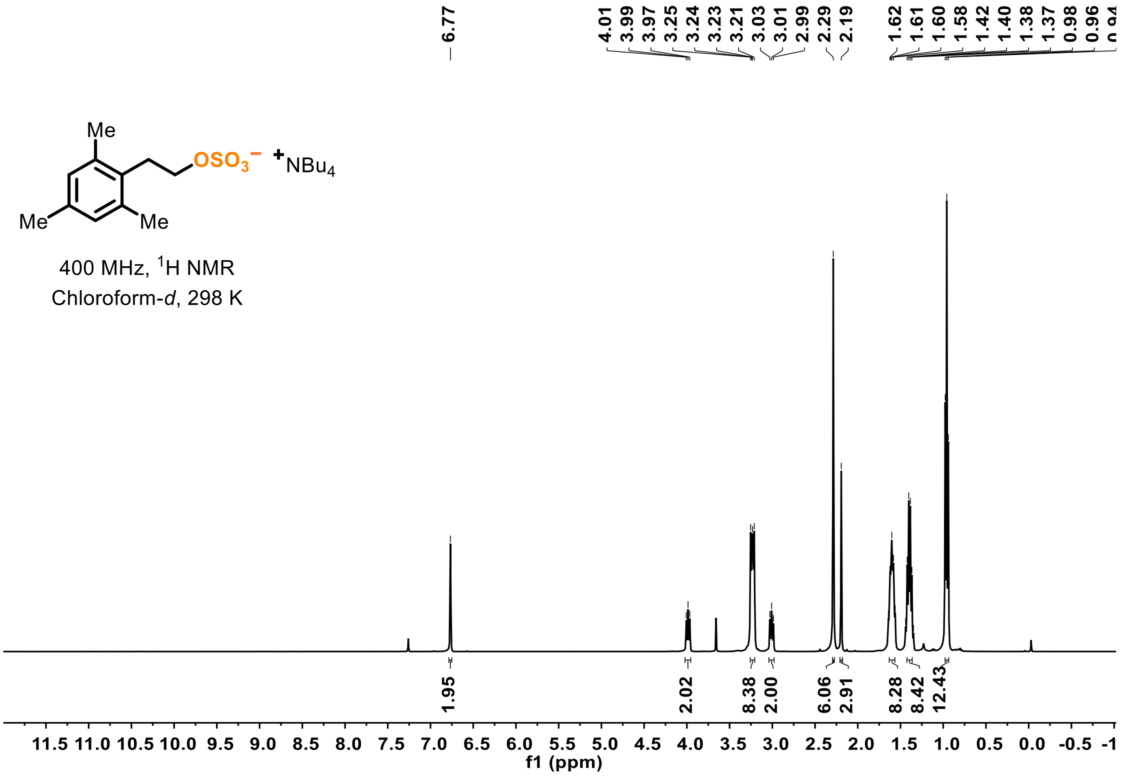

^{13}C NMR of tetrabutylammonium 2,4,6-trimethylphenylethanol sulfate (10)

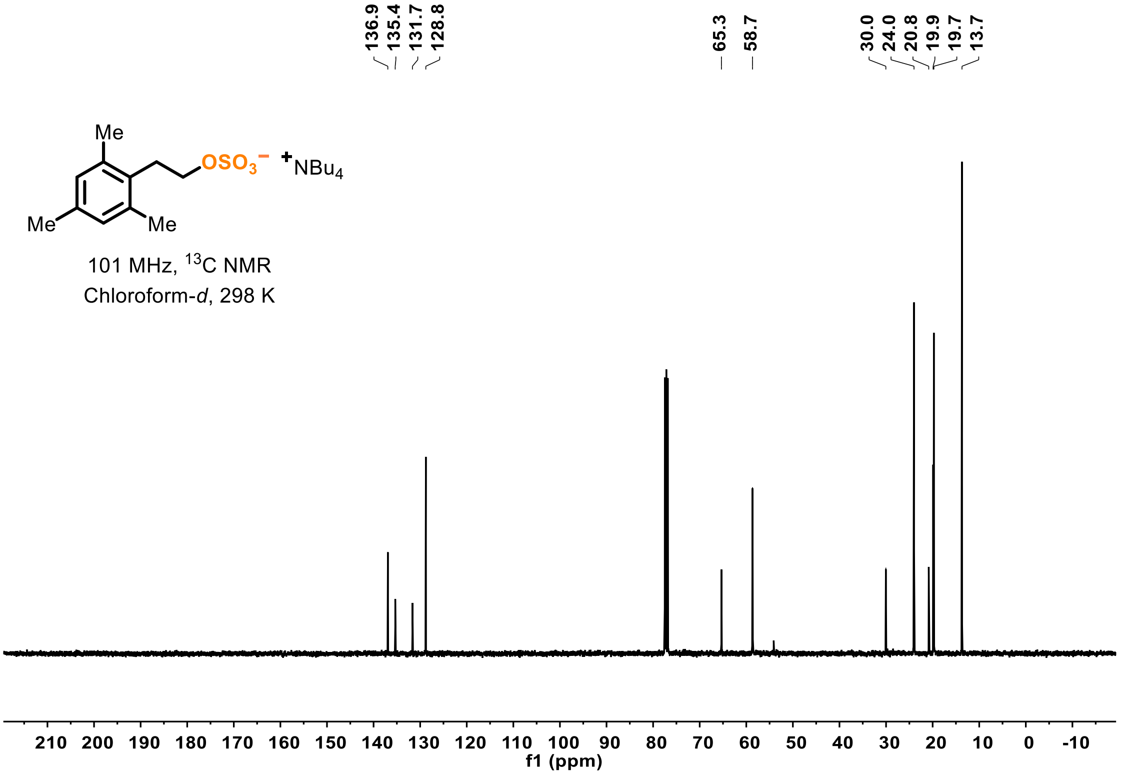

Tetrabutylammonium 4-pentenol sulfate (11)

^{1}H NMR of tetrabutylammonium 4-pentenol sulfate (11)

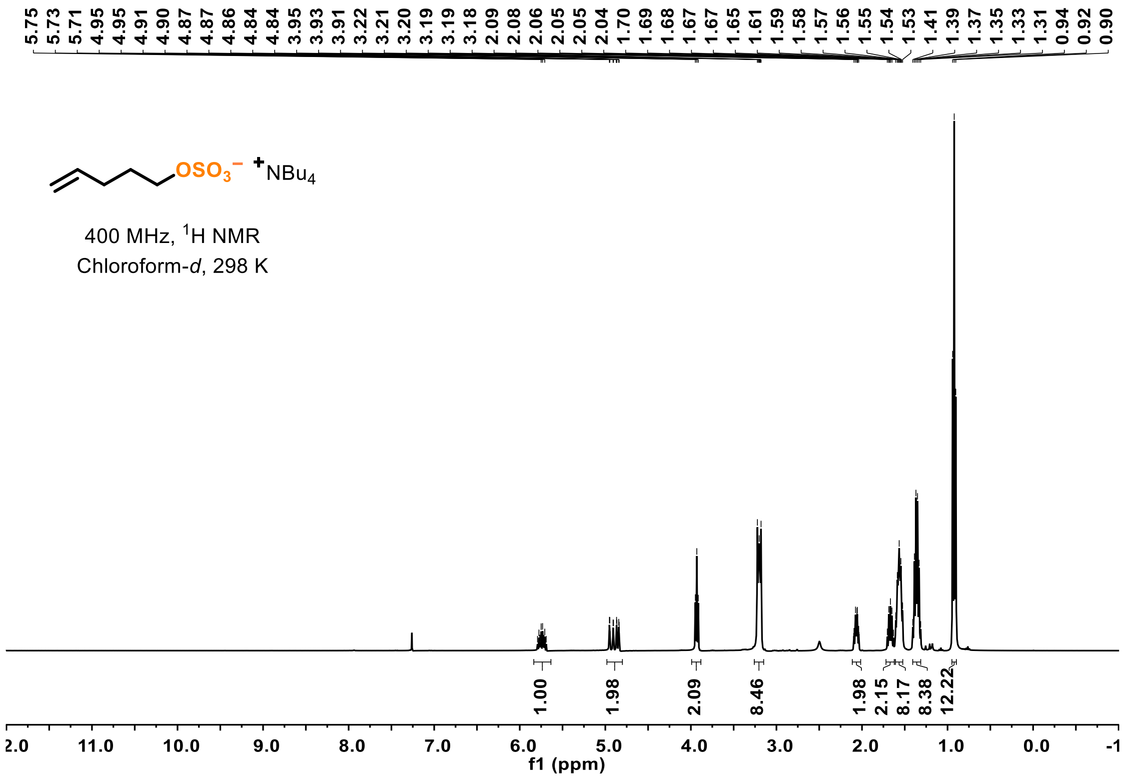

^{13}C NMR of tetrabutylammonium 4-pentenol sulfate (11)

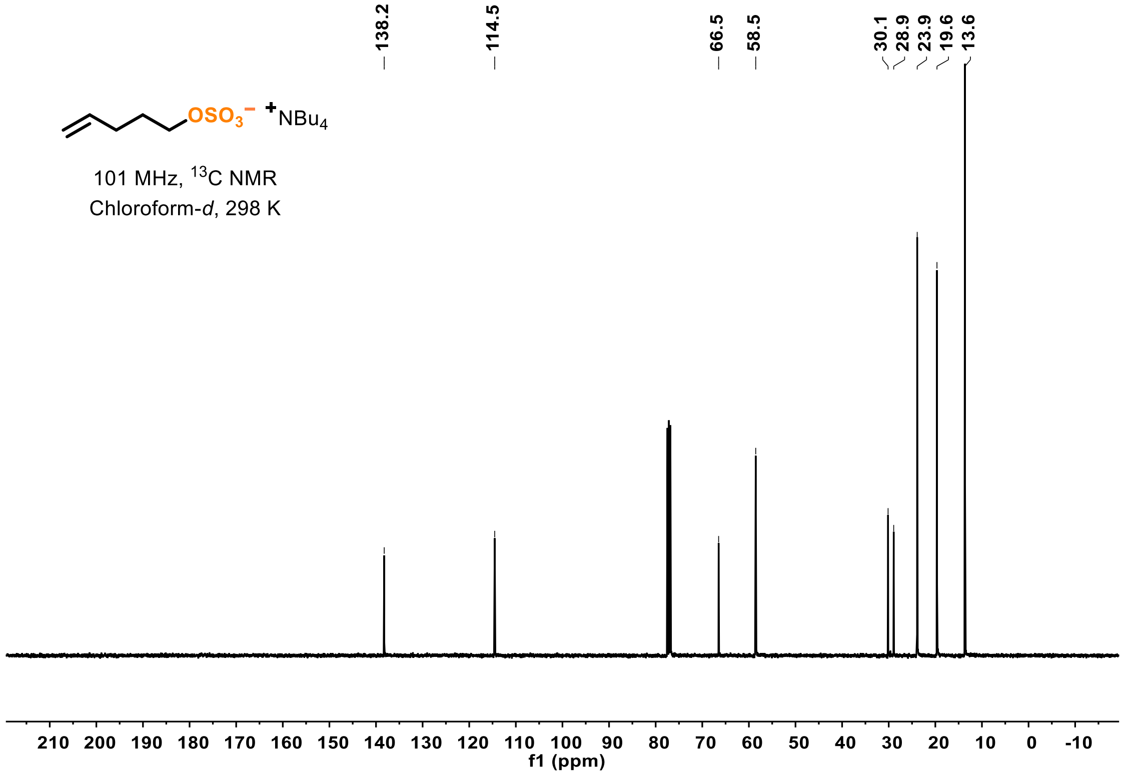

Tetrabutylammonium 4-pentynyl alcohol sulfate (12)

^{1}H NMR of tetrabutylammonium 4-pentynyl alcohol sulfate (12)

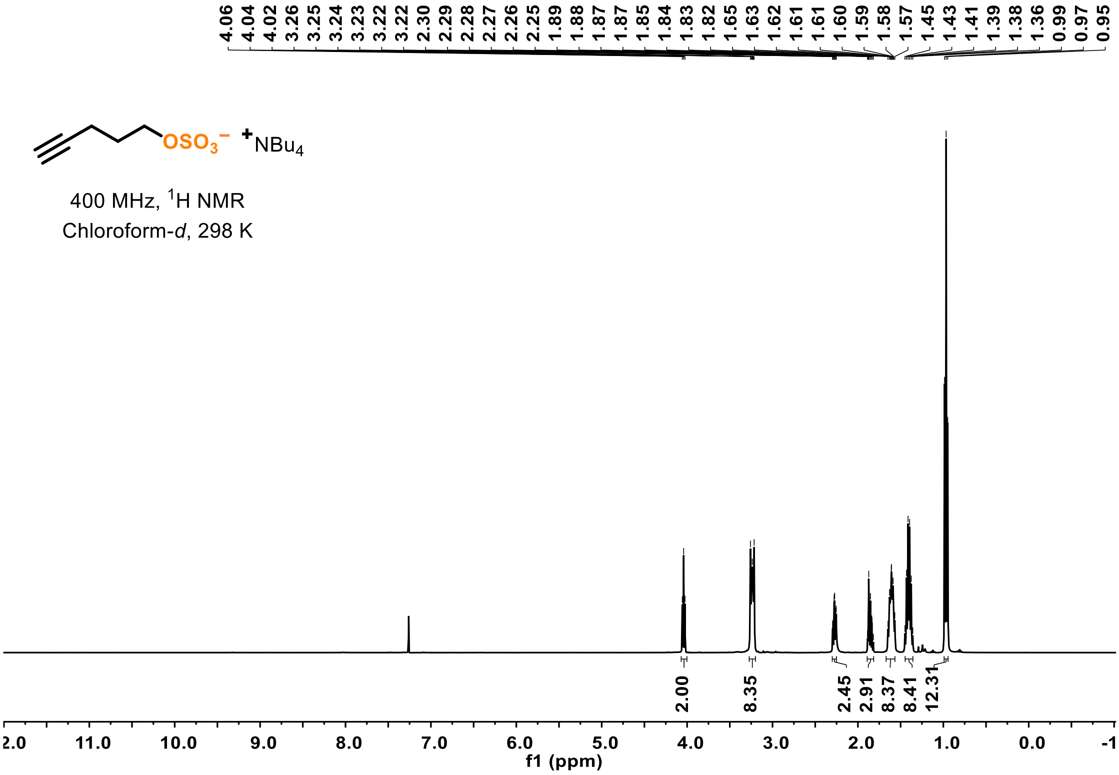

^{13}C NMR of tetrabutylammonium 4-pentynyl alcohol sulfate (12)

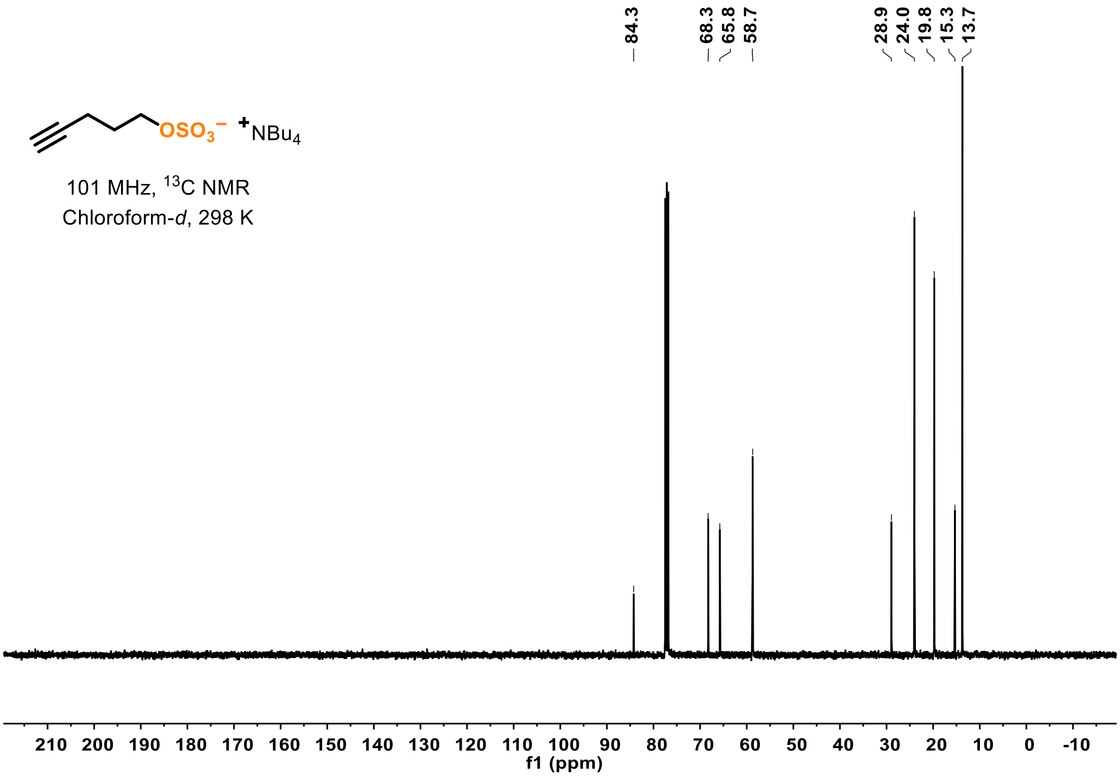

Tetrabutylammonium 3-phenyl-2-propynol sulfate (13)

^{1}H NMR of tetrabutylammonium 3-phenyl-2-propynol sulfate (13)

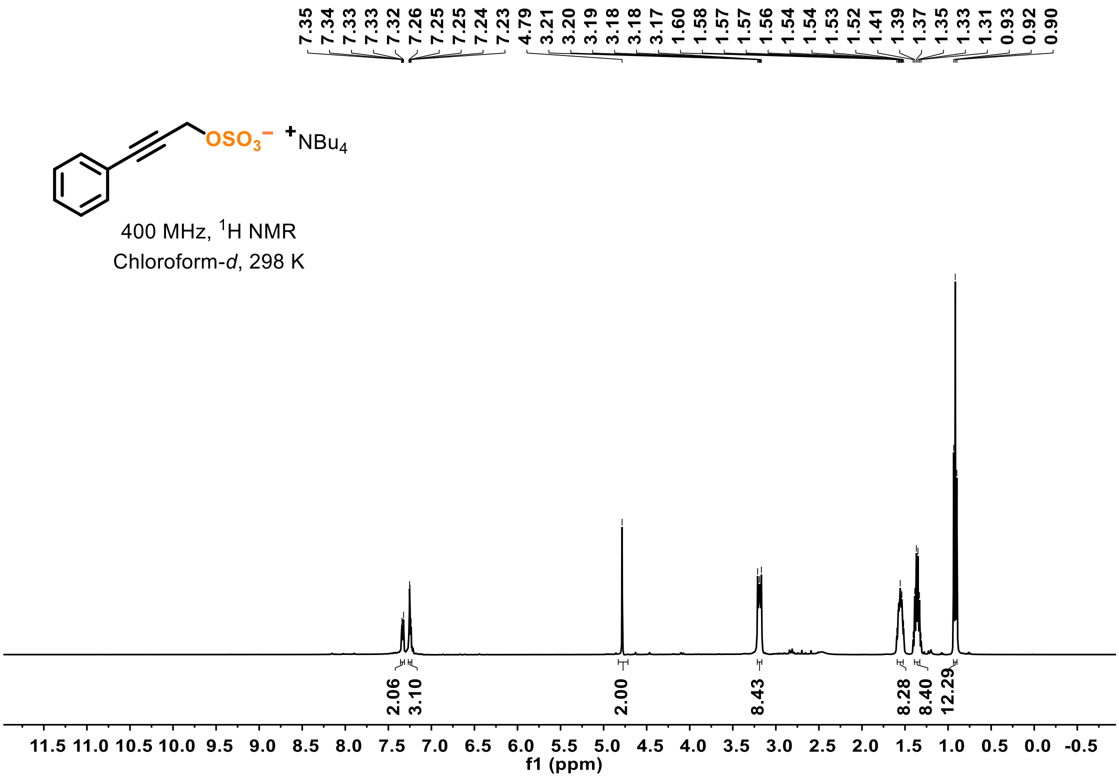

^{13}C NMR of tetrabutylammonium 3-phenyl-2-propynol sulfate (13)

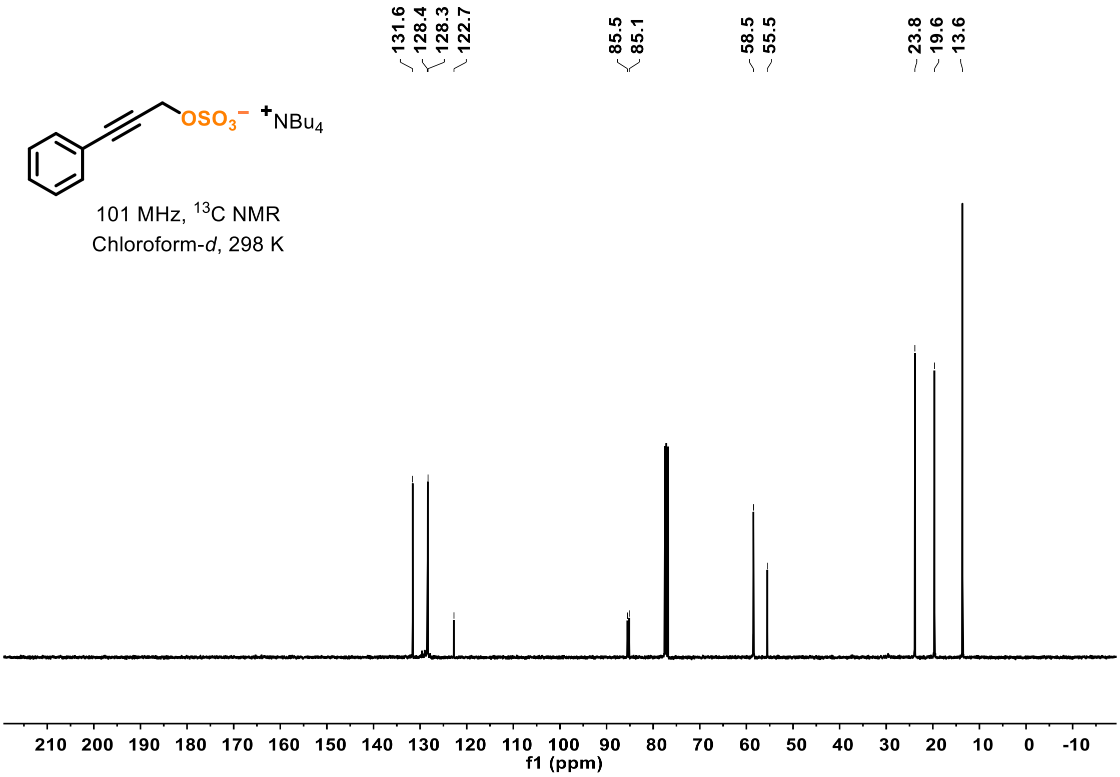

Tetrabutylammonium 4-ethenylphenylmethanol sulfate (14)

^{1}H NMR of tetrabutylammonium 4-ethenylphenylmethanol sulfate (14)

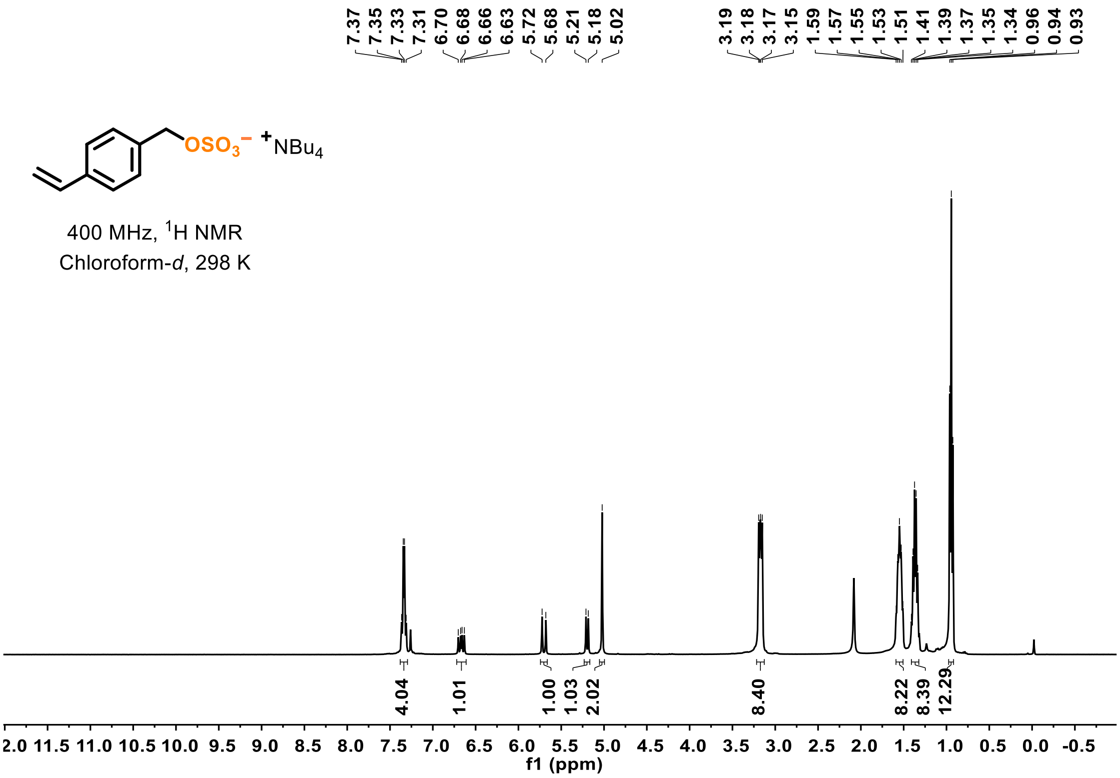

^{13}C NMR of tetrabutylammonium 4-ethenylphenylmethanol sulfate (14)

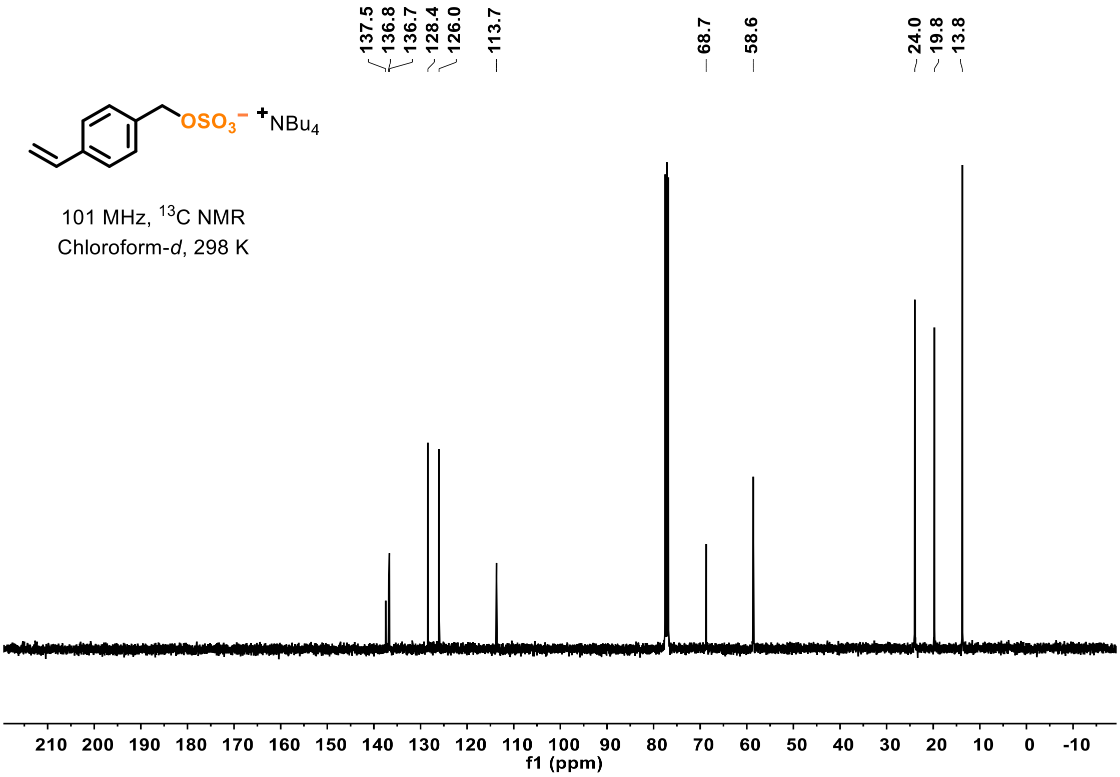

Tetrabutylammonium 4-pinacol-ester-phenylmethanol sulfate (15)

^{1}H NMR of tetrabutylammonium 4-pinacol-ester-phenylmethanol sulfate (15)

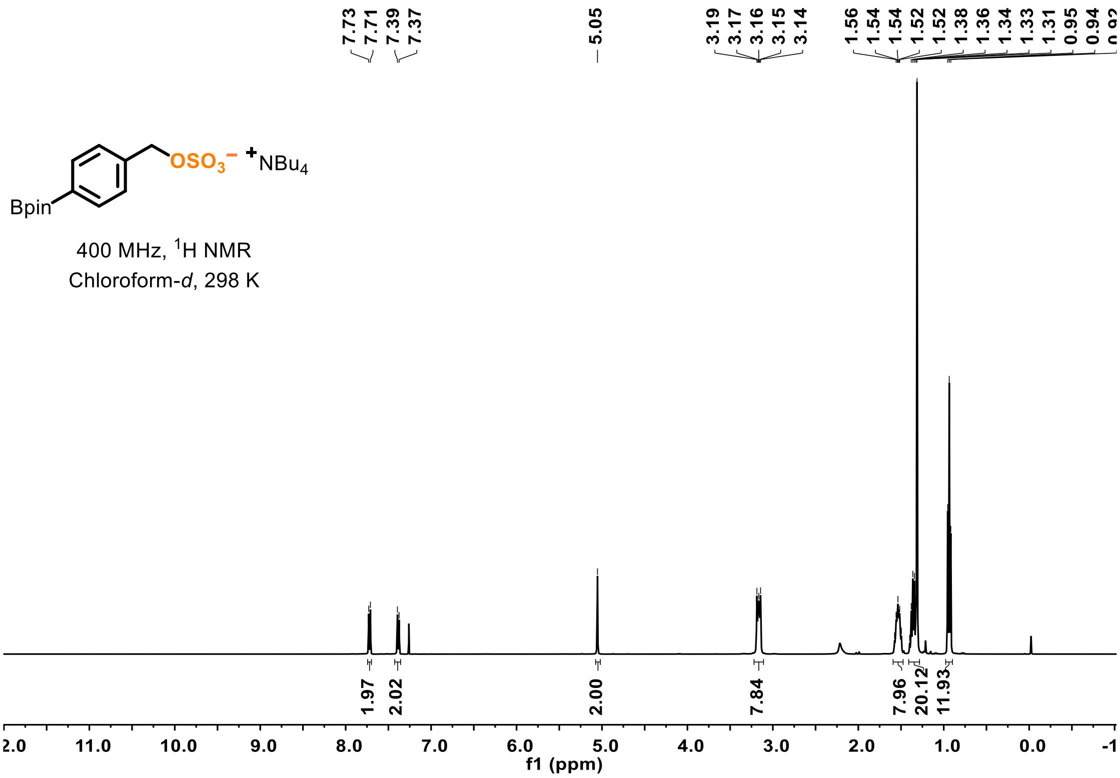

^{13}C NMR of tetrabutylammonium 4-pinacol-ester-phenylmethanol sulfate (15)

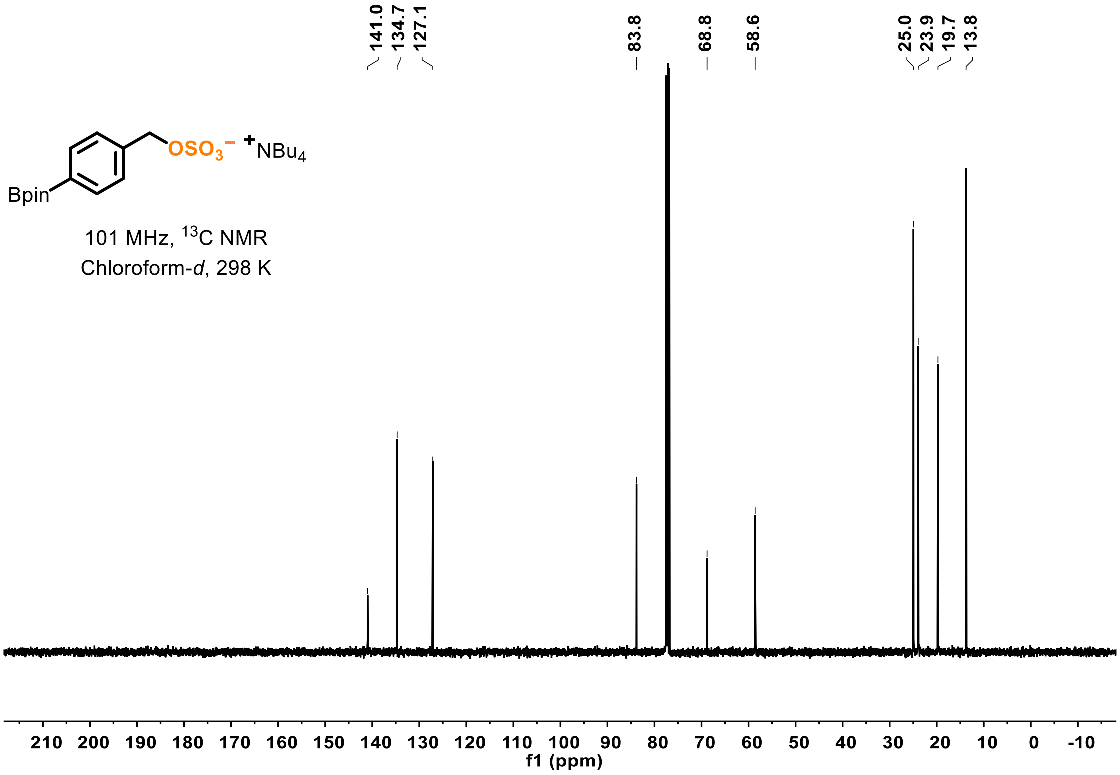

Tetrabutylammonium 4-formyllphenylmethanol sulfate (16)

^{1}H NMR of tetrabutylammonium 4-formyllphenylmethanol sulfate (16)

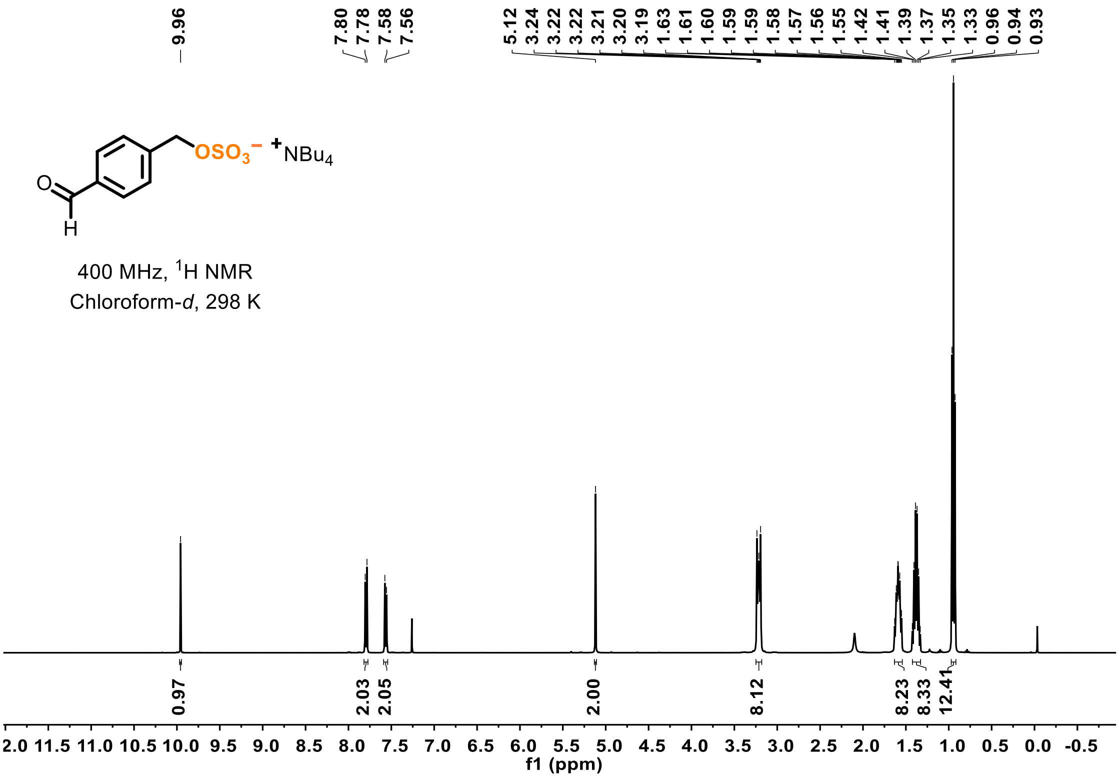

^{13}C NMR of tetrabutylammonium 4-formyllphenylmethanol sulfate (16)

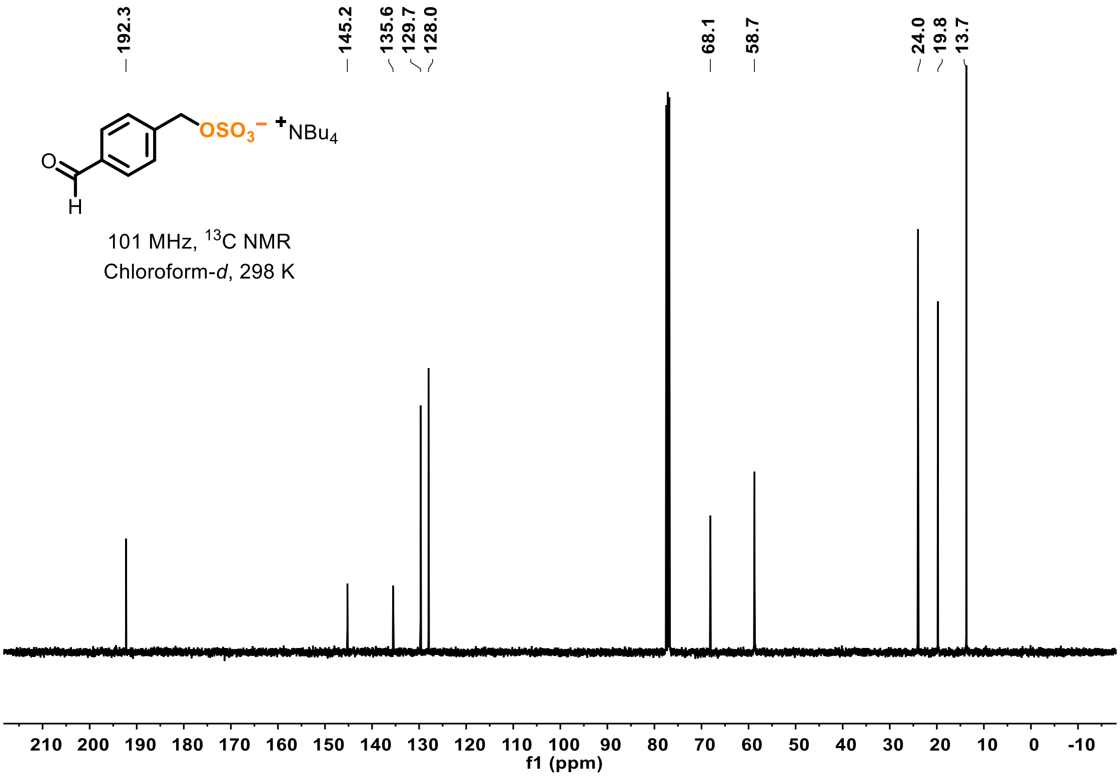

Tetrabutylammonium 4-sulfoxylphenylmethanol sulfate (17)

^{1}H NMR of tetrabutylammonium 4-sulfoxylphenylmethanol sulfate (17)

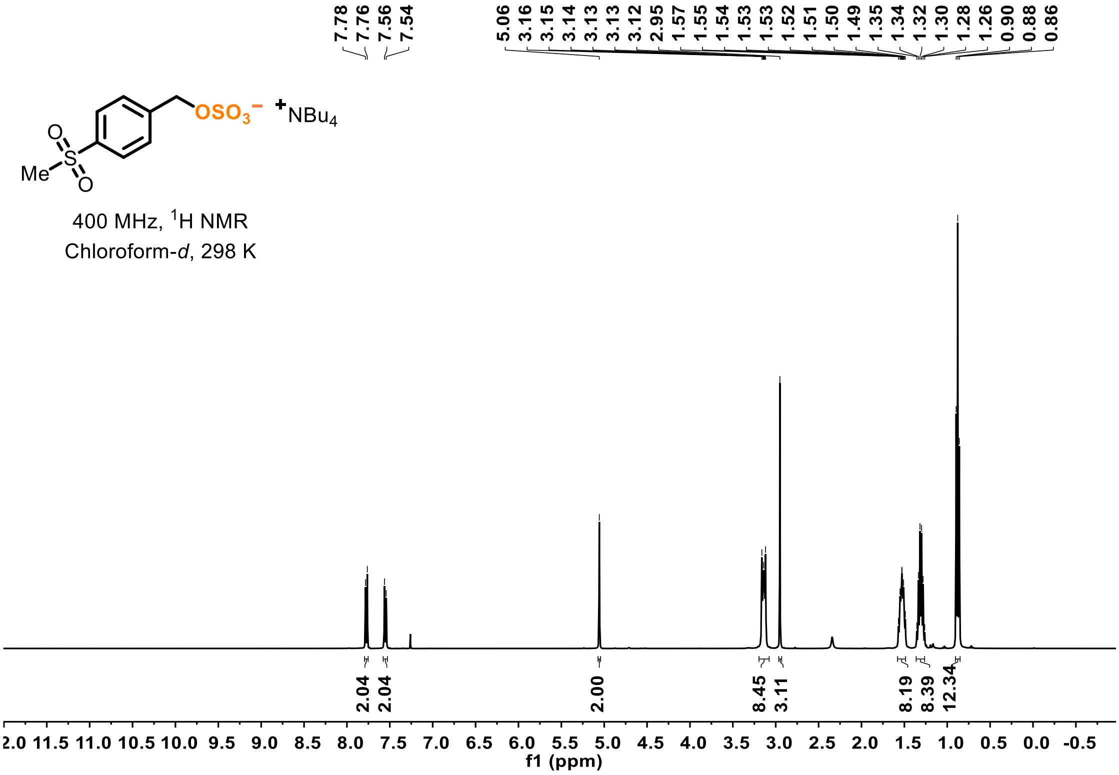

^{13}C NMR of tetrabutylammonium 4-sulfoxylphenylmethanol sulfate (17)

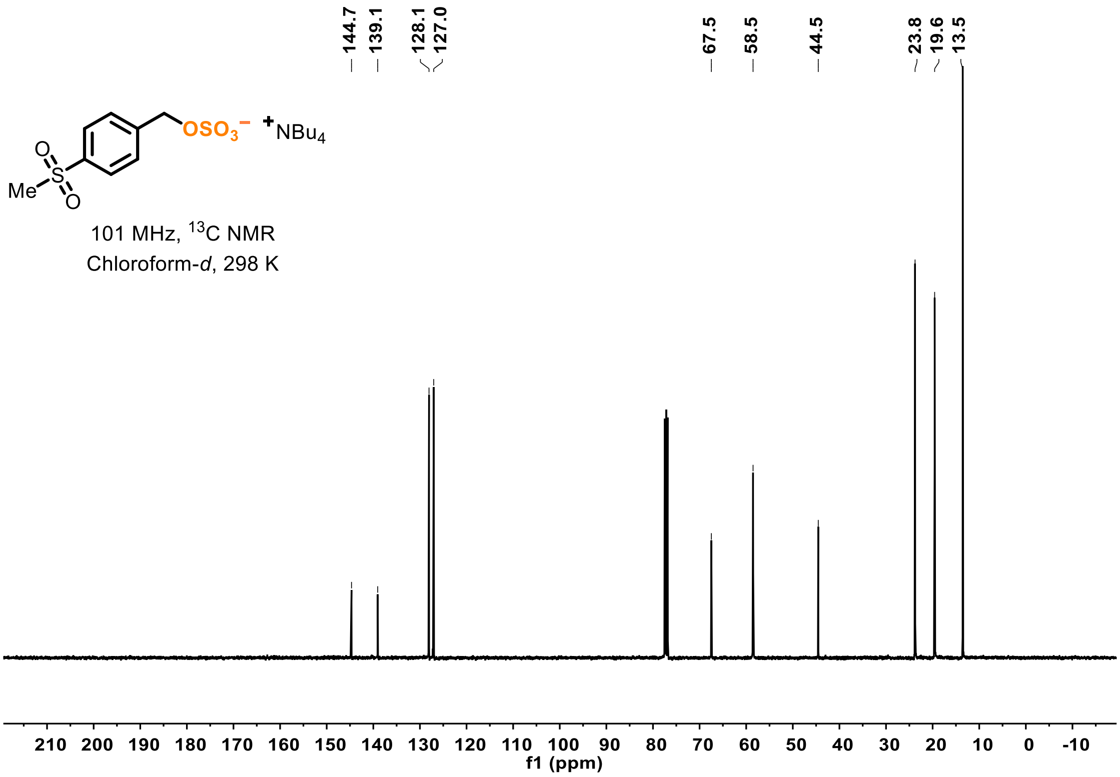

Tetrabutylammonium thiophene-3-ethanol sulfate (18)

^{1}H NMR of tetrabutylammonium thiophene-3-ethanol sulfate (18)

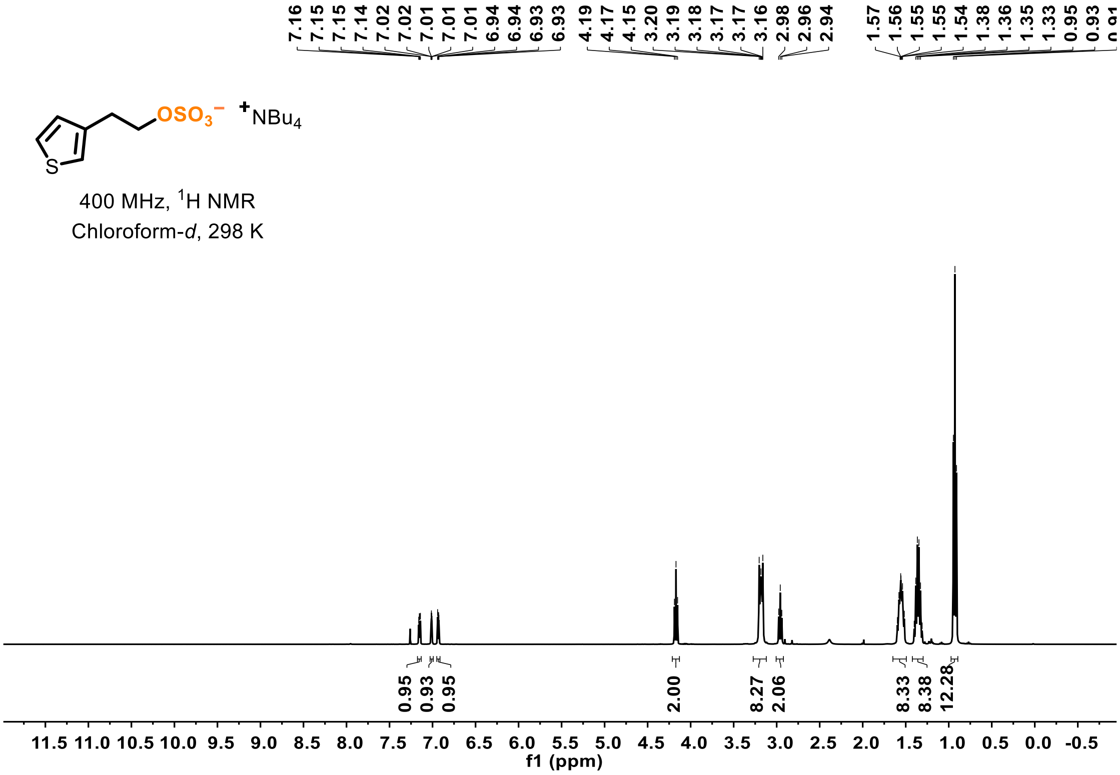

^{13}C NMR of tetrabutylammonium thiophene-3-ethanol sulfate (18)

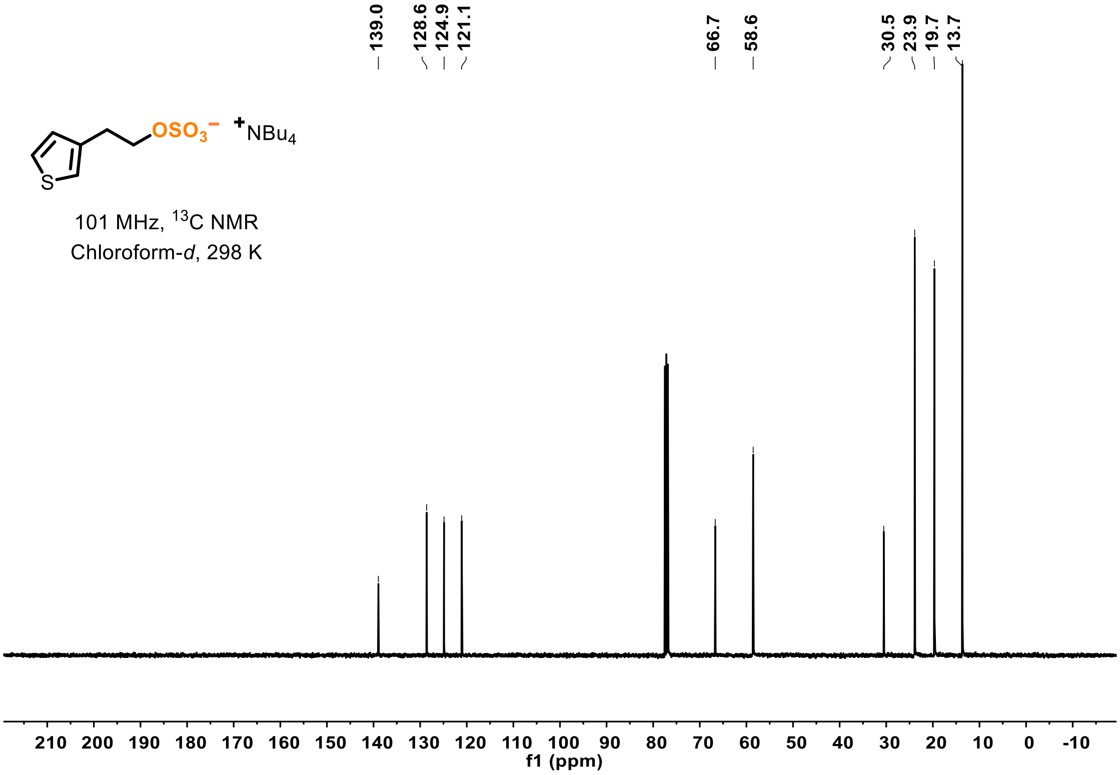

Tetrabutylammonium trichloroethanol sulfate (19)

^{1}H NMR of tetrabutylammonium trichloroethanol sulfate (19)

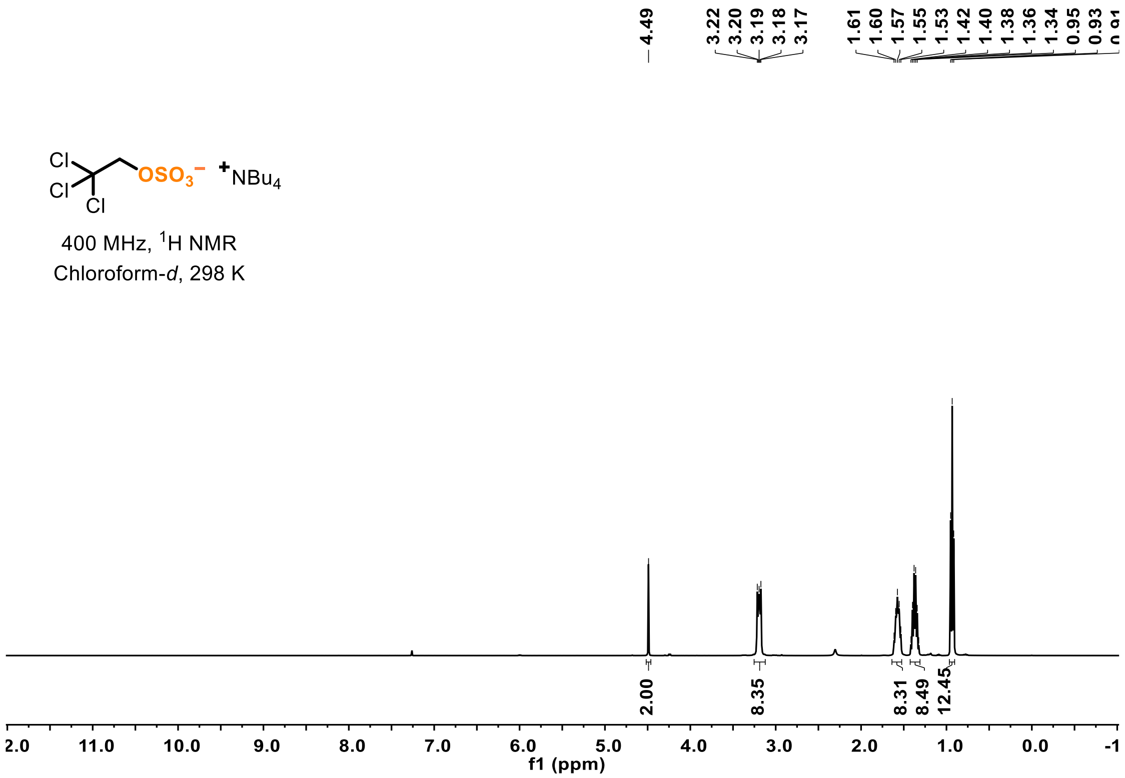

^{13}C NMR of tetrabutylammonium trichloroethanol sulfate (19)

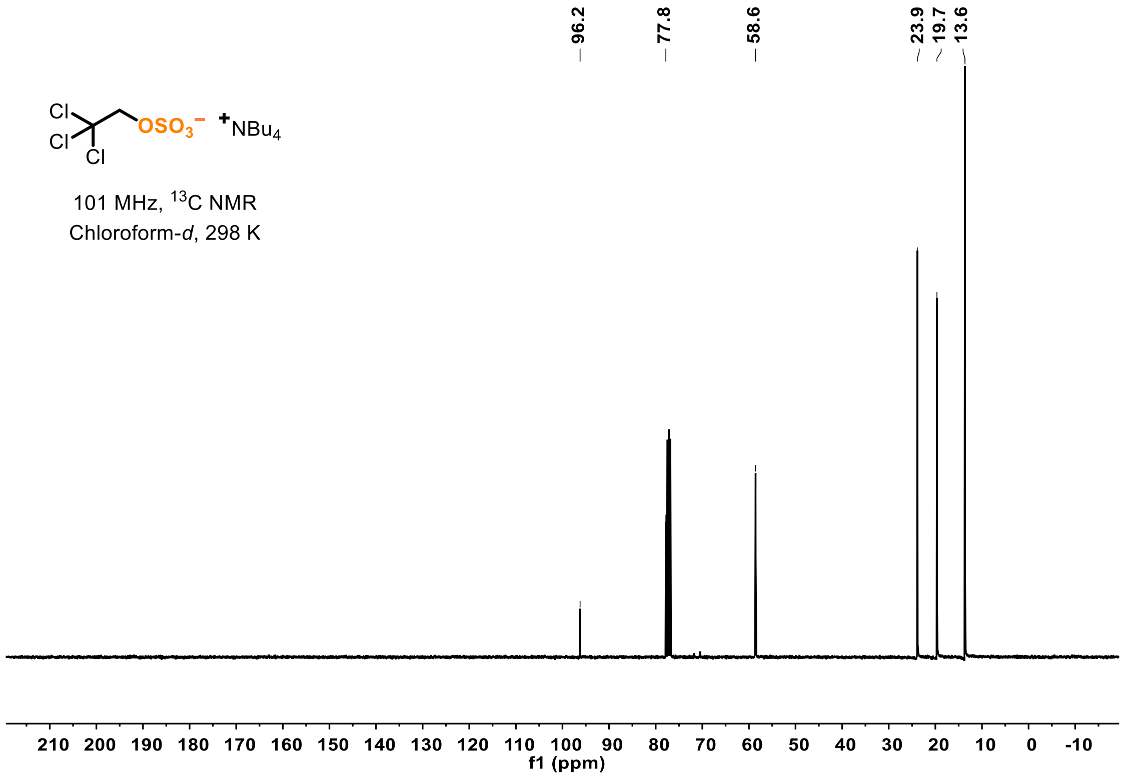

Tetrabutylammonium 2-phenylethanethiol sulfate (20)

^{1}H NMR of tetrabutylammonium 2-phenylethanethiol sulfate (20)

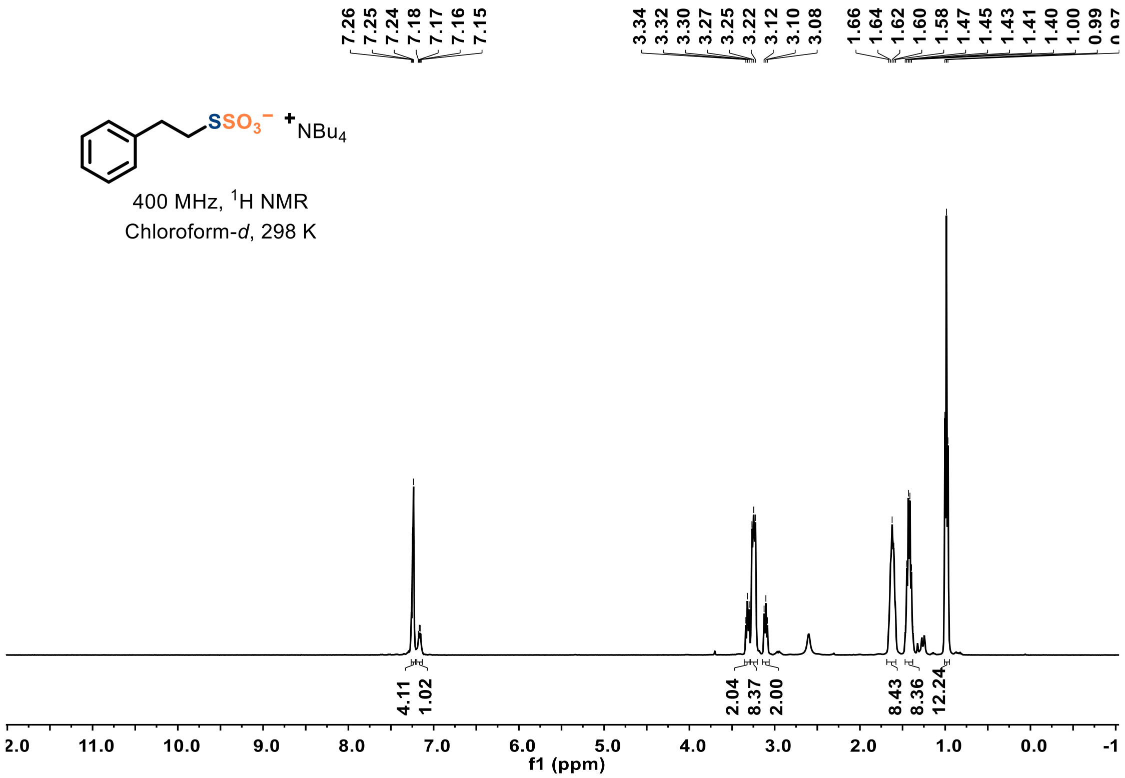

^{13}C NMR of tetrabutylammonium 2-phenylethanethiol sulfate (20)

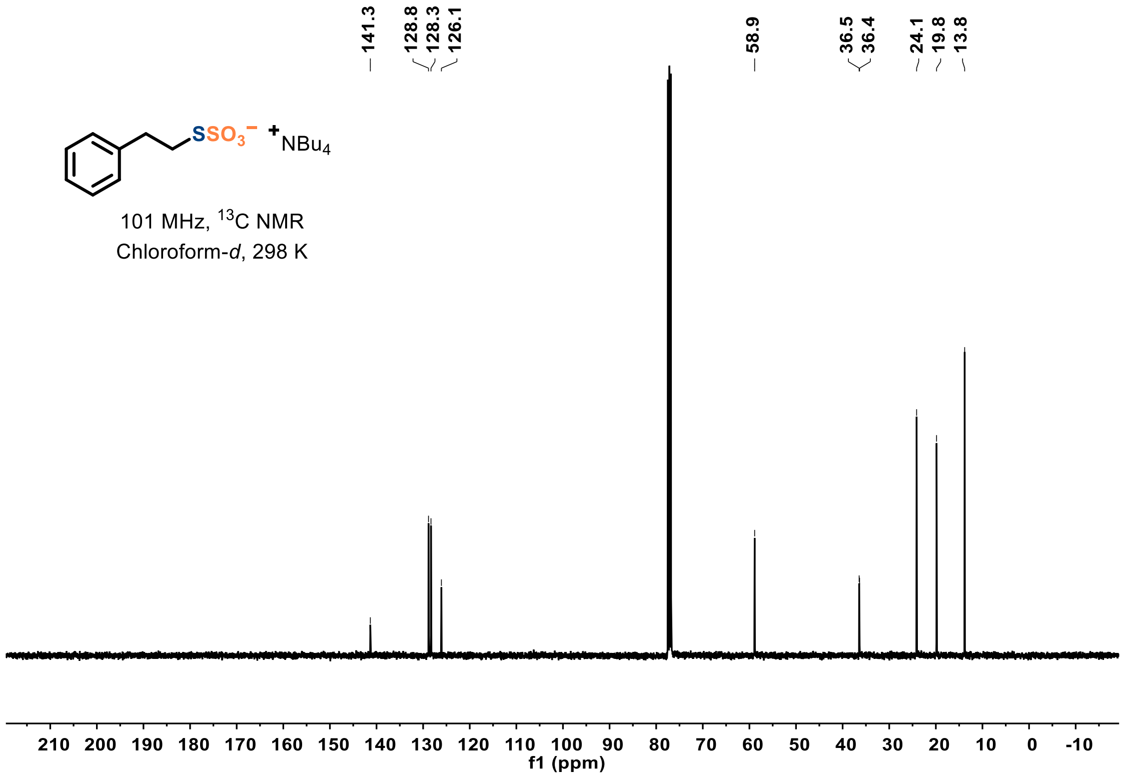

Tetrabutylammonium 4-phenyl-2-butanol sulfate (21)

^{1}H NMR of tetrabutylammonium 4-phenyl-2-butanol sulfate (21)

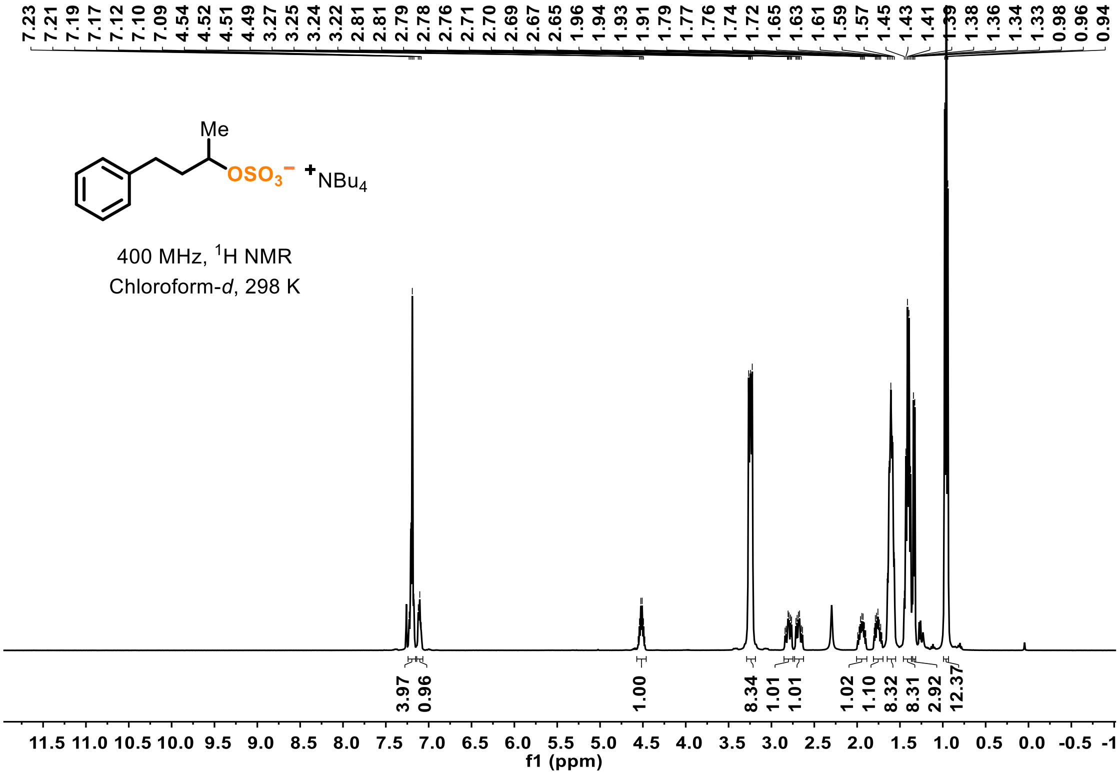

^{13}C NMR of tetrabutylammonium 4-phenyl-2-butanol sulfate (21)

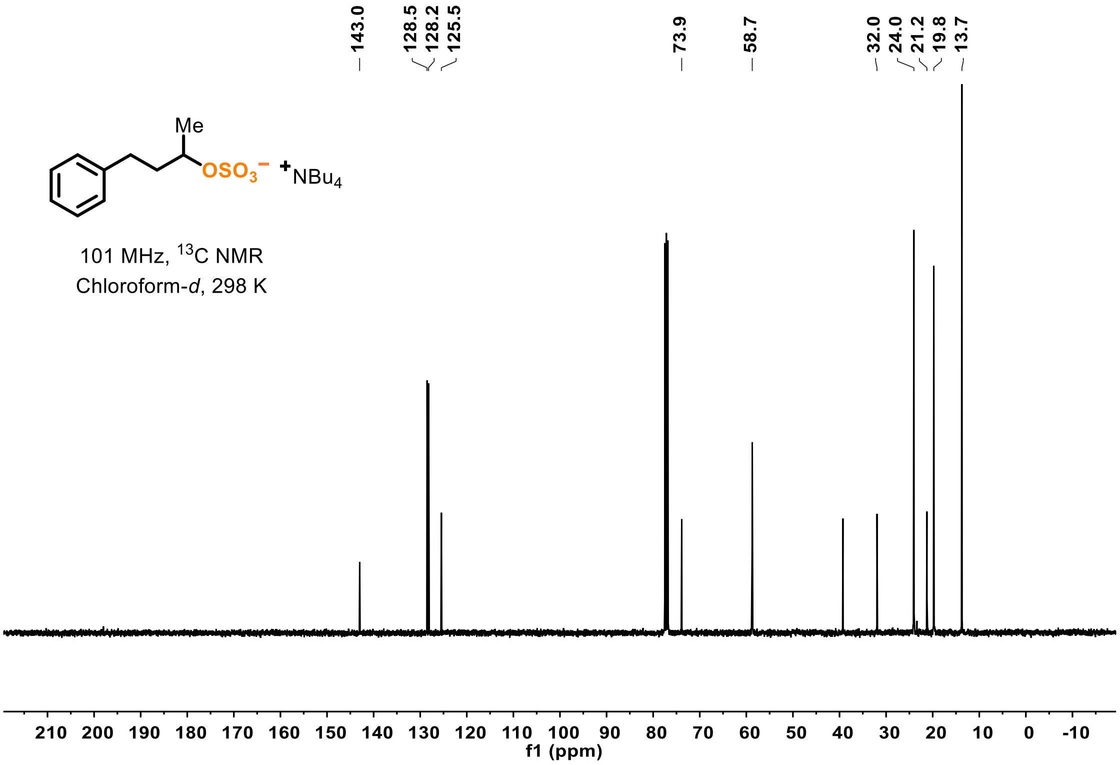

Tetrabutylammonium methyl 3-hydroxyhexanoate sulfate (22)

^{1}H NMR of tetrabutylammonium methyl 3-hydroxyhexanoate sulfate (22)

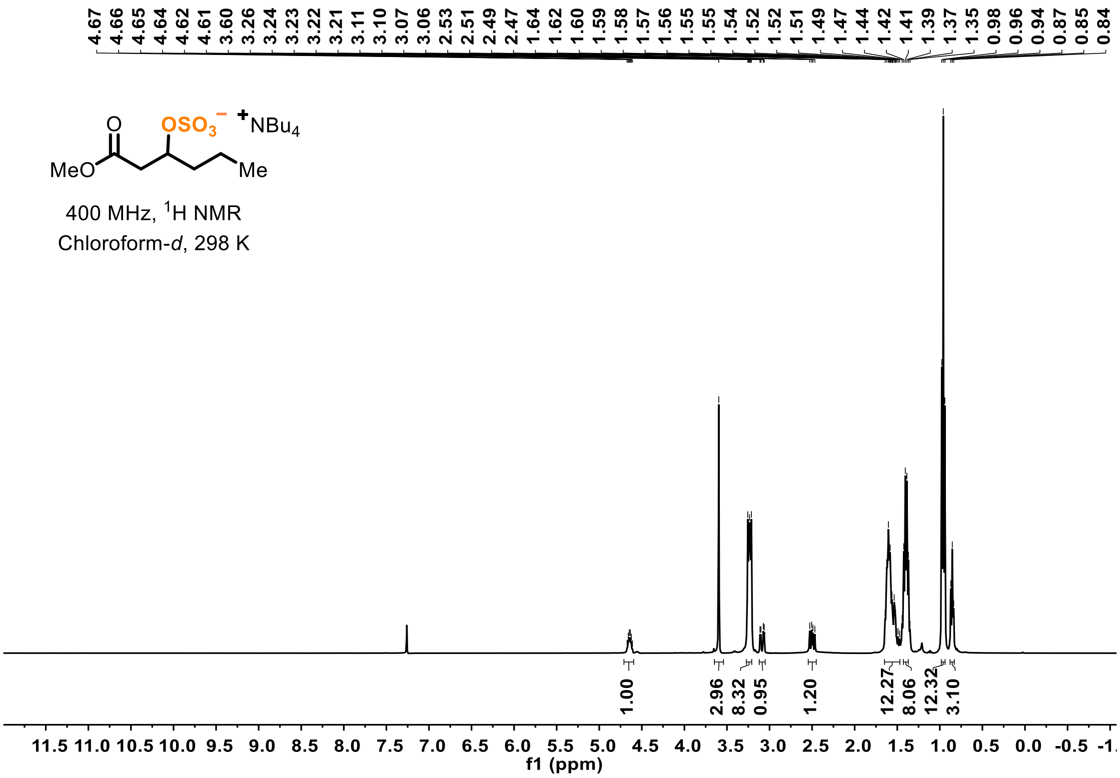

^{13}C NMR of tetrabutylammonium methyl 3-hydroxyhexanoate sulfate (22)

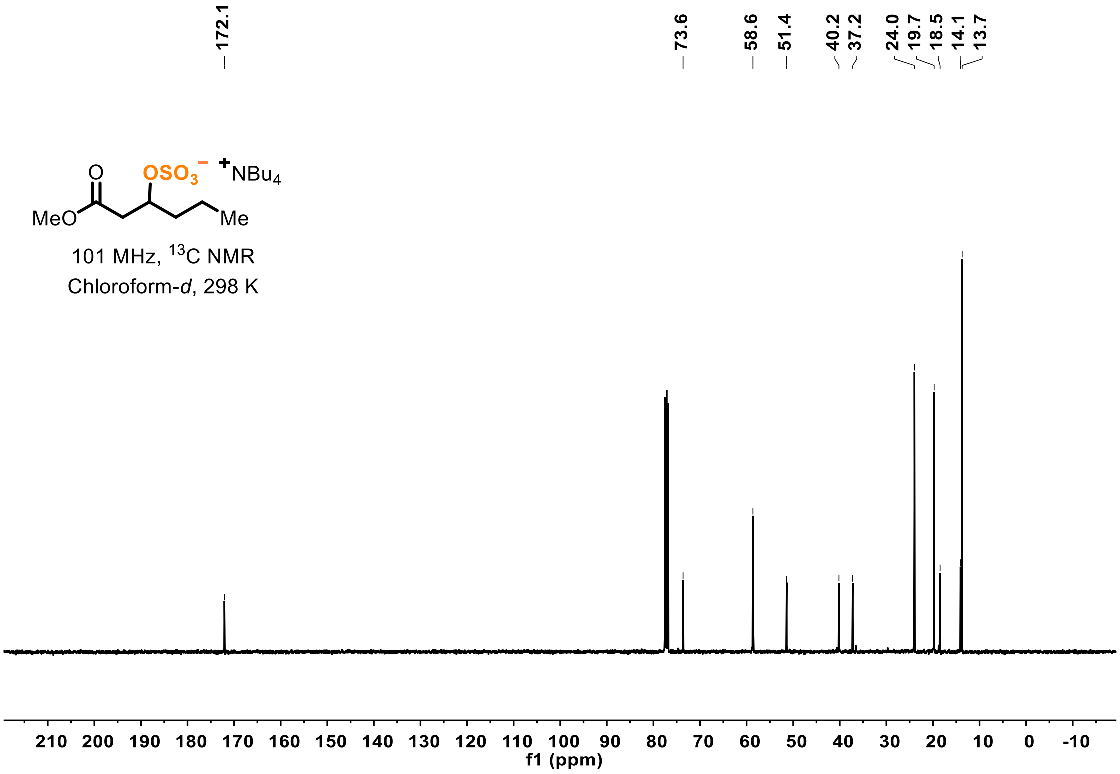

Tetrabutylammonium 2-indanol sulfate (23)

^{1}H NMR of tetrabutylammonium 2-indanol sulfate (23)

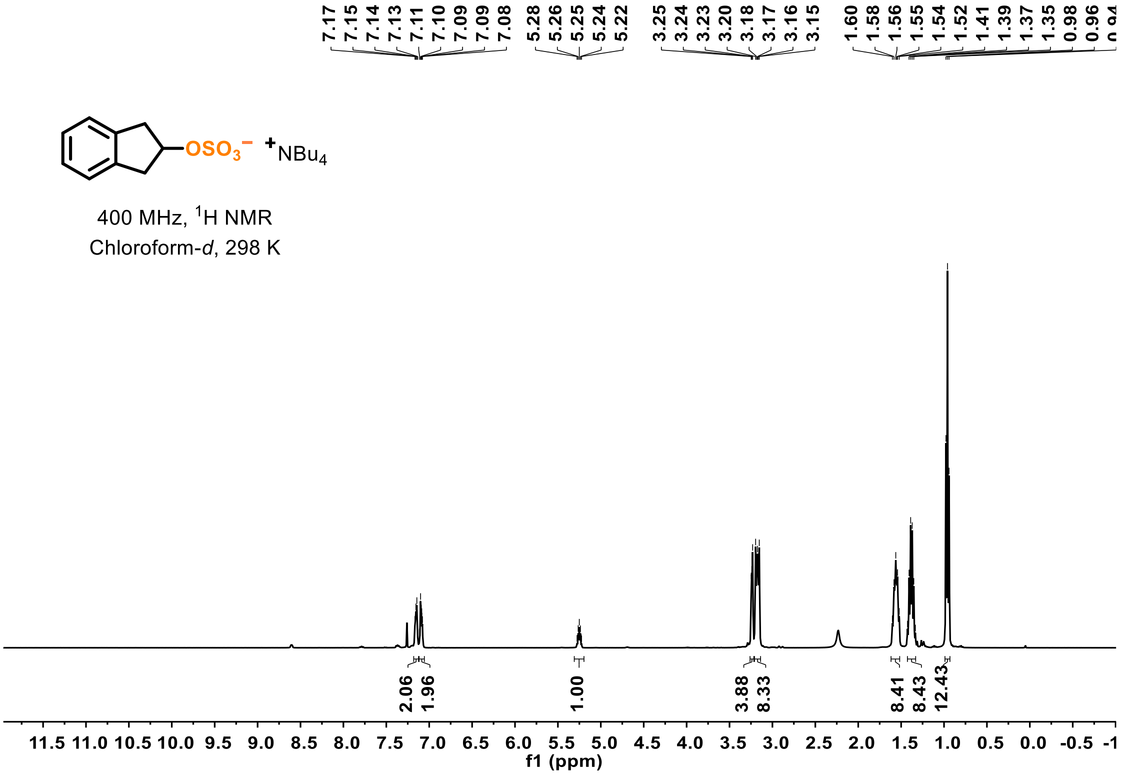

^{13}C NMR of tetrabutylammonium 2-indanol sulfate (23)

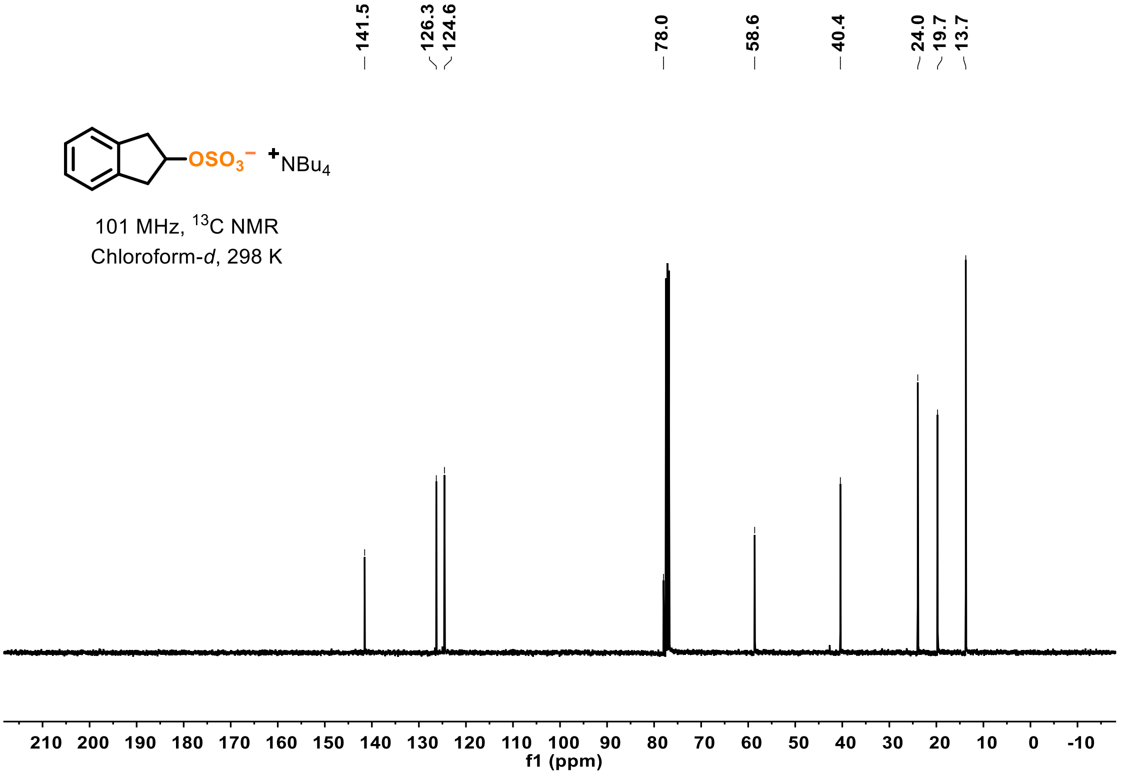

Tetrabutylammonium DL-menthol sulfate (24)

^{1}H NMR of tetrabutylammonium DL-menthol sulfate (24)

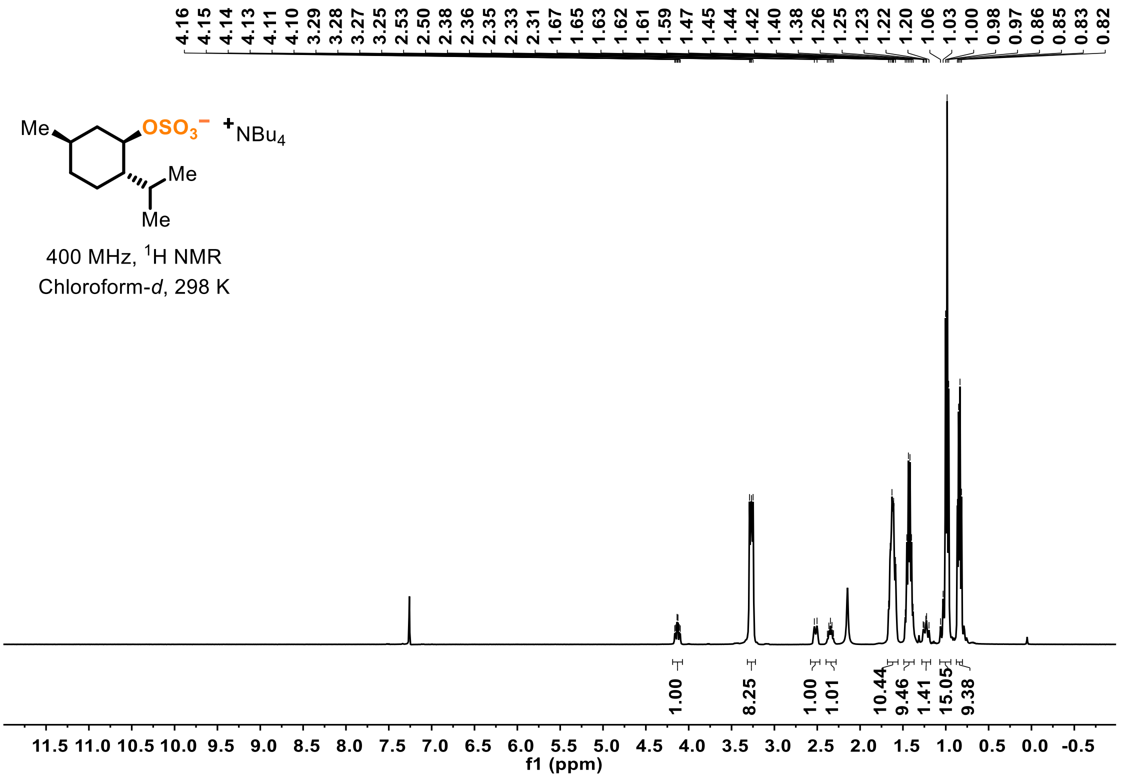

^{13}C NMR of tetrabutylammonium DL-menthol sulfate (24)

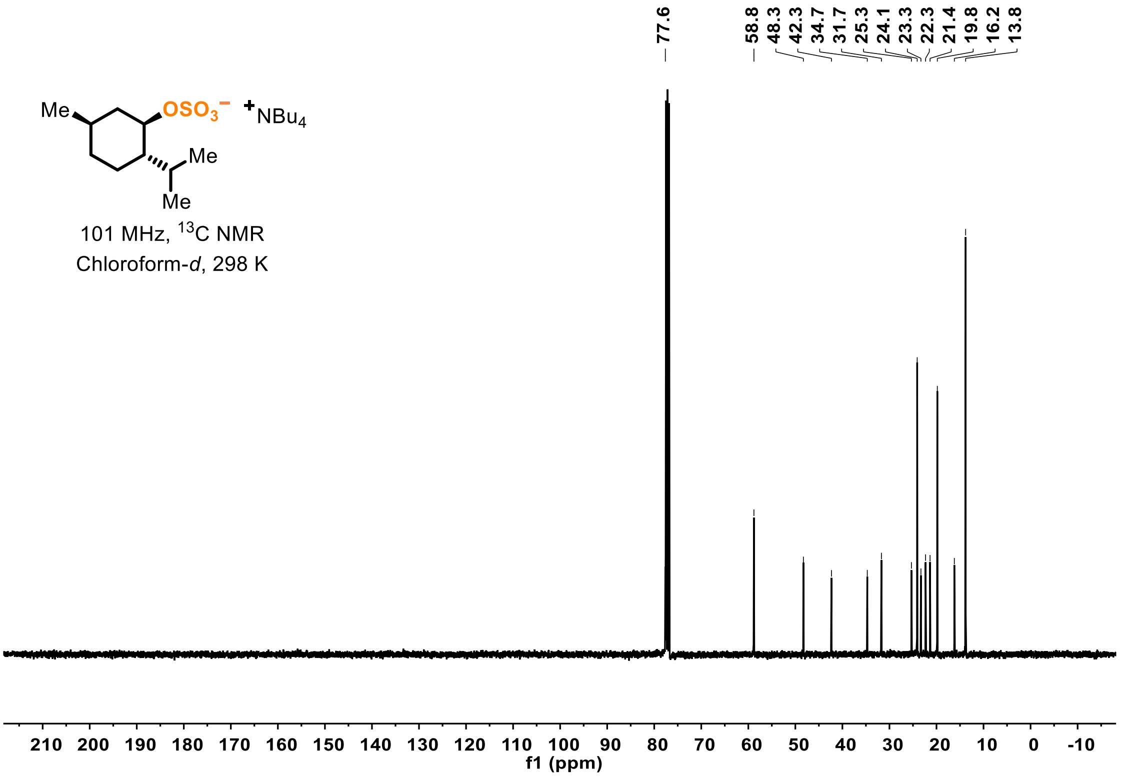

Tetrabutylammonium 2-adamantanol sulfate (25)

^{1}H NMR of tetrabutylammonium 2-adamantanol sulfate (25)

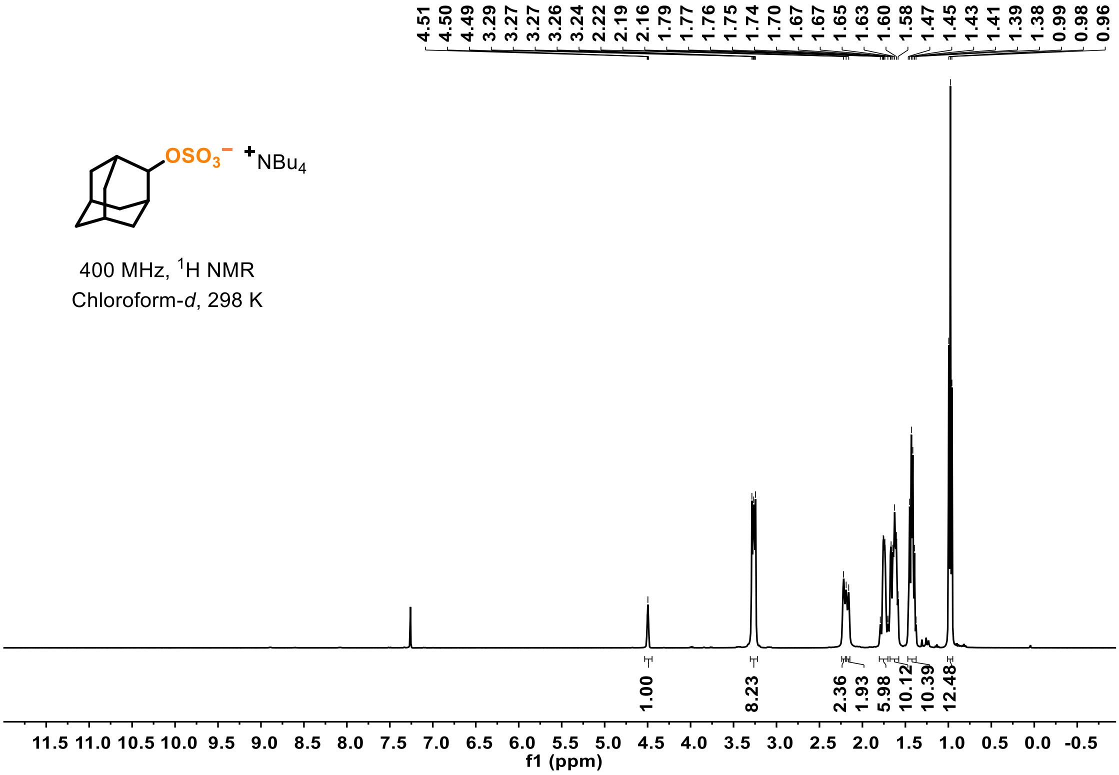

^{13}C NMR of tetrabutylammonium 2-adamantanol sulfate (25)

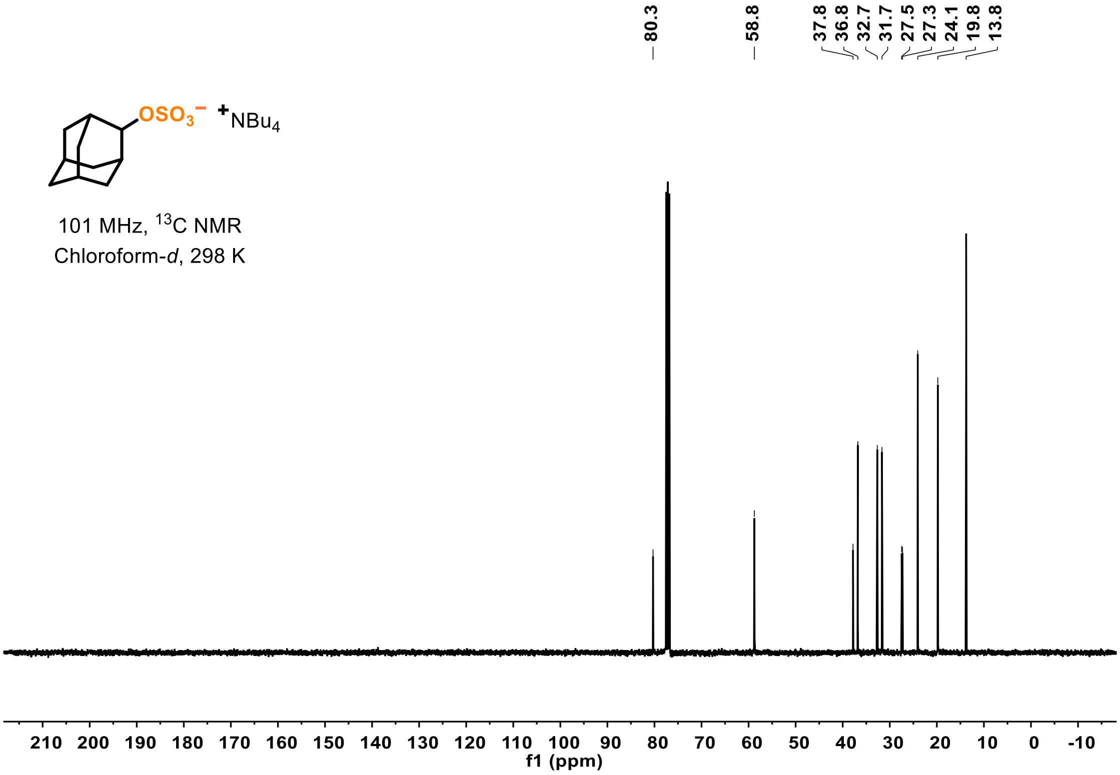

Tetrabutylammonium cyclododecanol sulfate (26)

^{1}H NMR of tetrabutylammonium cyclododecanol sulfate (26)

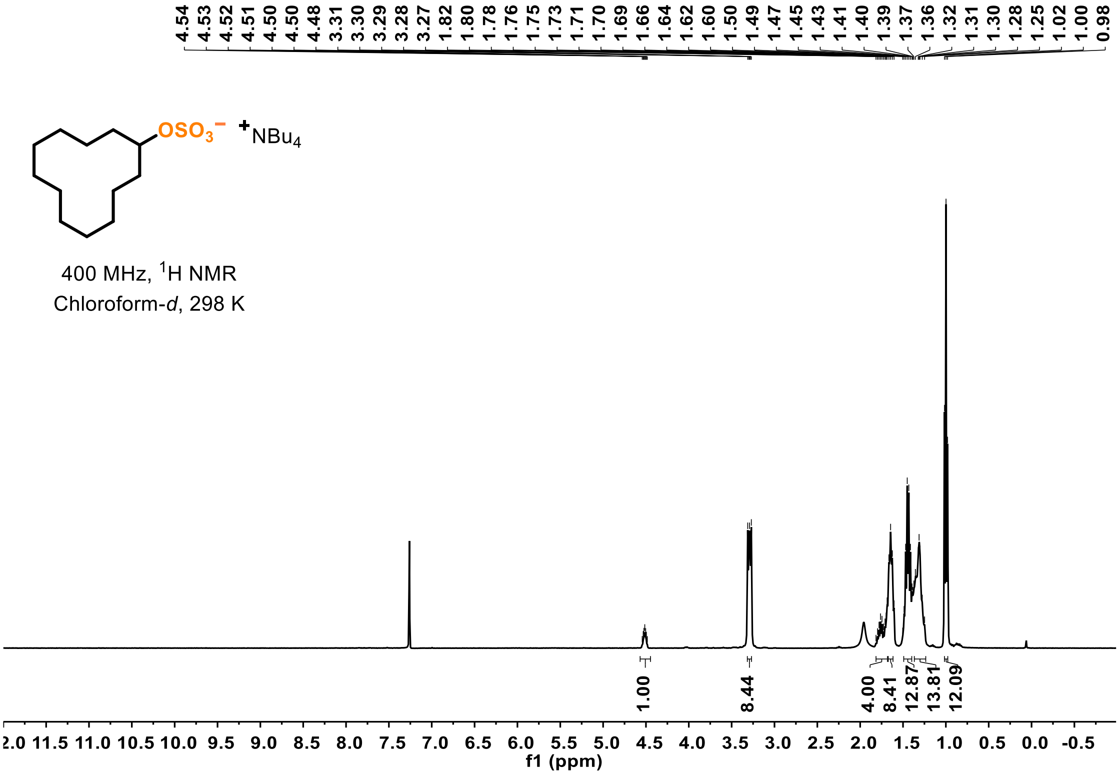

^{13}C NMR of tetrabutylammonium cyclododecanol sulfate (26)

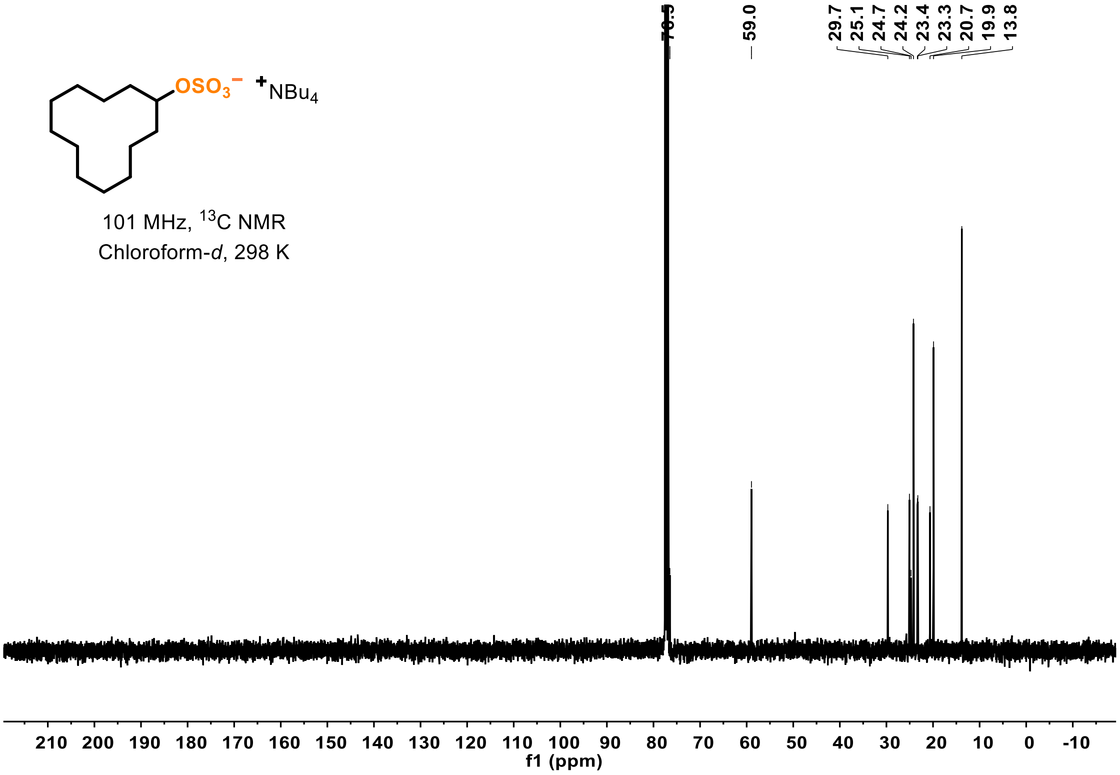

Tetrabutylammonium *N*-tosyl-4-piperidinol sulfate (27)

^{1}H NMR of tetrabutylammonium *N*-tosyl-4-piperidinol sulfate (27)

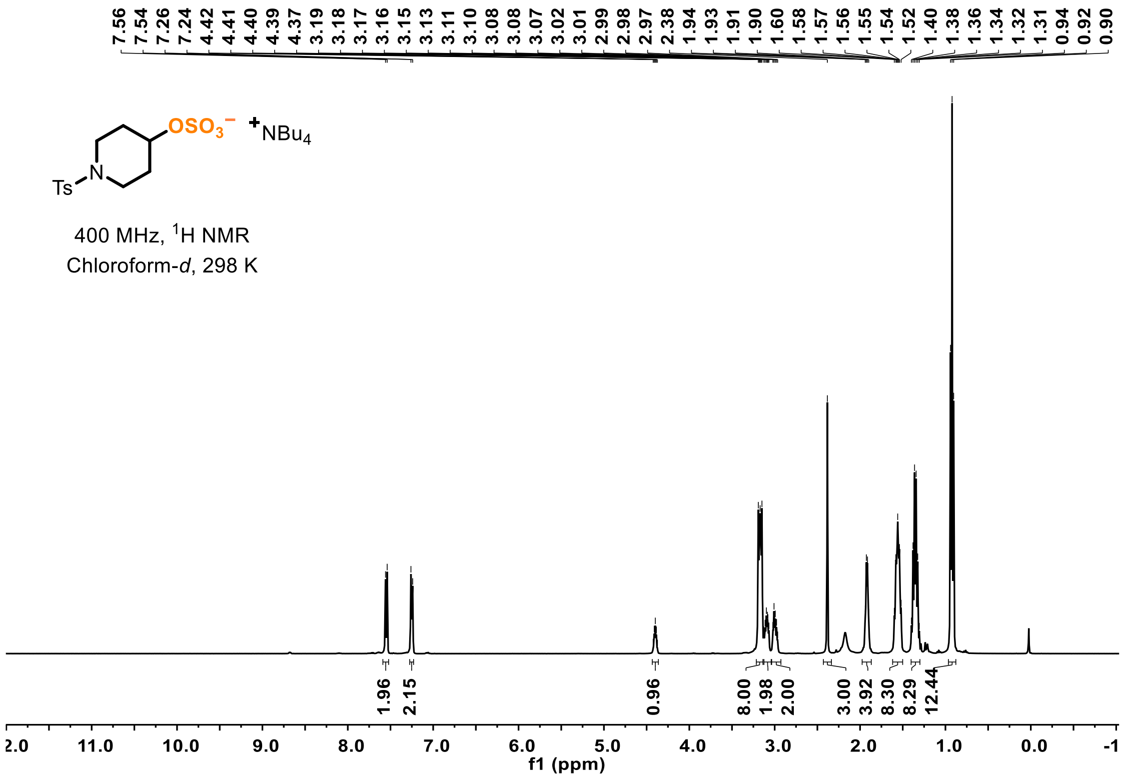

^{13}C NMR of tetrabutylammonium *N*-tosyl-4-piperidinol sulfate (27)

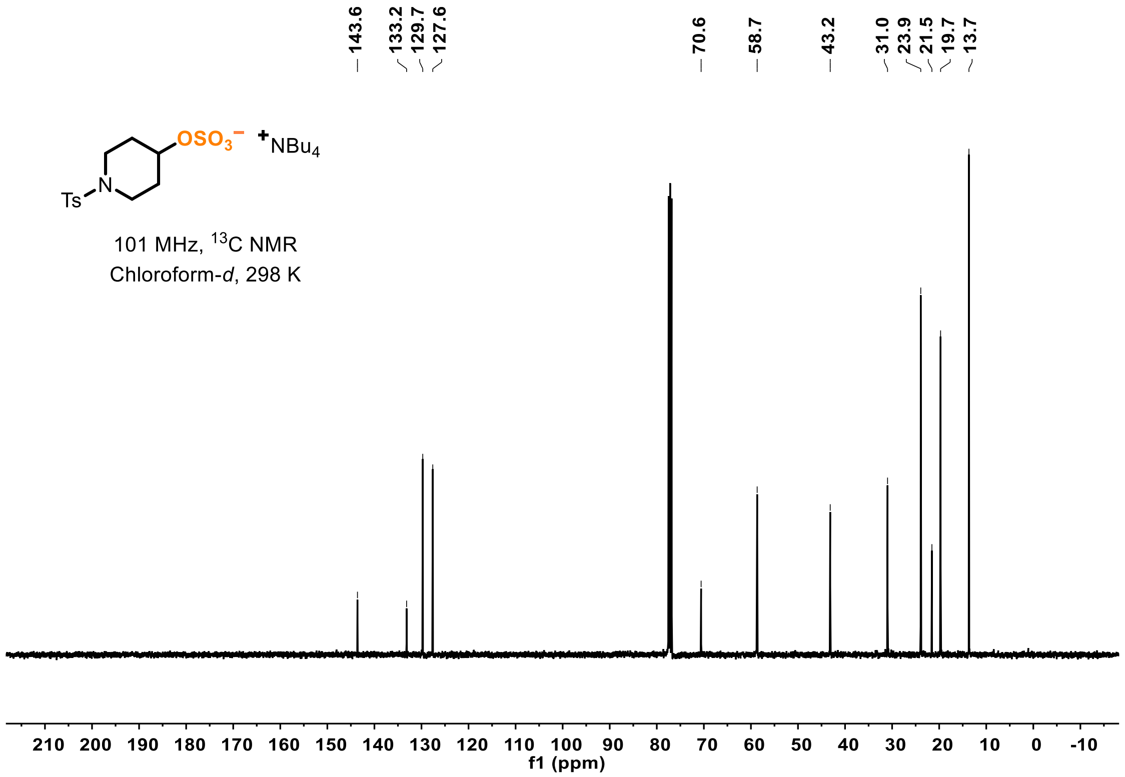

Tetrabutylammonium DL-pantolactone sulfate (28)

^{1}H NMR of tetrabutylammonium DL-pantolactone sulfate (28)

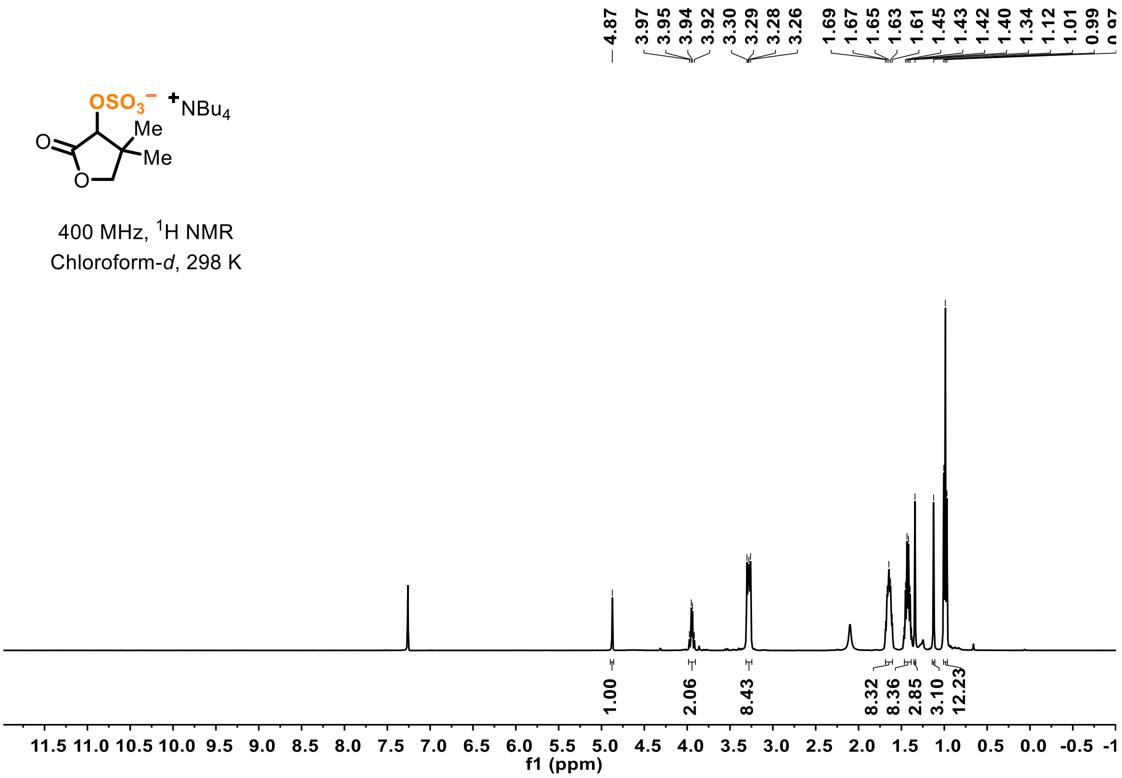

^{13}C NMR of tetrabutylammonium DL-pantolactone sulfate (28)

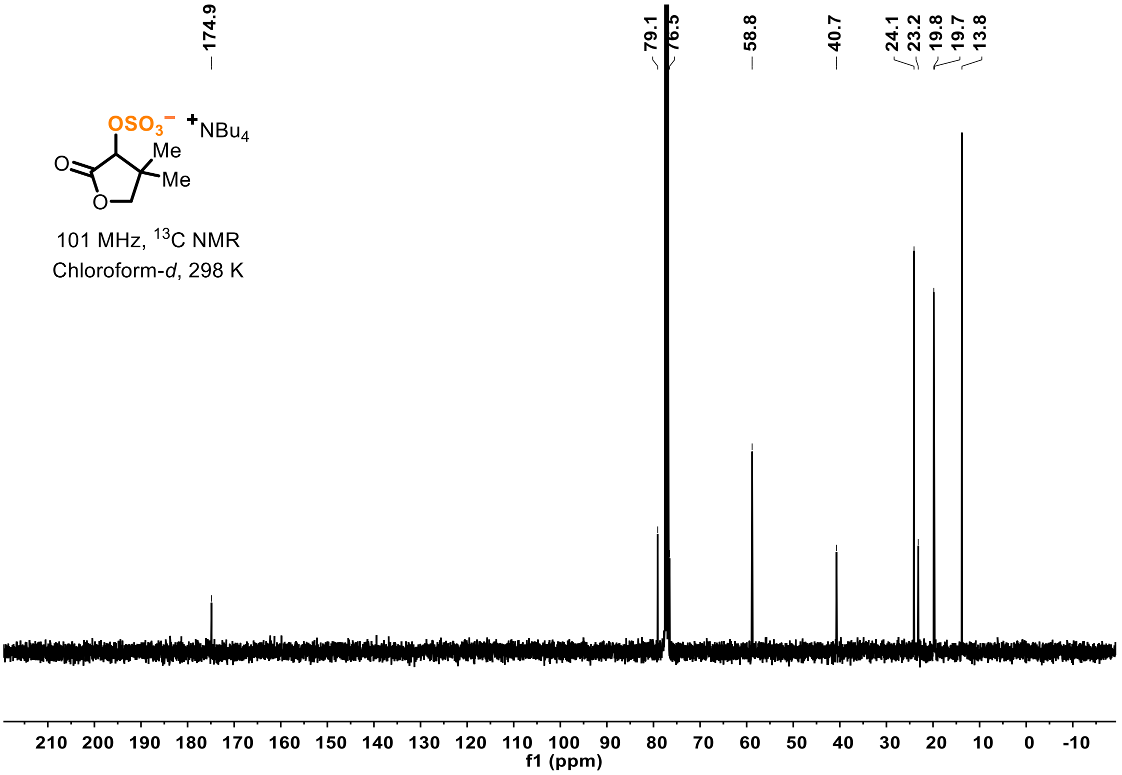

Tetrabutylammonium 4-chloro-6,7-dihydro-5H-[1]pyrindin-7-ol sulfate (29)

^{1}H NMR of tetrabutylammonium 4-chloro-6,7-dihydro-5H-[1]pyrindin-7-ol sulfate (29)

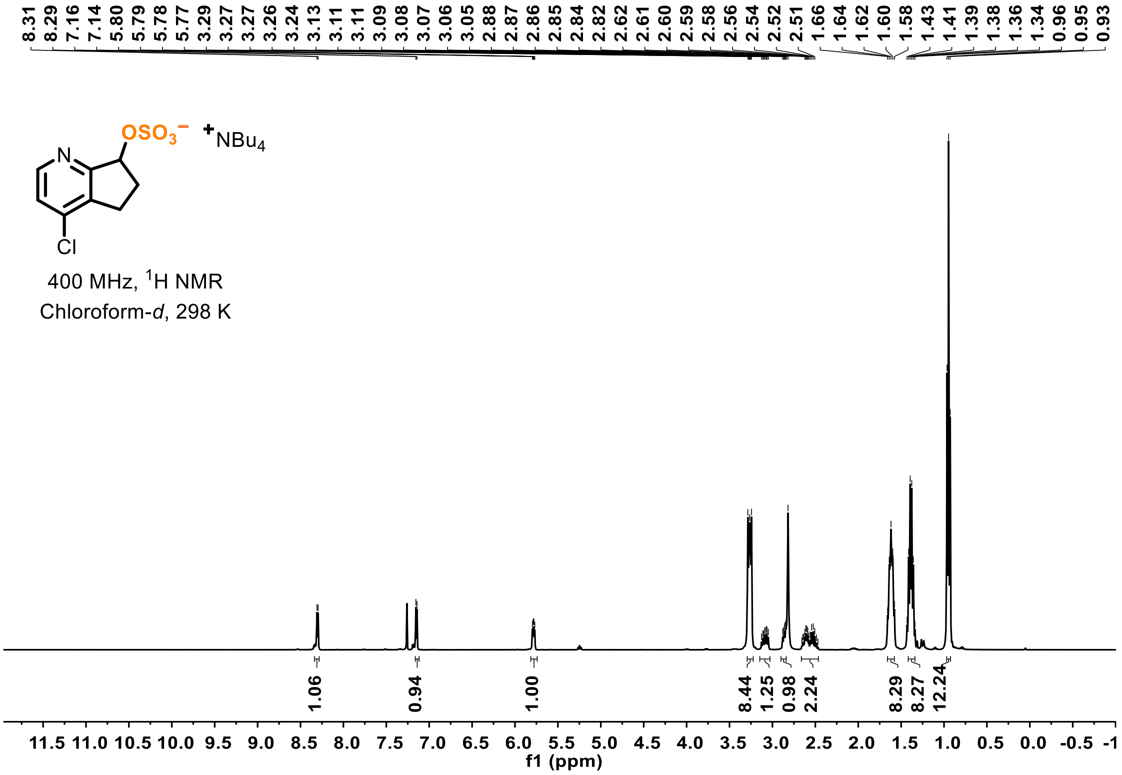

^{13}C NMR of tetrabutylammonium 4-chloro-6,7-dihydro-5H-[1]pyrindin-7-ol sulfate (29)

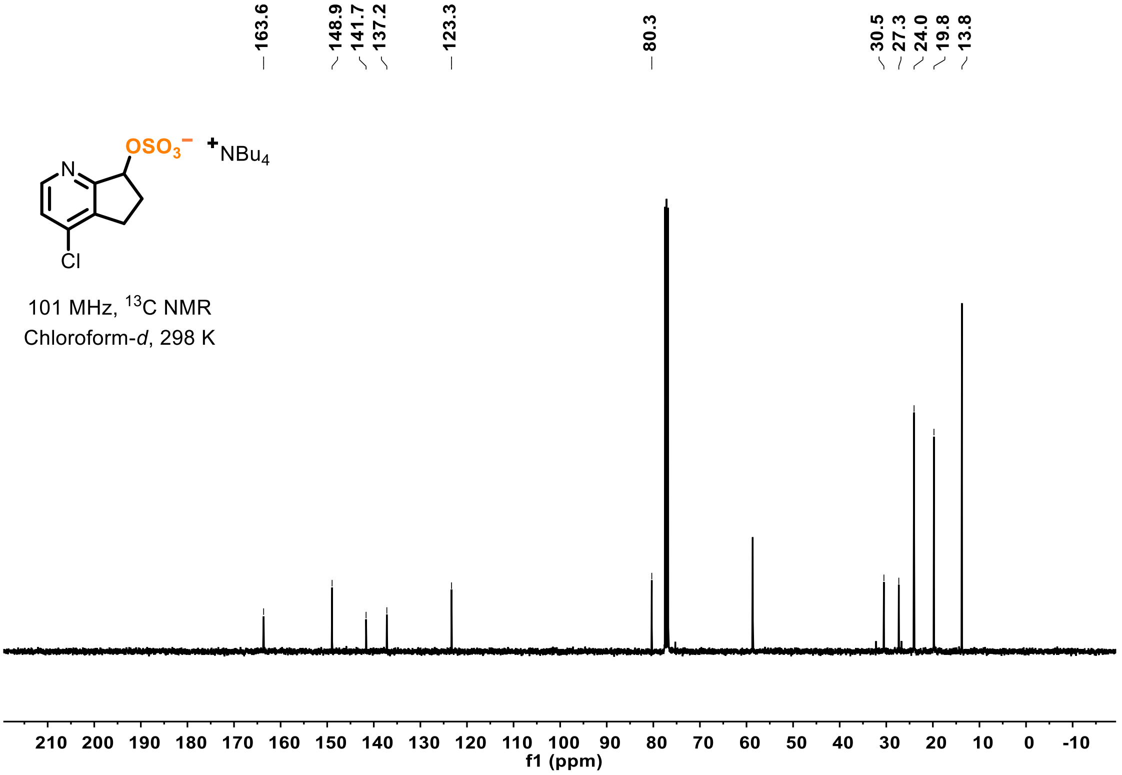

Tetrabutylammonium (1-hydroxycyclohexyl)phenyl-methanon sulfate (30)

^{1}H NMR of tetrabutylammonium (1-hydroxycyclohexyl)phenyl-methanon sulfate (30)

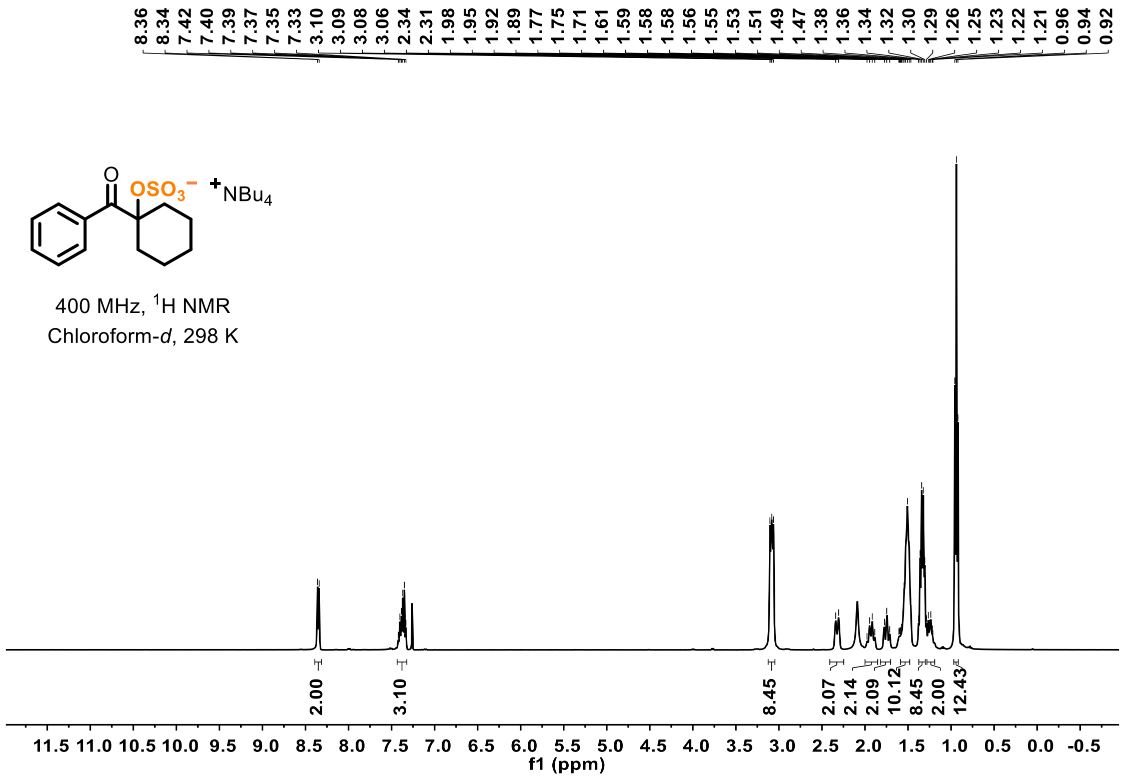

^{13}C NMR of tetrabutylammonium (1-hydroxycyclohexyl)phenyl-methanon sulfate (30)

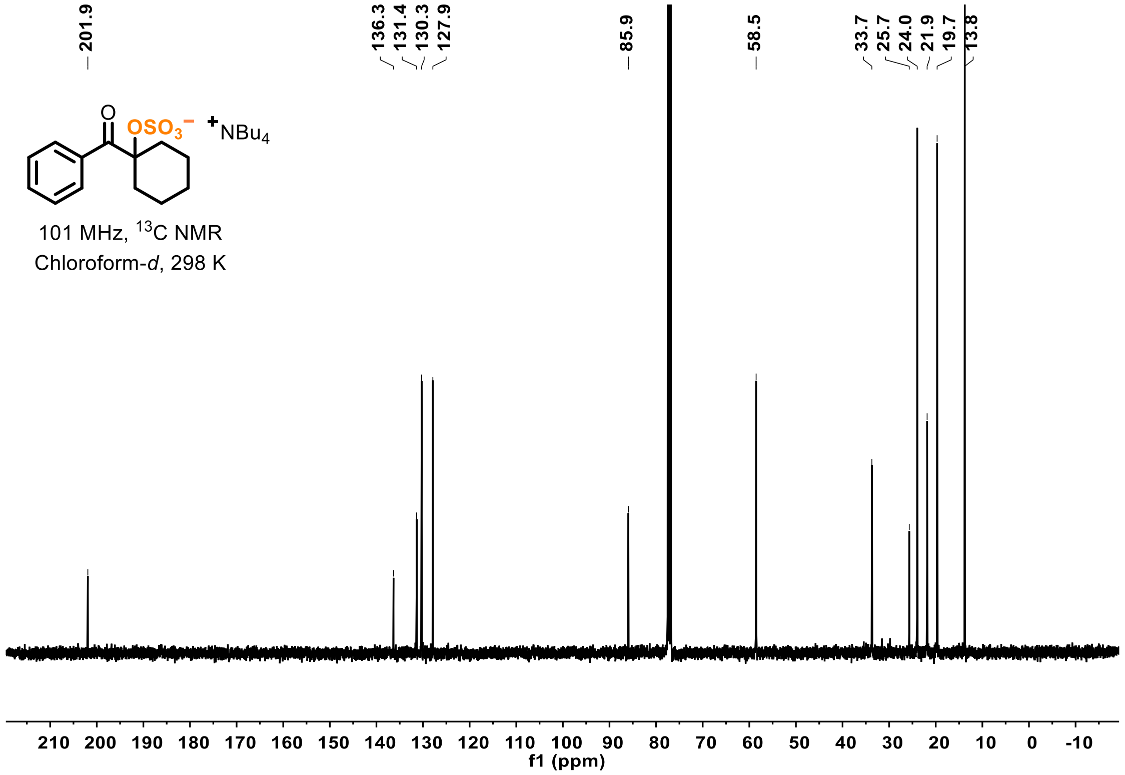

Tetrabutylammonium HOBt sulfate (31)

^1H NMR of tetrabutylammonium HOBt sulfate (31)

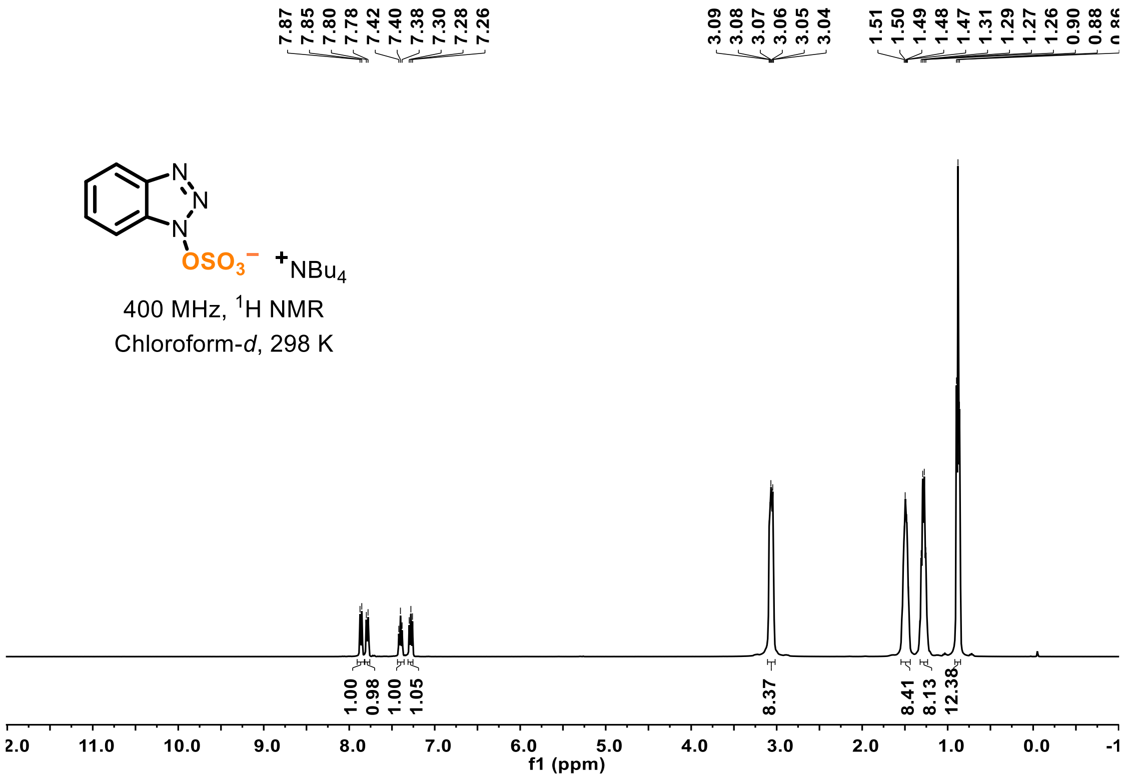

^{13}C NMR of tetrabutylammonium HOBt sulfate (31)

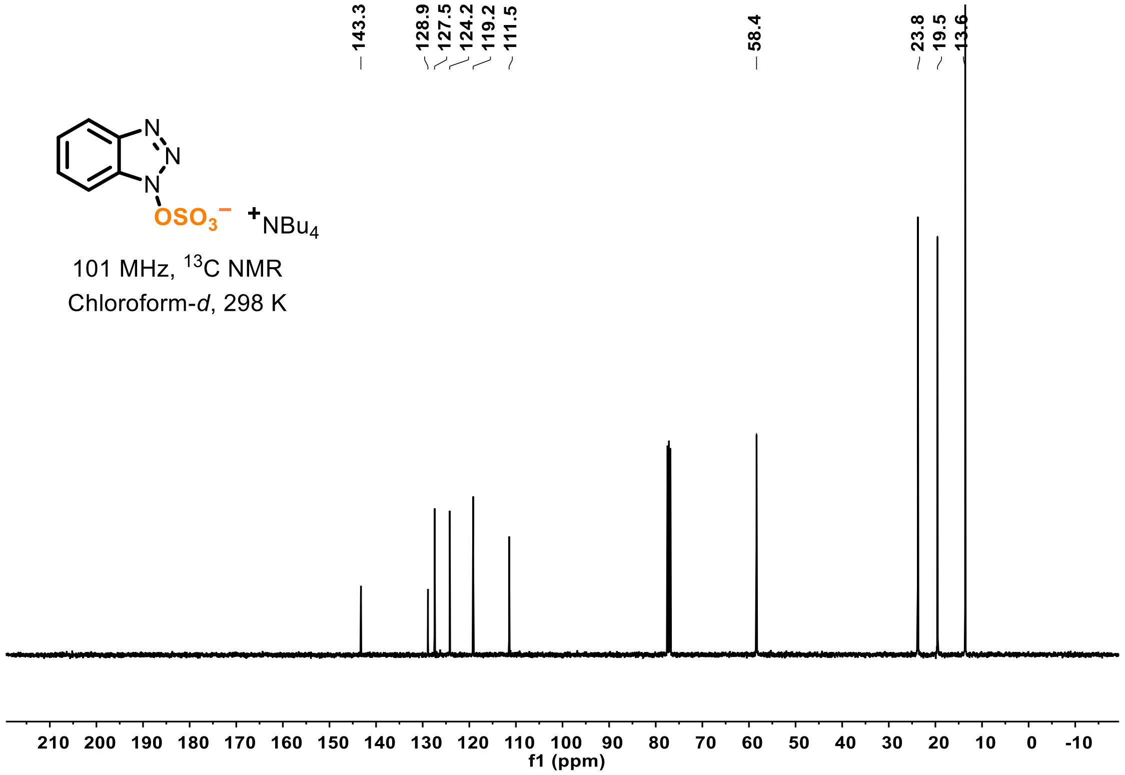

Tetrabutylammonium HOAt sulfate (32)

^{1}H NMR of tetrabutylammonium HOAt sulfate (32)

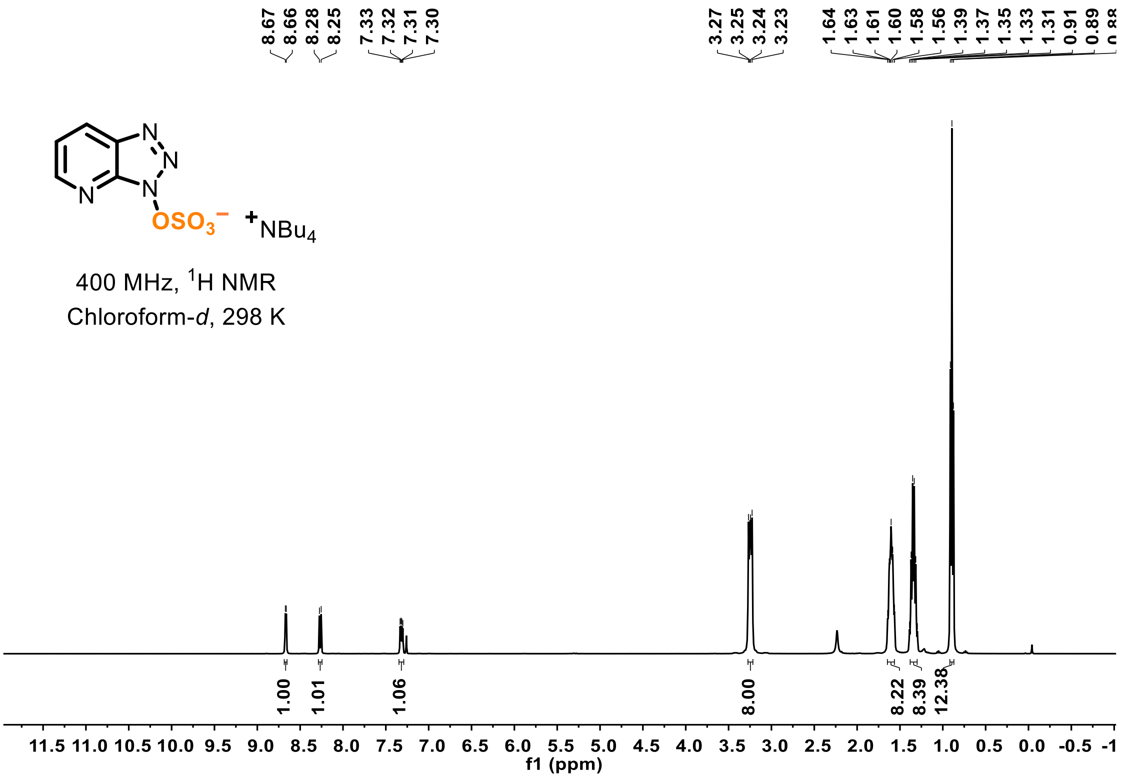

^{13}C NMR of tetrabutylammonium HOAt sulfate (32)

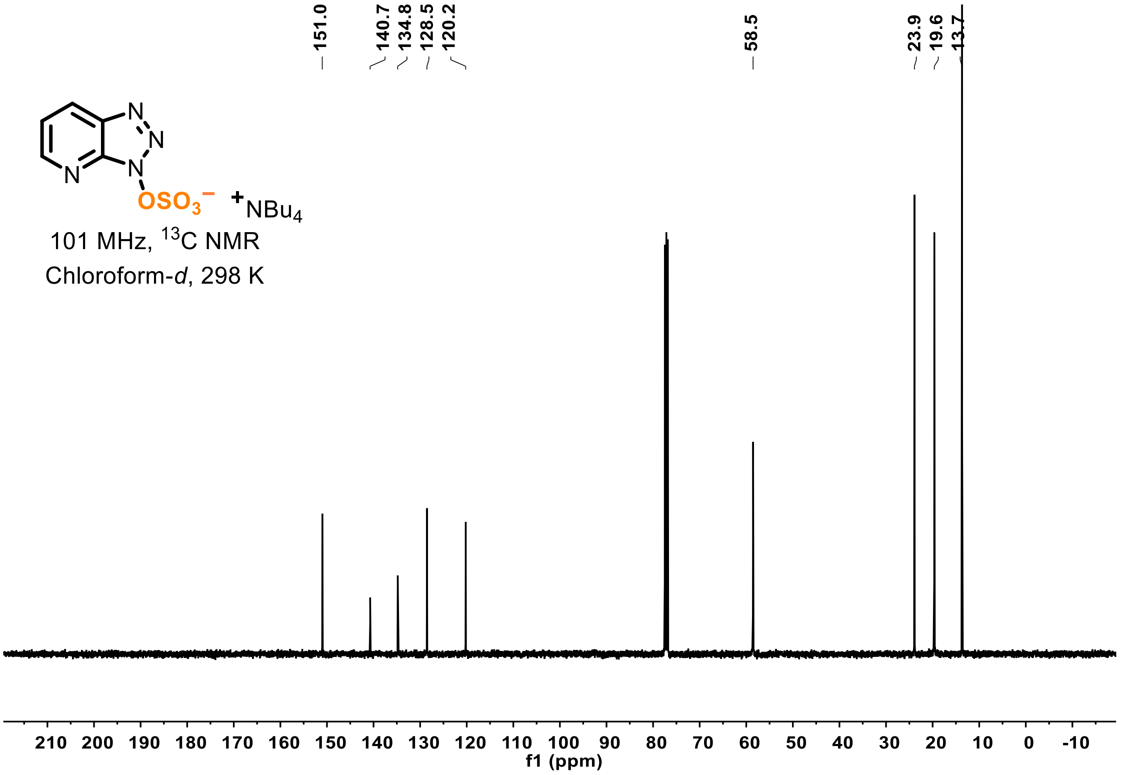

Tetrabutylammonium 4-phenylphenol sulfate (33)

^{1}H NMR of tetrabutylammonium 4-phenylphenol sulfate (33)

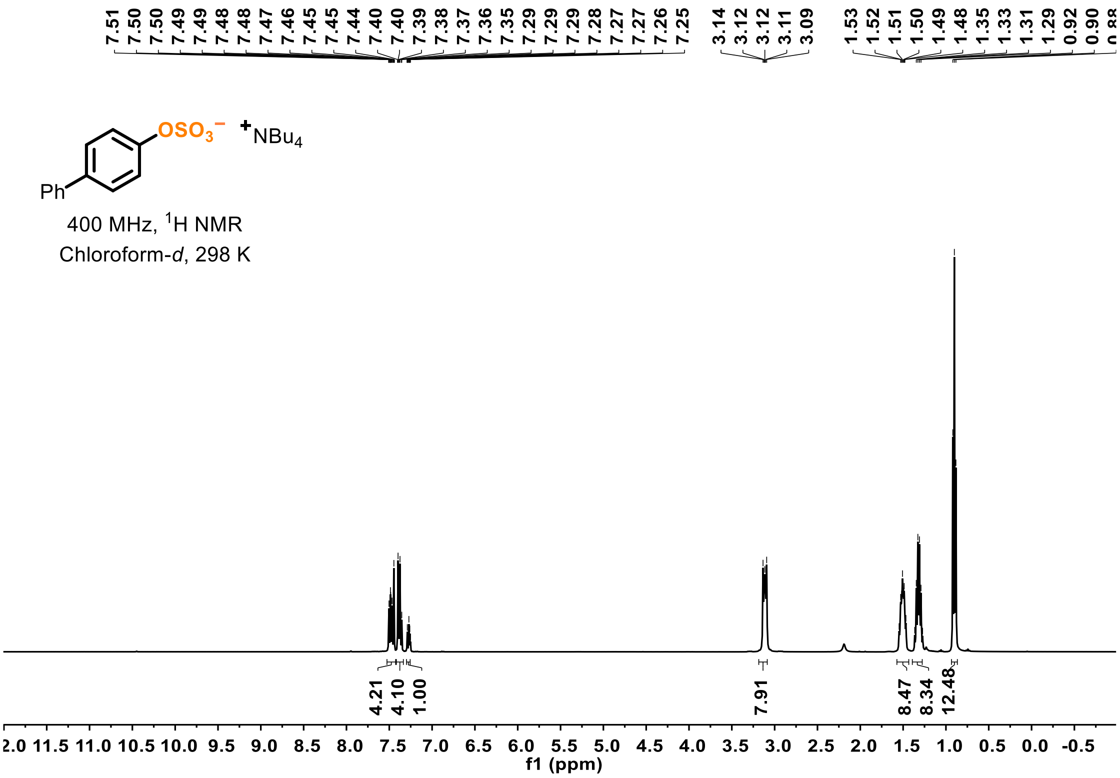

^{13}C NMR of tetrabutylammonium 4-phenylphenol sulfate (33)

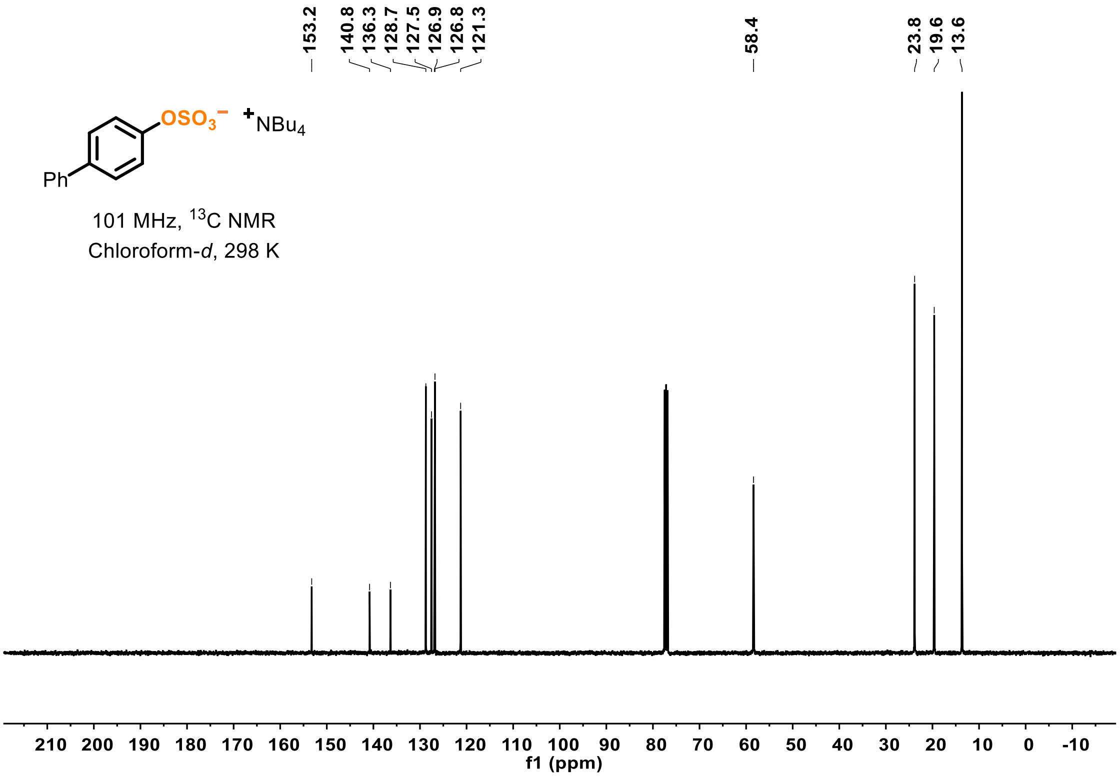

Tetrabutylammonium 1-naphthol sulfate (34)

^{1}H NMR of tetrabutylammonium 1-naphthol sulfate (34)

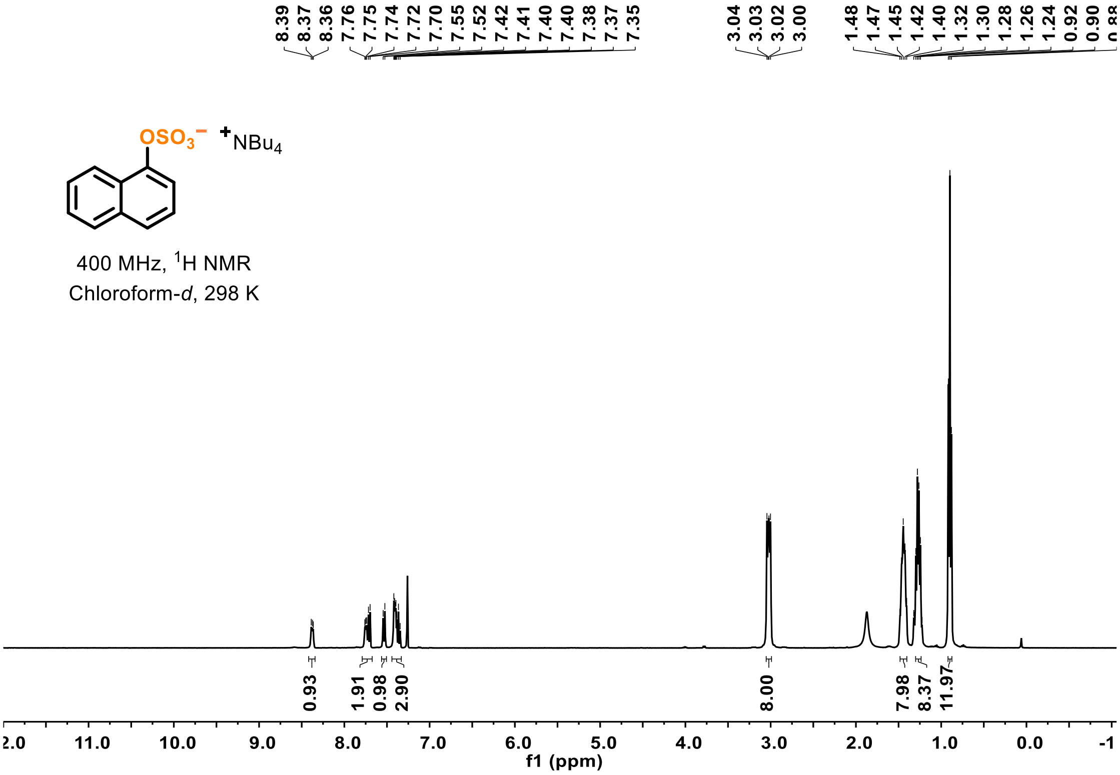

^{13}C NMR of tetrabutylammonium 1-naphthol sulfate (34)

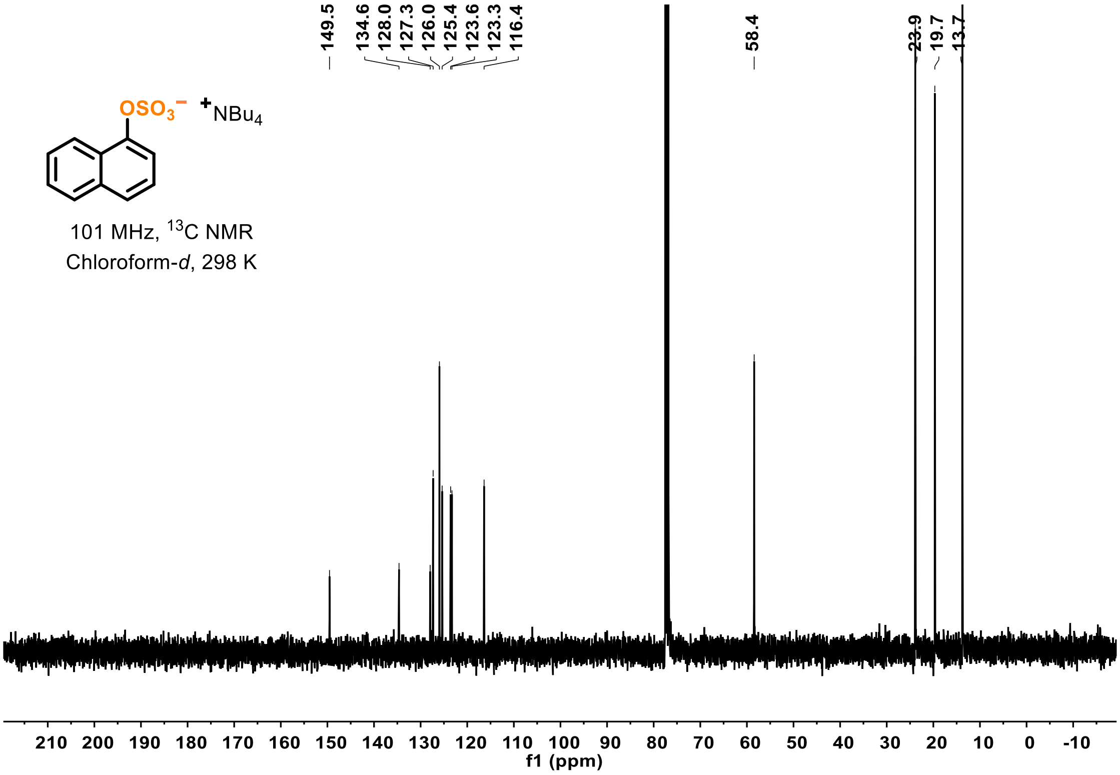

Tetrabutylammonium 4-ethylphenol sulfate (35)

^{1}H NMR of tetrabutylammonium 4-ethylphenol sulfate (35)

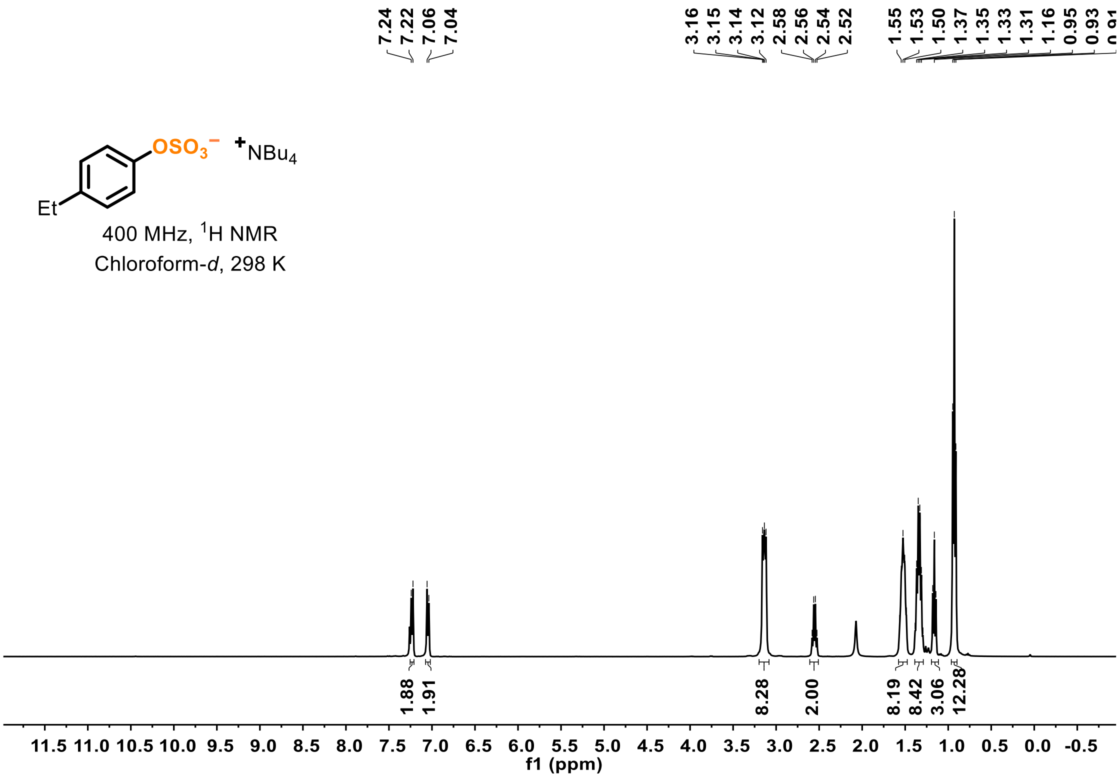

^{13}C NMR of tetrabutylammonium 4-ethylphenol sulfate (35)

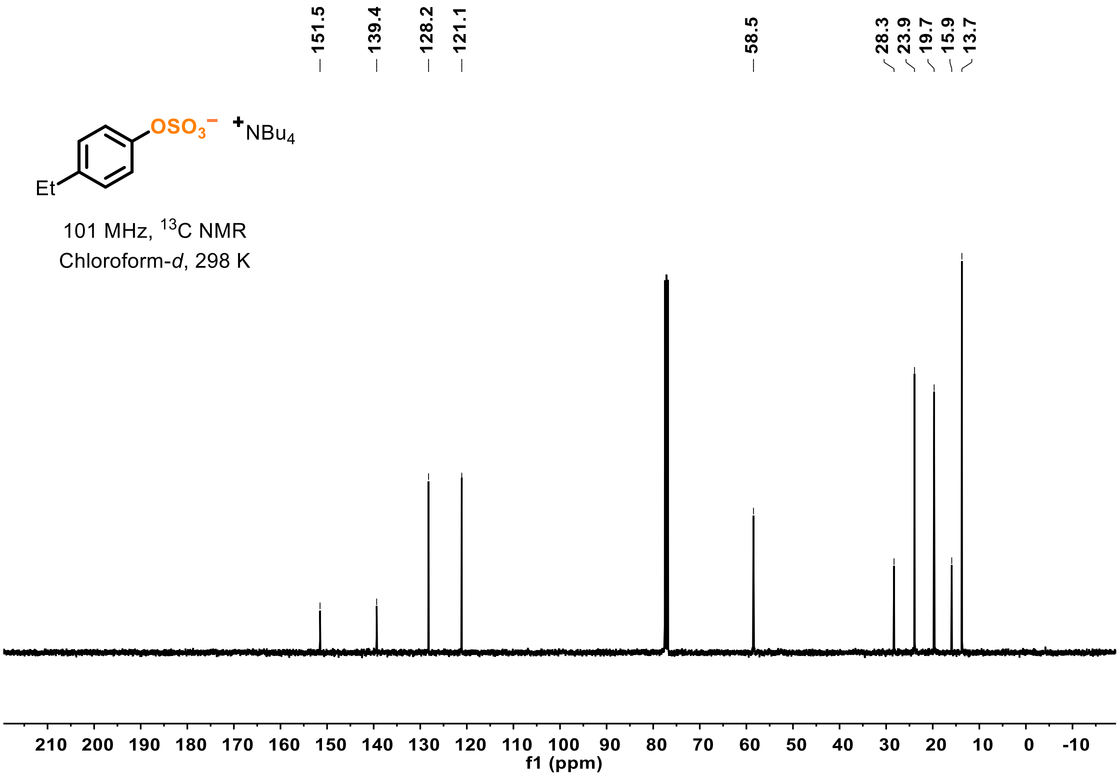

Tetrabutylammonium 4-(tert-butyl)phenol sulfate (36)

^{1}H NMR of tetrabutylammonium 4-(tert-butyl)phenol sulfate (36)

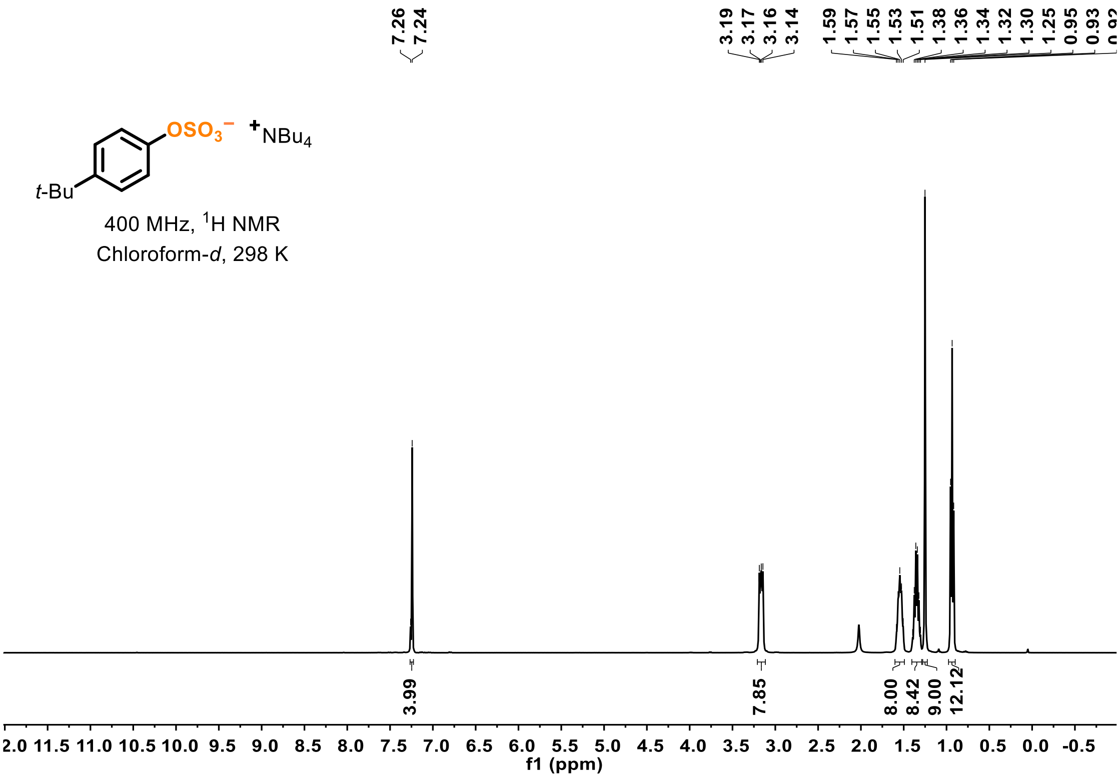

^{13}C NMR of tetrabutylammonium 4-(tert-butyl)phenol sulfate (36)

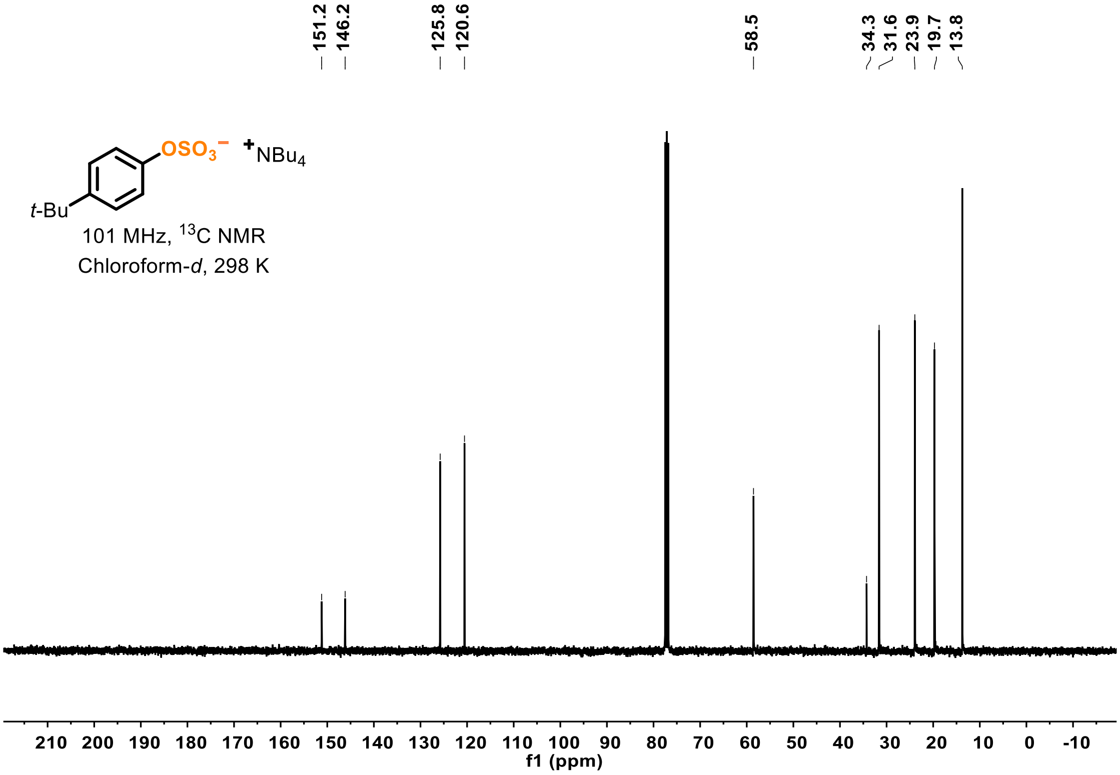

Tetrabutylammonium 4-(trifluoromethyl)phenol sulfate (37)

^{1}H NMR of tetrabutylammonium 4-(trifluoromethyl)phenol sulfate (37)

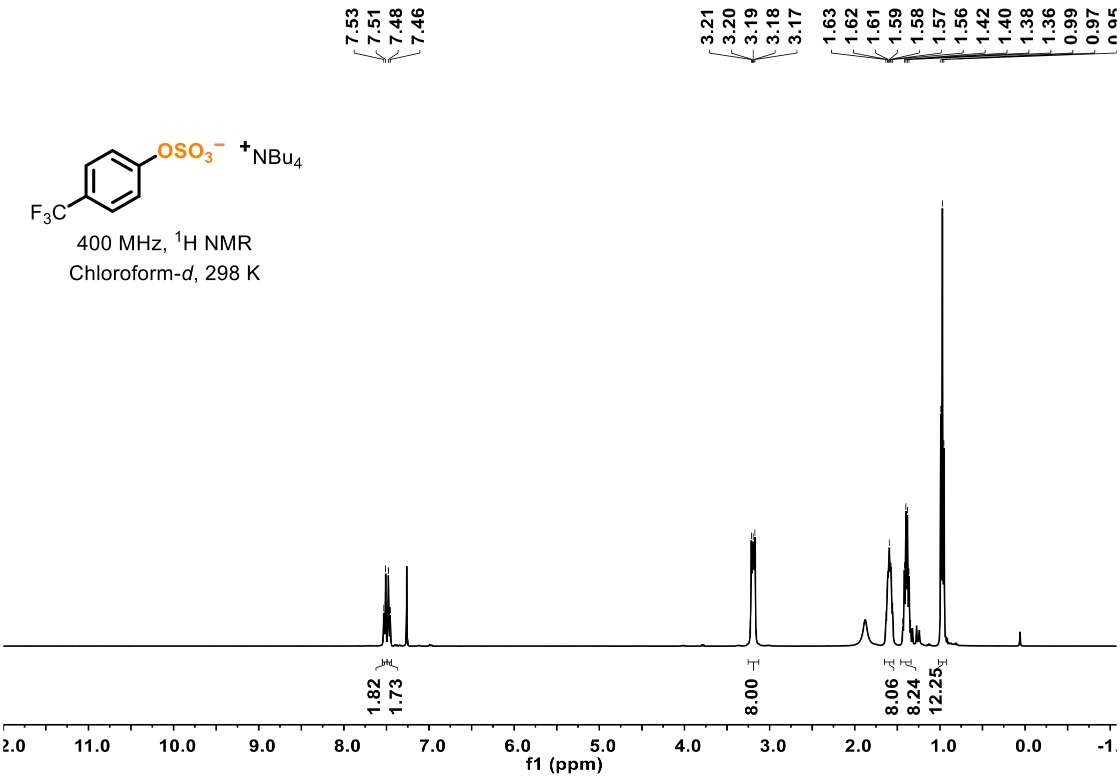

^{13}C NMR of tetrabutylammonium 4-(trifluoromethyl)phenol sulfate (37)

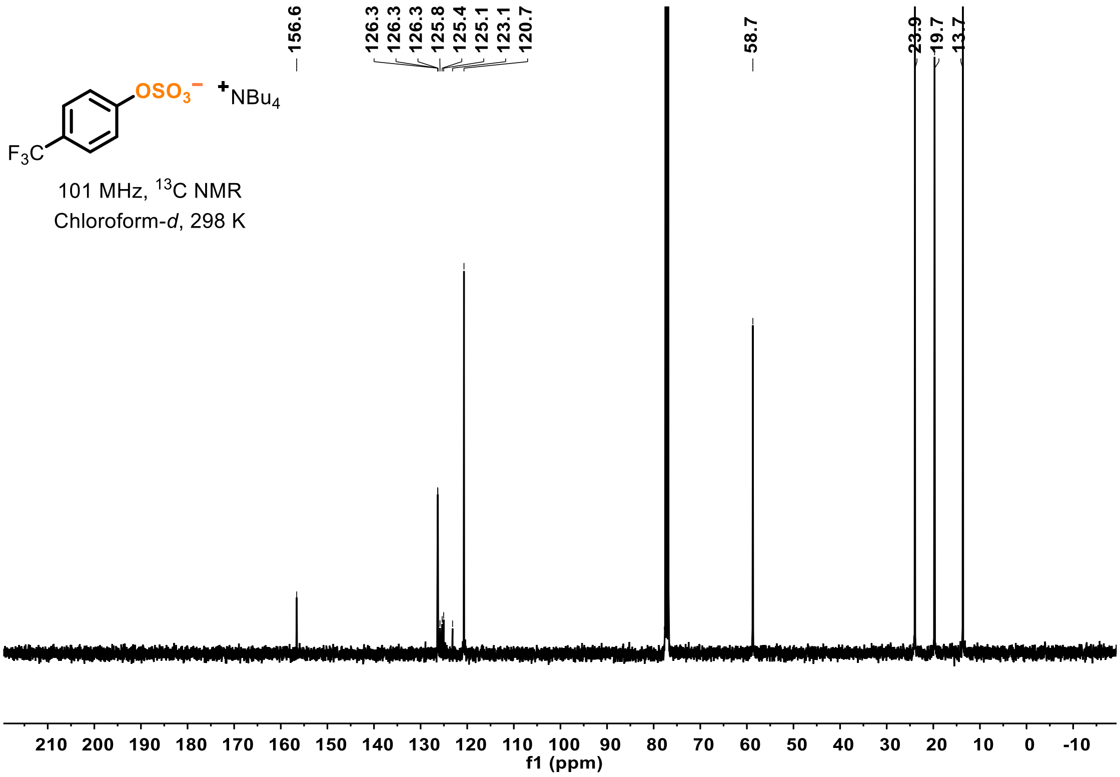

^{19}F NMR of tetrabutylammonium 4-(trifluoromethyl)phenol sulfate (37)

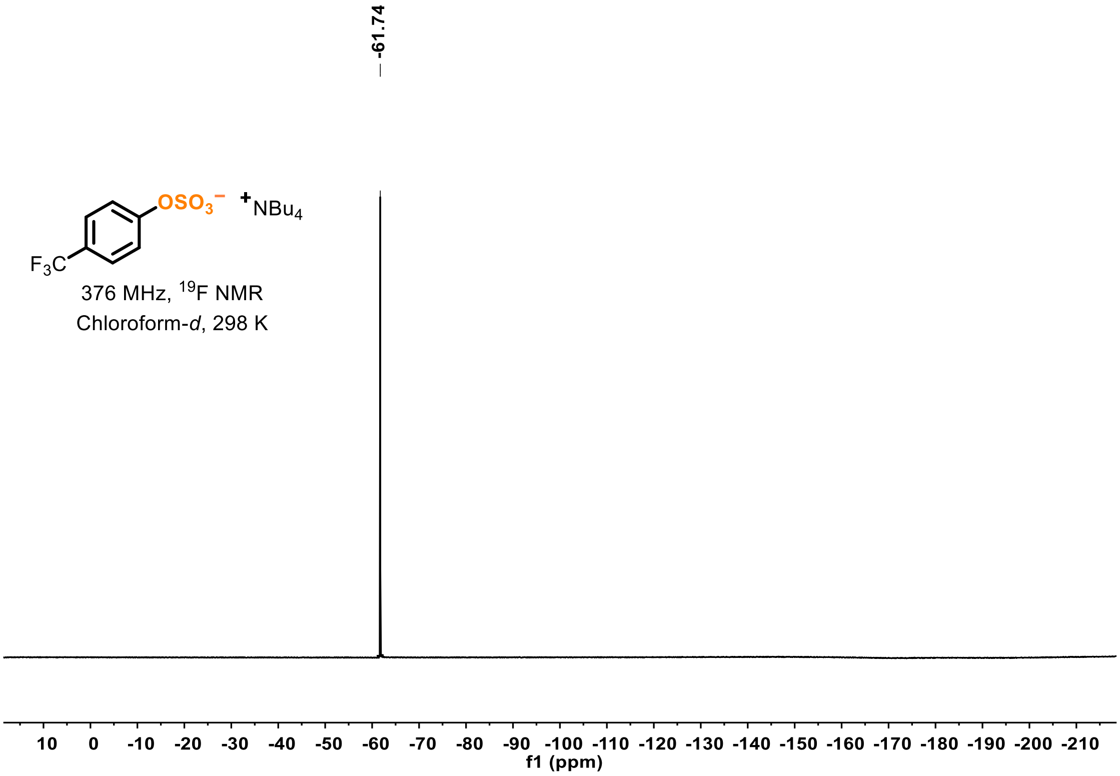

Tetrabutylammonium 4-cyanophenol sulfate (38)

^{1}H NMR of tetrabutylammonium 4-cyanophenol sulfate (38)

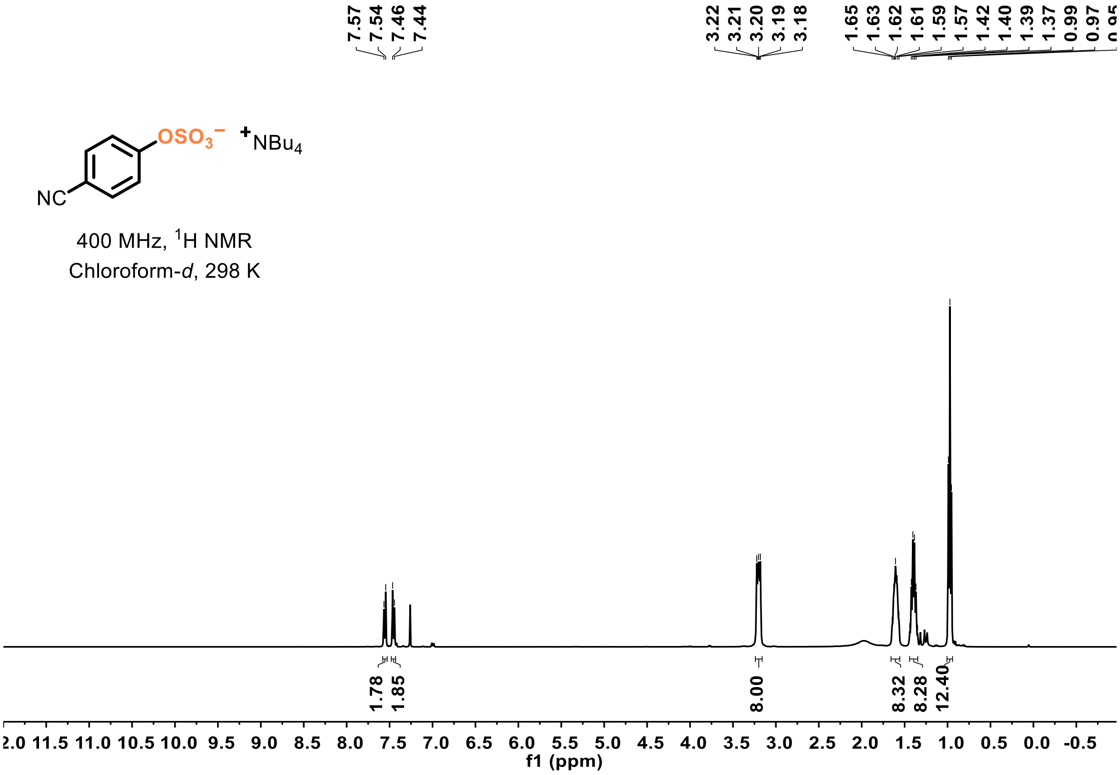

^{13}C NMR of tetrabutylammonium 4-cyanophenol sulfate (38)

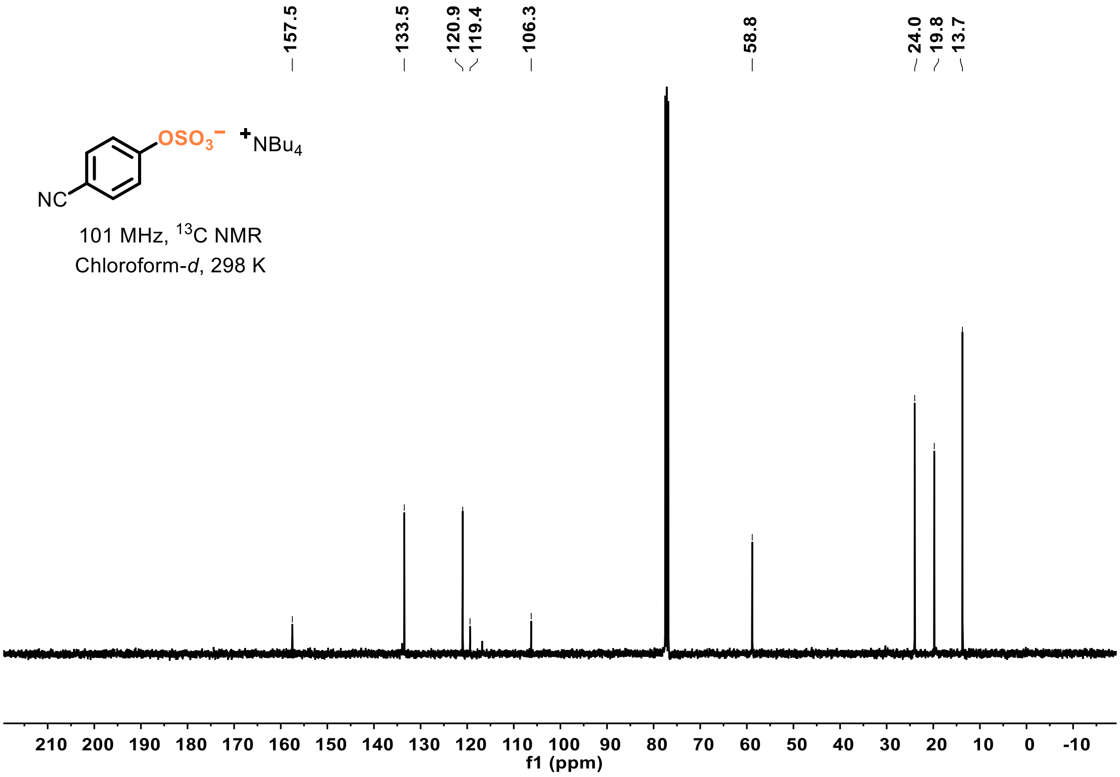

Tetrabutylammonium 4-acetophenol sulfate (39)

^{1}H NMR of tetrabutylammonium 4-acetophenol sulfate (39)

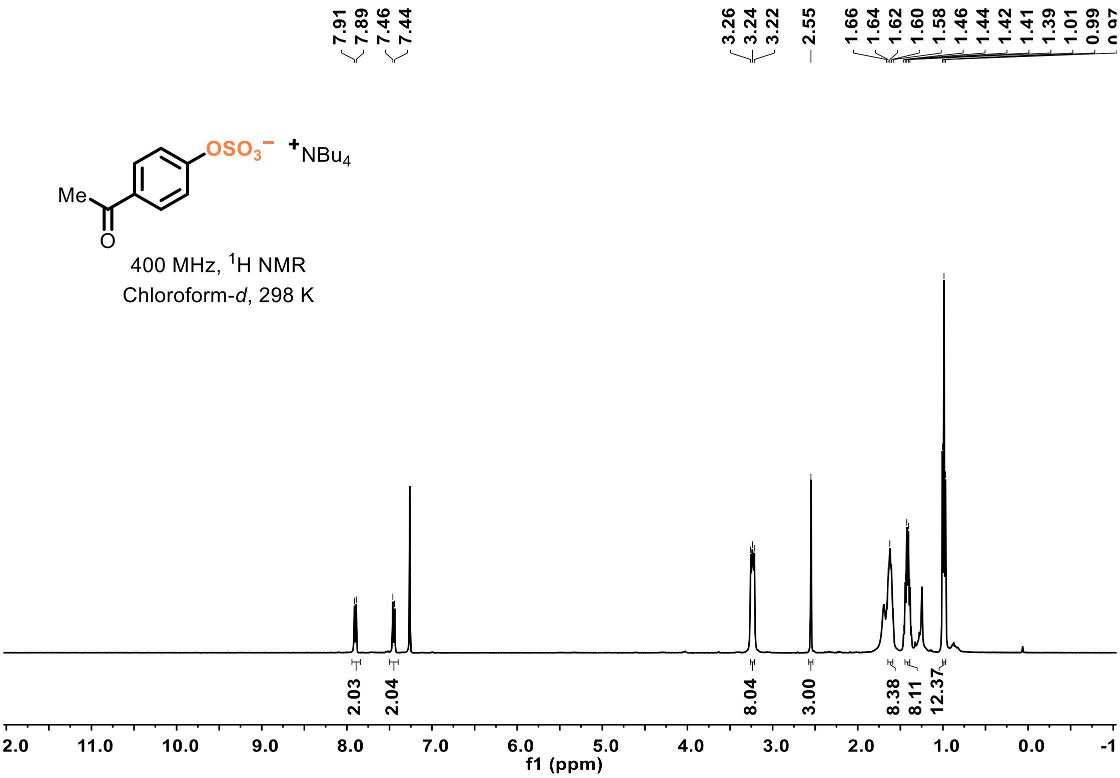

^{13}C NMR of tetrabutylammonium 4-acetophenol sulfate (39)

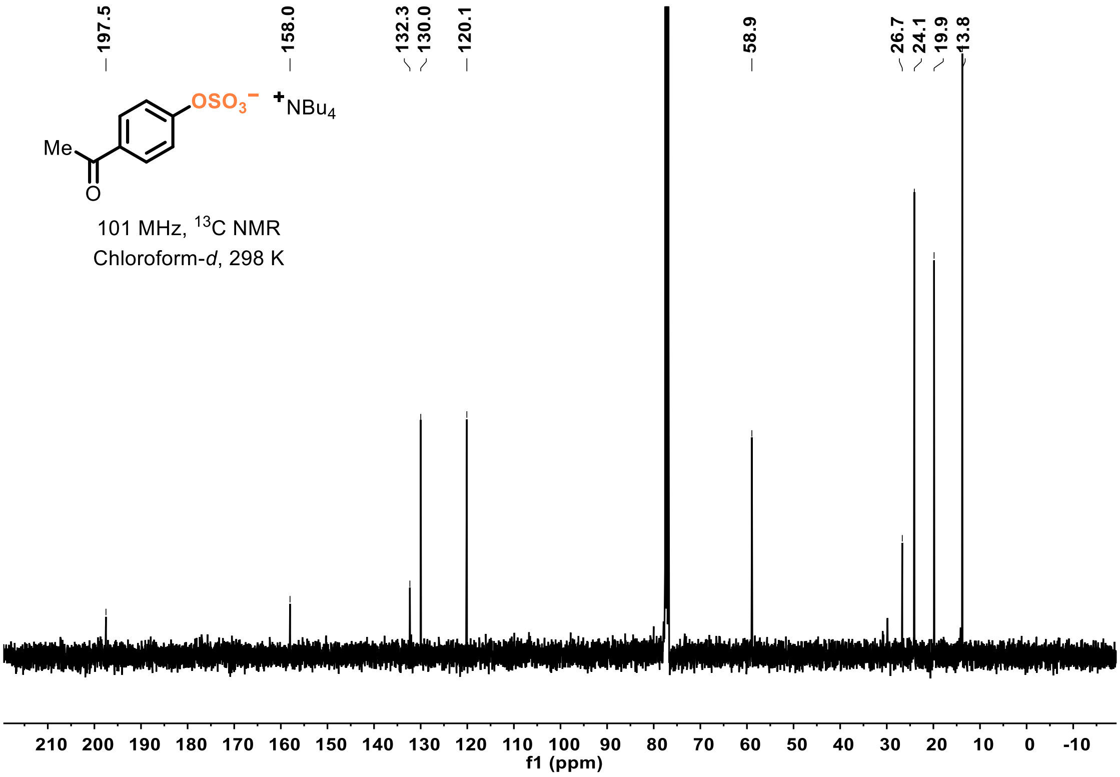

Tetrabutylammonium 2-iodophenol sulfate (40)

^{1}H NMR of tetrabutylammonium 2-iodophenol sulfate (40)

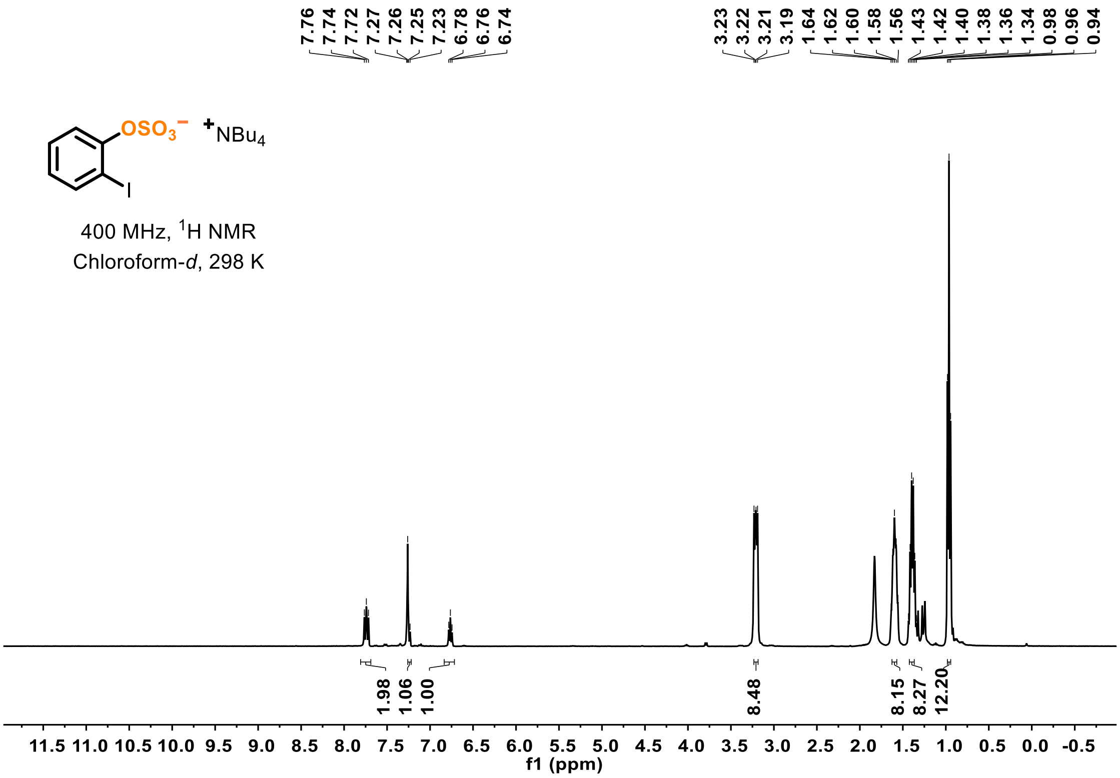

^{13}C NMR of tetrabutylammonium 2-iodophenol sulfate (40)

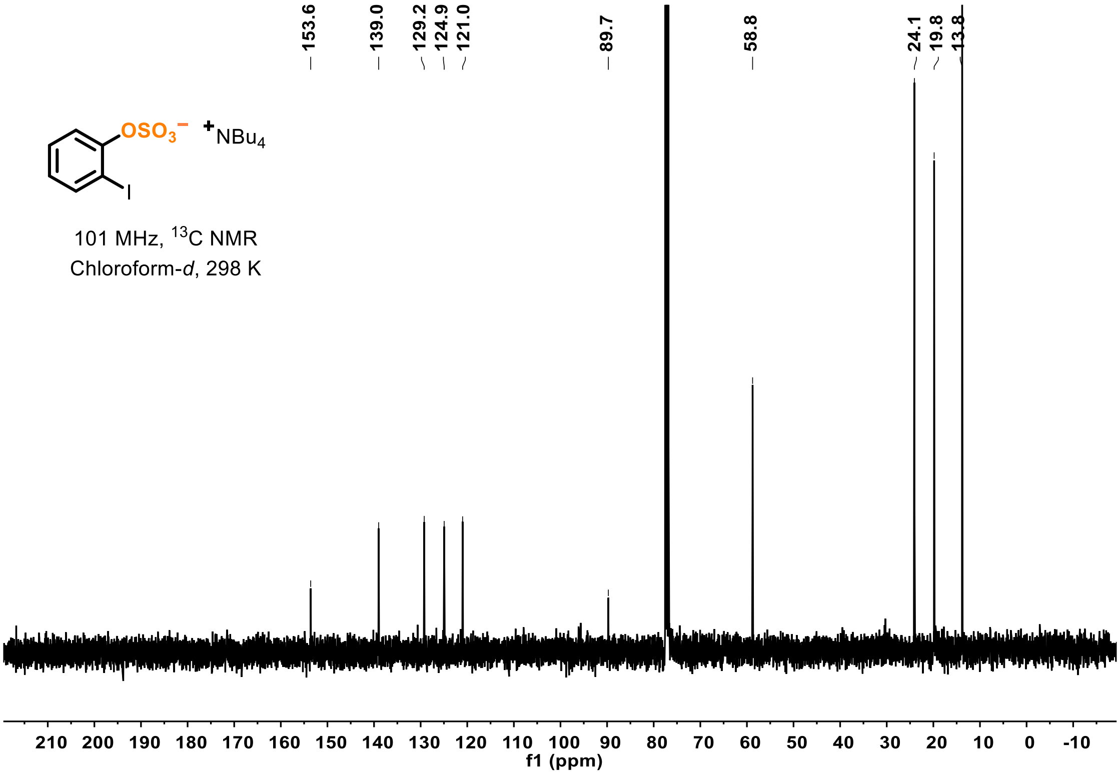

Tetrabutylammonium 2-phenylphenol sulfate (41)

^{1}H NMR of tetrabutylammonium 2-phenylphenol sulfate (41)

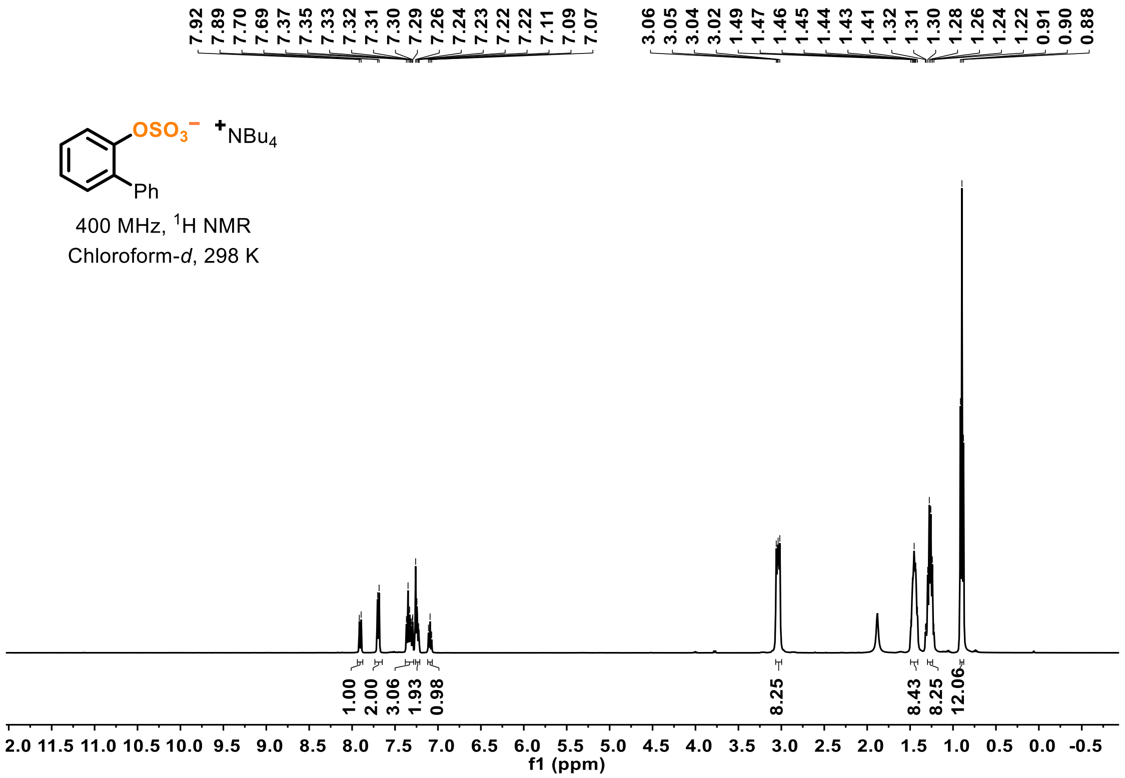

^{13}C NMR of tetrabutylammonium 2-phenylphenol sulfate (41)

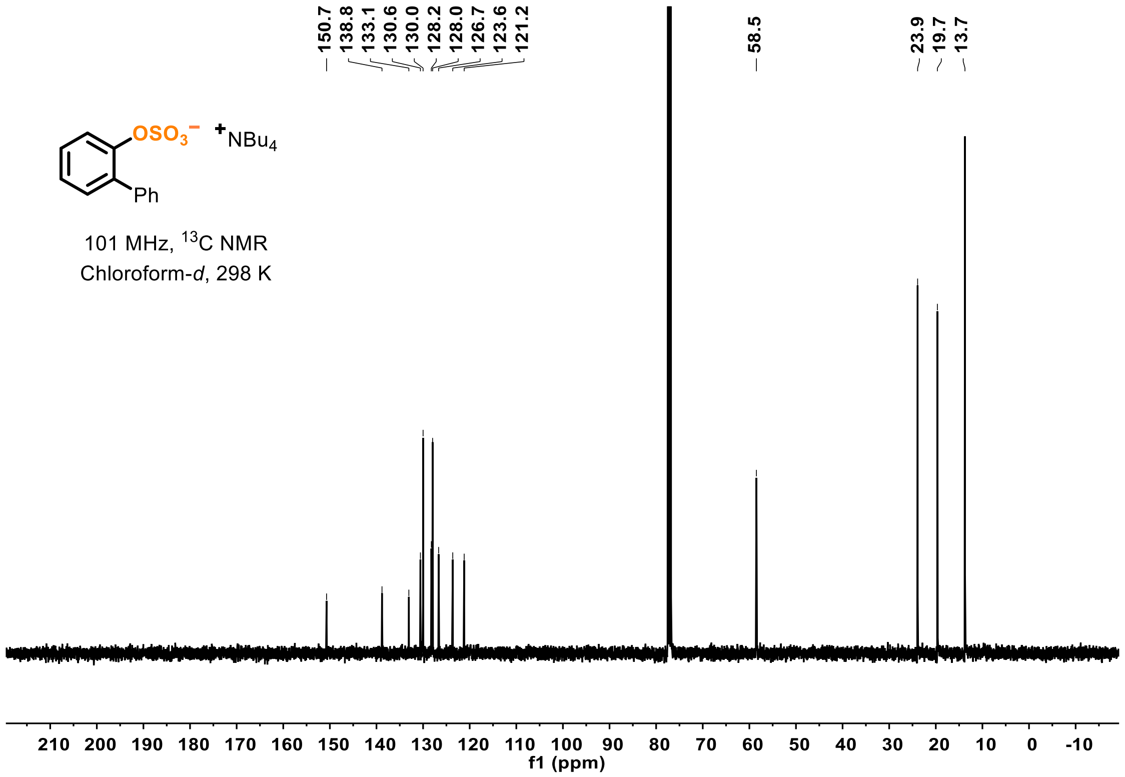

Tetrabutylammonium 3-methoxyphenol sulfate (42)

^{1}H NMR of tetrabutylammonium 3-methoxyphenol sulfate (42)

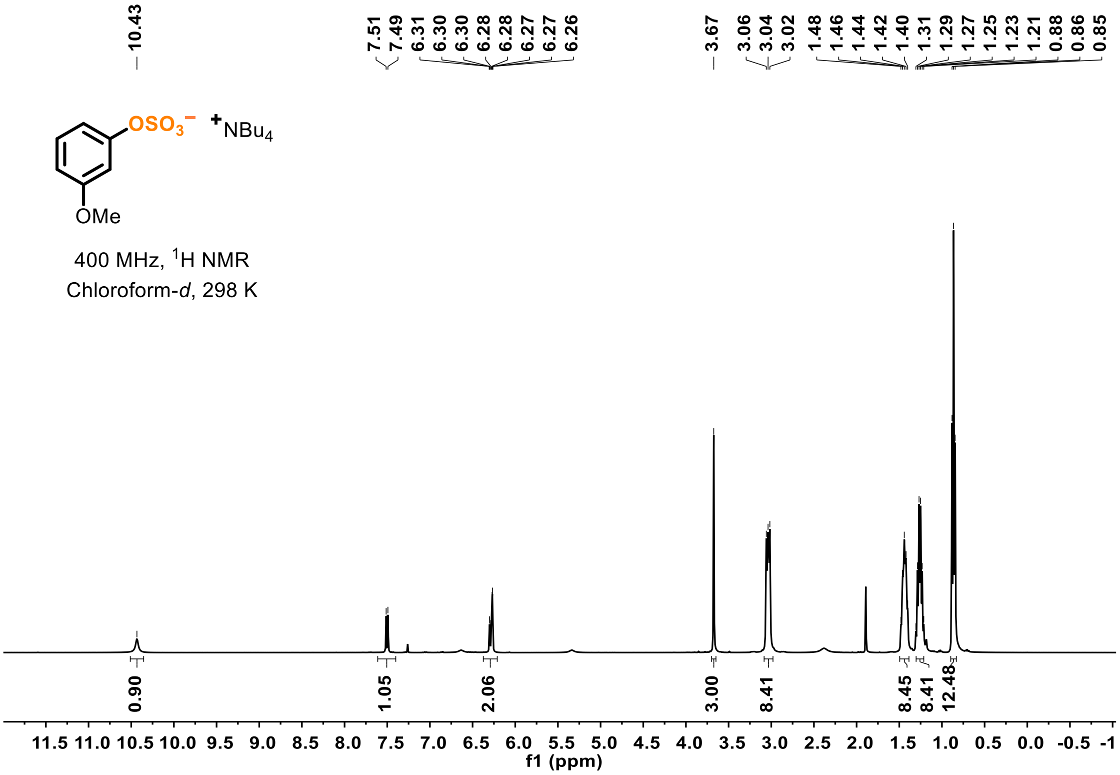

^{13}C NMR of tetrabutylammonium 3-methoxyphenol sulfate (42)

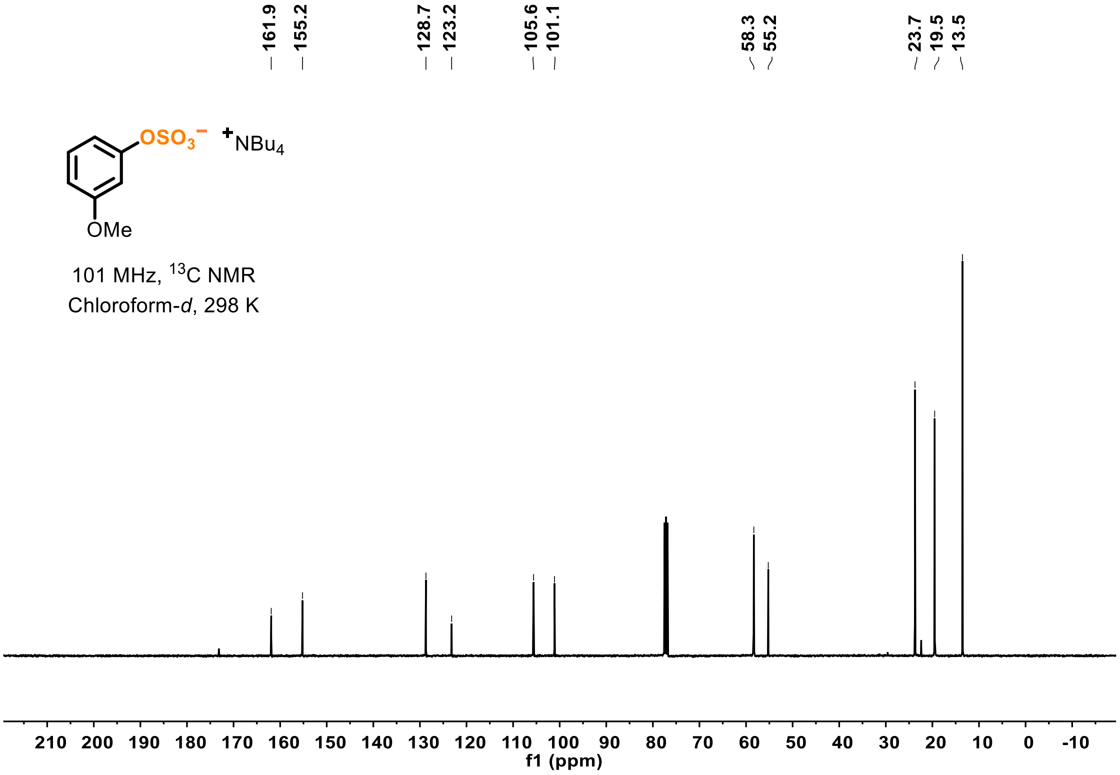

Tetrabutylammonium methyl 3-hydroxybenzoate sulfate (43)

^{1}H NMR of tetrabutylammonium methyl 3-hydroxybenzoate sulfate (43)

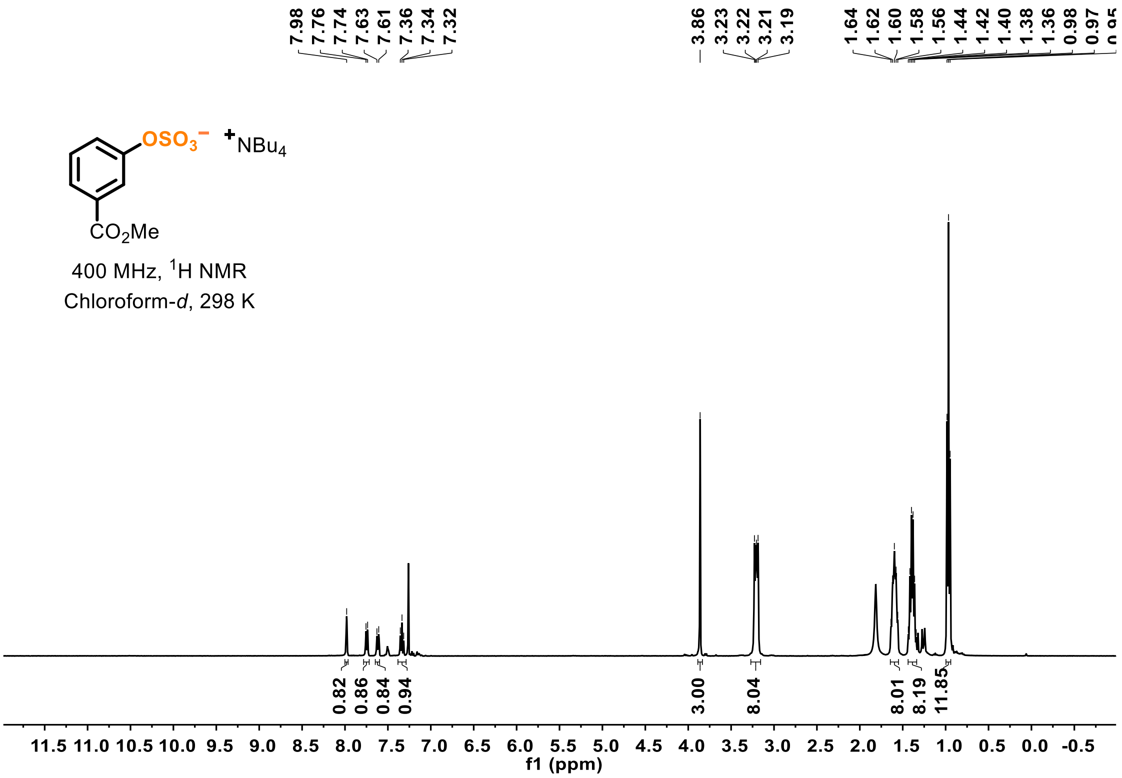

^{13}C NMR of tetrabutylammonium methyl 3-hydroxybenzoate sulfate (43)

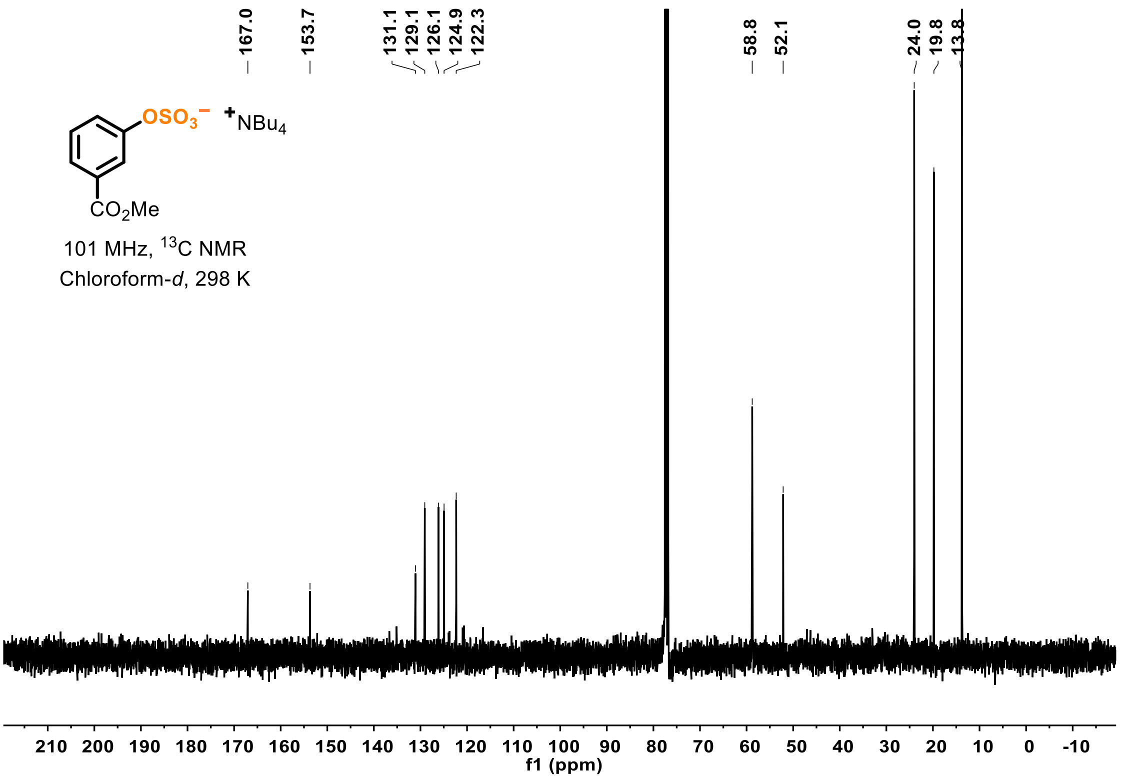

Tetrabutylammonium 4-chloro-3-ethylphenol sulfate (44)

^{1}H NMR of tetrabutylammonium 4-chloro-3-ethylphenol sulfate (44)

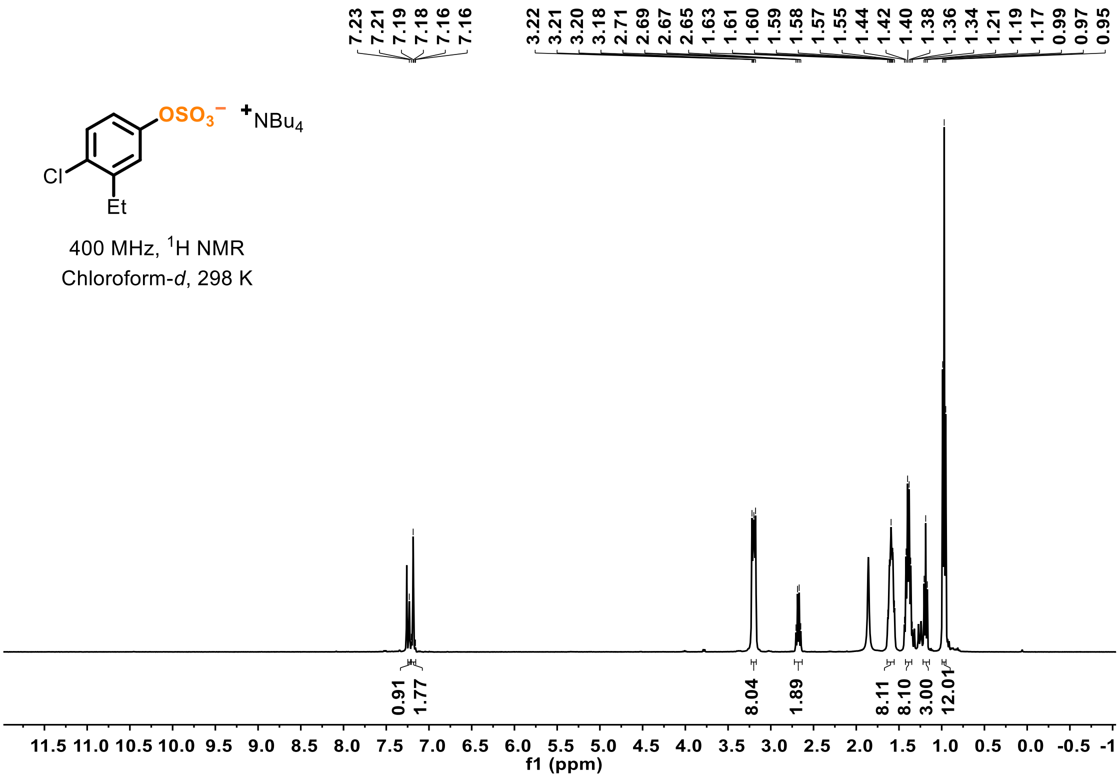

^{13}C NMR of tetrabutylammonium 4-chloro-3-ethylphenol sulfate (44)

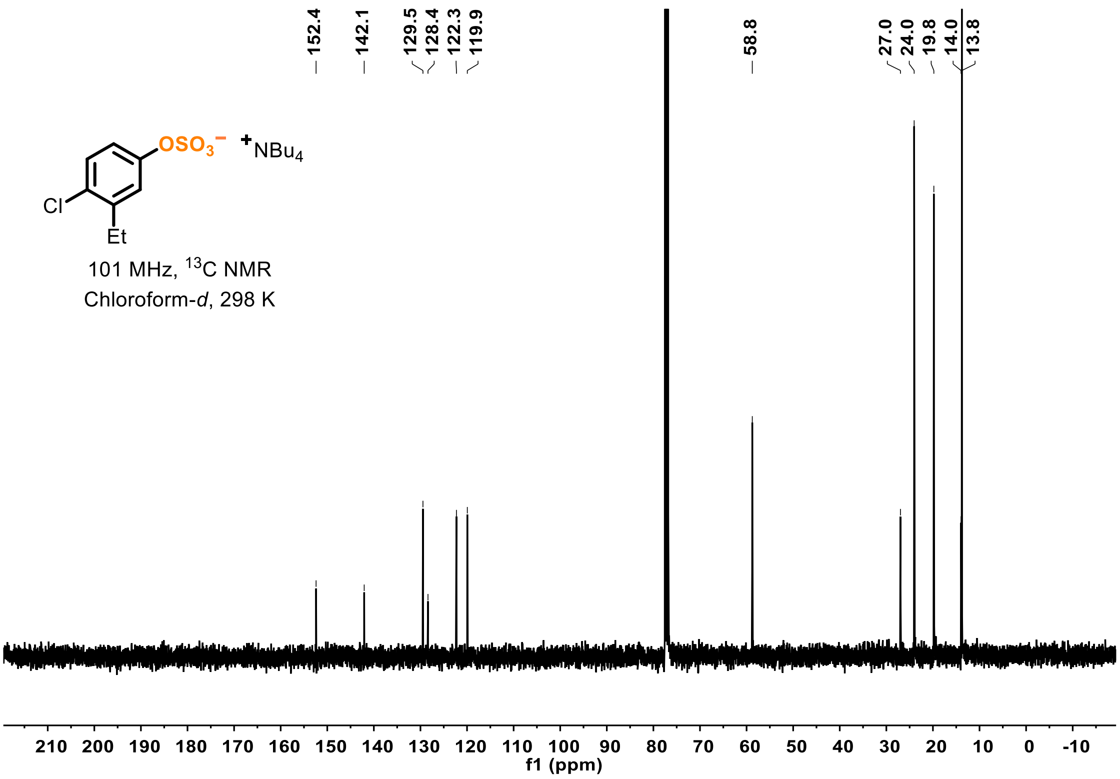

Tetrabutylammonium 4-methyl-benzenethio sulfate (45)

^{1}H NMR of tetrabutylammonium 4-methyl-benzenethio sulfate (45)

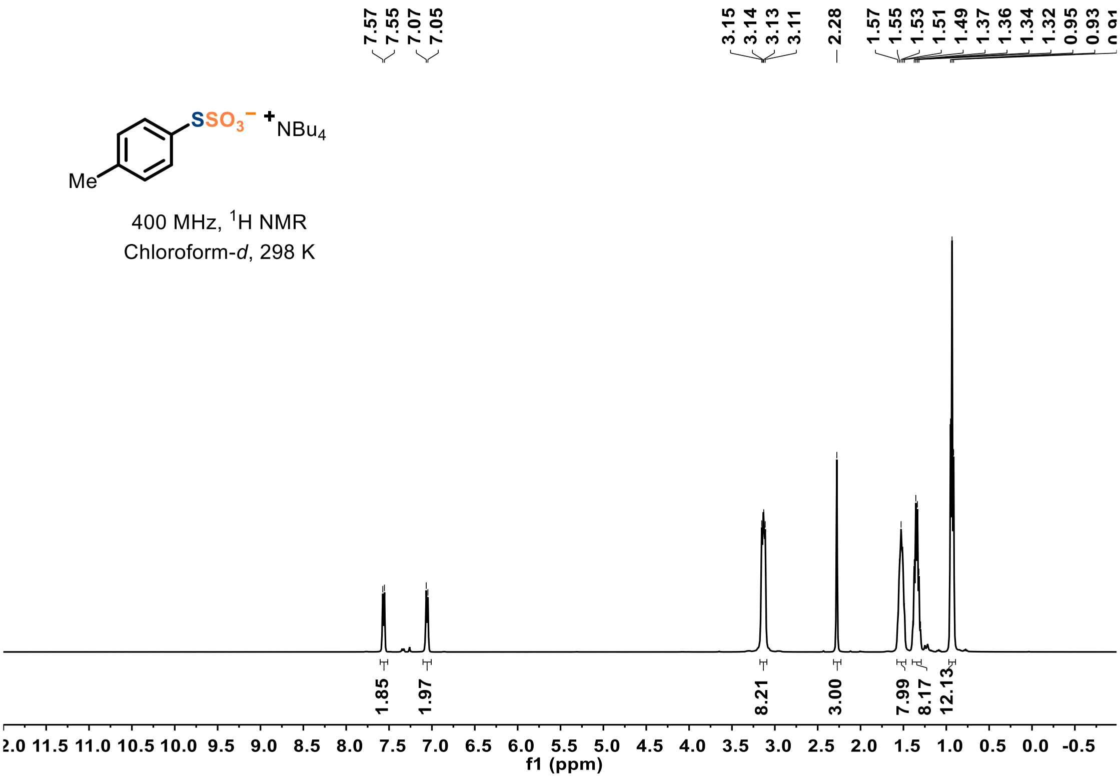

^{13}C NMR of tetrabutylammonium 4-methyl-benzenethio sulfate (45)

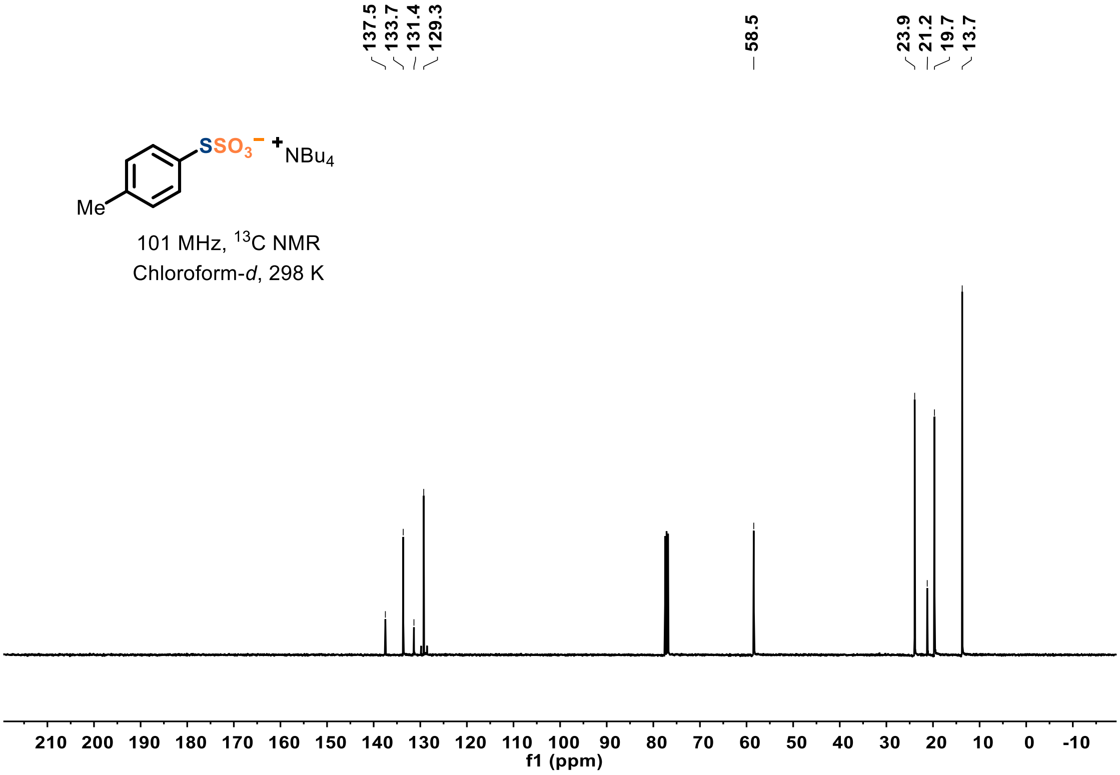

Tetrabutylammonium saccharide derivative sulfate (46)

^{1}H NMR of tetrabutylammonium saccharide derivative sulfate (46)

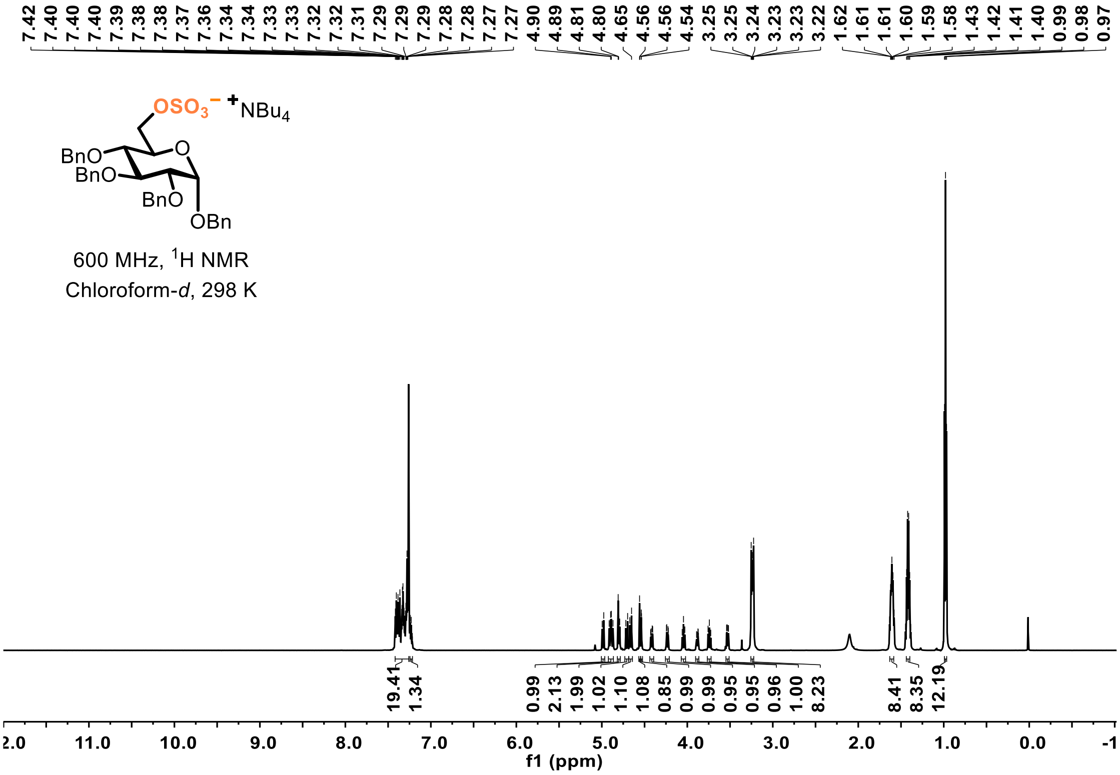

^{13}C NMR of tetrabutylammonium saccharide derivative sulfate (46)

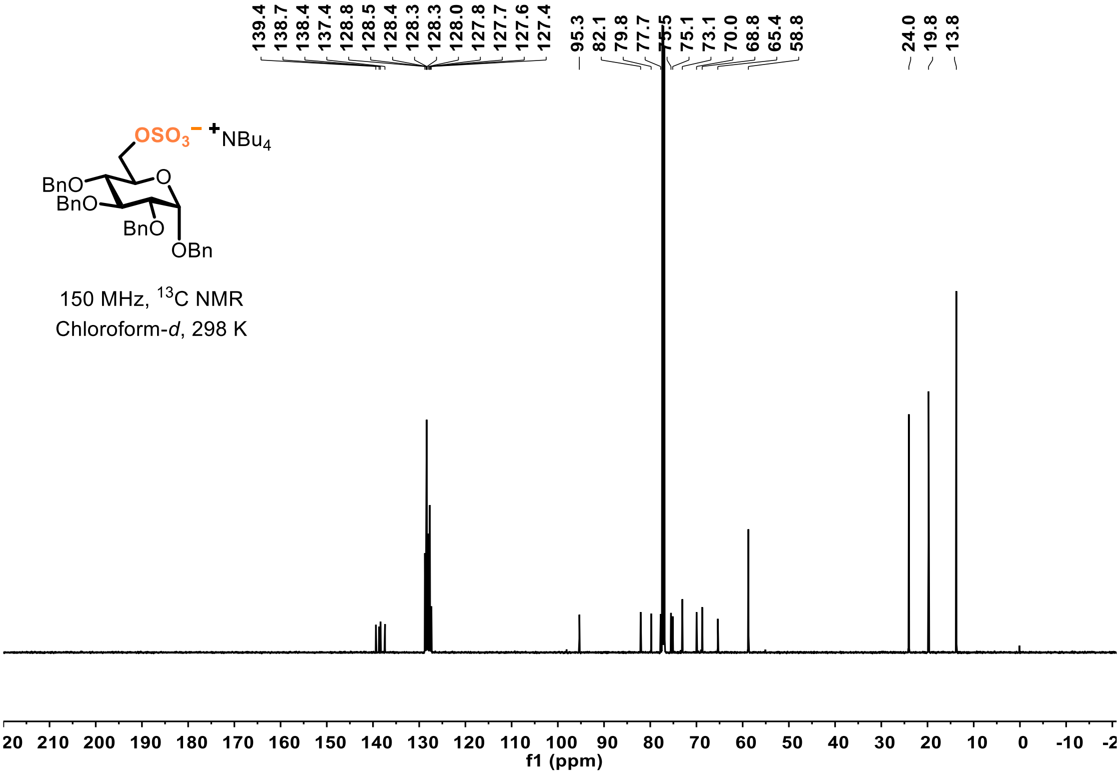

Tetrabutylammonium saccharide derivative sulfate (47)

^{1}H NMR of tetrabutylammonium saccharide derivative sulfate (47)

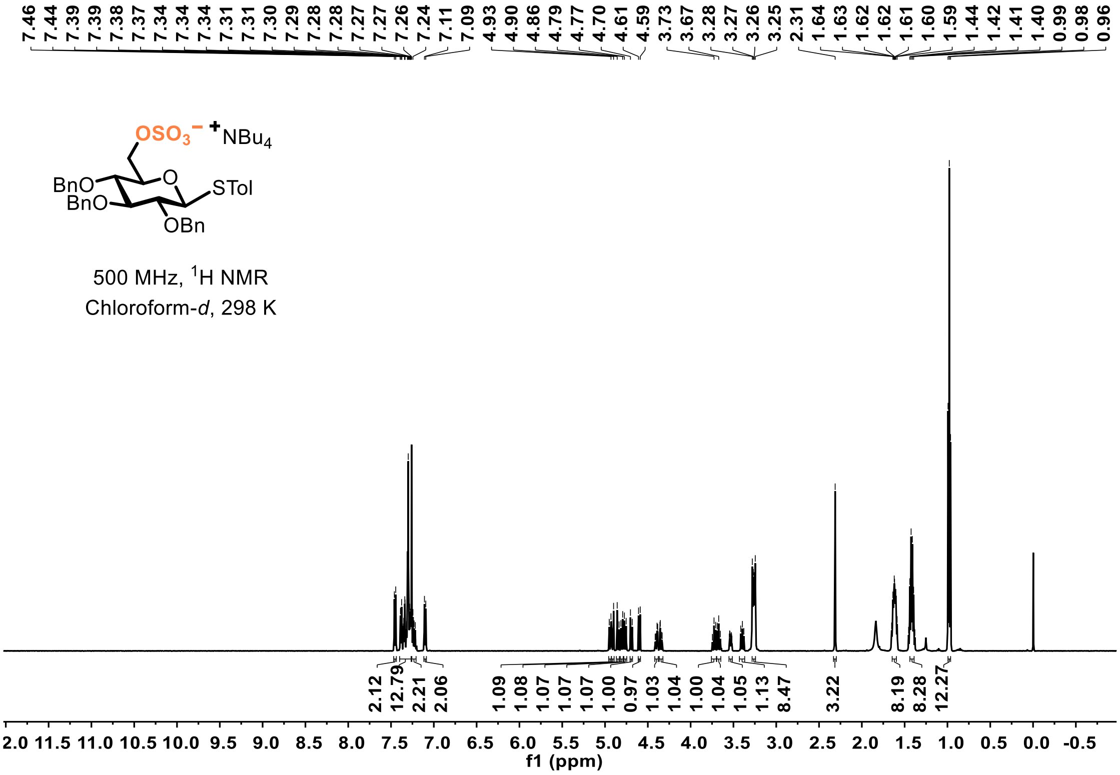

^{13}C NMR of tetrabutylammonium saccharide derivative sulfate (47)

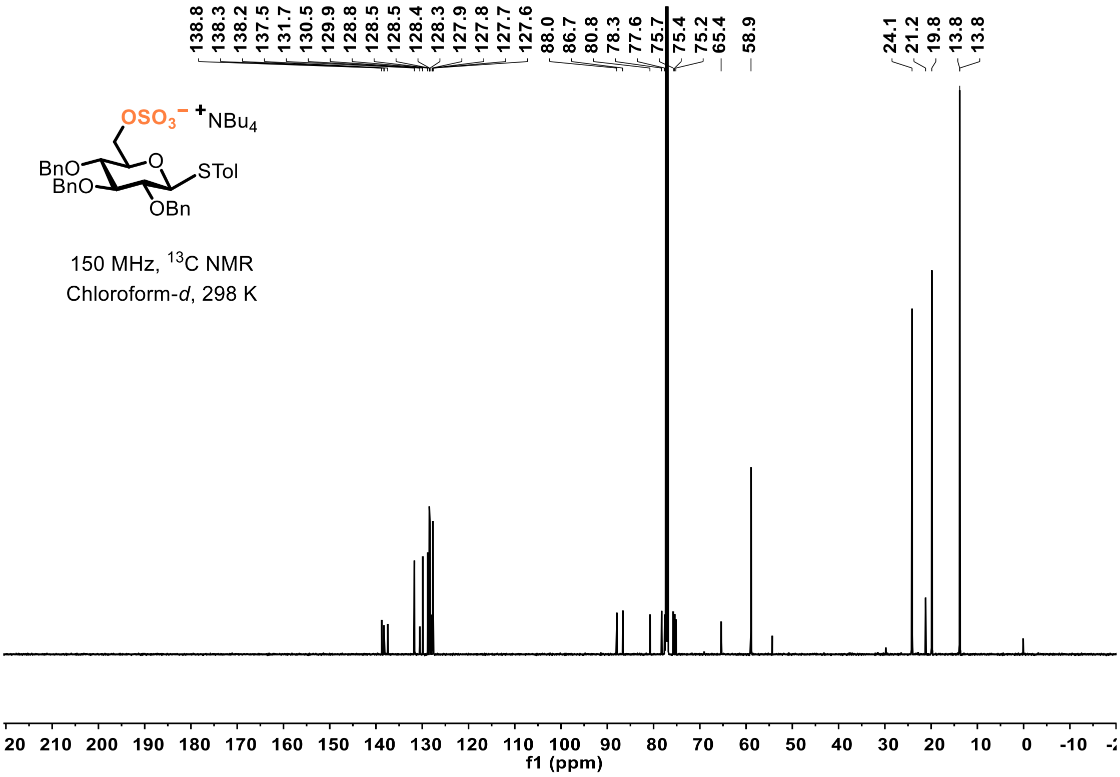

Tetrabutylammonium saccharide derivative sulfate (48)

^{1}H NMR of tetrabutylammonium saccharide derivative sulfate (48)

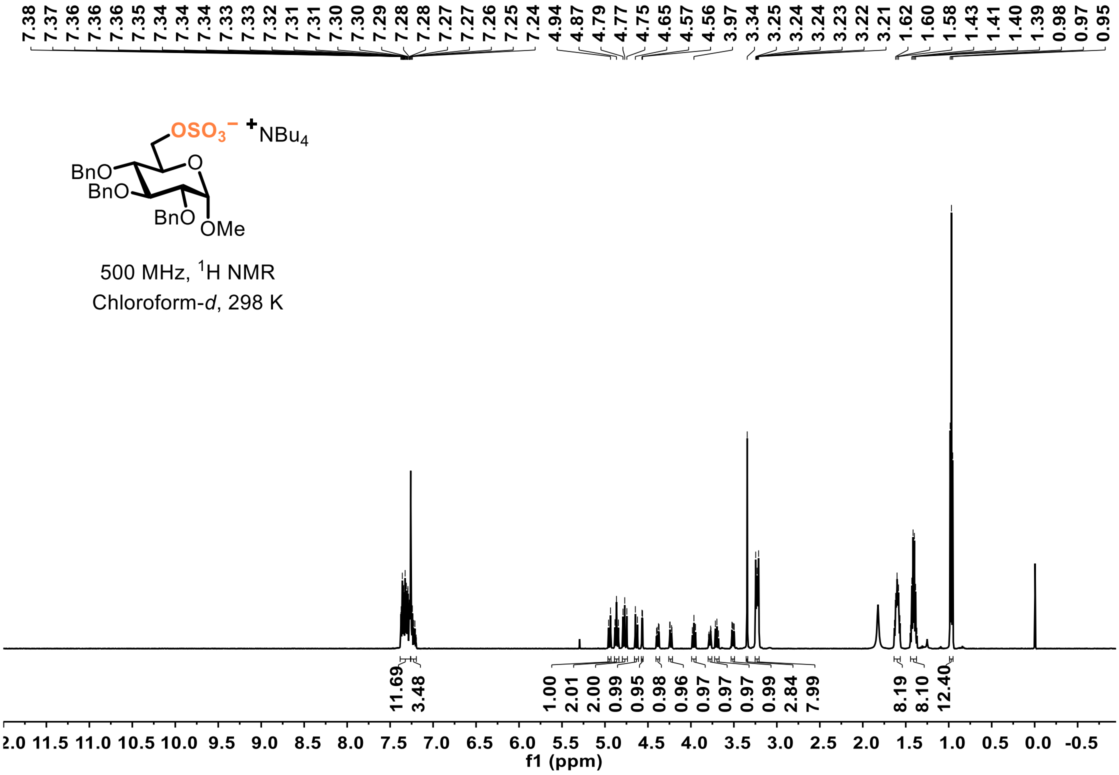

^{13}C NMR of tetrabutylammonium saccharide derivative sulfate (48)

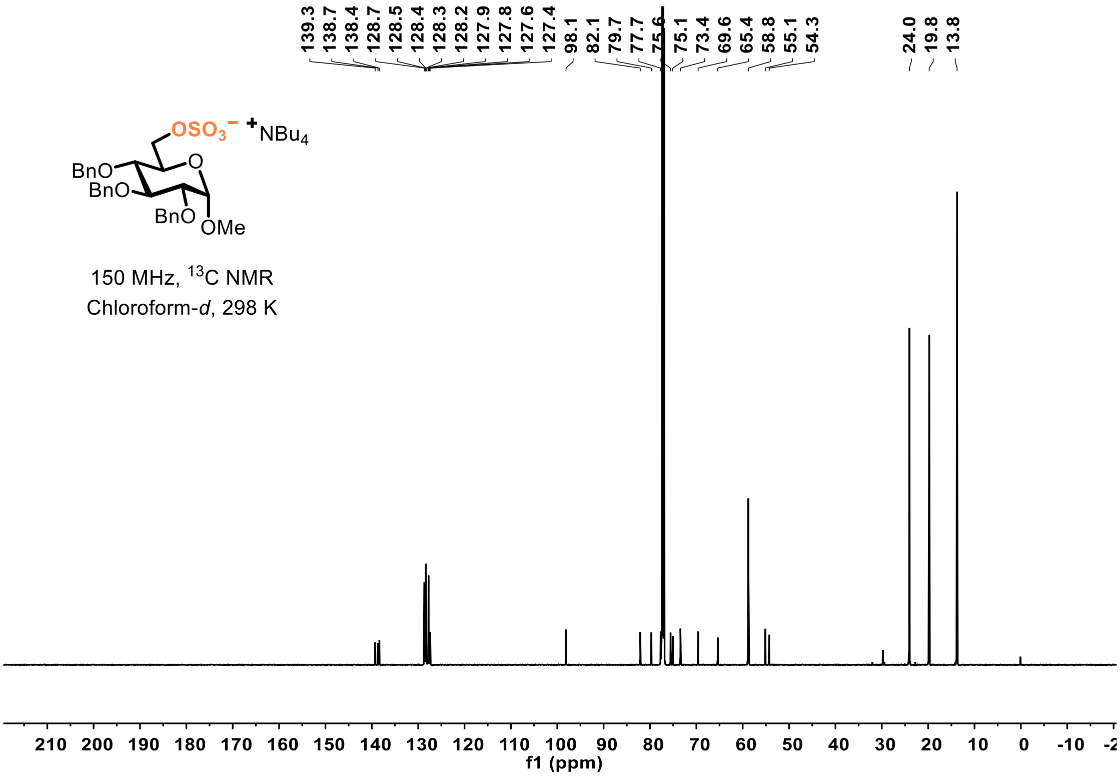

Tetrabutylammonium saccharide derivative sulfate (49)

^{1}H NMR of tetrabutylammonium saccharide derivative sulfate (49)

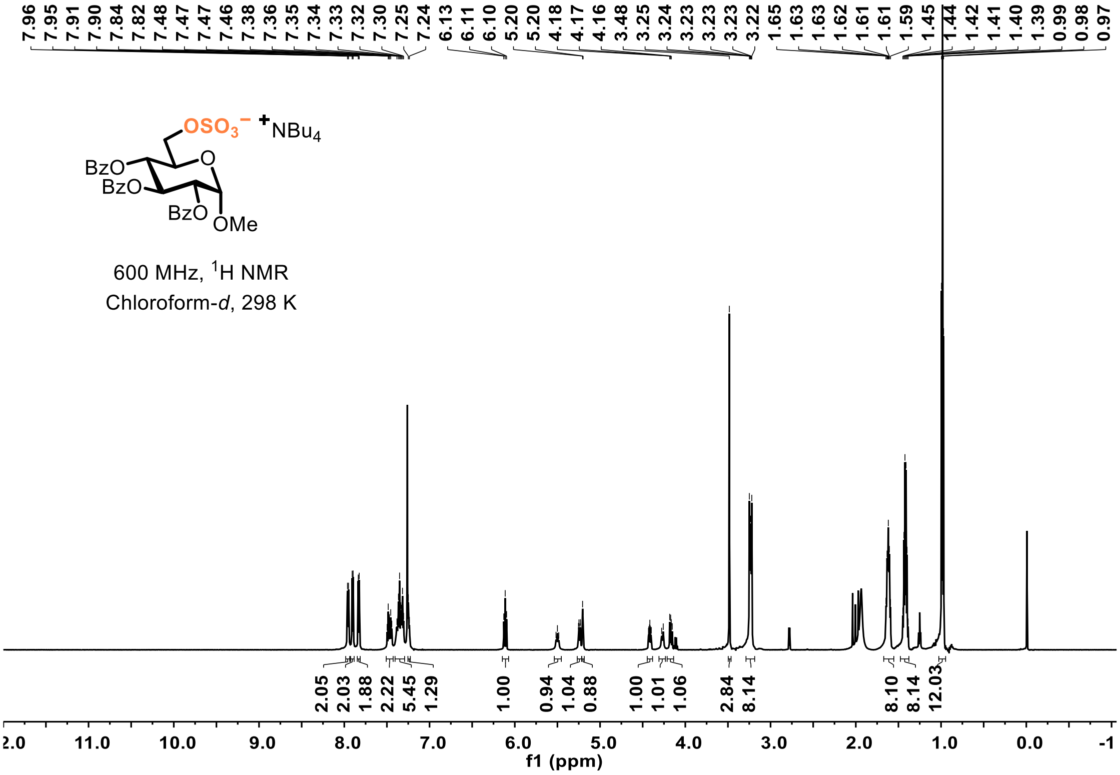

^{13}C NMR of tetrabutylammonium saccharide derivative sulfate (49)

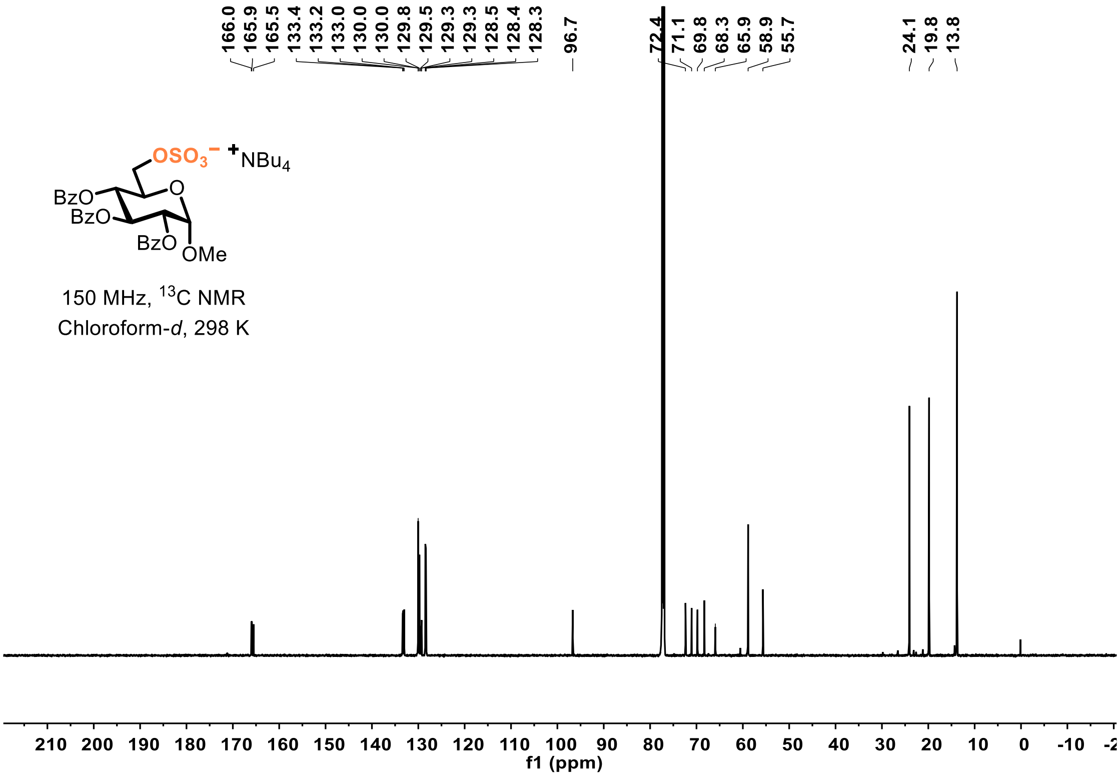

Tetrabutylammonium saccharide derivative sulfate (50)

^{1}H NMR of tetrabutylammonium saccharide derivative sulfate (50)

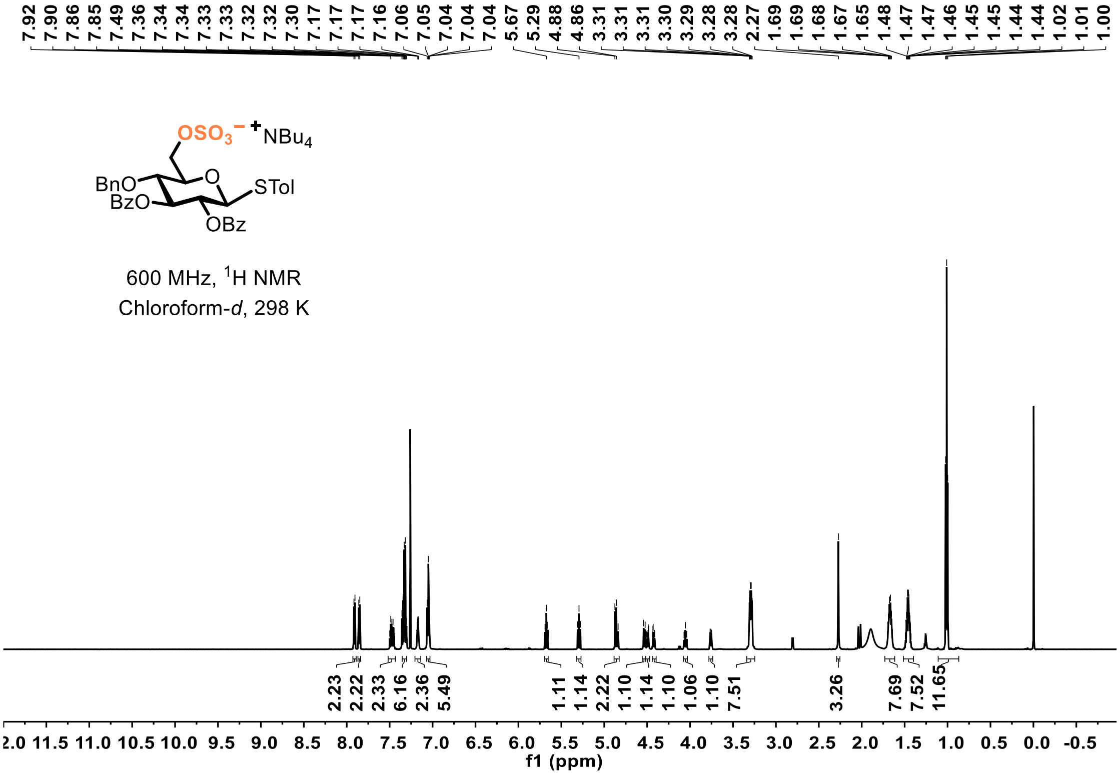

^{13}C NMR of tetrabutylammonium saccharide derivative sulfate (50)

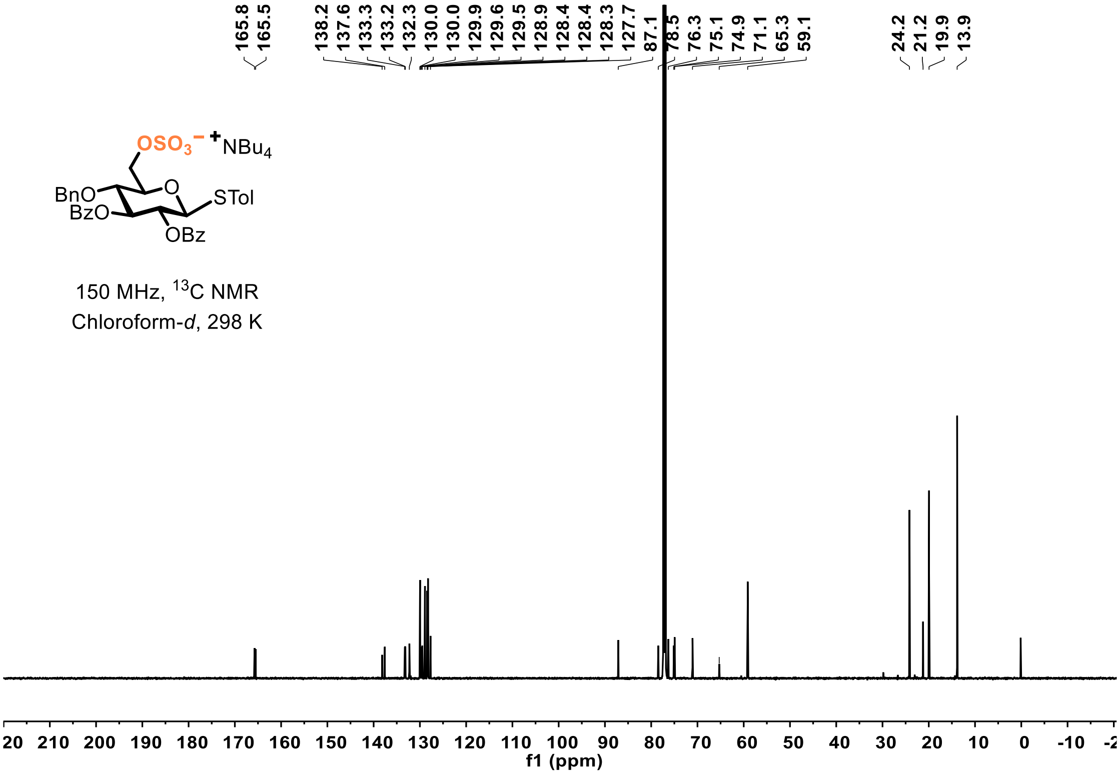

Tetrabutylammonium saccharide derivative sulfate (51)

^{1}H NMR of tetrabutylammonium saccharide derivative sulfate (51)

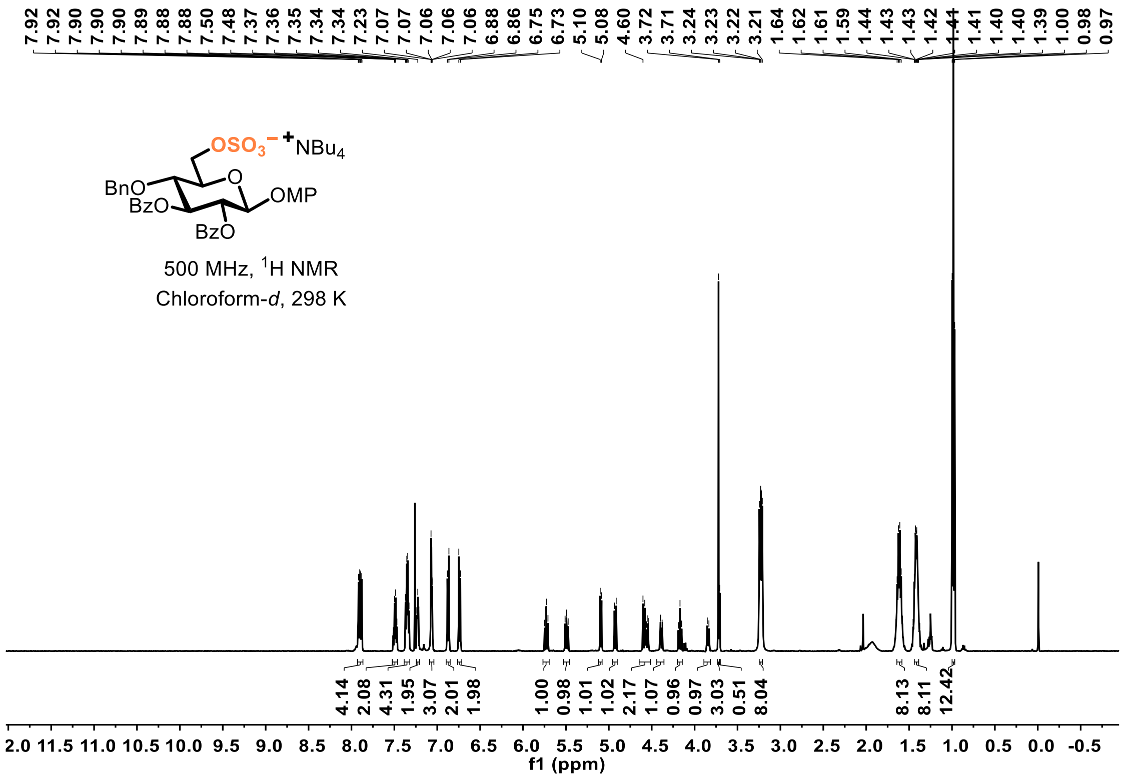

^{13}C NMR of tetrabutylammonium saccharide derivative sulfate (51)

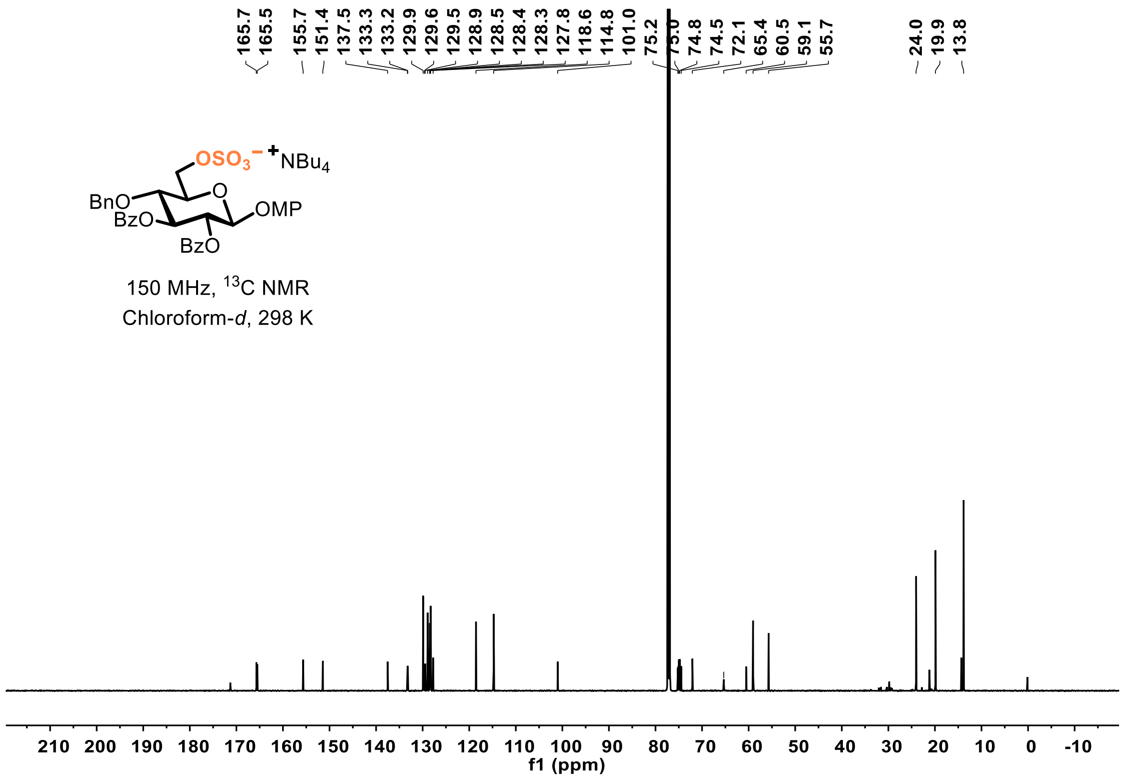

Tetrabutylammonium saccharide derivative sulfate (52)

^1H NMR of tetrabutylammonium saccharide derivative sulfate (52)

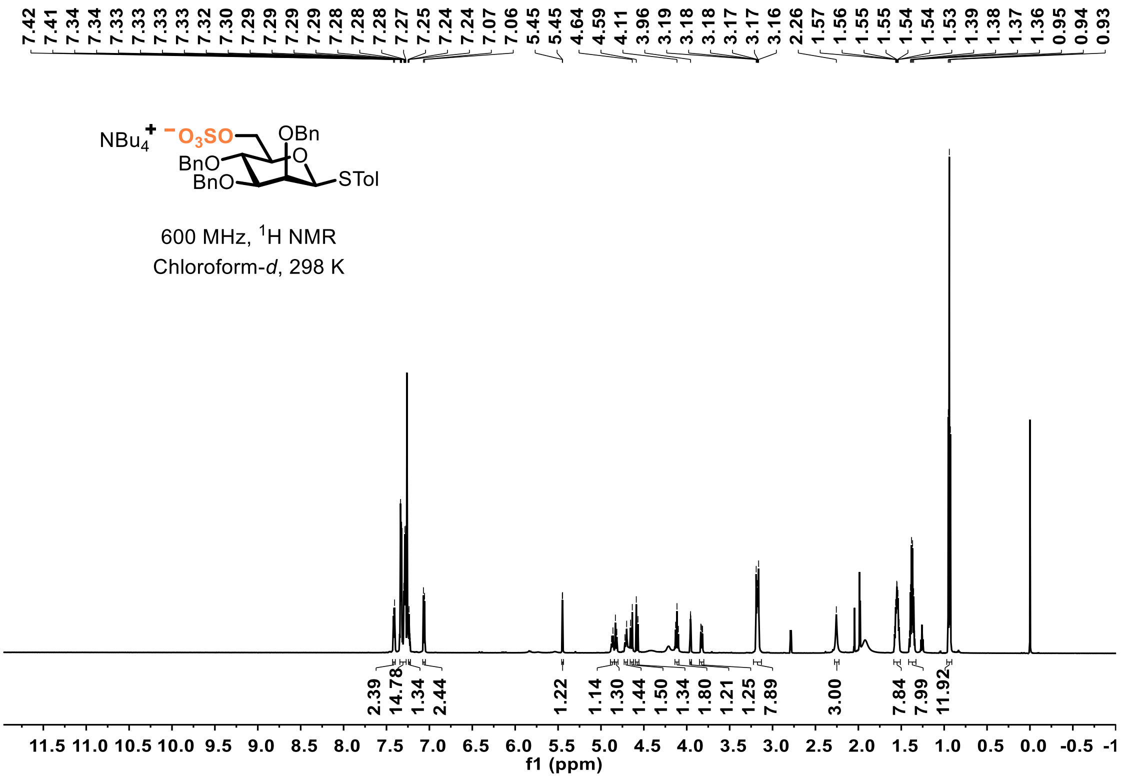

^{13}C NMR of tetrabutylammonium saccharide derivative sulfate (52)

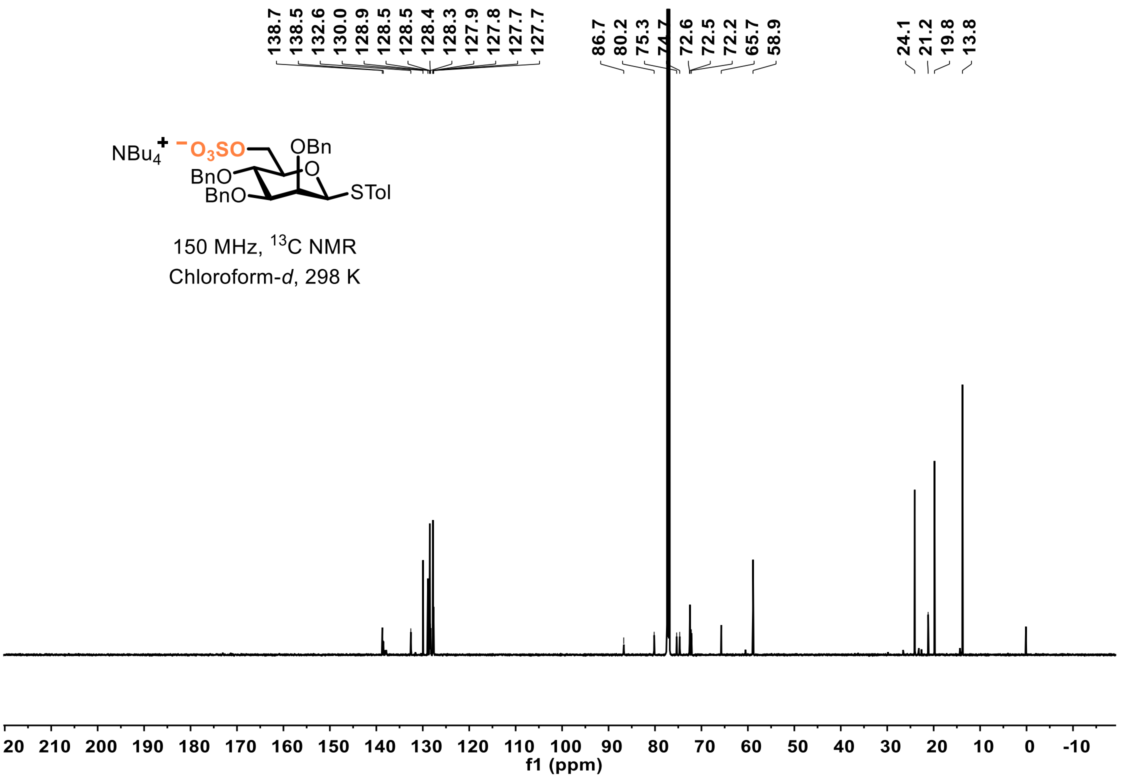

Tetrabutylammonium saccharide derivative sulfate (53)

^{1}H NMR of tetrabutylammonium saccharide derivative sulfate (53)

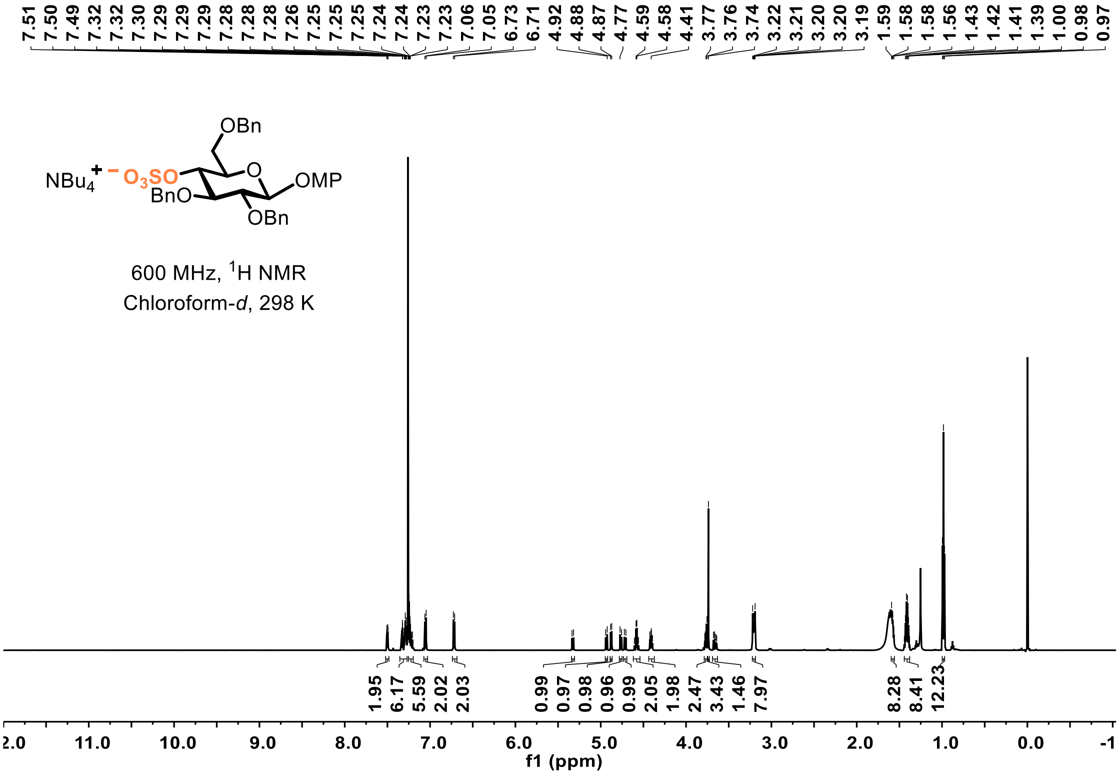

^{13}C NMR of tetrabutylammonium saccharide derivative sulfate (53)

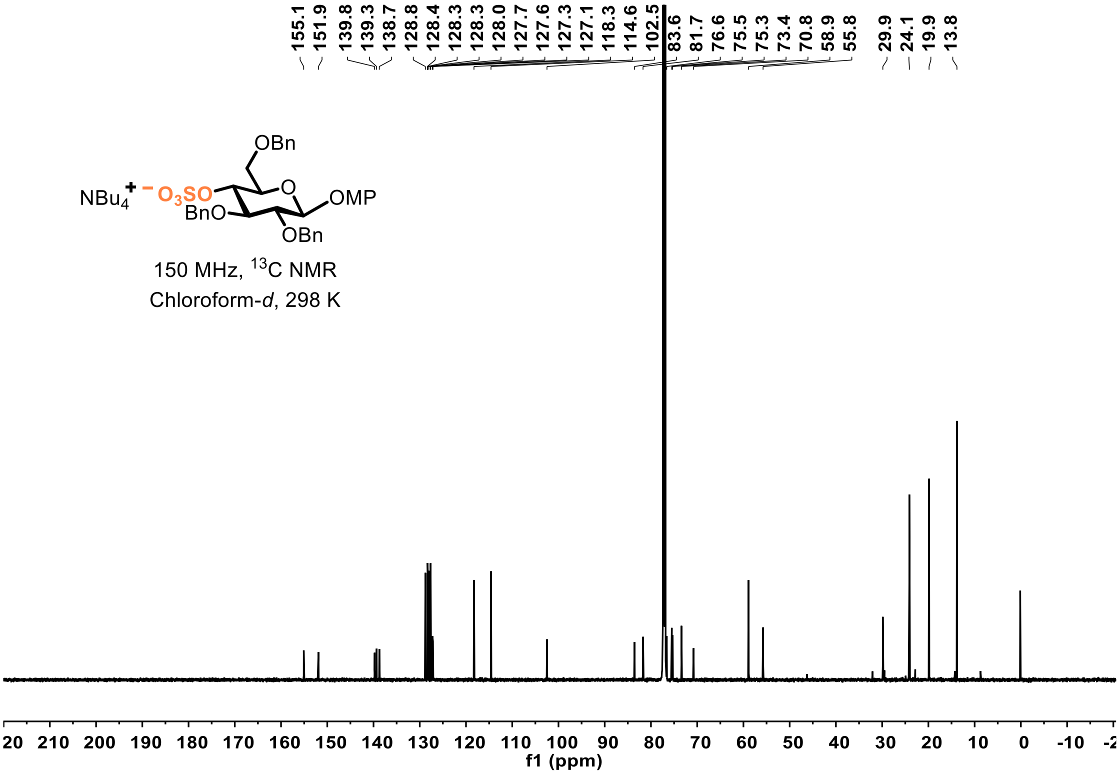

Tetrabutylammonium saccharide derivative sulfate (54)

^{1}H NMR of tetrabutylammonium saccharide derivative sulfate (54)

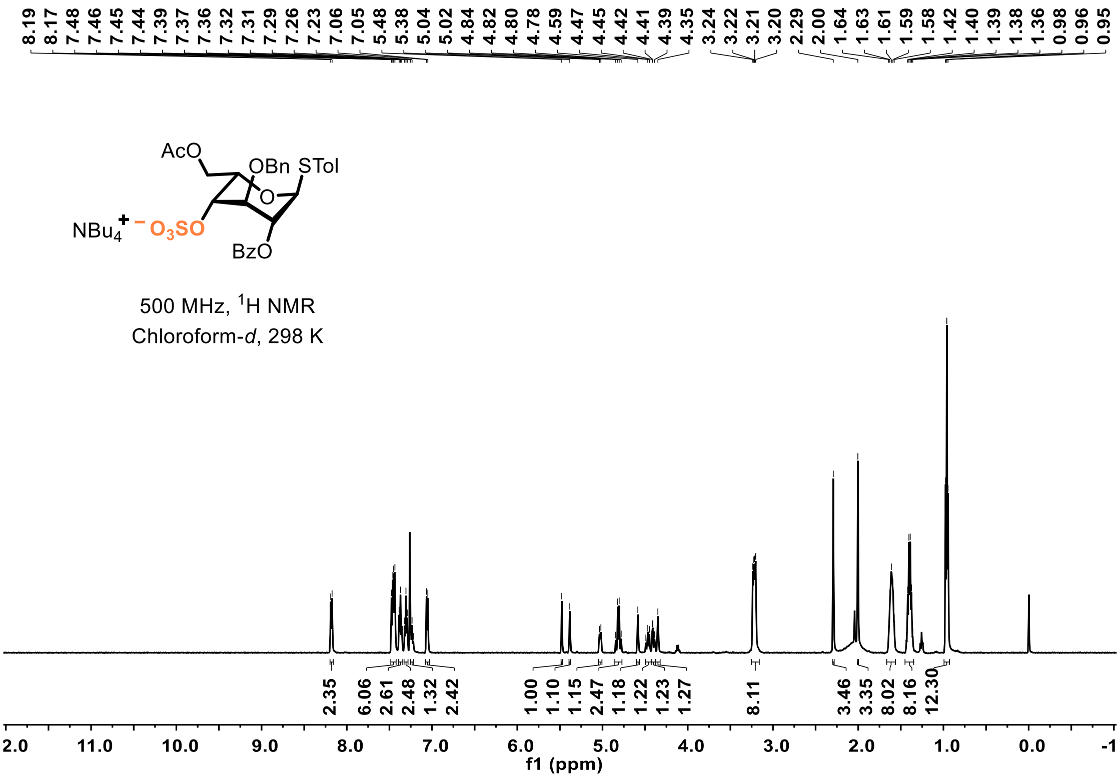

^{13}C NMR of tetrabutylammonium saccharide derivative sulfate (54)

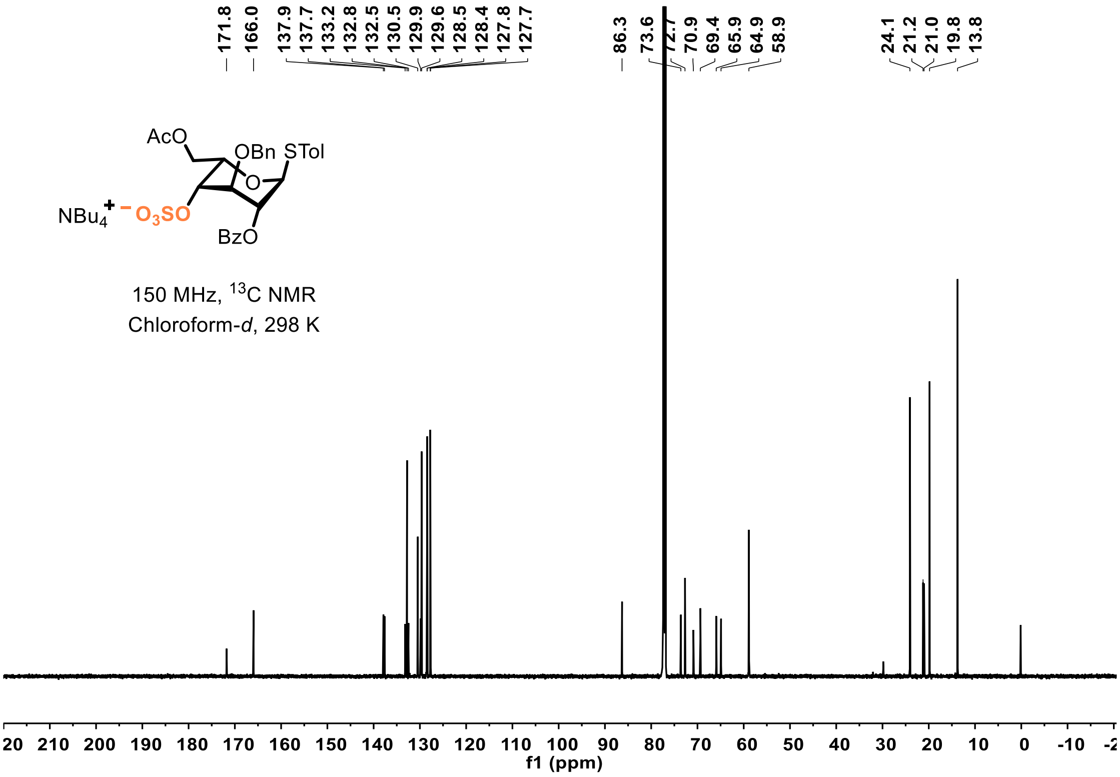

Tetrabutylammonium saccharide derivative sulfate (55)

^{1}H NMR of tetrabutylammonium saccharide derivative sulfate (55)

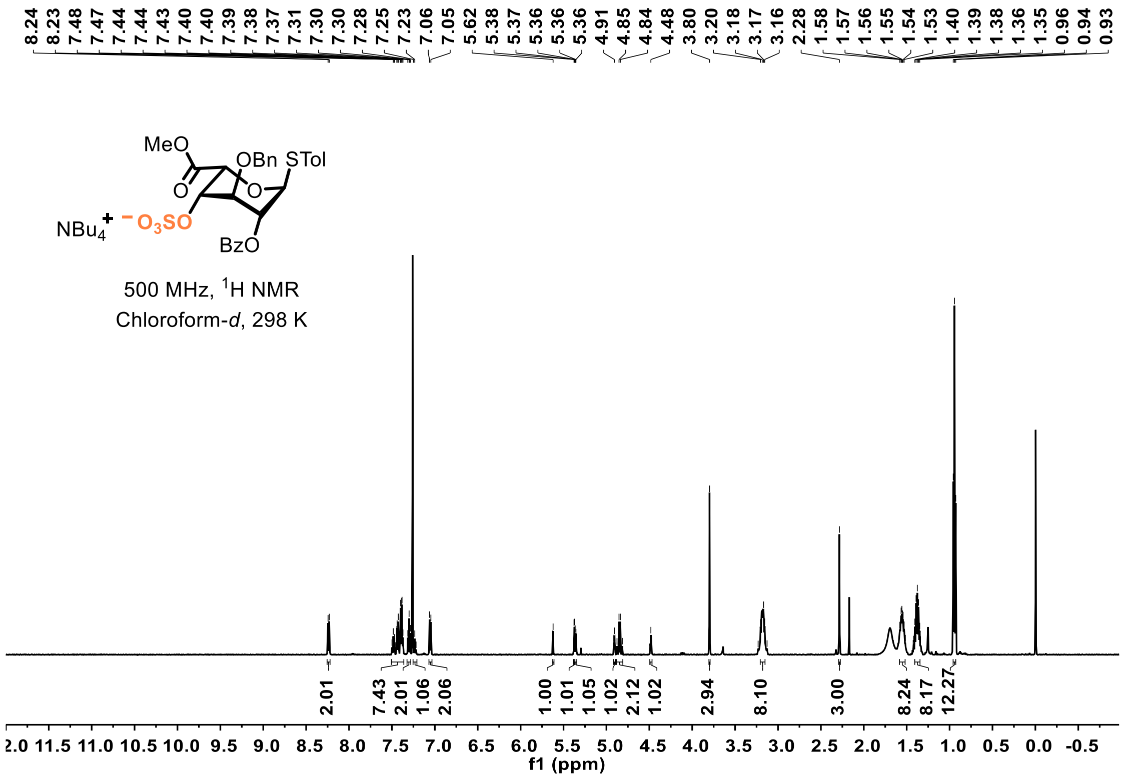

^{13}C NMR of tetrabutylammonium saccharide derivative sulfate (55)

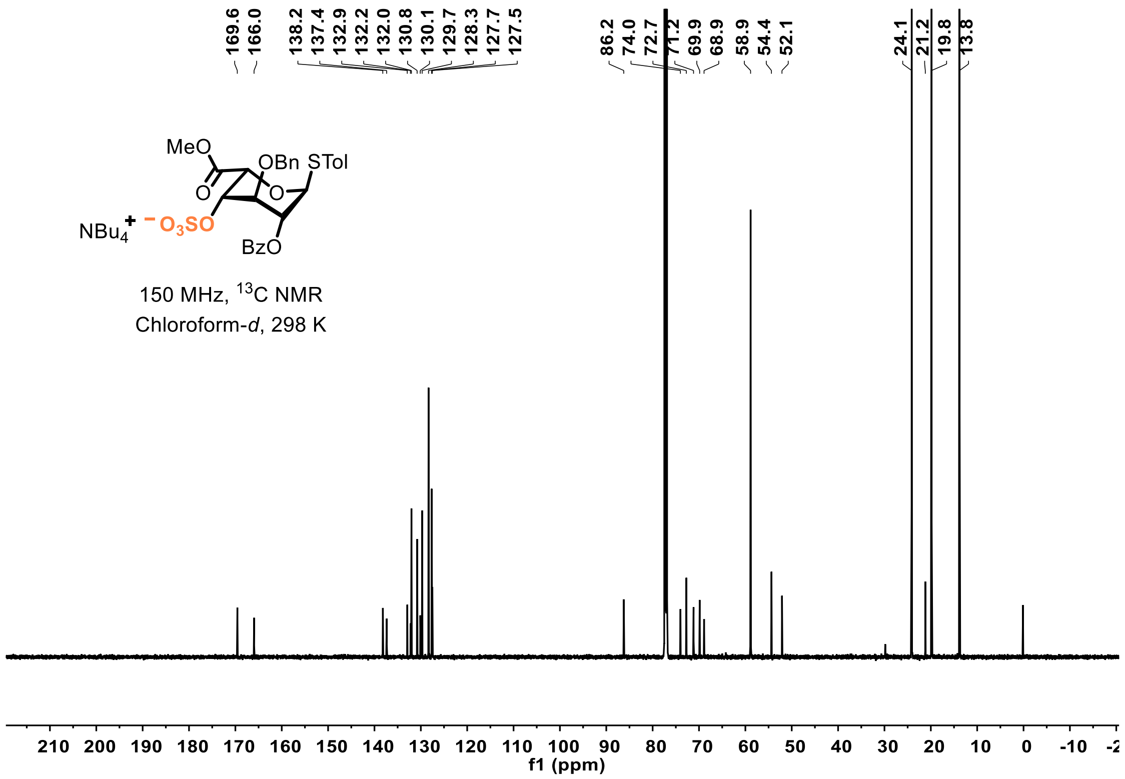

Tetrabutylammonium saccharide derivative sulfate (56)

^{1}H NMR of tetrabutylammonium saccharide derivative sulfate (56)

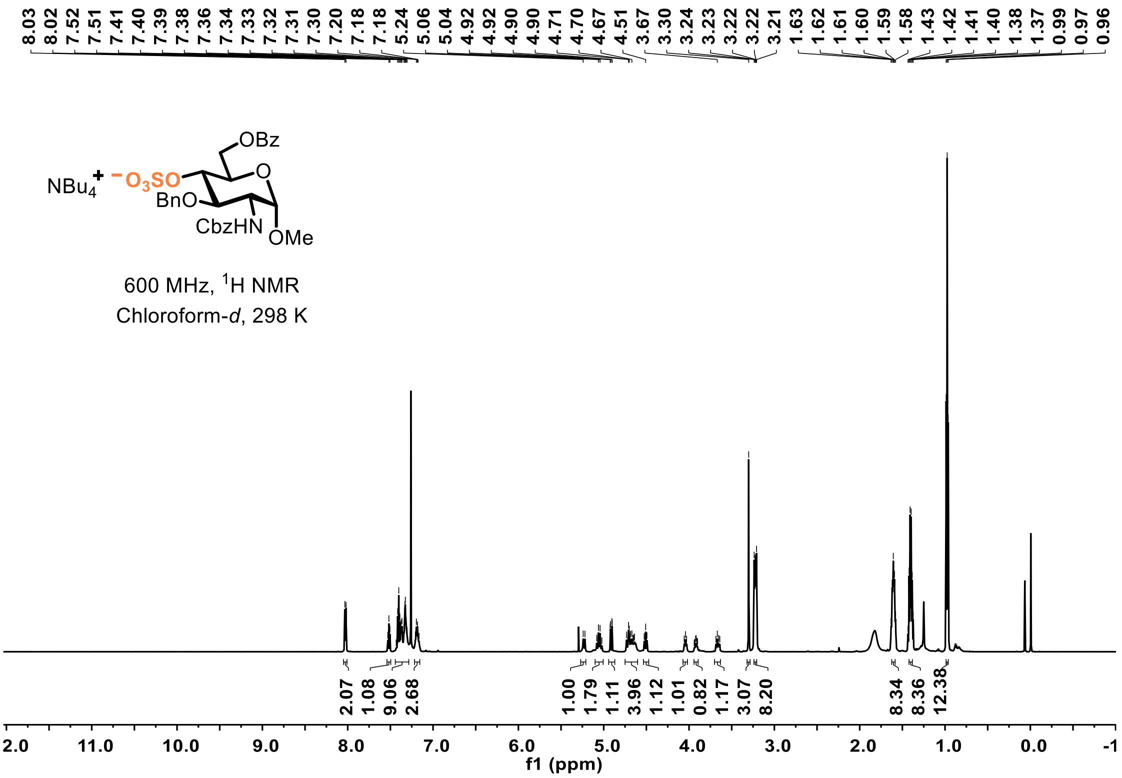

^{13}C NMR of tetrabutylammonium saccharide derivative sulfate (56)

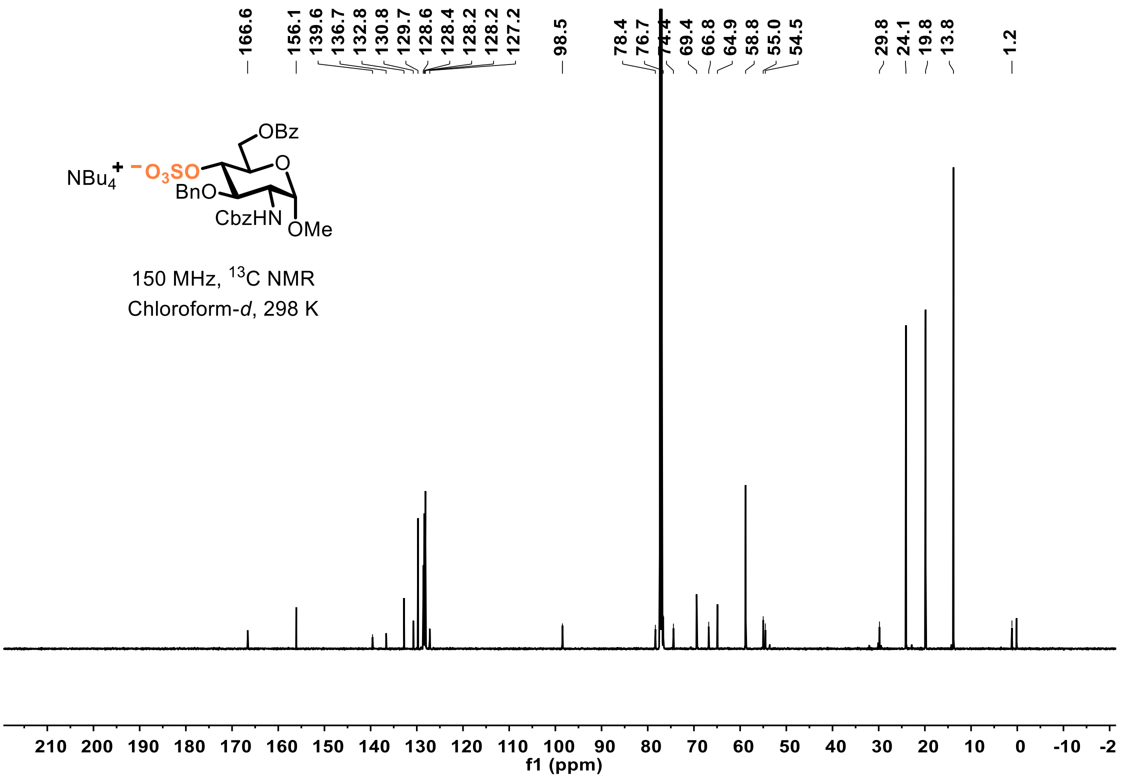

Tetrabutylammonium saccharide derivative sulfate (57)

^{1}H NMR of tetrabutylammonium saccharide derivative sulfate (57)

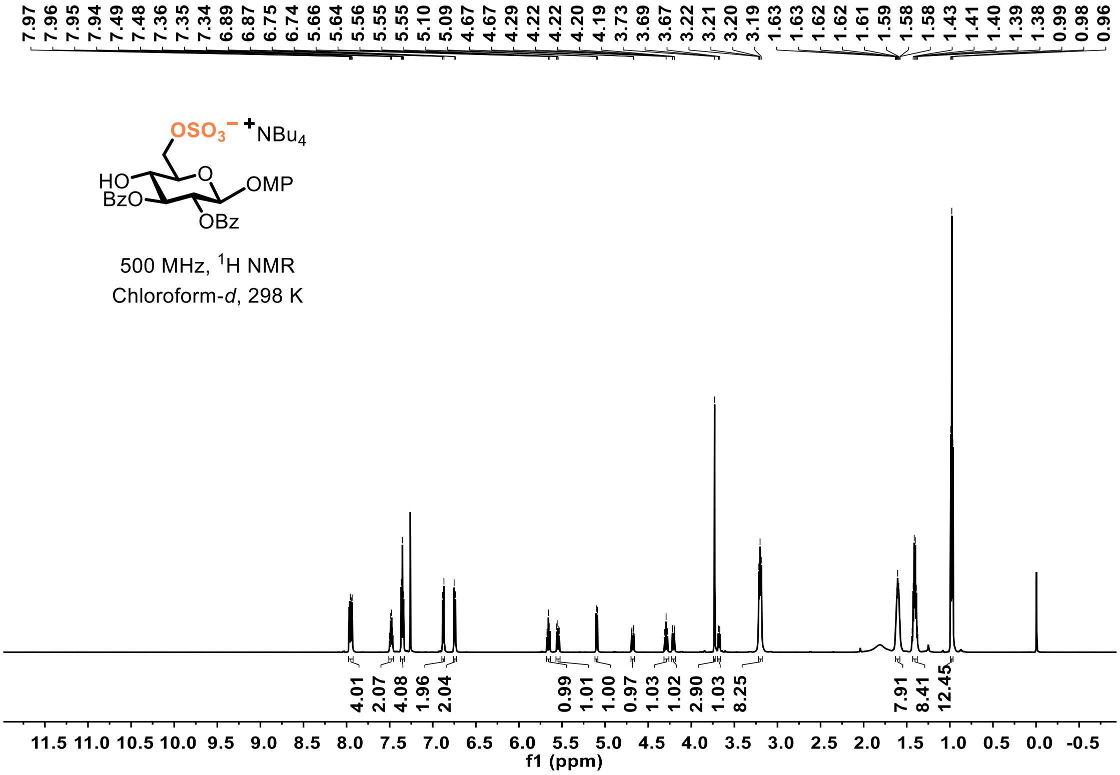

^{13}C NMR of tetrabutylammonium saccharide derivative sulfate (57)

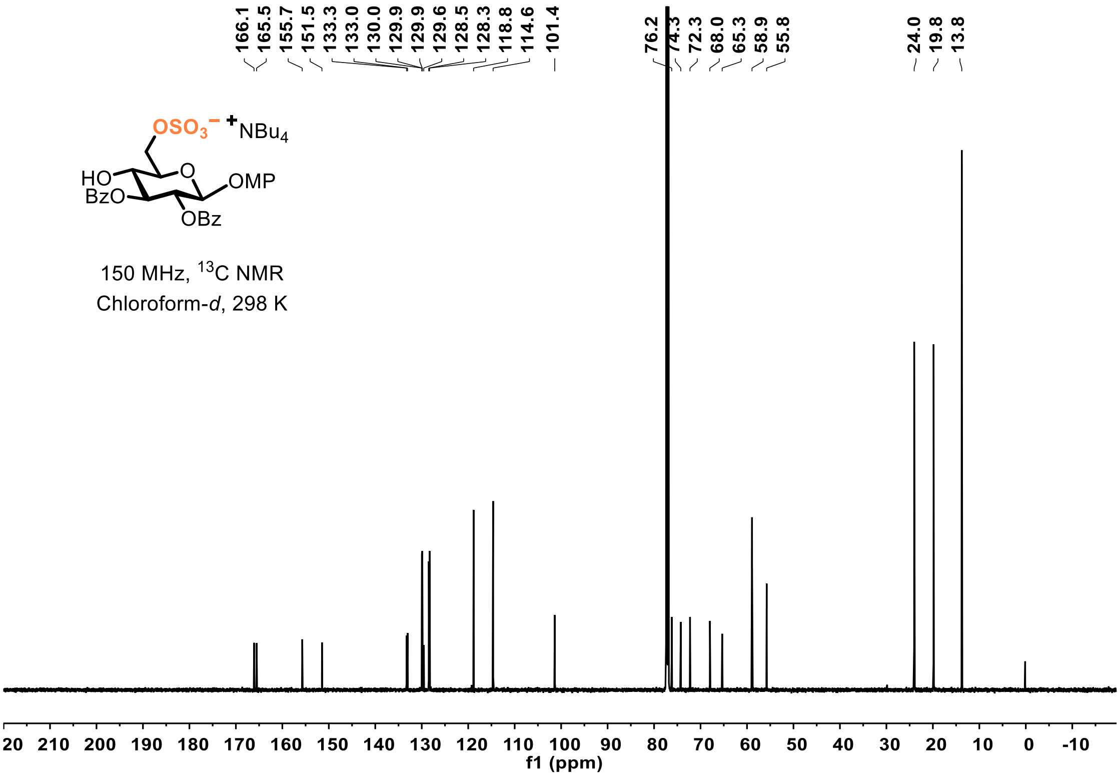

Tetrabutylammonium saccharide derivative sulfate (58)

^{1}H NMR of tetrabutylammonium saccharide derivative sulfate (58)

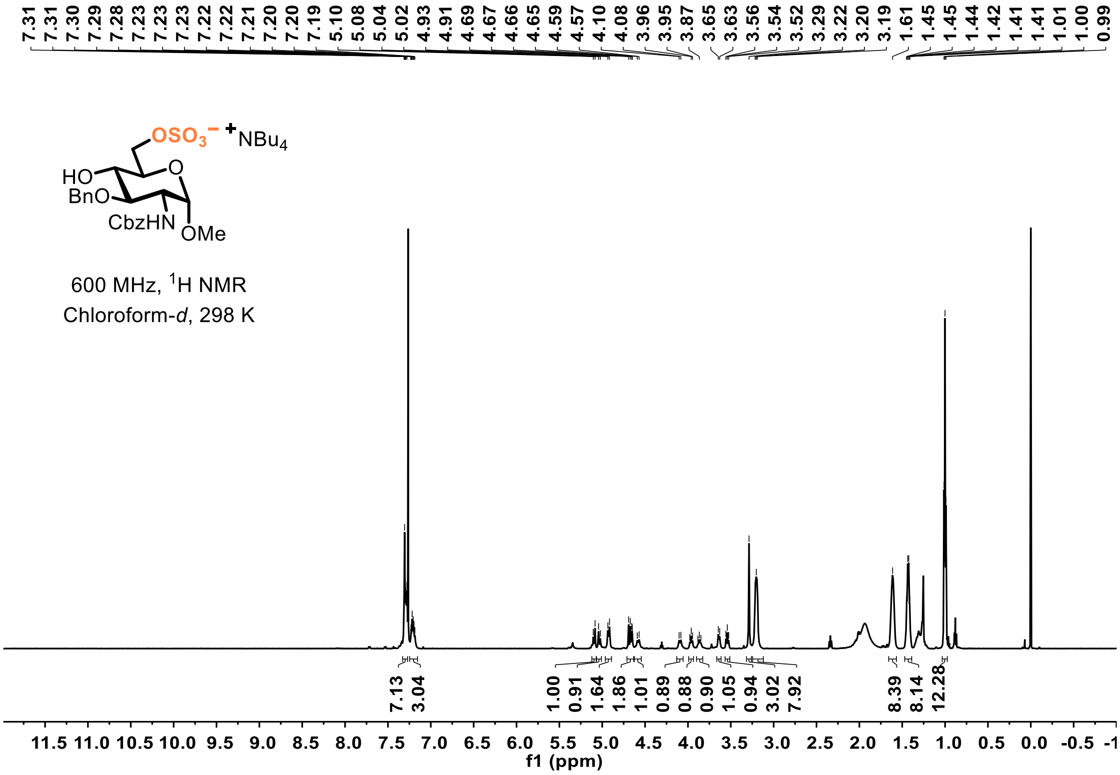

^{13}C NMR of tetrabutylammonium saccharide derivative sulfate (58)

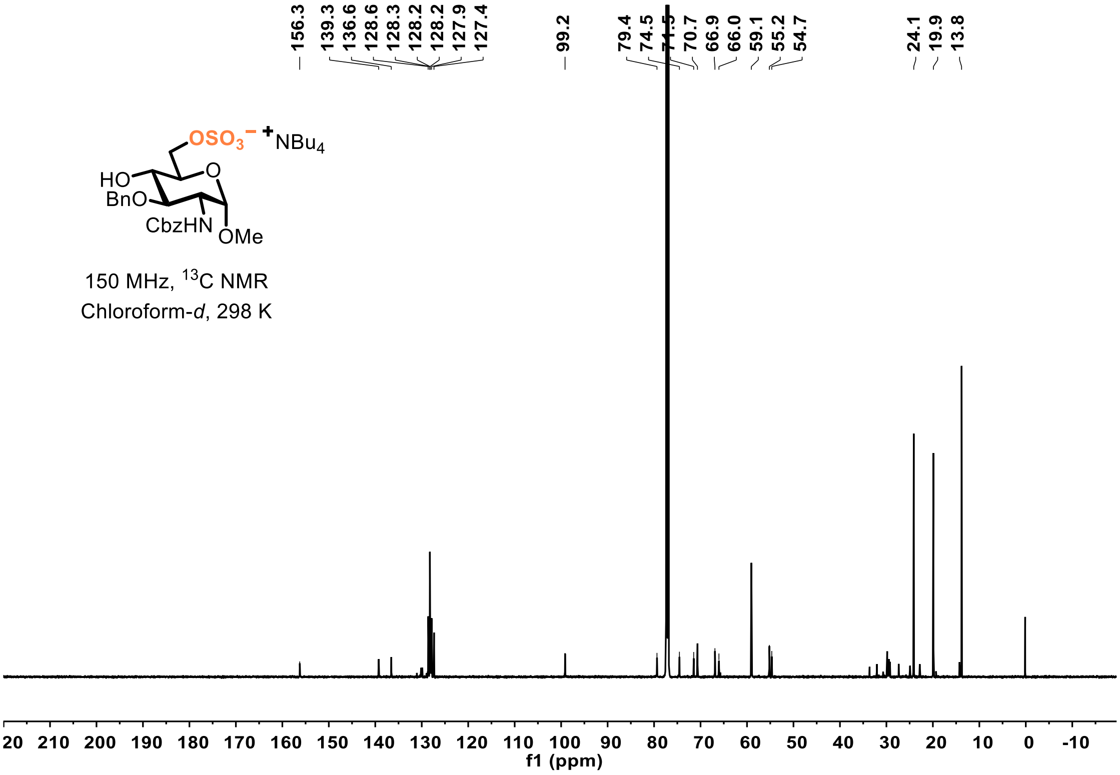

Tetrabutylammonium saccharide derivative sulfate (59)

^{1}H NMR of tetrabutylammonium saccharide derivative sulfate (59)

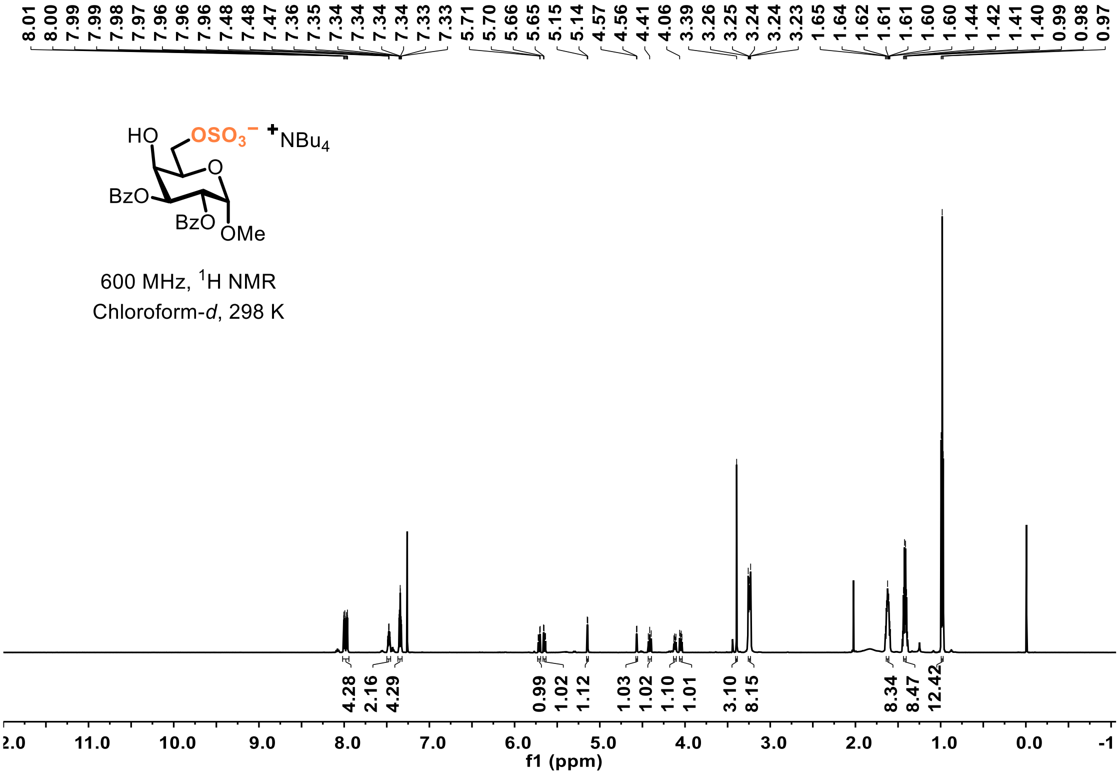

^{13}C NMR of tetrabutylammonium saccharide derivative sulfate (59)

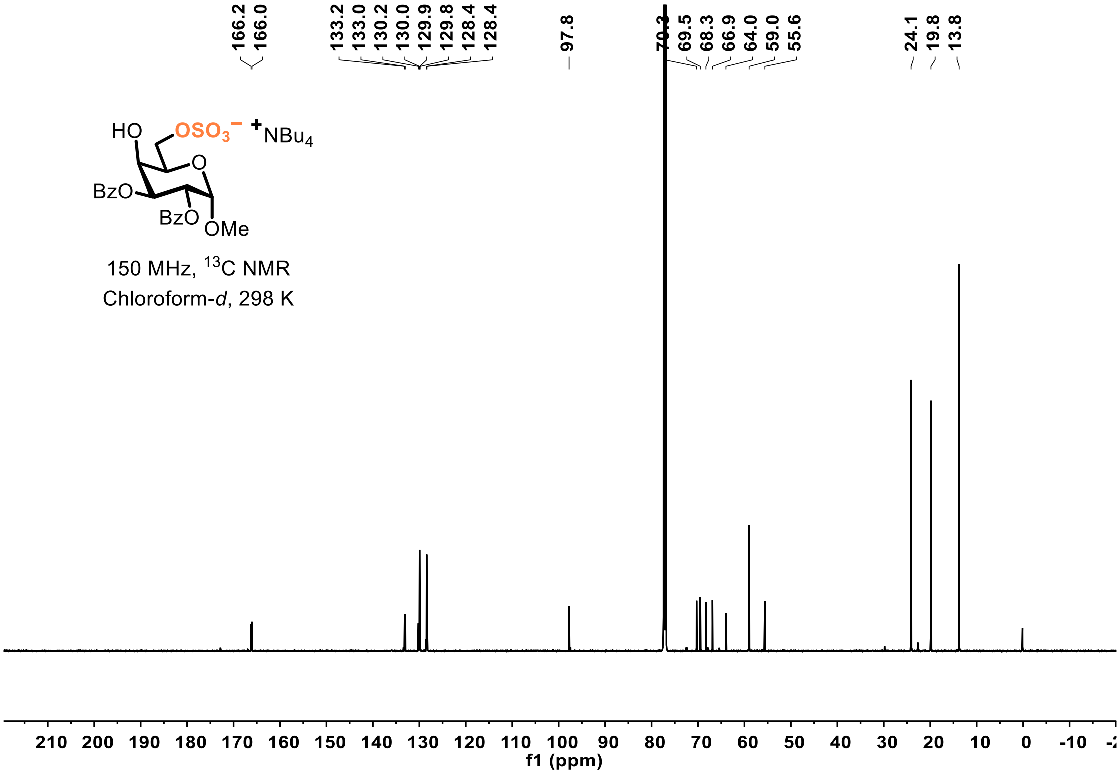

Tetrabutylammonium 4-(4-(3-hydroxypropoxy)phenyl)-2-methylbutan-2-ol sulfate (60)

^{1}H NMR of tetrabutylammonium 4-(4-(3-hydroxypropoxy)phenyl)-2-methylbutan-2-ol sulfate (60)

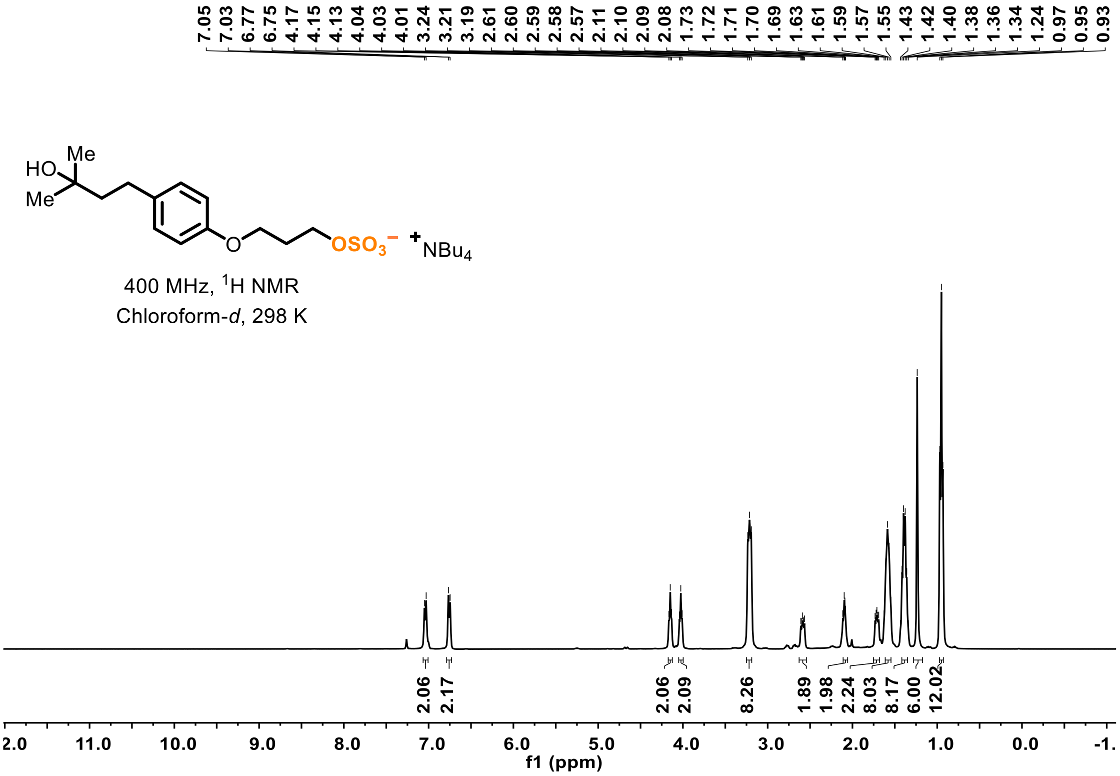

^{13}C NMR of tetrabutylammonium 4-(4-(3-hydroxypropoxy)phenyl)-2-methylbutan-2-ol sulfate (60)

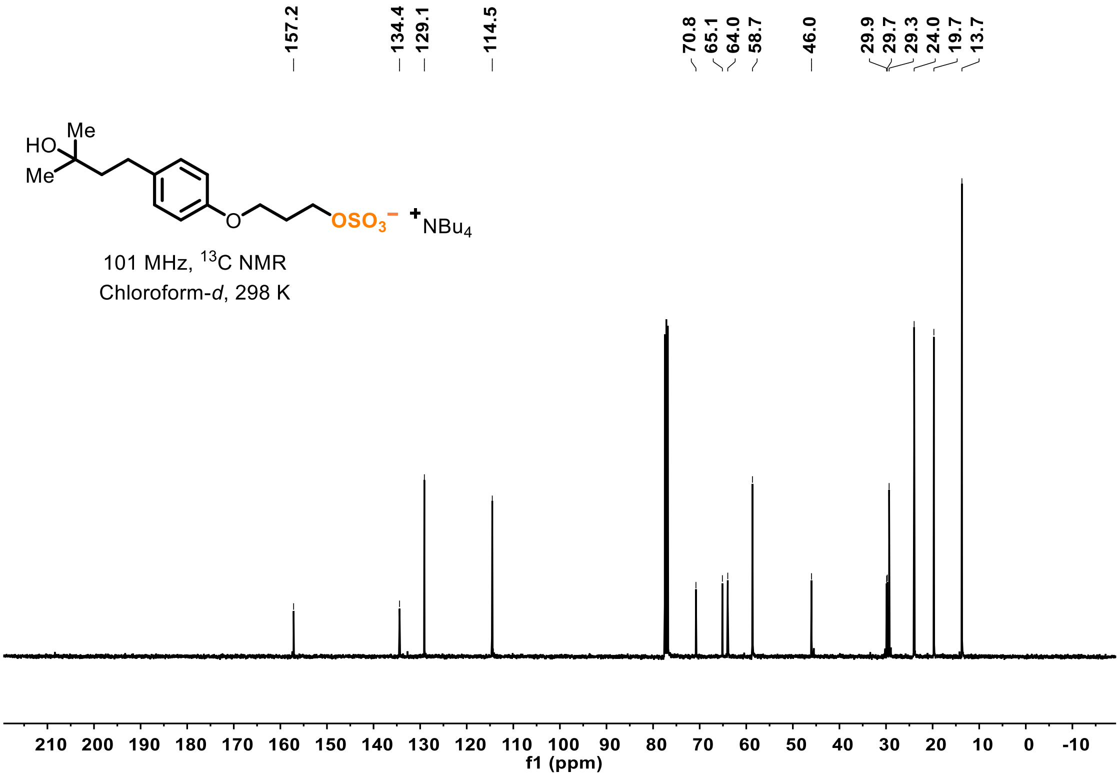

Sodium 4-hydroxyphenethyl alcohol sulfate (61)

^{1}H NMR of sodium 4-hydroxyphenethyl alcohol sulfate (61)

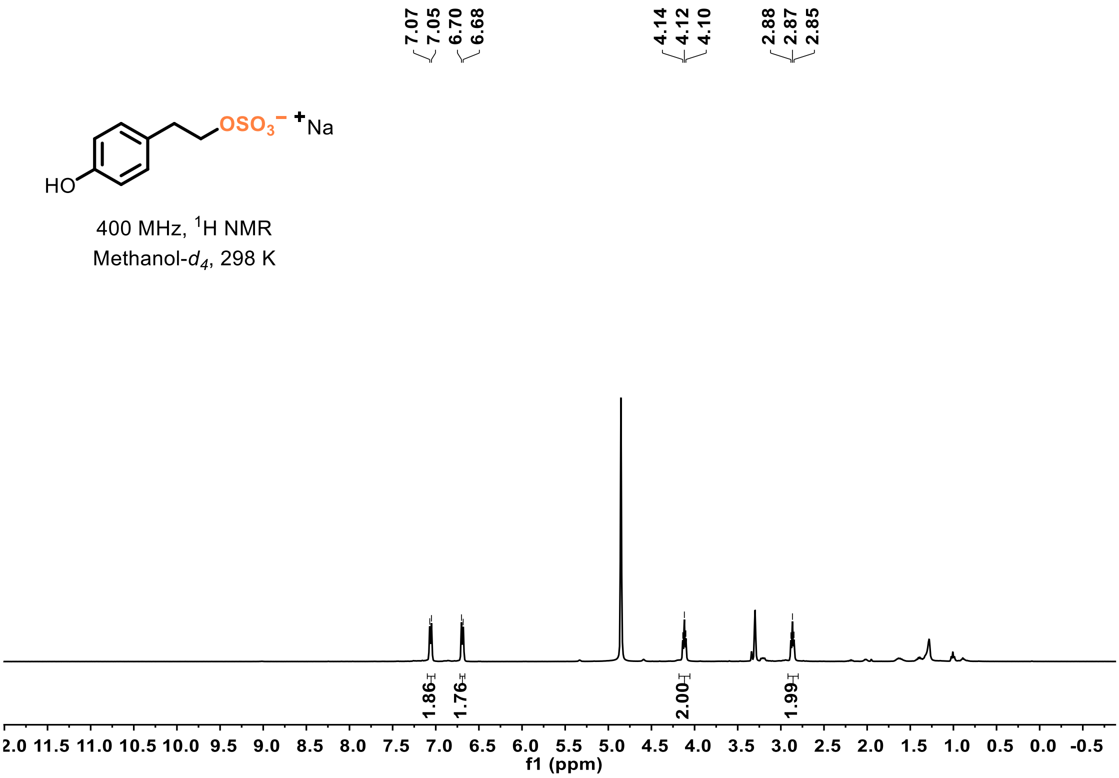

^{13}C NMR of sodium 4-hydroxyphenethyl alcohol sulfate (61)

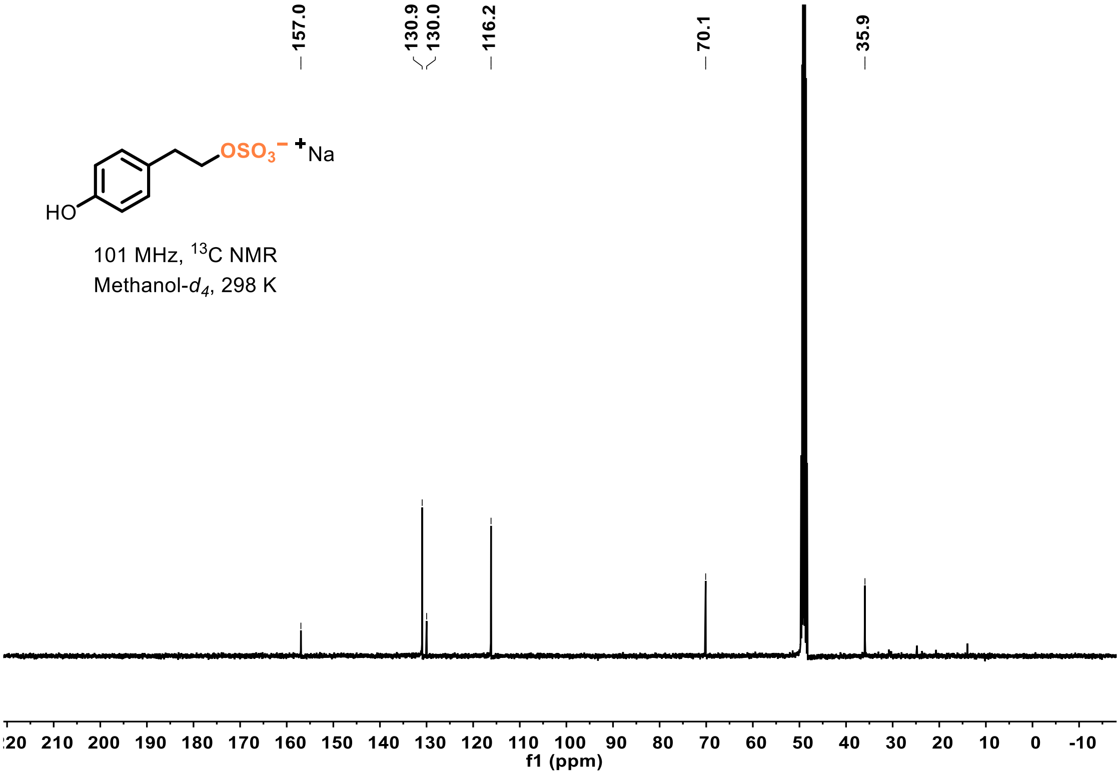

Tetrabutylammonium proline derivative sulfate (62)

^{1}H NMR of tetrabutylammonium proline derivative sulfate (62)

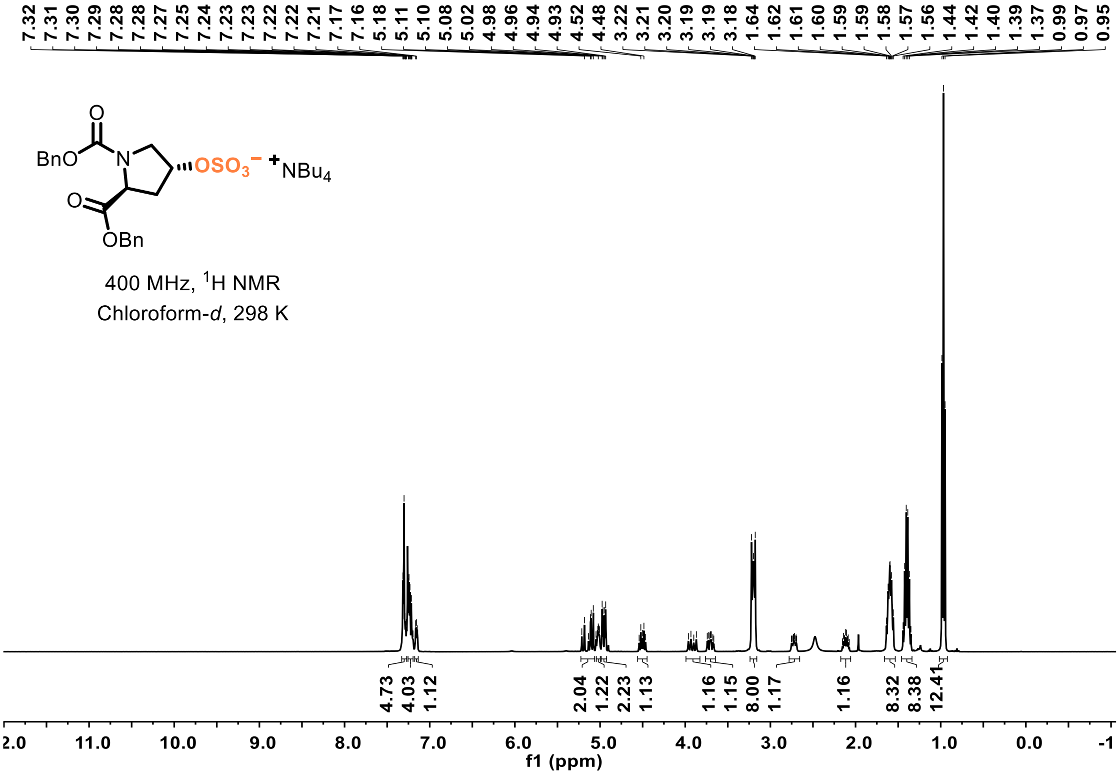

^{13}C NMR of tetrabutylammonium proline derivative sulfate (62)

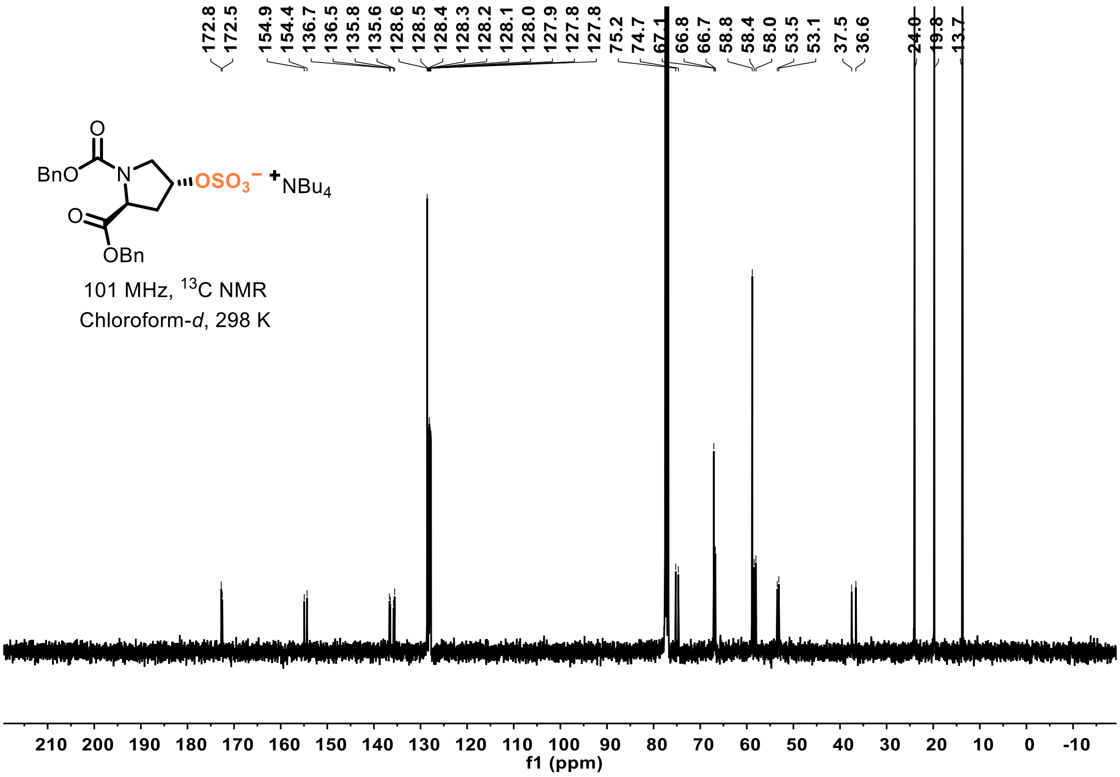

Tetrabutylammonium serine derivative sulfate (63)

^{1}H NMR of tetrabutylammonium serine derivative sulfate (63)

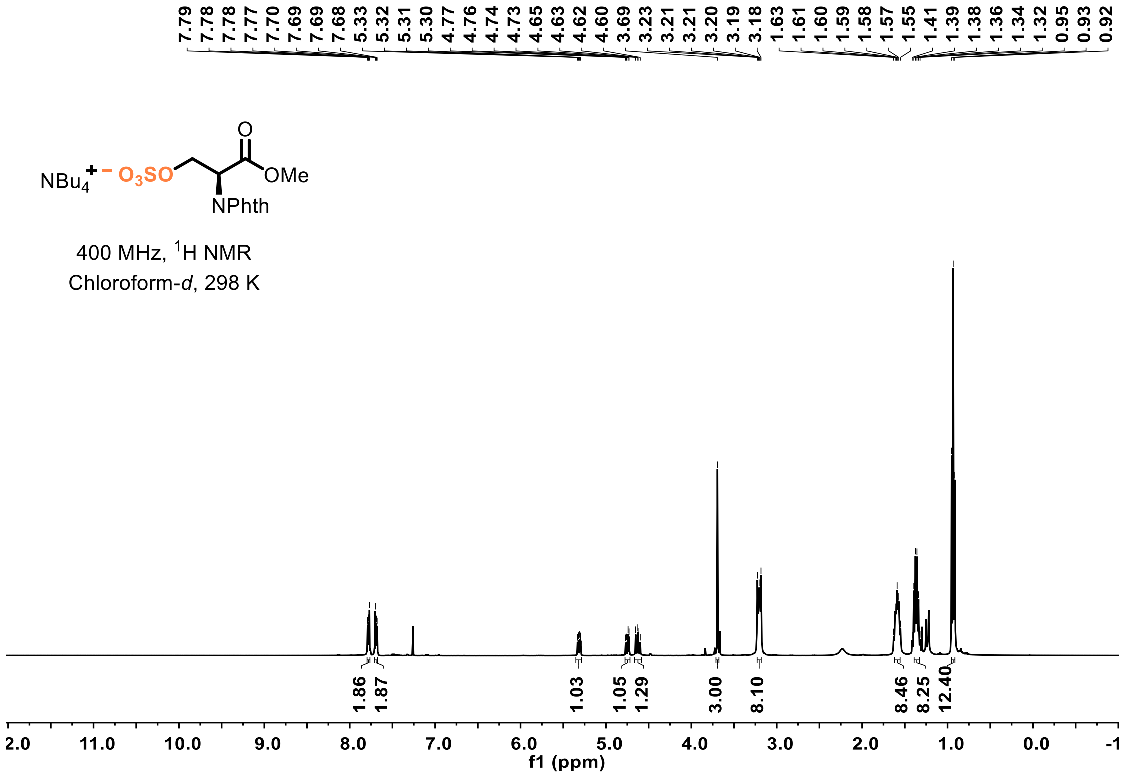

^{13}C NMR of tetrabutylammonium serine derivative sulfate (63)

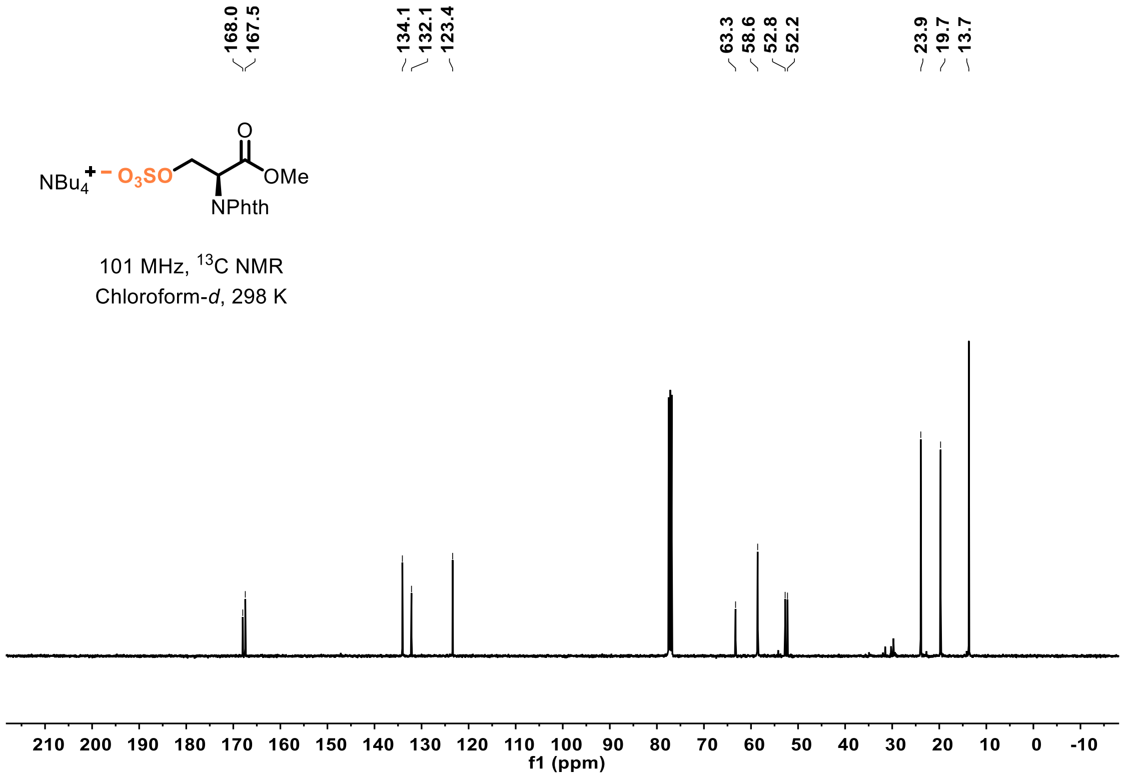

Tetrabutylammonium picaridin sulfate (64)

^{1}H NMR of tetrabutylammonium picaridin sulfate (64)

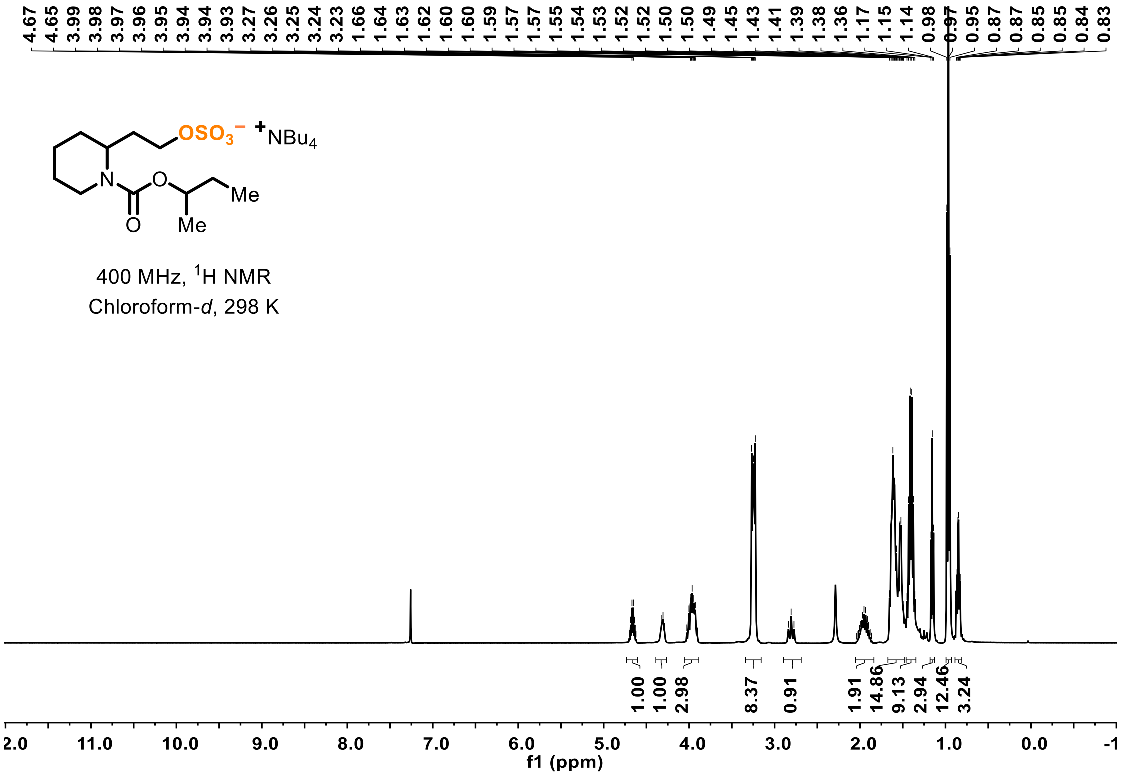

^{13}C NMR of tetrabutylammonium picaridin sulfate (64)

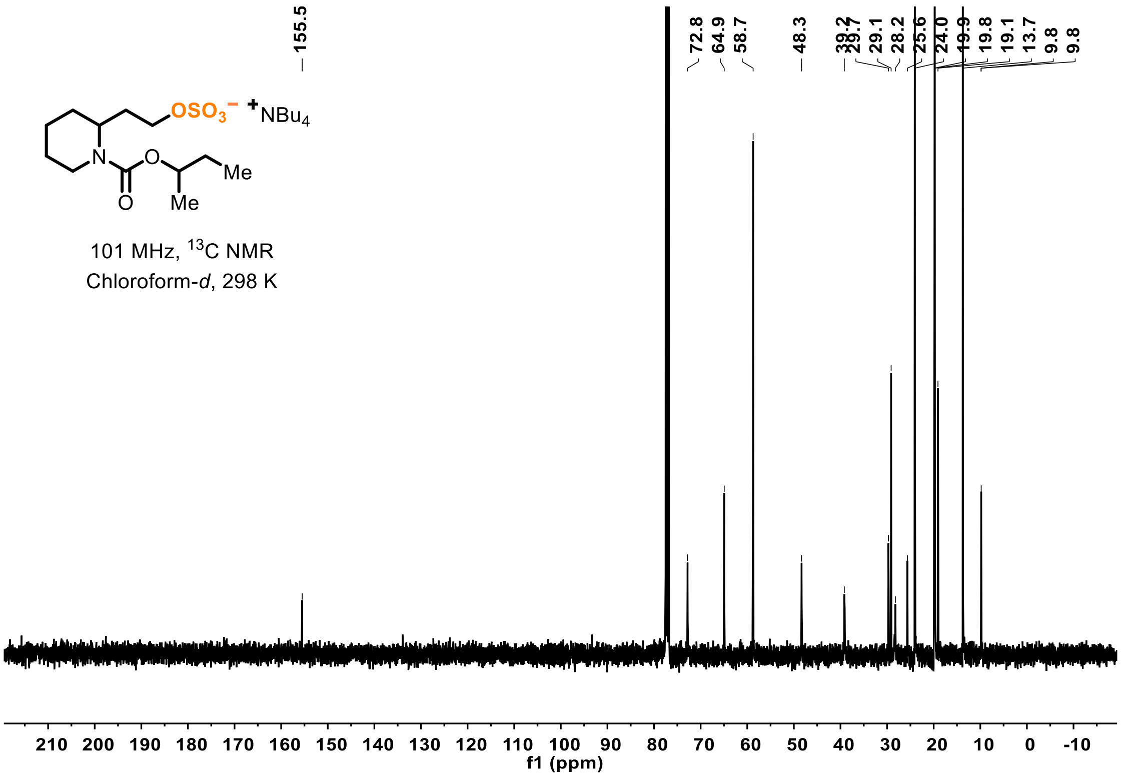

Tetrabutylammonium aloe emodin sulfate (65)

^{1}H NMR of tetrabutylammonium aloe emodin sulfate (65)

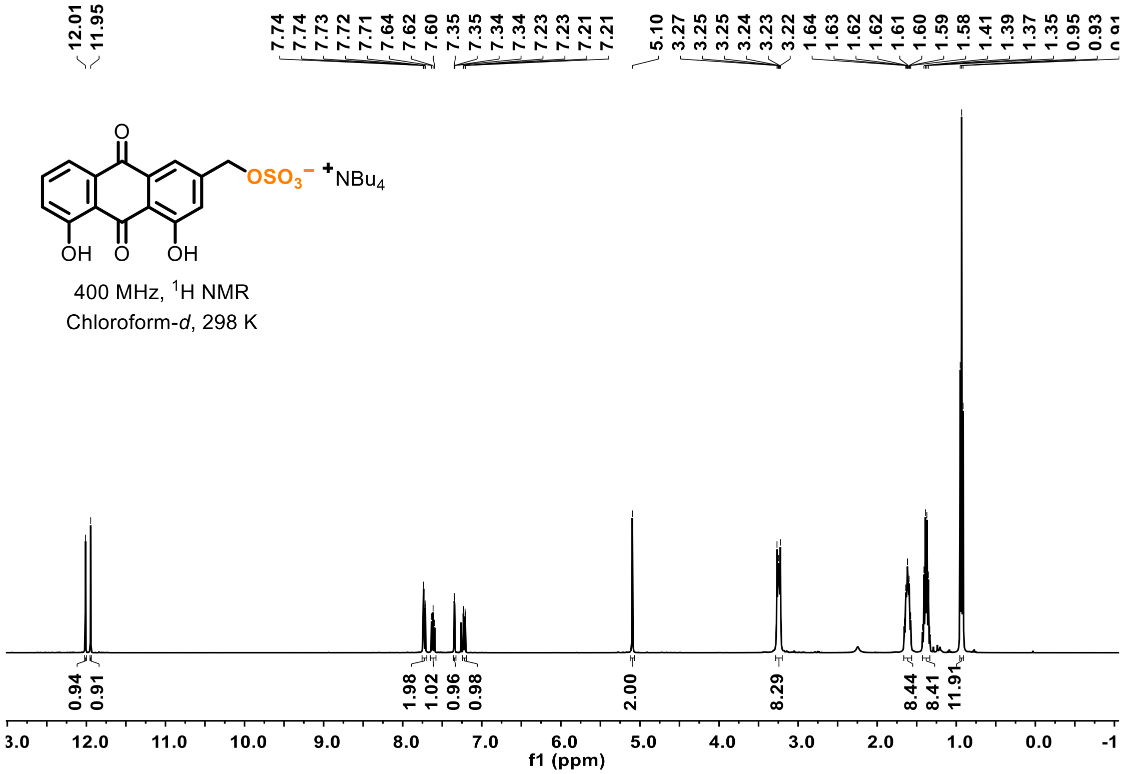

^{13}C NMR of tetrabutylammonium aloe emodin sulfate (65)

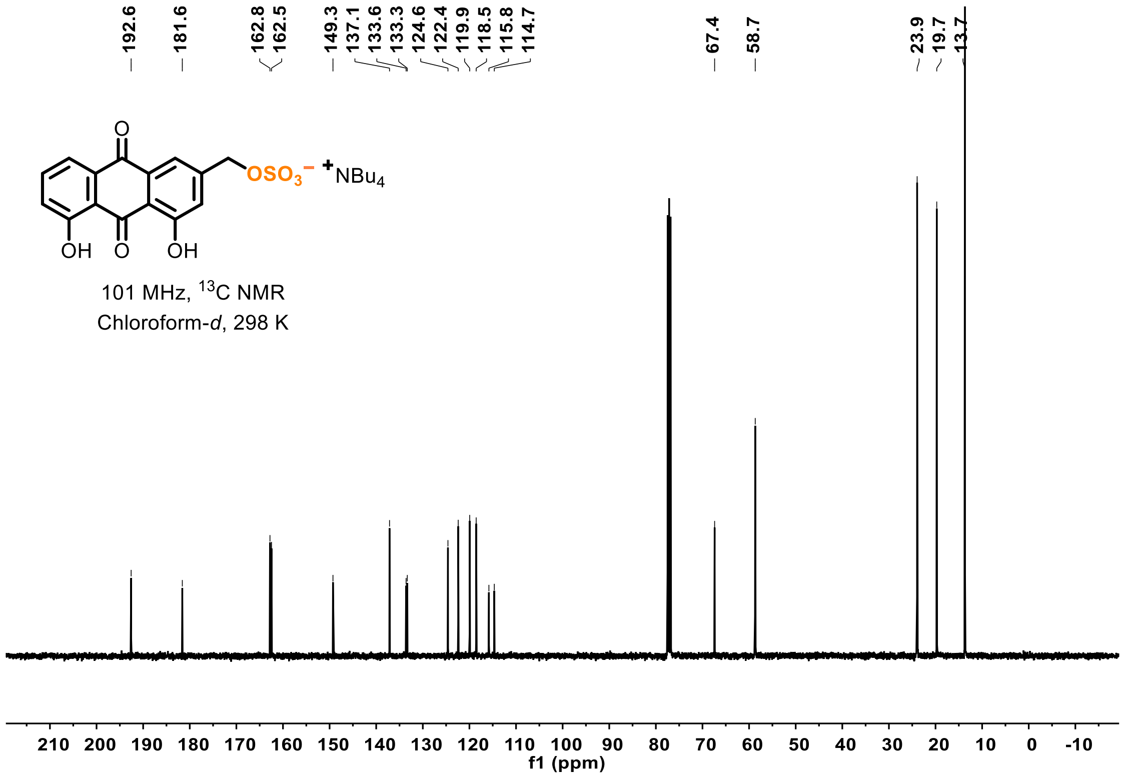

Tetrabutylammonium ospemifene sulfate (66)

^{1}H NMR of tetrabutylammonium ospemifene sulfate (66)

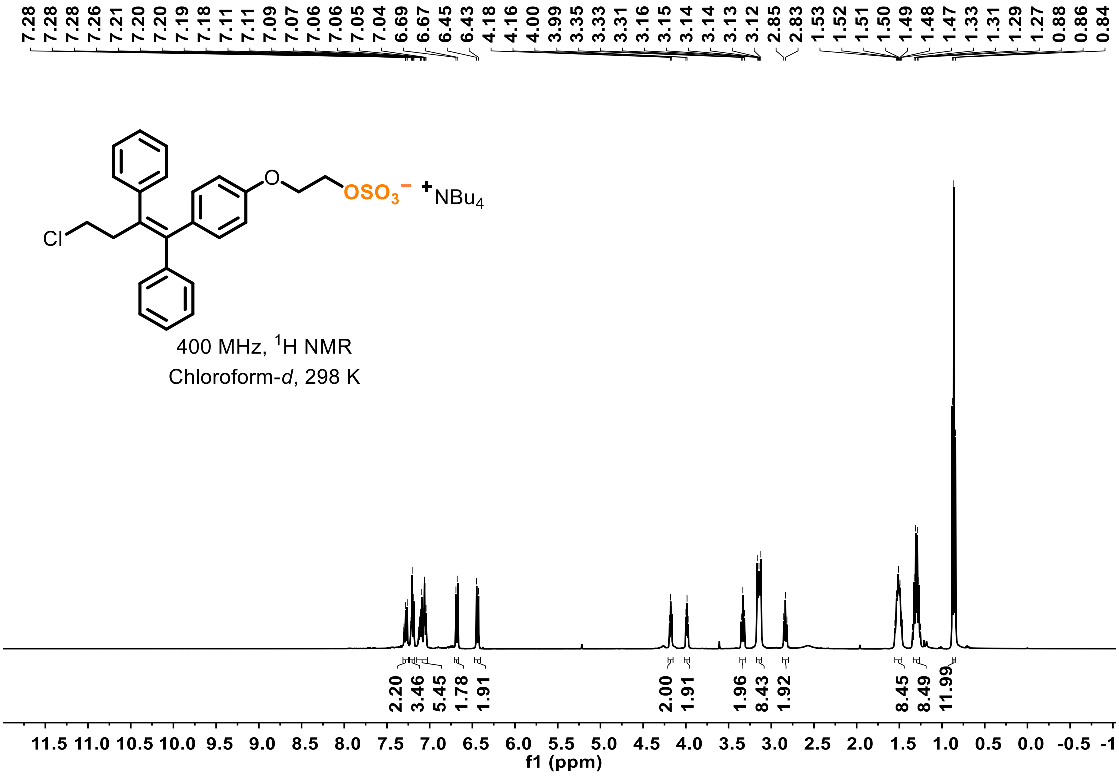

^{13}C NMR of tetrabutylammonium ospemifene sulfate (66)

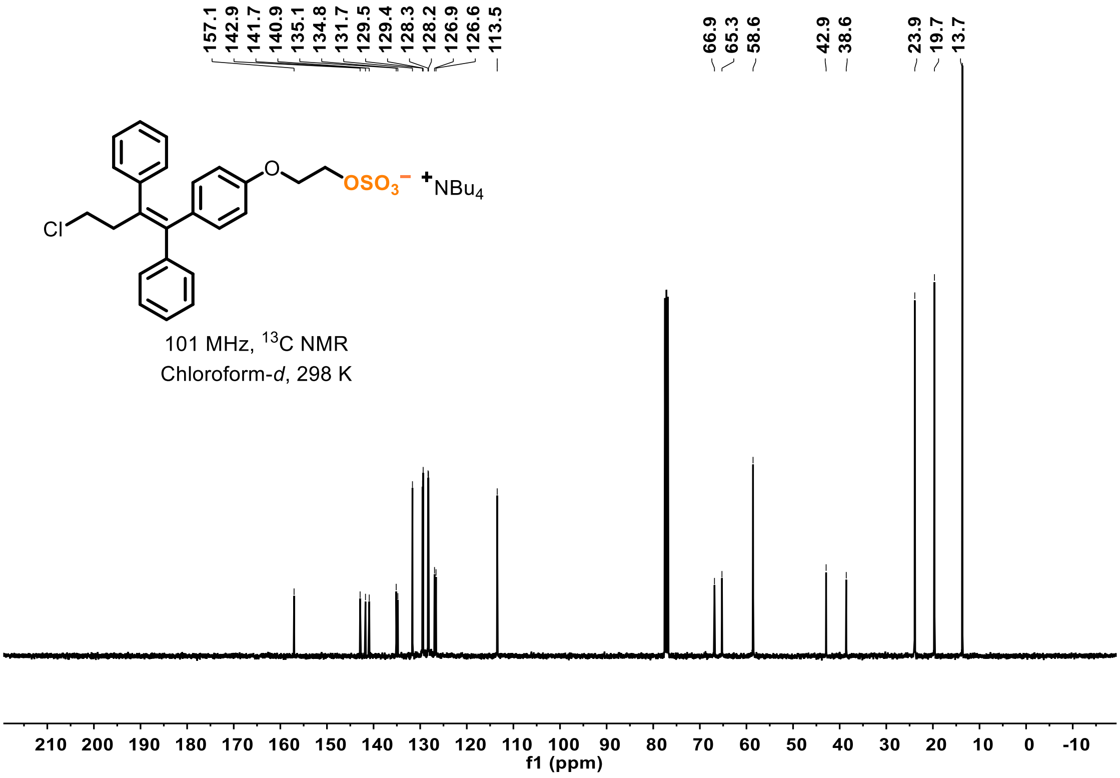

Tetrabutylammonium testosterone sulfate (67)

^{1}H NMR of tetrabutylammonium testosterone sulfate (67)

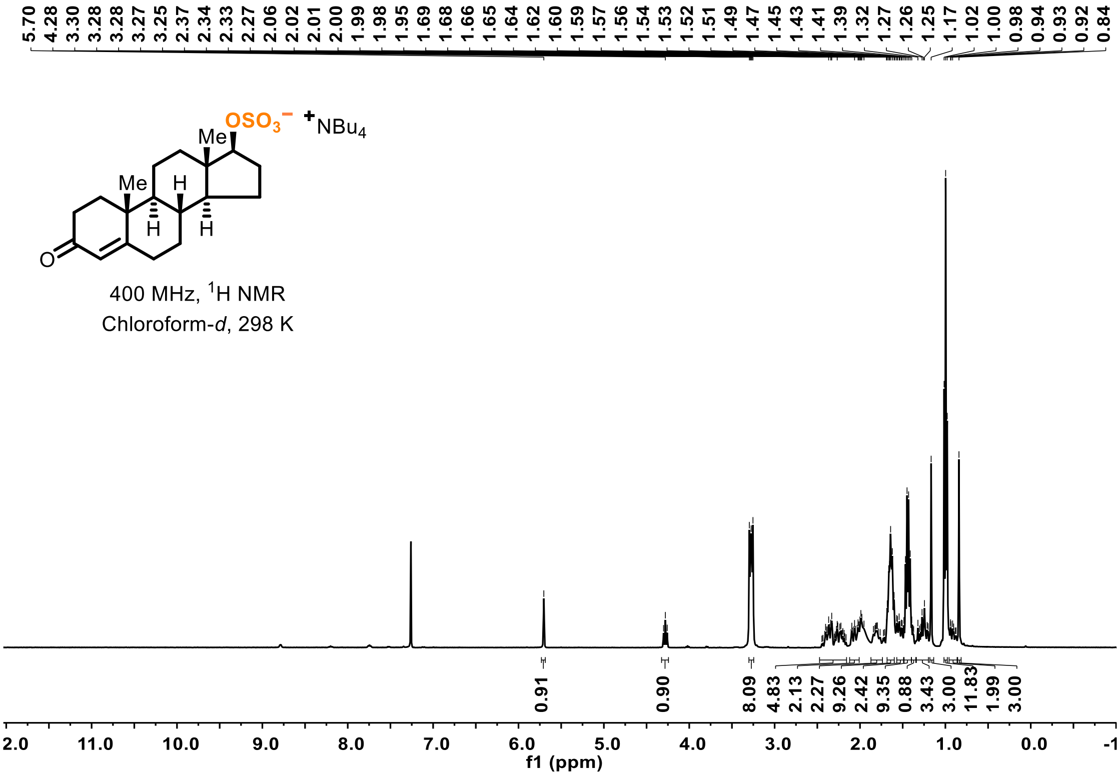

^{13}C NMR of tetrabutylammonium testosterone sulfate (67)

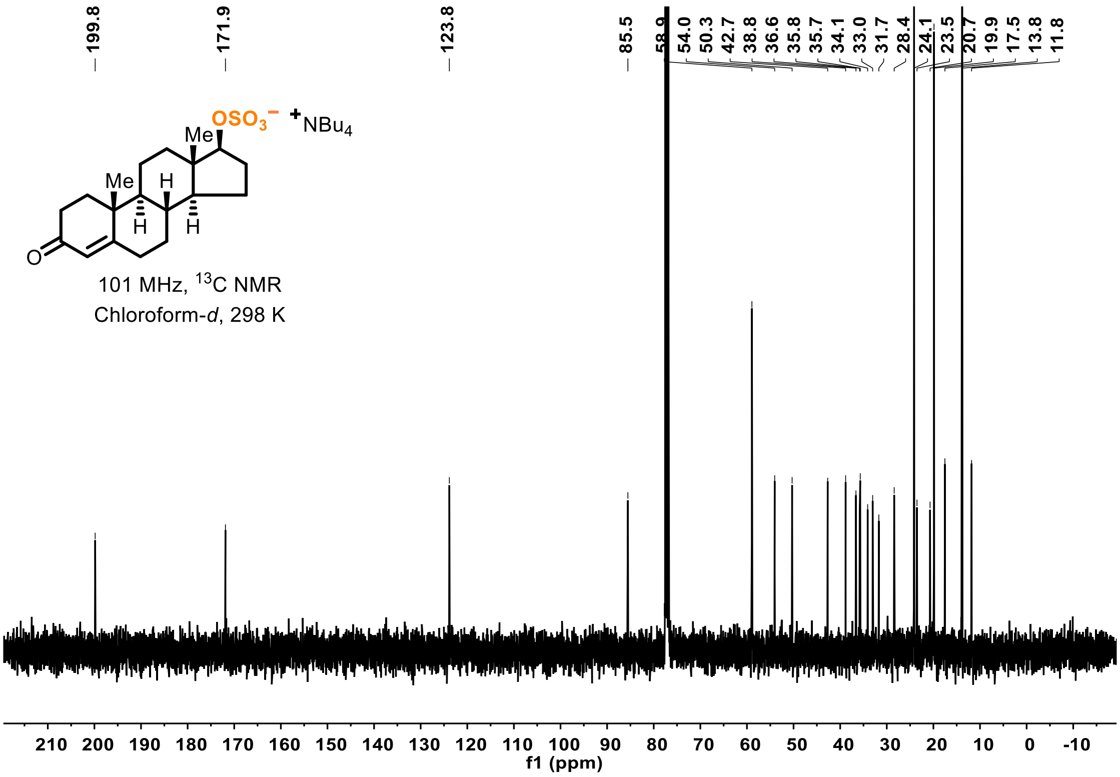

Tetrabutylammonium diosgenin sulfate (68)

^{1}H NMR of tetrabutylammonium diosgenin sulfate (68)

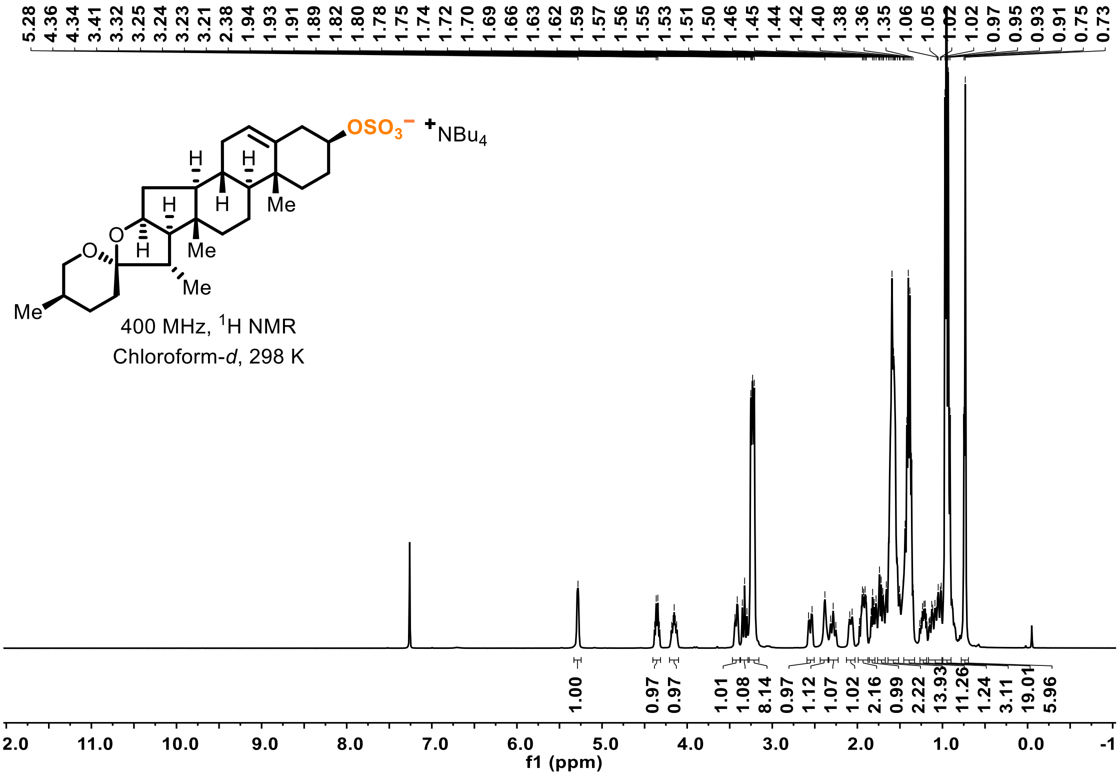

^{13}C NMR of tetrabutylammonium diosgenin sulfate (68)

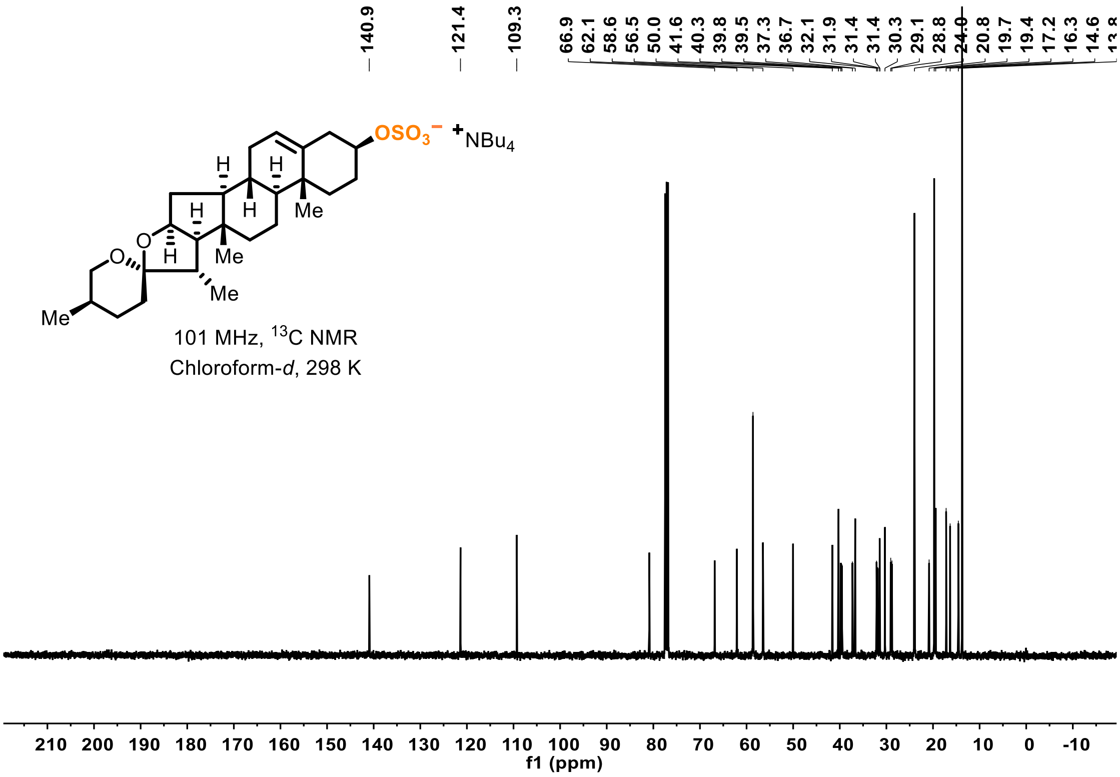

Tetrabutylammonium cholesterol sulfate (69)

^1H NMR of tetrabutylammonium cholesterol sulfate (69)

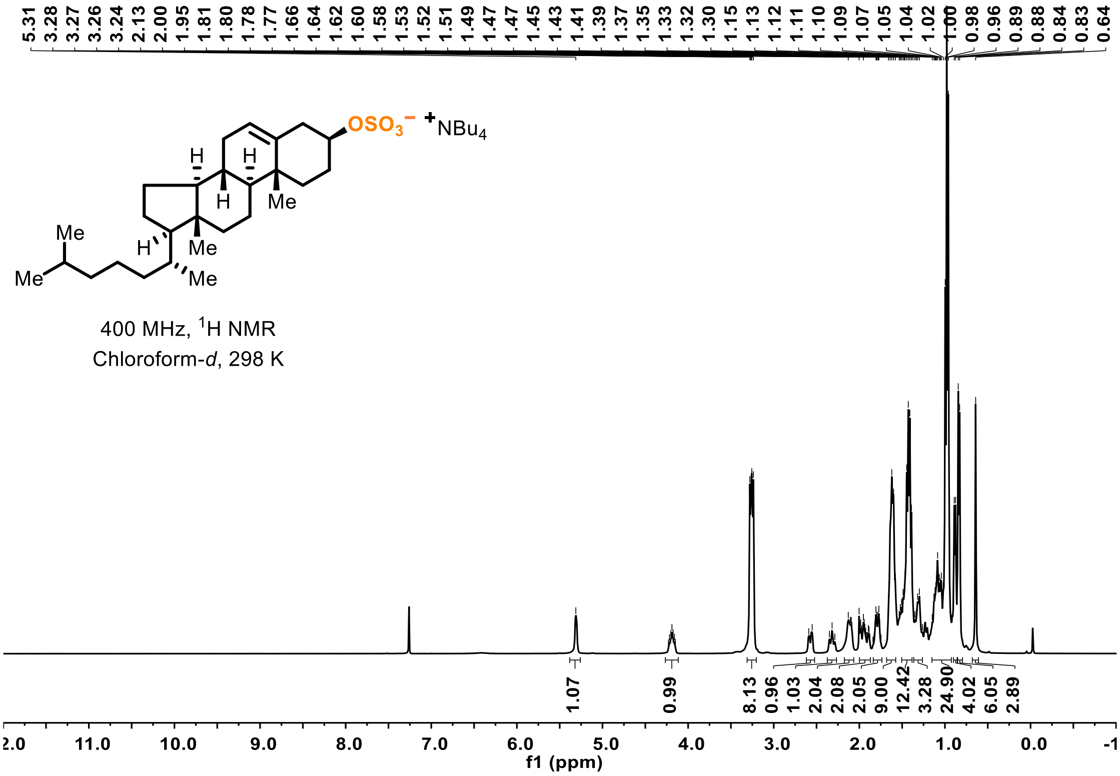

^{13}C NMR of tetrabutylammonium cholesterol sulfate (69)

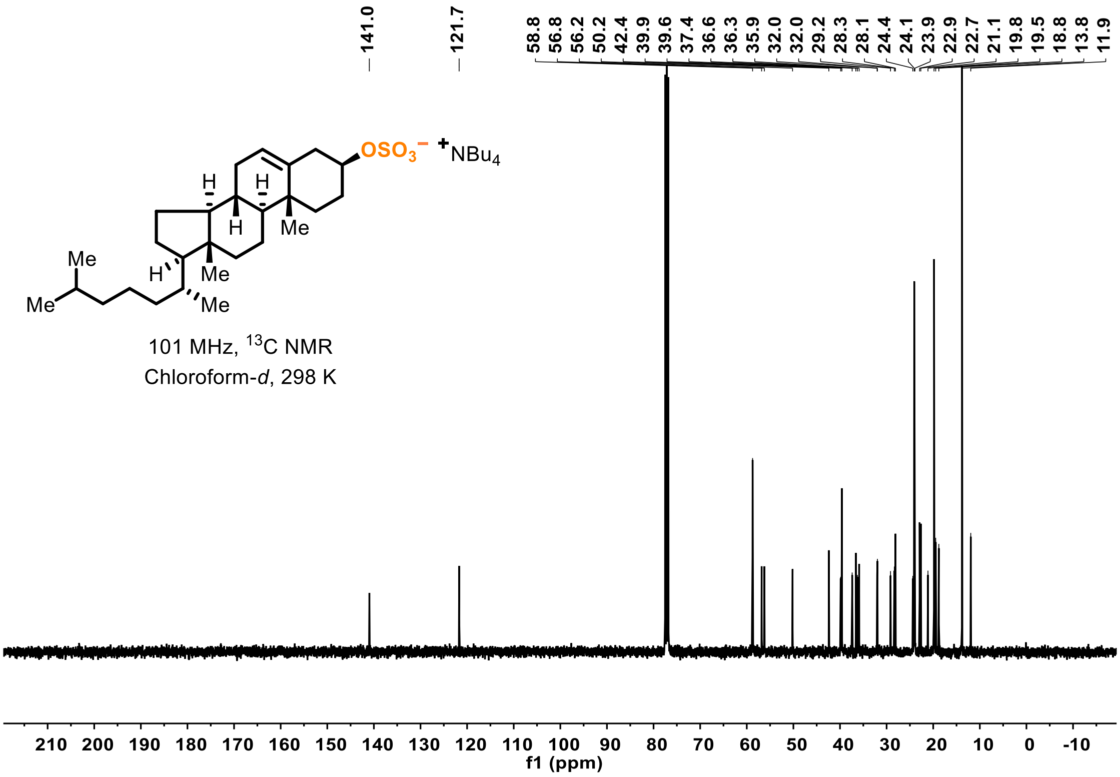

3-phthalimido-1-propanol methyl sulfate (1-B)

^{1}H NMR of 3-phthalimido-1-propanol methyl sulfate (1-B)

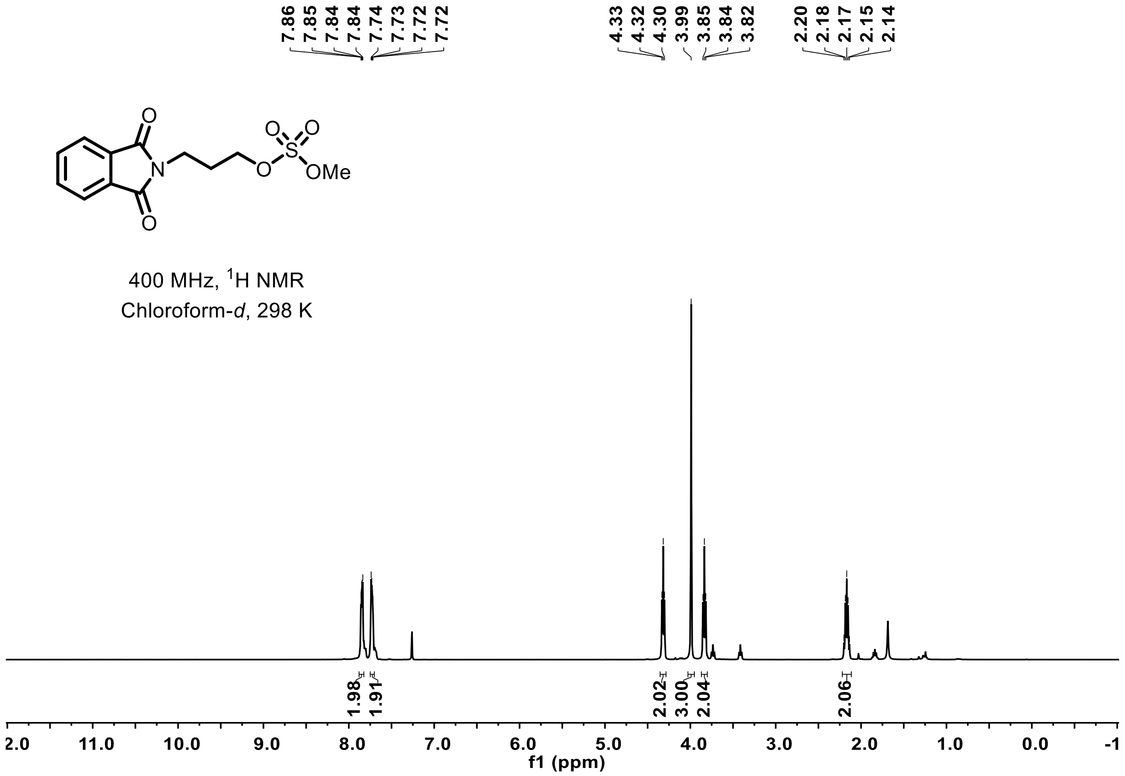

^{13}C NMR of 3-phthalimido-1-propanol methyl sulfate (1-B)

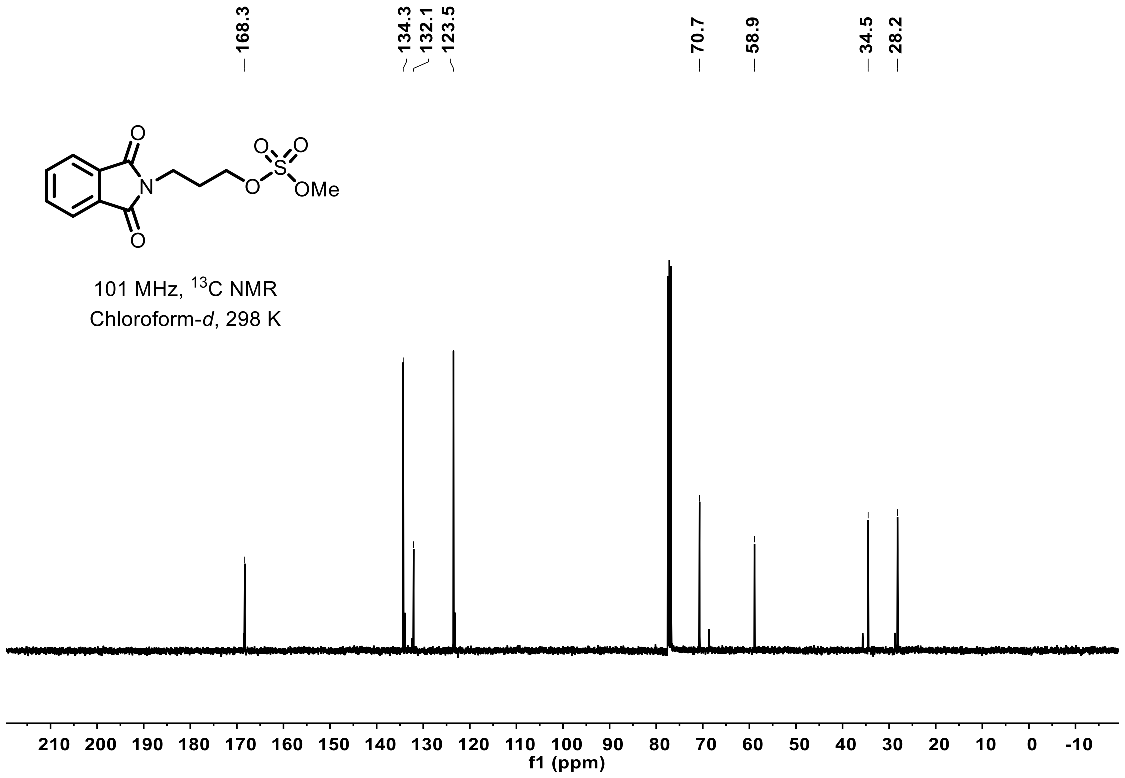

4-biphenylmethanol-^{18}O (^{18}O-70a)

^{1}H NMR of 4-biphenylmethanol-^{18}O (^{18}O-70a)

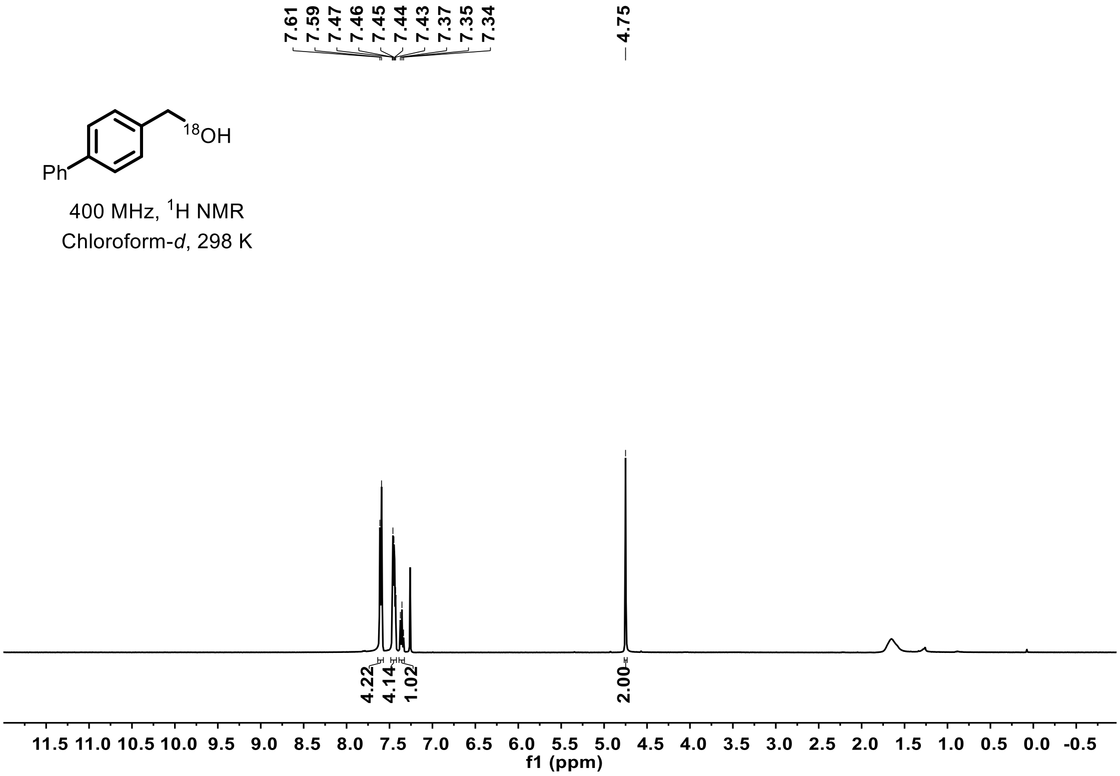

^{13}C NMR of 4-biphenylmethanol-^{18}O (^{18}O-70a)

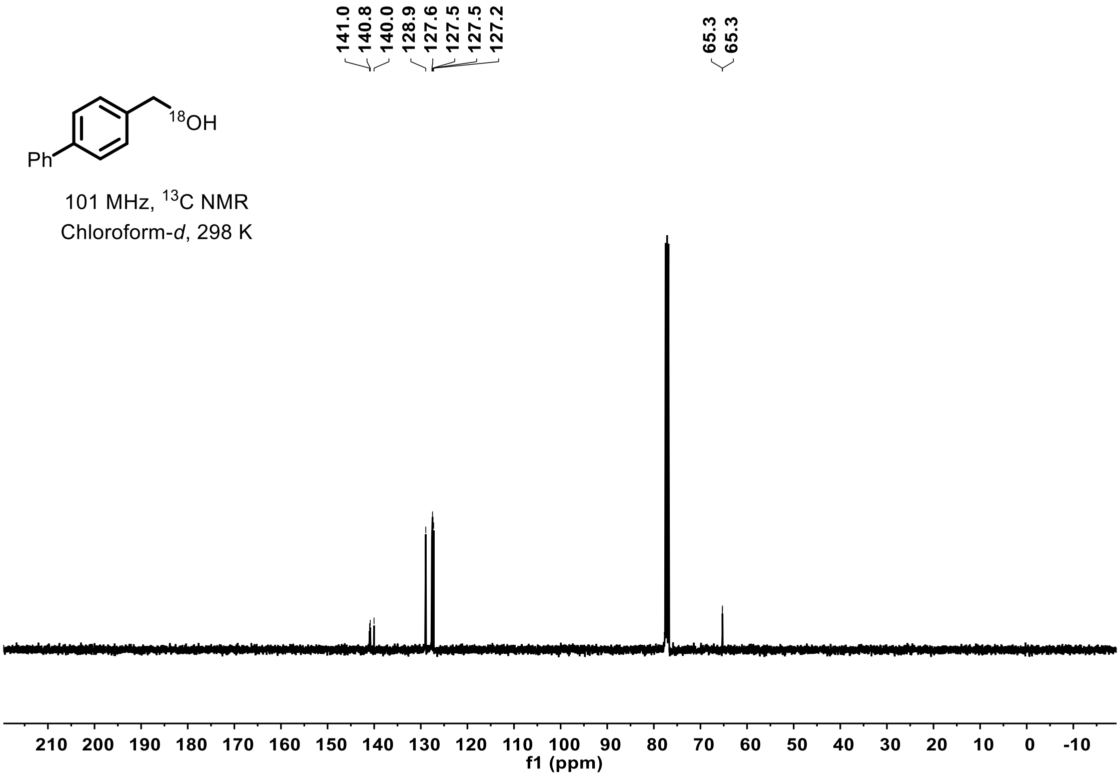

Tetrabutylammonium 4-biphenylmethanol-^{18}O sulfate (^{18}O-70)

^{1}H NMR of tetrabutylammonium 4-biphenylmethanol-^{18}O sulfate (^{18}O-70)

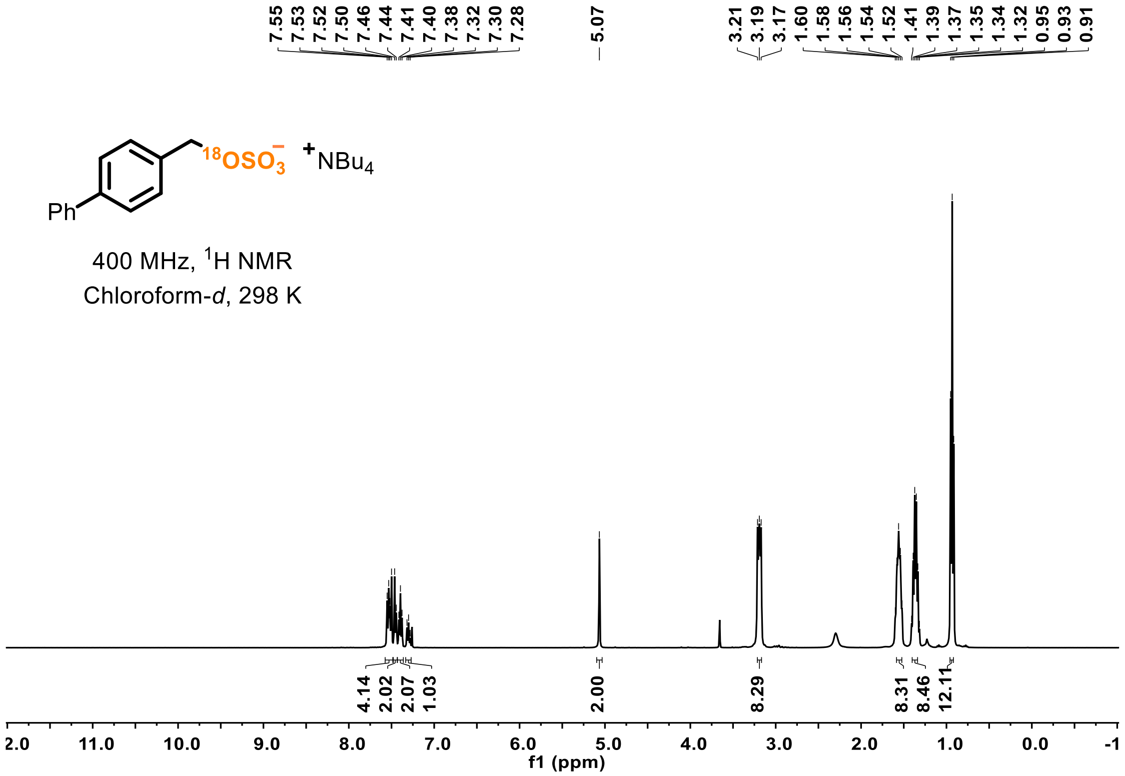

^{13}C NMR of tetrabutylammonium 4-biphenylmethanol-^{18}O sulfate (^{18}O-70)

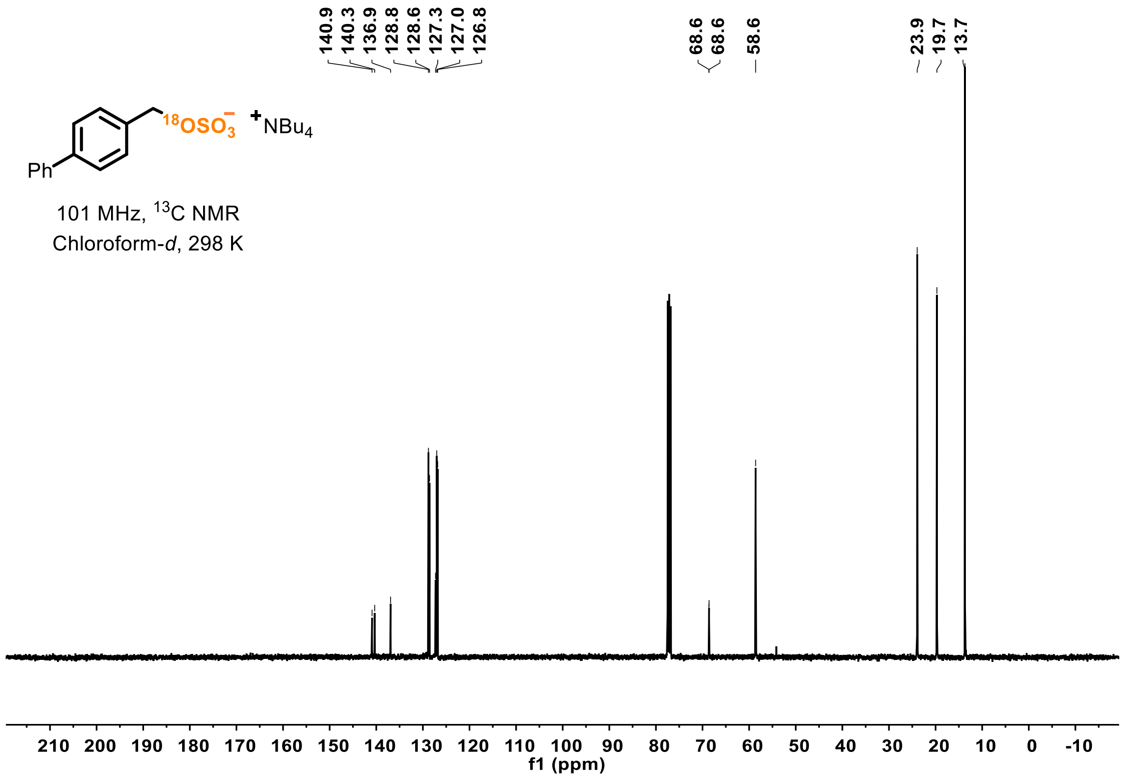

Supplementary References

1. Soulard, V., Villa, G., Vollmar, D. P. & Renaud, P. Radical deuteration with D_2O: Catalysis and mechanistic insights. *J. Am. Chem. Soc.* **140**, 155-158 (2018).2. Guo, J. *et al*. Rapid deoxyfluorination of alcohols with N-Tosyl-4-chlorobenzenesulfonimidoyl fluoride (sulfoxFluor) at room temperatur. *Chem. Eur. J.* **25**, 7259-7264 (2019).
3. Chen, Z., Fan, H., Yang, S., Bian, G. & Song, L. Chiral sensors for determining the absolute configurations of α-amino acid derivatives. *Org. Biomol. Chem***.** **16**, 8311-8317 (2018).
4. Montero Bastidas, J. R., Oleskey, T. J., Miller, S. L., Smith III, M. R. & Maleczka Jr., R. E. Para-selective, iridium-catalyzed C–H borylations of sulfated phenols, benzyl Alcohols, and anilines directed by ion-pair electrostatic interactions. *J. Am. Chem. Soc*. **141**, 15483-15487 (2019).
5. Yu, Q. *et al*. Synthesis of benzylic alcohols by decarboxylative hydroxylation. *Org. Lett.* **25**, 47-52 (2023).
6. Liang, A., Thakkar, J. N. & Desai, U. R. Study of physico-chemical properties of novel highly sulfated, aromatic, mimetics of heparin and heparan sulfate. *J. Pharm. Sci.* **99**, 1207–1216 (2009)..
7. Yamagishi, H. *et al*. Metal–organic nanotube with helical and propeller-chiral motifs composed of a C10-symmetric double-decker nanoring *J. Am. Chem. Soc.* **137**, 7628-7631 (2015).
